# RNA_Seq_ Based Transcriptional Profiling of *Pseudomonas aeruginosa* PA14 after Short- and Long-Term Anoxic Cultivation in Synthetic Cystic Fibrosis Sputum Medium

**DOI:** 10.1371/journal.pone.0147811

**Published:** 2016-01-28

**Authors:** Muralidhar Tata, Michael T. Wolfinger, Fabian Amman, Nicole Roschanski, Andreas Dötsch, Elisabeth Sonnleitner, Susanne Häussler, Udo Bläsi

**Affiliations:** 1 Department of Microbiology, Immunobiology and Genetics, Max F. Perutz Laboratories, Center of Molecular Biology, University of Vienna, Dr. Bohr-Gasse 9, 1030 Vienna, Austria; 2 Center for Integrative Bioinformatics Vienna, Max F. Perutz Laboratories, Center of Molecular Biology, University of Vienna, Dr. Bohr-Gasse 9, 1030 Vienna, Austria; 3 Department of Chromosome Biology, Max F. Perutz Laboratories, Center of Molecular Biology, University of Vienna, Dr. Bohr-Gasse 9, 1030 Vienna, Austria; 4 Free University Berlin, Institute of Animal Hygiene and Environmental Health, Robert-von-Ostertag-Str. 7–13, 14163 Berlin, Germany; 5 Department of Molecular Bacteriology, Helmholtz Center for Infection Research, Inhoffenstraße 7, 38124 Braunschweig, Germany; 6 Institute of Molecular Bacteriology, Twincore, Center for Experimental and Clinical Infection Research, Feodor-Lynen-Straße 7, 30625 Hannover, Germany; 7 Institute for Theoretical Chemistry, University of Vienna Währinger Straße 17, 1090 Vienna, Austria; East Carolina University School of Medicine, UNITED STATES

## Abstract

The opportunistic human pathogen *Pseudomonas aerugino*sa can thrive under microaerophilic to anaerobic conditions in the lungs of cystic fibrosis patients. RNA_Seq_ based comparative RNA profiling of the clinical isolate PA14 cultured in synthetic cystic fibrosis medium was performed after planktonic growth (OD_600_ = 2.0; P), 30 min after shift to anaerobiosis (A-30) and after anaerobic biofilm growth for 96h (B-96) with the aim to reveal differentially regulated functions impacting on sustained anoxic biofilm formation as well as on tolerance towards different antibiotics. Most notably, functions involved in sulfur metabolism were found to be up-regulated in B-96 cells when compared to A-30 cells. Based on the transcriptome studies a set of transposon mutants were screened, which revealed novel functions involved in anoxic biofilm growth.In addition, these studies revealed a decreased and an increased abundance of the *oprD* and the *mexCD-oprJ* operon transcripts, respectively, in B-96 cells, which may explain their increased tolerance towards meropenem and to antibiotics that are expelled by the MexCD-OprD efflux pump. The OprI protein has been implicated as a target for cationic antimicrobial peptides, such as SMAP-29. The transcriptome and subsequent Northern-blot analyses showed that the abundance of the *oprI* transcript encoding the OprI protein is strongly decreased in B-96 cells. However, follow up studies revealed that the susceptibility of a constructed PA14Δ*oprI* mutant towards SMAP-29 was indistinguishable from the parental wild-type strain, which questions OprI as a target for this antimicrobial peptide in strain PA14.

## Introduction

*Pseudomonas spp*. infects plants, nematodes and mammals. In humans the opportunistic pathogen *P*. *aeruginosa* can thrive under microaerophilic to anaerobic conditions in the lungs of patients suffering from cystic fibrosis [1], a genetic disorder caused by mutations in the *c*ystic *f*ibrosis *t*ransmembrane conductance *r*egulator (CFTR) gene. Mutations in the CFTR gene may result in formation of thick mucus in the respiratory airways, which leads to reduced oxygen availability [2].

In the absence of oxygen, *P*. *aeruginosa* can utilize nitrate, nitrite or nitrous oxide as terminal electron acceptors in an ATP generating pathway known as nitrate respiration [3]. In the absence of these compounds, energy can be generated *via* substrate level phosphorylation by arginine fermentation [4]. In addition, pyruvate can serve as an energy source to sustain long-term survival during anaerobiosis [5]. The transition between aerobic to anaerobic growth is regulated by the global transcriptional regulator Anr, which responds to oxygen limitation through an [4Fe-4S]^2+^ cluster [6]. Regulation by Anr alone is required and sufficient for the survival of *P*. *aeruginosa* during anoxic growth utilizing pyruvate or arginine as an energy source. In contrast, the expression of genes, encoding enzymes for the denitrification pathway requires in addition the transcriptional regulators Dnr and the nitrate responsive two-component system NarX/NarL [7].

*P*. *aeruginosa* is notorious for its high level intrinsic resistance towards several antibiotics. Intrinsic resistance can result from a reduced permeability to given antibiotics [8]. In addition, antibiotic detoxifying mechanisms can contribute to intrinsic resistance such as chromosomally encoded antibiotic-inactivating enzymes [9], multidrug resistance (MDR) efflux pumps [10] or target-protecting factors such as chromosomally encoded Qnr proteins [11]. In addition, *P*. *aeruginosa* tolerance to antibiotics can be affected by phenotypic variation [12], the formation of specialized persister cells [13], by quorum sensing [14], and by the overproduction of the matrix polysaccharide alginate [15]. Moreover, the ability of *P*. *aeruginosa* to form robust biofilms during anoxic growth contributes to an increased antibiotic tolerance [16].

Biofilm formation is a complex process, which involves different stages; initial attachment of the cells to the surface [17], micro colony formation [18], formation of the extracellular matrix and biofilm maturation [18]. Several transcriptome studies with *P*. *aeruginosa* revealed regulatory mechanisms involved in biofilm formation and adaptation. In the majority of these studies, *P*. *aeruginosa* strains were aerobically grown in LB broth [19–24]. In contrast, Tielen *et al*. [25] compared the transcriptomes of *P*. *aeruginosa* O1 (PAO1) after anoxic growth in artificial urine medium and in 10-fold diluted LB medium, which revealed regulatory and metabolic networks for the adaptation of *P*. *aeruginosa* biofilms to urinary tract-like conditions. Only one study was reported wherein a comparative transcriptome analysis was performed with PAO1 after growth in the presence and absence of oxygen in minimal medium [26]. Eichner *et al*. identified PAO1 genes that are predominantly expressed during hypoxic growth in artificial cystic fibrosis sputum medium [27]. In addition, several proteome studies were conducted with *P*. *aeruginosa* after aerobic and anoxic growth in LB broth [28–33] to assess alterations in the bacterial protein content.

In the present study, we performed RNA_Seq_ based comparative RNA profiling of the clinical isolate PA14 cultured in *s*ynthetic *c*ystic *f*ibrosis *m*edium (SCFM) [34] after planktonic growth in the presence of oxygen (OD_600_ = 2.0), 30 min after shift to anaerobiosis and after anaerobic biofilm growth for 96h with the aim to unravel functions required for sustained anoxic biofilm formation. In addition, we sought to analyze the differential abundance of known antibiotic-resistance genes during planktonic growth (OD_600_ = 2.0) and after anaerobic biofilm growth for 96h with the objective to shed more light on the increased tolerance to antibiotics of anoxic biofilms.

## Materials and Methods

### Bacterial strains and growth conditions

The clinical isolate of *Pseudomonas aeruginosa*, PA14, was used in all experiments. The synthetic cystic fibrosis sputum medium was prepared a described by Palmer *et al*. [34] except that the concentration of FeSO_4_·7H_2_O was increased to 100 μM and that of KNO_3_ to 100 mM. This was done to allow for increased anaerobic biofilm formation after 96h, which was required for the extraction of sufficient amounts of RNA for subsequent RNA_Seq_ analysis.

### RNA_Seq_ library construction and sequence analysis

Total RNA was prepared from two biological replicates of PA14 after planktonic growth in the presence of oxygen (OD_600_ = 2.0; P), 30 min after shift to anaerobiosis (A-30) and after anaerobic biofilm growth for 96h (B-96). P: 25 ml of SCFM were inoculated at an initial OD_600_ of 0.05 and grown under aeration (shaking at 165 rpm) at 37°C. The cells were harvested for RNA preparation at an OD_600_ of 2.0. A-30: 25 ml of SCFM were inoculated as described above. When the cells reached an OD_600_ of 0.4, cultivation was continued for 30 min in an anaerobic chamber and the cells were harvested thereafter for RNA preparation. Under these conditions anaerobiosis was confirmed by employing an anaerobic indicator strip (Oxoid). B-96: 5 ml polypropylene tubes were filled with 1 ml of SCFM medium, which was inoculated with PA14 (OD_600_ = 0.05). The cultures were then incubated for 96 hours at 37°C in a 2.5-liter anaerobic jar containing a gas pack (AN25; AnaeroGen, Oxoid, United Kingdom). The total content of the polypropylene tube was used for RNA preparation. Total RNA from all samples was isolated using the TRIzol reagent (Ambion) according to the manufacturer’s instructions. The samples were DNase I treated, followed by phenol-chloroform-isoamyl alcohol extraction and ethanol precipitation. The MICROBExpress Kit (Ambion) was used to deplete rRNA from total RNA samples. Libraries were constructed using NEBNext® Ultra™ Directional RNA Library Prep Kit from Illumina. 100 bp single end sequence reads were generated using the Illumina HiSeq 2000 platform at the Vienna Biocenter Campus Science Support Facility (http://www.csf.ac.at). Quality control assessment of the raw reads using FastqQC (http://www.bioinformatics.babraham.ac.uk/projects/fastqc/) obviated further pre-processing. Sequencing adapter removal was performed with cutadapt [35]. Mapping of the samples against the PA14 reference genome (NCBI accession number NC_008463.1) was performed with Segemehl [36] with default parameters. Reads mapping to regions annotated as either rRNA or tRNA were discarded from all data and ignored for all follow up analysis steps. The mapped sequencing data were prepared for visualization using the ViennaNGS tool box and visualized within the UCSC Genome Browser [37]. Reads per genes were counted using BEDTools [38] and the Refseq annotation of *P*. *aeruginosa* (NC_008463.1). Differential gene expression analysis was performed with DESeq (version 1) [39]. All genes with a fold change greater than 2.5 and a multiple testing adjusted p-value below 0.05 were considered to be significantly modulated. The raw sequencing reads were deposited in the NCBI sequence read archive (SRA) as a study under the accession number SRP062593.

### RNA isolation and Northern-blot analysis

Total RNA was isolated from the samples using the TRIzol Reagent (Ambion). For Northern- blot analysis, 8 μg of total RNA were heated at 65°C for 5 minutes in loading buffer (5 mM EDTA, 0.025% xylene cyanol, 0.025% bromophenol blue dissolved in formamide) and resolved on 8% polyacrylamide/8 M urea gels. The RNA was transferred onto Hybond N+ nylon membranes (GEHealthcare) using a semi-dry electroblotting apparatus (Trans blot SD cell, BioRad) set at 13 V for 45 min and then UV-cross-linked. All DNA oligonucleotide probes (S1 Table) were 5'-end labeled with [γ-^32^P] ATP using T4 polynucleotide kinase. The respective [^32^P]-labelled oligonucleotides were heated at 95°C for 2 min, added to the pre-hybridized membrane and incubated at 52°C overnight. Pre-hybridization and hybridization were performed in Roti®Hybrid Quick (Carl Roth, Karlsruhe, Germany) supplemented with 0.1 mg/ml salmon sperm DNA. As loading control, a 5S rRNA-specific oligonucleotide was used (S1 Table). The hybridization signals were visualized using a PhosphorImager (Molecular Dynamics).

### Determination of MICs

Minimum inhibitory concentrations (MICs) for different antibiotic (Table 1) were determined using the broth micro-dilution method. Antimicrobial agents were prepared in serial dilutions in SCFM medium within the dilution range stated in Table 1. Briefly, for aerobic growth, the bacterial cultures were grown at 37°C in SCFM with an agitation rate of 165 rpm to an OD_600_ of 2.0. Then, different concentrations of the antibiotics resuspended in 100 μl SCFM were added to 200 μl aliquots of bacterial cultures and incubation was continued for 14h in 96 well plates. For determination of the MICs in anoxic biofilms, 200 μl bacterial cultures were grown anaerobically in SCFM for 96 hours in 96 well plates using the anaerobic chamber. After 96 hours different concentrations of the antibiotics resuspended in 100 μl SCFM were added and incubation was continued for 14h. The MICs were determined as the lowest concentration of each antimicrobial agent that inhibited growth.

**Table 1:** MICs of different antibiotics for B-96 cells versus P cells.

Class	Antibiotic	Range tested (μg/ml)	MIC (μg/ml)B-96 vs P
Macrolides	Azythromycin	256–0.25	>256 vs 2
Quinolones	Ciprofloxacin	32–0.031	>32 vs 4
Quinolones	Norfloxacin	256–0.25	>256 vs 16
Polymyxins	Colistin	256–0.25	16 vs 2
Aminoglycosides	Gentamycin	256–0.25	64 vs 0.5
Carbapenems	Meropenem	32–0.031	>4 vs 0.25
Tetracycline	Tetracycline	256–0.25	>256 vs 32

### Biofilm assays

A static crystal violet assay [40] was used to assess biofilm formation of PA14 and of different transposon mutants thereof after anoxic growth. The cultures were inoculated as described above and incubated for 96h. Then, the contents of tubes were removed and washed 3 times with water and air dried. The tubes were stained with 1ml of 0.1% (w/v) crystal violet and incubated at room temperature for 10 min. The tubes were washed and air dried. The stain attached to the tubes was solubilized using 95% ethanol. Biofilm formation was assessed by measuring the optical density of each sample at a wavelength of 595 nm. The results of 3 individual experiments were averaged.

### Construction of a PA14 *oprI* deletion mutant and susceptibility test towards SMAP-29

An *oprI* inframe deletion mutant was constructed by homologous recombination [6]. Briefly, the upstream (703 bp) and downstream (715 bp) flanking sequences of the *oprI* gene were PCR-amplified using the oligonucleotide pair F95/H95 (upstream region) and G95/I95 (downstream region) (S1 Table). Recombinant PCR was employed to generate a DNA fragment containing the in frame deletion in *oprI*. The fragment was cloned into the KpnI and XbaI sites of plasmid pME3087 [6]. Sequencing of the resulting plasmid verified the deletion between nucleotides 2.362.200–2.362.540 (PA14 genome coordinates). The plasmid was transformed into PA14 and chromosomally integrated through selection for tetracycline resistance as previously described [6]. Double crossover mutants were then selected for the loss of plasmid (tetracycline sensitivity). The deletion of the *oprI* coding region was confirmed by PCR.

PA14 and PA14Δ*oprI* strains were grown aerobically in LB medium. Approximately 1 x 10^5^ cells were treated with different concentrations of SMAP-29 for 3h. Serial dilutions were then plated on LB agar plates and the CFU´s were determined after overnight growth at 37°C.

### Construction of plasmids pTLoprI and pTLoprD

To construct the translational *oprI*::*lacZ* fusion gene, a 261bp fragment (nt -225 to nt +36 with regard to the A (+1) of the start codon of *oprI*) including the *oprI* promoter [41] was amplified by PCR using the oligonucleotide pair K99/M99 (S1 Table) and chromosomal DNA of strain PA14 as template. The PCR fragment was cleaved with EcoRI and PstI and then ligated into the corresponding sites of plasmid pME6015 [42], abutting the 12^th^ codon of *oprI* to the 8^th^ codon of the *lacZ* gene.

For construction of of the *oprD*::*lacZ* chimeric gene, a 461-bp fragment (nt -440 to nt +21 with regard to the A(+1) of start codon of *oprD*) including the *oprD* promoter [43] was amplified by PCR using the oligonucleotide pair F112/H112 (S1 Table) and chromosomal DNA of strain PA14 as template. The PCR fragment was cleaved with EcoRI and PstI and then ligated into the corresponding sites of plasmid pME6015 [42], abutting the 7^th^ codon of *oprD* to the 8^th^ codon of *lacZ*.

### RT-qPCR

Total RNA was isolated from P- and B-96 cells as described above for the RNA_Seq_ analyses. Total RNA from M-96 cells was isolated after growth of the culture under aerobic / microaerophilic conditions without shaking for 96 hours. For cDNA synthesis, 1 μg RNA template was mixed with 0.5 μg of random primers (Promega). The mixture was then treated at 65°C for 5 min, followed by 5 min incubation on ice. cDNA synthesis was performed with AMV reverse transcriptase (Promega) according to the instructions of the manufacturer. 5μl of 8-fold diluted cDNA was used as a template for PCR performed with 5 x HOT FIREPol EvaGreen® qPCR Mix Plus (Medibena). Three biological replicates and three technical replicates were used for each experiment. The primers (S1 Table) were designed with Primer 3 software (http://frodo.wi.mit.edu/primer3). The transcript levels of the *rpoD* gene were used for normalization [44]. Changes in the mRNA levels were estimated as previously described [45].

### β-galactosidase assay

Strain PA14 harbouring plasmids pTLoprI and pTLoprD, respectively, was grown at 37°C in SCFM in the presence of 100 μg/ml tetracyline. Overnight cultures were diluted to an OD_600_ 0.05 in fresh medium and allowed to grow either to an OD_600_ of 2.0 (P) or under anoxic conditions for 96h (B-96). The cells were then permeabilized with 5% toulene and the β-galactosidase activity was determined as described [46].

## Results and Discussion

### Up-regulated pathways at 30 min after anaerobic shift and after 96h of anoxic biofilm formation

**Fig 1:**
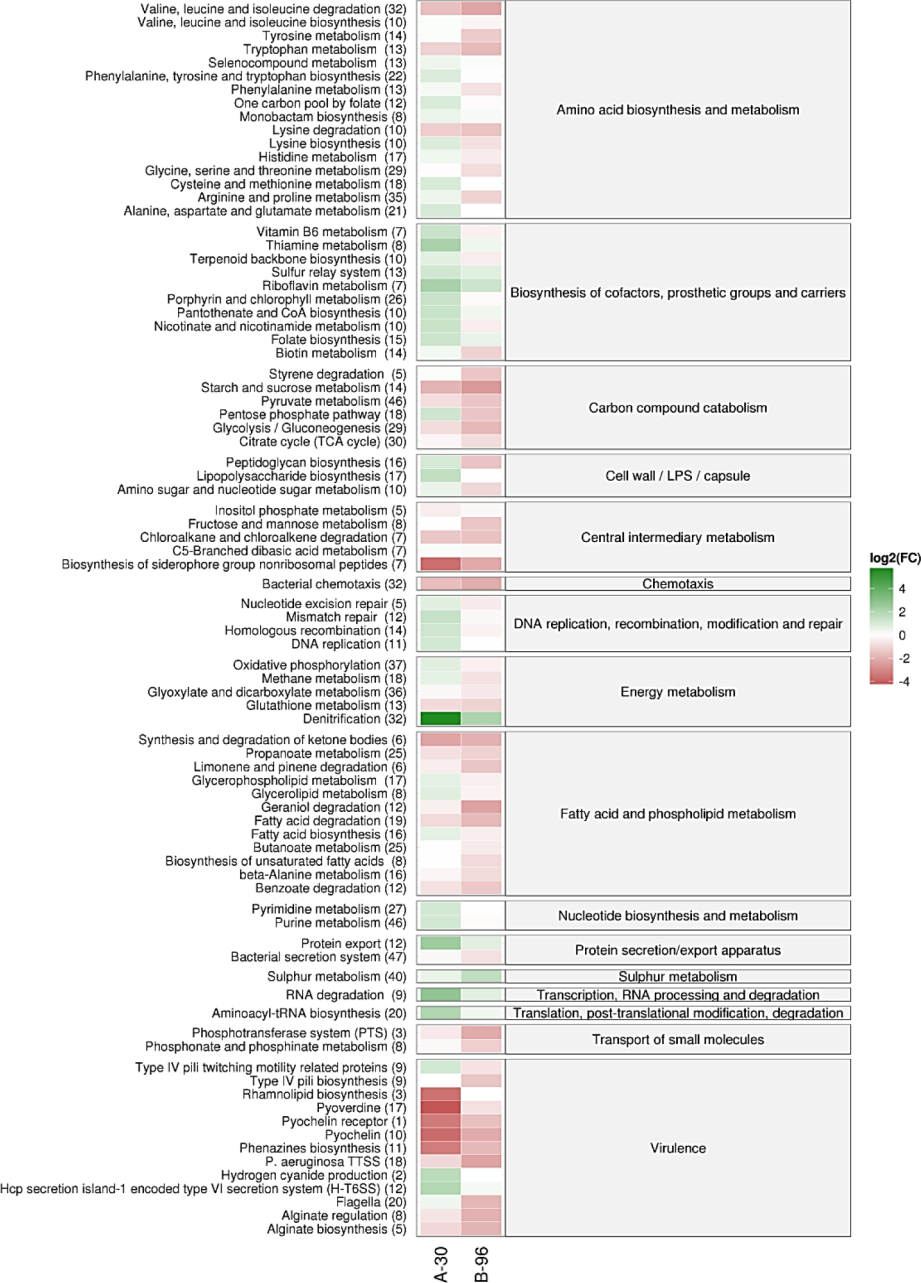
Meta-analysis of normalized expression of differentially abundant transcripts under the conditions A-30 and B-96 when compared with condition P. The genes are grouped into the corresponding pathways (http://www.kegg.jp/kegg-bin/show_organism?org=pau). For each group the overall behavior was summarized by the averaged log_2_ fold change of its significantly modulated members. The column denotes A-30 versus P and B-96 versus P, respectively. The color code shown in the scale at the right denotes log_2_-fold changes. Red indicates an overall decrease and green indicates an overall increase in the mRNA levels of genes in a particular pathway. The numbers of genes within each group are indicated by the numbers given in parenthesis.

First, we compared the PA14 transcriptomes after a shift for 30 min to anaerobiosis (A-30) and after 96h of anoxic biofilm formation (B-96) with planktonically growing cells (P; OD_600_ = 2.0). All genes, annotated in the NCBI database were included in the differential gene expression analysis. A p-value (adjusted for multiple testing) of 0.05 was set as a threshold for significance and the change in abundance (fold-change) had to exceed +/-2.5 for a given transcript in order to be considered differentially abundant. When compared with condition P, 1843 transcripts were found to be differentially abundant under the conditions A-30 and B-96, whereby with 1500 transcripts a significant overlap was observed between A-30 and B-96. Transcripts found to be down-regulated under the conditions A-30 and B-96 or both with respect to P (Fig 1; Table A in S2 Table) were not included in our quest for functions impacting on sustained anoxic biofilm formation in SCFM.

To identify functional classes of genes / pathways that are up-regulated under both conditions, A-30 and B-96 when compared with P, all log_2_ transformed fold-changes of significantly modulated genes belonging to previously characterized pathways (http://www.kegg.jp/kegg-bin/show_organism?org=pau) were compared. The following up-regulated pathways were identified under the conditions A-30 and B-96 when compared with P.

#### Monobactam biosynthesis

Transcripts encoding proteins involved in monobactam biosynthesis are significantly more abundant. (Fig 1; S2 Table). Monobactam synthesis is required for balancing peptidoglycan synthesis [47] and has also been suggested to have antibacterial effects [48]. The up-regulation of the monobactum biosynthesis pathway during anaerobiosis could therefore play a role in survival of *Pseudomonas* in competitive environments.

#### Biosynthesis of cofactors

Transcripts encoding functions required for the biosynthesis of cofactors, prosthetic groups and carriers are significantly more abundant (Fig 1; S2 Table), indicating their requirement in numerous anaerobic oxidation and reduction reactions required for anaerobic growth [49].

#### Energy metabolism

During anoxic growth, *P*. *aeruginosa* can use nitrate as a terminal electron acceptor [3]. As anticipated, the transcripts encoding enzymes required for the denitrification pathway, *viz* the nitrate reductase encoding *nar*-operon, the nitrite reductase encoding *nir*-operon, the NO reductase encoding *nor*-operon, the nitrous dioxide reductase encoding *nos-*operon and the regulator *nosR*, are highly abundant (Fig 1; S2 Table). The transcripts encoding proteins required for molybdenum cofactor (MoCo) synthesis, which is required for nitrate reductase activity are also significantly more abundant [50]. The transcripts coding for the succinate dehydrogenase complex (*sdhABCD*), which is required for electron tunneling during nitrate respiration [51], and the *atpIBEFHAGFC* cluster transcripts encoding the only ATP synthase complex of *P*. *aeruginosa* were also significantly increased (Fig 1; S2 Table).

#### Protein secretion

The observed increase of transcripts encoding proteins for export apparatuses (Fig 1; S2 Table) might be attributed to their role in release of cellular material as a result of metabolic turnover, cell-cell communication, virulence, antibiotic resistance etc. [52].

#### Sulphur metabolism

The majority of transcripts encoding functions involved in sulfur assimilation are up-regulated in A-30 and B-96 cells when compared with condition P. However, it should be noted that these transcripts were significantly more abundant in B-96 cells when compared with A-30 cells (Fig 1; S2 Table). Bacteria acquire sulfur through the sulfate assimilation pathway leading to the production of sulfide, which is then incorporated into sulfur containing organic molecules [53]. Sulfur is essential for cofactor synthesis required for the anaerobic regulators Anr and Dnr as well as for several enzymes involved in denitrification [54, 55].

Since genes involved in sulfur metabolism are known to be up-regulated in response to sulfate starvation [56] and as the B-96 cells were incubated without addition of fresh medium, it was possible that changes in the abundance of the transcripts resulted from sulfur starvation rather than from the anoxic environment. To distinguish between these possibilities, we monitored the expression levels of one representative transcript, *msuE*, in P and B-96 cells. As shown in S1 Fig, when compared with B-96 cells the *msuE* transcript was not up-regulated in M-96 biofilms after oxygenic / microaerophilic growth for 96h without shaking, indicating that the observed up-regulation of the genes involved in sulfur metabolism is indeed induced during prolonged anaerobiosis.

#### Transcription / Translation / RNA processing

Transcripts encoding functions required for RNA processing and in particular transcripts encoding tRNA synthases were also significantly more abundant (Fig 1; S2 Table) during anaerobiosis.

Next, we compared our data set with a recent transcriptome analysis [27] and with previous proteome data [30, 33], all of which were performed during anaerobic growth. Most of the marker transcripts deemed to be required for metabolic adaptation to the hypoxic lung environment [27] showed either no difference or were even less abundant in our data set. Only the transcript abundance of *accB* (A-30), *idh* (B-96) and *nuoA* (B-96) were likewise increased under the conditions examined here. When compared with the proteome analyses of Wu *et al*. [30] and Platt *et al*. [33], we detected 384 and 60 concurrent transcripts with differential abundance (marked in Table A in S2 Table), respectively. The variations observed with the omics studies can be most likely ascribed to the different conditions and media used under / in which the experiments were performed. Nevertheless, the up-regulation of the *mreB* transcript, those for molybdenum cofactor synthesis, the *sdhABCD*, the *atpIBEFHAGFC*, the *nar*, the *nir* and the *nos* operon transcripts in A-30 cells and/or in B-96 cells concurred with proteome studies, wherein the levels of the corresponding proteins were found to be increased during anaerobic growth [30, 33].

### Up-regulated transcripts in B-96 biofilms that impact on anoxic biofilm formation

**Fig 2:**
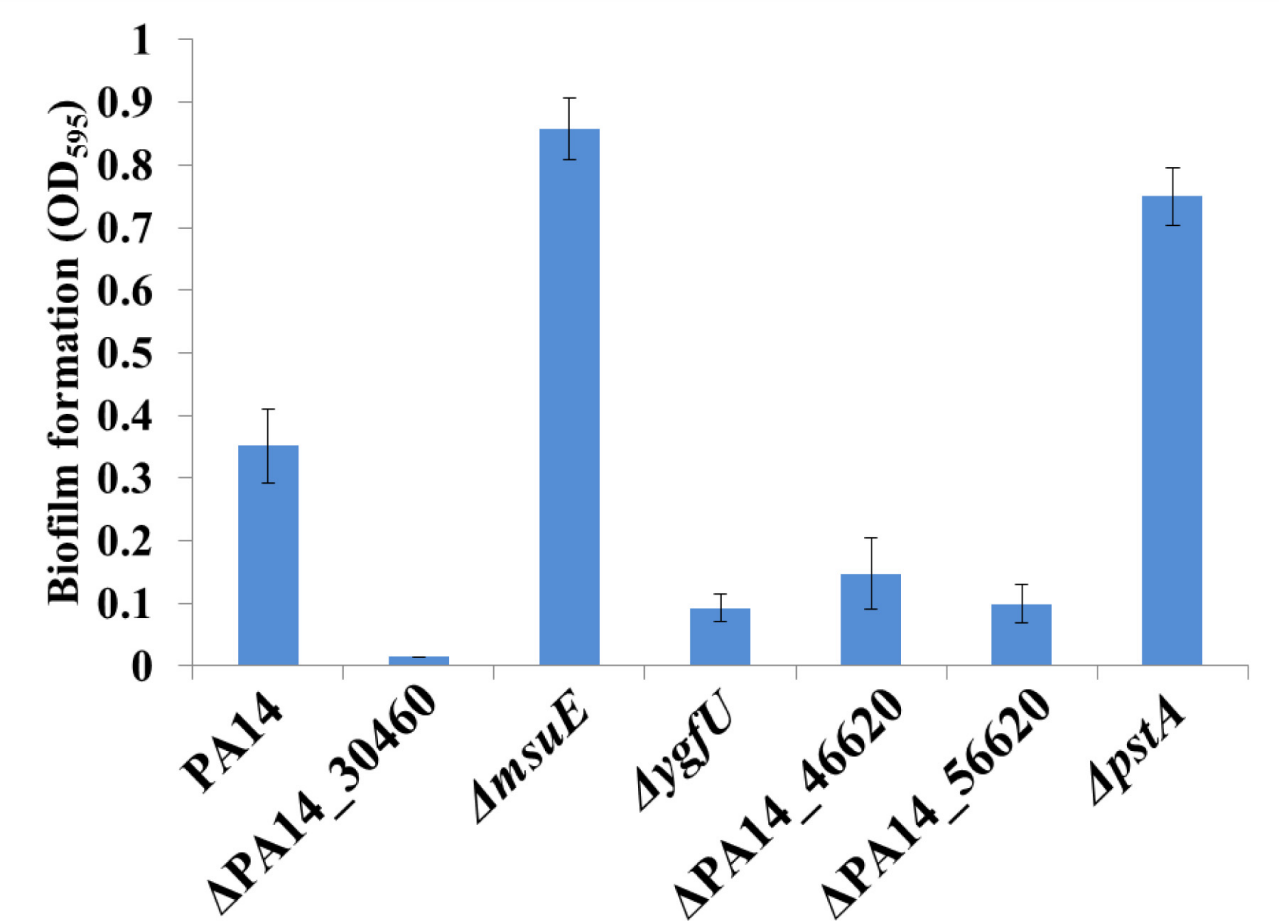
Biofilm formation of PA14 and transposon mutants thereof after anaerobic growth in SCFM medium after 96h. Biofilm formation was quantified by measuring the absorbance at 595 nm after crystal violet staining. The results are averaged from three independent experiments.

To identify functions required for sustained anoxic biofilm formation we next focused on transcripts that were up-regulated under condition B-96 when compared with condition P, and which showed no differential abundance between condition A-30 and condition P. Among them, we selected either single genes or genes that represent the first gene of an operon that were (i) at least 10-fold up-regulated under condition B-96 *versus* P, that were (ii) not differentially abundant under condition A-30 *versus* P, and (iii) for which insertion mutants were available from the PA14 transposon library [57]. The 26 selected genes for which insertion mutants were available are listed in S3 Table. Out of the corresponding 26 transposon mutants tested for anoxic biofilm formation, one showed no (PA14_30460) and three showed reduced (*ygfU*; *pyeR*; PA14_46620) anoxic biofilm formation, whereas insertional inactivation of the *pstA* and *msuE* genes caused increased anoxic biofilm formation (Fig 2).

The inactivation of the putative flavin-dependent oxidoreductase (PA14_30460 / PA2600) gene abolished anoxic biofilm formation. According to the Kegg pathway (http://www.kegg.jp/kegg-bin/show_organism?org=pau), this enzyme is predicted to be involved in converting organosulfonates into the corresponding aldehyde and sulfite. In contrast, the inactivation of the *msuE* gene resulted in increased anoxic biofilm formation. The *musE* gene is the first gene of the *msuEDC* operon encoding the NADH-dependent flavin mononucleotide (FMN) reductase, which provides reduced FMN to flavin mononucleotide (FMNH2)-dependent monooxygenases (MsuD), which in turn catalyzes the desulfonation of organosulfonates in the presence of oxygen [56]. The MsuE and PA14_30640 proteins are both involved in metabolizing organosulfonates, which are absent in SCFM. Thus, it is rather obscure why the inactivation of these functions has an opposite effect on anoxic biofilm formation. We can only speculate that these proteins have an additional function(s) that impacts on anoxic biofilm growth.

Inactivation of PA14_46620 (no homologue in PAO1) resulted in reduced anoxic biofilm formation. PA14_46620 encodes a putative FAD dependent pyridine nucleotide-disulfide oxidoreductase [58], which catalyzes disulfide bond formation. Forty proteins containing disulfide bonds were identified in *P*. *aeruginosa* [59]. Some of these including the molybdopterin biosynthetic protein B2 [60] and the ATPase synthase alpha chain [59] play an important role in anaerobic metabolism or in initial stages of biofilm formation (Type 4 fimbrial biogenesis protein PilE and PilA) [19]. It is thus formally possible that inactivation of PA14_46620 impacts on the functionality of these proteins.

The *ygfU* transposon mutant formed less biofilms, which was also observed under aerobic conditions in LB medium [61]. The gene encodes a putative purine permease [62]. As uracil is known to impact on biofilm formation [63], the biofilm phenotype of the *ygfU* transposon mutant could result from a defect in uracil uptake.

The *pyeR* transposon mutant was impaired in anoxic biofilm formation. The *pyeR* gene encodes a transcriptional regulator belonging to the ArsR family. It is located within an operon coding for an uncharacterized transporter (PA14_56620) that belongs to the major facilitator superfamily (MFS). PyeR was shown to be required for tight micro-colony formation [64].

The *pstA* gene is part of the phosphate-specific transport operon (*pstABC*), which is de-repressed under phosphate limitation. The inactivation of the *pstA* gene resulted in increased anoxic biofilm formation. This phenotype seems to contrast that reported for *P*. *aureofaciens*, in which aerobic biofilm formation is inhibited under phosphate starvation conditions [65]. However, Müsken *et al*. [61] reported likewise an impaired aerobic biofilm formation of a *pstC* transposon mutant after growth in LB medium.

### Differential abundance of PA14 antibiotic resistance genes in anoxic biofilms: correlation with the susceptibility towards different antimicrobials

Next, we sought to correlate the differential abundance of transcripts encoding known or putative resistance functions in B-96 cells with the tolerance of anoxic biofilms towards different antimicrobials. The identified transcripts that showed at least a +/- 5-fold change (p-value < 0.05) in anoxic biofilms (B-96) when compared with planktonically growing cells (P) are listed in Table 2. Transcripts with a fold change lower than +/-5 are only listed if they are part of an operon, wherein the majority of transcripts showed a fold change higher than +/- 5. The majority of the differentially abundant transcripts encode either regulators or structural components of RND drug efflux pumps (Table 2).

**Table 2:** Altered abundance of transcripts encoding antibiotic tolerance functions. Fold change under the condition B-96 when compared with P. Only ≥ ± 5 fold change was considered.

PA14-ID	Gene	Fold change B-96 vs P	p-value	Substrate(s)	References
PA14_09500	*opmD*	-9.78	1.02E-53	Fluoroquinolones	[66–68]
PA14_09520	*mexI*	-6.03	4.33E-22	″	
PA14_09530	*mexH*	-6.60	9.19E-39	″	
PA14_09540	*mexG*	-4.24	8.18E-24	″	
PA14_60810	*nfxB*	3.60	7.34E-03	Fluoroquinolones, β -lactams, Tetracycline, Chloramphenicol, Macrolides, Trimethoprim, Novobiocin	[69,70]
PA14_60820	*oprJ*	3.96	1.27E-02	″	
PA14_60830	*mexD*	6.28	5.70E-02	″	
PA14_60850	*mexC*	7.81	7.22E-02	″	
PA14_60860	*nfxB*	7.08	1.55E-04	″	
PA14_51880	*oprD*	-5.07	2.25E-30	Carbapenem	[71–73]
	*oprI*	-18.26	9.42E-54	hRNase7 and cationic α-helical antimicrobial peptides (AMP)	[74]

The genes constituting the MexGHI-opmD efflux pump were down-regulated in B-96 cells when compared with P. This pump was shown to be involved in the export of the antibiotic norfloxacin [67]. When tested for norfloxacin susceptibility under the conditions P and B-96, we observed an increased tolerance to the antibiotic in anoxic biofilms when compared with planktonically growing cells (Table 1), which apparently conflicts with the reduced abundance of the *mexGHI-opmD* transcripts. However, gyrase inhibitors such as norfloxacin primarily affect fast growing cells [75]. We therefore hypothesize that the slow growth of B-96 cells effects intrinsic resistance and accounts for their decreased susceptibility towards norfloxacin rather than the variations in the level of the *mexGHI-opmD* transcripts.

The genes encoding the MexCD-OprJ efflux pump were up-regulated in B-96 cells versus P cells. When over-produced, this pump was shown to provide resistance to several classes of antibiotics including fluoroquinolones, ß-lactams, tetracycline, chloramphenicol, macrolides, trimethoprim and novobiocin [43]. Accordingly, we observed an increased tolerance to azithromycin, ciprofloxacin, gentamycin and tetracycline (Table 1). The observed susceptibility pattern concurred with the observed up-regulation of the *mexCD-oprJ* transcripts (Table 2). The RNA_Seq_ results were representatively verified for the *mexD* gene by RT-qPCR. This analysis showed that the *mexD* transcript levels were approximately 5-fold increased in B-96 cells (S2 Fig), which was in accordance with the RNA_Seq_ data. Nevertheless, as noted above we cannot exclude that other factors may also account for the increased tolerance to these antibiotics.

The *oprD* gene transcript, which was ~ 5-fold reduced (Table 2) in B-96 anoxic biofilm, encodes an outer membrane porin serving as an entry port for carbapenems [71]. In line with the transcriptome data, B-96 cells displayed a > 16-fold increased MIC towards meropenem when compared with planktonically growing cells. To verify that *oprD* expression is indeed down-regulated in anoxic biofilms, we made use of a translational *oprD*::*lacZ* reporter gene as a means to monitor the production of OprD. The ß-galactosidase activity conferred by the OprD-LacZ protein in strain PA14(pTLoprD) was determined during planktonic growth (OD_600_ = 2.0) and in anoxic biofilms (B-96). As shown in S3 Fig, OprD-LacZ production was reduced in B-96 anoxic biofilms, which can explain the increased tolerance to meropenem (Table 1).

**Fig 3:**
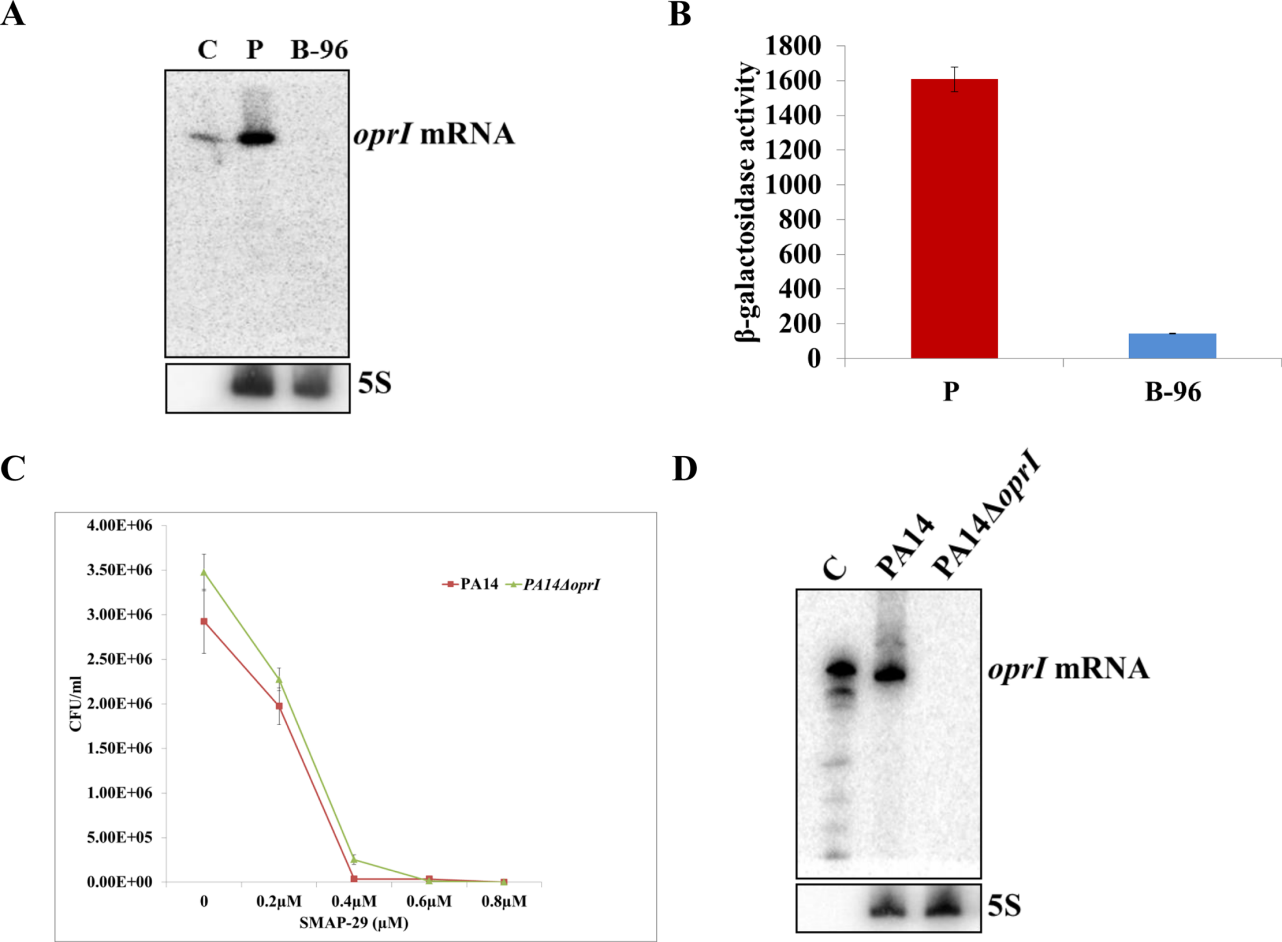
OprI is not required for susceptibility of PA14 towards the antimicrobial peptide SMAP-29. **A)** Determination of the levels of *oprI* mRNA in P cells (P) and B-96 cells (B-96) by Northern-blot analysis. *In-vitro* transcribed *oprI* mRNA (0.5 ng) was used as a control (C). 5S rRNA served as a loading control. **B)** The strains were grown planktonically to an OD_600_ of 2.0 in SCFM (P) and for 96 hours under anaerobic conditions (B-96). Then, the cultures were harvested and the β-galactosidase activities were determined. The bars depict β-galactosidase values conferred by the translational OprI-LacZ protein in strain PA14(pTLoprI) under the conditions P and B-96. The error bars represent standard deviations from three independent experiments. **C)** Susceptibility of PA14 (red) and PA14Δ*oprI* (green) towards the cationic peptide, SMAP-29, under aerobic conditions. The experiment was performed as outlined in Materials and Methods. **D)** Determination of the *oprI* mRNA levels by Northern-blot analysis in strains PA14 and PA14∆*oprI* at the time of addition of SMAP-29 to the cultures. *In vitro* transcribed *oprI* mRNA (1 ng) was used as a control (C). 5S rRNA served as a loading control.

The highly abundant 8-kDa outer membrane lipoprotein OprI can exist in free and peptidoglycan-bound form [76]. It has been reported that OprI is targeted by cationic antimicrobial peptides / proteins such as SMAP-29, LL37 or human RNase7 in *P*. *aeruginosa* O1 [74]. The encoded OprI protein was shown to be produced in PA14 and is 100% homologous to that of PAO1 [77]. The RNA_Seq_ analysis revealed that the *oprI* transcript is ~ 18-fold down-regulated in B-96 cells (Table 2). For verification, Northern-blot analyses were performed with total RNA isolated from P-cells and from B-96 cells using a radio-labelled probe for *oprI* mRNA. A strong *oprI* specific signal was observed in the RNA sample purified from planktonically growing cells, whereas no signal was detected in that isolated from B-96 cells, indicating that *oprI* is not expressed in anoxic biofilms (Fig 3A). In addition, we employed a translational *oprI*::*lacZ* reporter gene, transcription of which is directed by the authentic *oprI* promoter, to assess the synthesis of OprI under conditions P and B-96 in strain PA14 (pTLoprI), respectively. When compared with condition P, OprI-LacZ synthesis was strongly reduced in B-96 anoxic biofilms (Fig 3B), which suggested that B-96 cells might exhibit resistance or at least highly increased tolerance towards SMAP-29, whereas P-cells would be assumed to be sensitive. To test this, P-cells (OD_600_ = 2.0) and B-96 cells grown in SCFM medium were further incubated for 14 h in the presence of different concentrations (0.1 μM– 1.6 μM) of the antimicrobial peptide SMAP-29. Despite the presence of the *oprI* transcript and translation of the *oprI*::*lacZ* fusion gene in P-cells (Fig 3A and 3B), the addition of SMAP-29 did not inhibit growth of either culture in SCFM medium (not shown). However, as the bactericidal activity of antimicrobial peptides is affected by divalent cations [74] it was possible that the composition of the SCFM medium impacted on the susceptibility towards SMAP-29.

This prompted us to revisit the role of OprI as a target for SMAP-29 in strain PA14 under the same condition as previously described for strain PAO1 [74]. In addition, an in frame PA14 *oprI* deletion mutant was constructed to unambiguously check whether OprI is required as a target for SMAP-29 in strain PA14. The strains PA14 and PA14Δ*oprI* were grown aerobically in LB medium and approximately 1 x 10^5^ cells were treated with different concentrations of SMAP-29 for 3h as previously reported for strain PAO1 [74]. The CFU was determined after overnight growth and plating of serial dilutions on LB plates. Under these conditions both strains were equally susceptible to SMAP-29 (Fig 3C) although the *oprI* transcript was only detectable in the wild-type strain (Fig 3D). Hence, these results clearly question OprI as a cellular target for SMAP-29 in strain PA14.

## Conclusions

The RNA_Seq_ based comparative RNA profiling of the clinical isolate PA14 cultured in SCFM under the conditions P, A-30 and B-96 not only highlighted again known functions required for anaerobiosis, but revealed also functions involved in the sulfur metabolism that impact on anoxic biofilm formation. In addition, these studies revealed a decreased and increased abundance of the *oprD* gene and the *mexCD-oprJ* operon genes, respectively, in B-96 cells. This observation can explain the increased tolerance towards meropenem and to antibiotics which are expelled by the MexCD-OprJ efflux pump. Arguably, the SCFM medium used here only approximates to the conditions of the cystic fibrosis lung. It remains thus open whether the same correlations apply to the natural setting in the patient.

The vast difference in abundance of the *oprI* transcript in P- and B-96 cells prompted us to revisit the requirement of OprI as a target for cationic antimicrobial peptides. The sensitivity of the PA14Δ*oprI* mutant towards SMAP-29 was found to be indistinguishable from the parental wild-type strain during logarithmic oxygenic growth. This observation obviously questions OprI as a target for this antimicrobial peptide in strain PA14.

## Supporting Information

S1 Fig Levels of the *msuE* transcript determined by RT-qPCR. Total RNA was prepared from PA14 grown under the conditions P (red bars), B-96 (blue bars) and M-96 (green bars). The levels of the *msuE* transcript were determined by RT-qPCR using the primer pair X124/Y124 (S1 Table) and after normalization to the *rpoD* mRNA levels. The values represent the means and SDs (standard deviations) of changes in comparison with the *msuE* transcript level in anoxic biofilms (B-96), which was set to one. All results are the average of at least three independent experiments and the error bars represent SDs.
(TIF)

S2 Fig Levels of the *mexD* transcript determined by RT-qPCR. Total RNA was prepared from PA14 grown under the conditions P (red bars) and B-96 (blue bars). The levels of the *mexD* transcript were determined by RT-qPCR using the primer pair J124/K124 (S1 Table) and after normalization to the *rpoD* mRNA levels. The values represent the means and SDs (standard deviations) of changes in comparison with the *mexD* transcript level in anoxic biofilms (B-96), which was set to one. All results are the average of at least three independent experiments and the error bars represent SDs.
(TIF)

S3 Fig OprD-LacZ production during planktonic growth and in anoxic biofilms. The strains were grown planktonically to an OD_600_ of 2.0 in SCFM (P) and for 96 hours under anaerobic conditions (B-96). Then, the cultures were harvested and the β-galactosidase activities were determined. The bars depict β-galactosidase values conferred by the translational OprD-LacZ protein in strain PA14 (pTLoprD) under the conditions P and B-96. The error bars represent standard deviations from three independent experiments.
(TIF)

S1 Table Oligonucleotides used in this study. (DOCX)

S2 Table A) Functional classification of transcripts that are differentially abundant under the conditions A-30 and B-96 when compared with condition P. B) Up-regulated metabolic pathways under anoxic conditions. (DOCX)

S3 Table Selected up-regulated functions in B-96 cells scrutinized for anoxic biofilm formation. (DOCX)
