# Supplementary figures and images for: RNASeq Based Transcriptional Profiling of Pseudomonas aeruginosa PA14 after Short- and Long-Term Anoxic Cultivation in Synthetic Cystic Fibrosis Sputum Medium
Source: PLoS One. 2016 Jan 28;11(1):e0147811. doi: 10.1371/journal.pone.0147811 (PMC4731081; doi:10.1371/journal.pone.0147811)

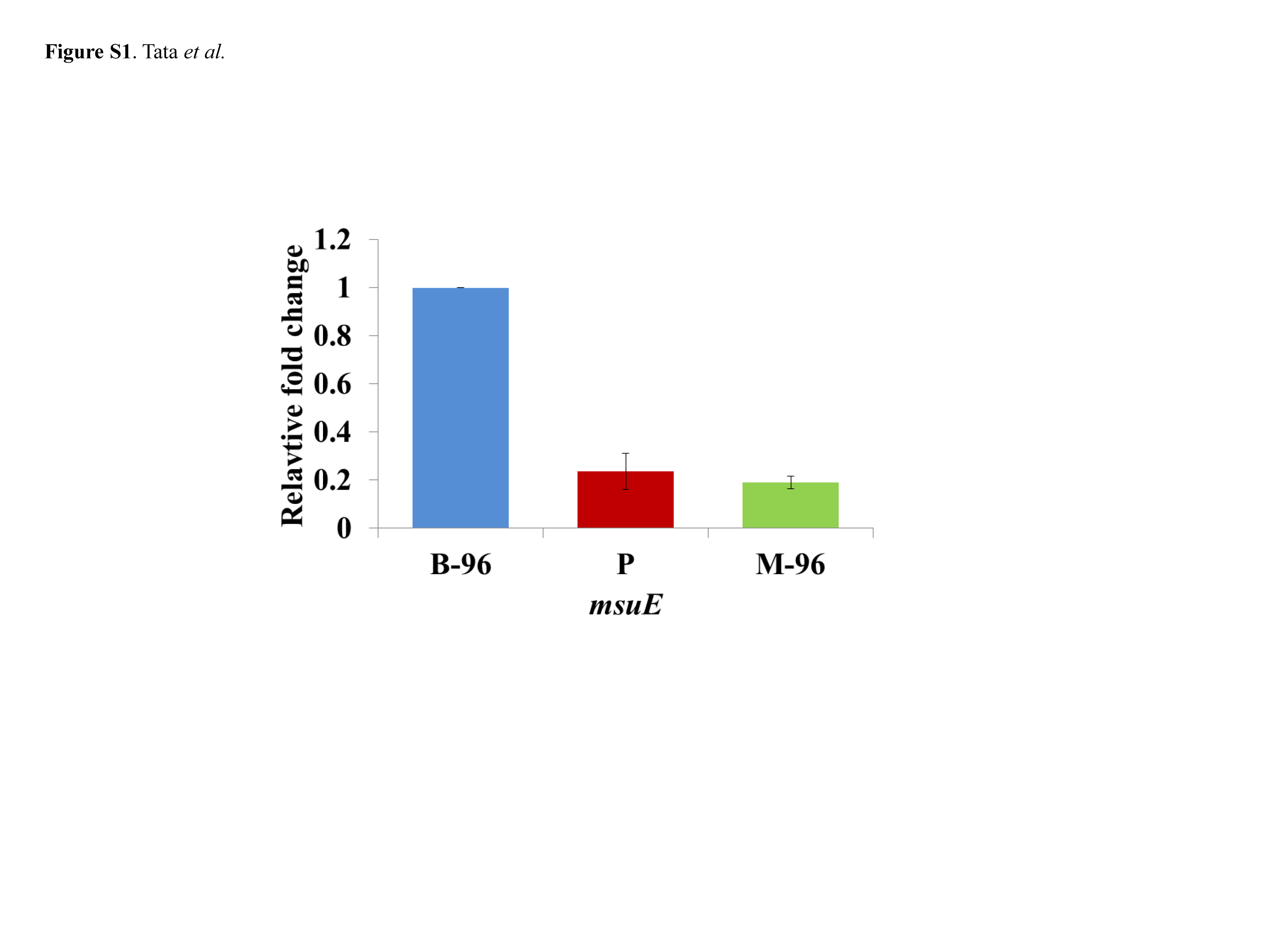

Supplement: S1 Fig — Total RNA was prepared from PA14 grown under the conditions P (red bars), B-96 (blue bars) and M-96 (green bars). The levels of the msuE transcript were determined by RT-qPCR using the primer pair X124/Y124 (S1 Table) and after normalization to the rpoD mRNA levels. The values represent the means and SDs (standard deviations) of changes in comparison with the msuE transcript level in anoxic biofilms (B-96), which was set to one. All results are the average of at least three independent experiments and the error bars represent SDs. (TIF) [file pone.0147811.s001.tif]

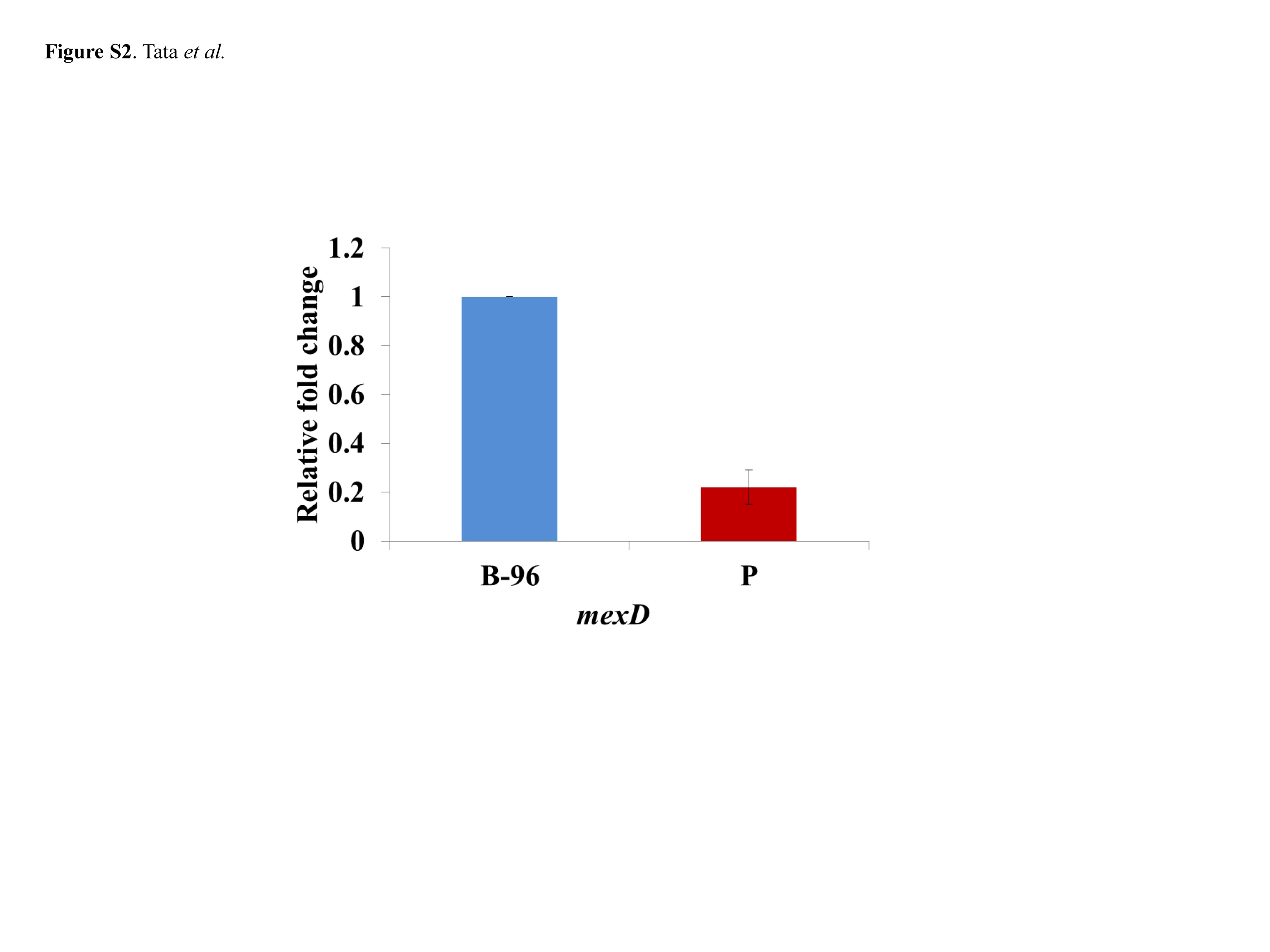

Supplement: S2 Fig — Total RNA was prepared from PA14 grown under the conditions P (red bars) and B-96 (blue bars). The levels of the mexD transcript were determined by RT-qPCR using the primer pair J124/K124 (S1 Table) and after normalization to the rpoD mRNA levels. The values represent the means and SDs (standard deviations) of changes in comparison with the mexD transcript level in anoxic biofilms (B-96), which was set to one. All results are the average of at least three independent experiments and the error bars represent SDs. (TIF) [file pone.0147811.s002.tif]

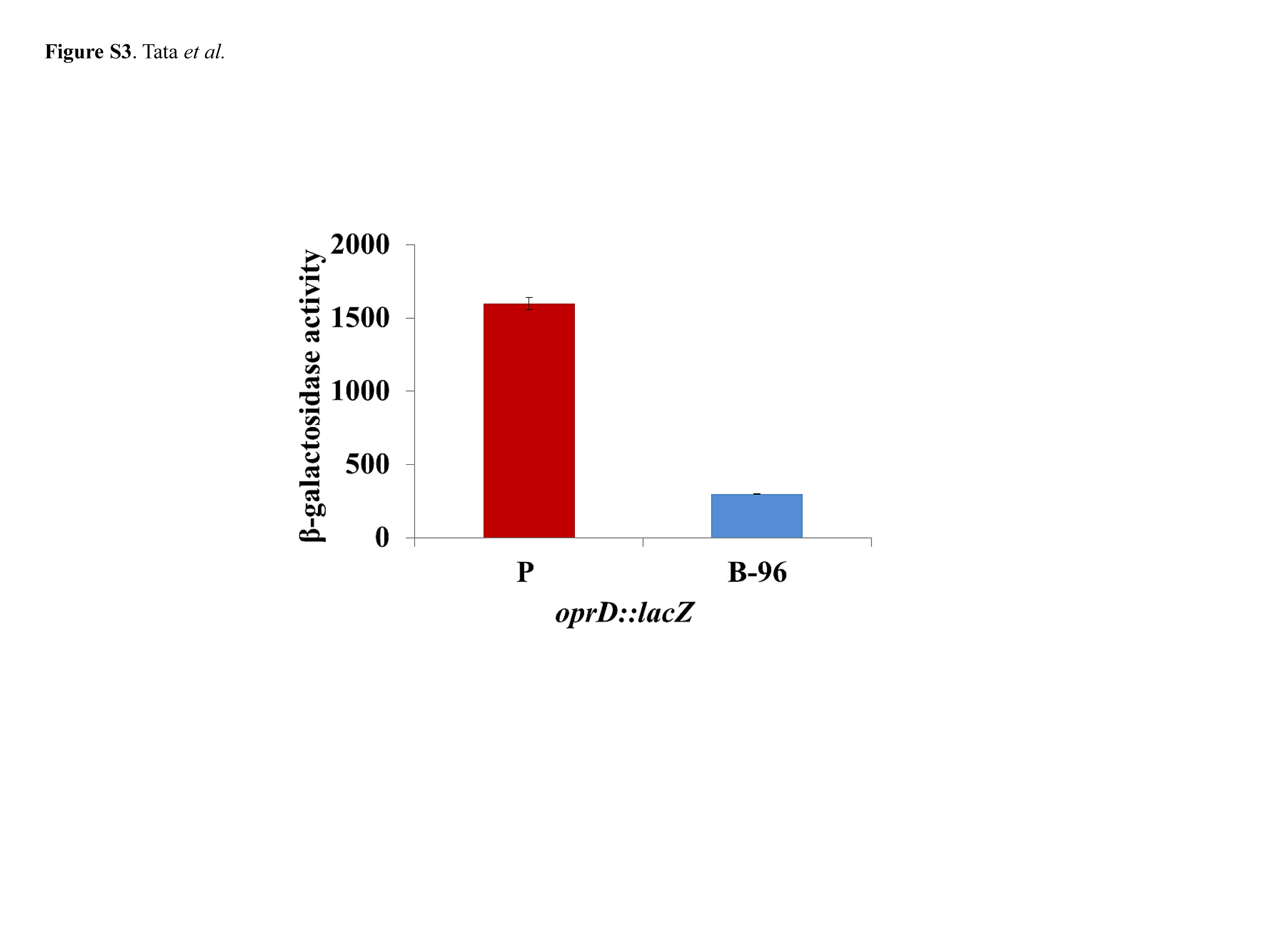

Supplement: S3 Fig — The strains were grown planktonically to an OD600 of 2.0 in SCFM (P) and for 96 hours under anaerobic conditions (B-96). Then, the cultures were harvested and the β-galactosidase activities were determined. The bars depict β-galactosidase values conferred by the translational OprD-LacZ protein in strain PA14 (pTLoprD) under the conditions P and B-96. The error bars represent standard deviations from three independent experiments. (TIF) [file pone.0147811.s003.tif]
