# Supplementary material for: RNASeq Based Transcriptional Profiling of Pseudomonas aeruginosa PA14 after Short- and Long-Term Anoxic Cultivation in Synthetic Cystic Fibrosis Sputum Medium
Source: PLoS One. 2016 Jan 28;11(1):e0147811. doi: 10.1371/journal.pone.0147811 (PMC4731081; doi:10.1371/journal.pone.0147811)
Supplement: S1 Table — (DOCX) [file pone.0147811.s004.docx]

**Table S1**. **Oligonucleotides used in this study.** Restriction sites are highlighted in bold and are in italics.

| **Designation** | **Sequence (5' → 3')** | **Utilization** |
| --- | --- | --- |
| I84 | CAGCAATCTCCCCAGCCGGGG | Detection of *oprI* |
| F95 | GCATAC***GGTACC***CGCGAGGGCTGGGGTTCGCCGATC | Deletion of *oprI* |
| G95 | GCTCAT***TCTAGA***GCGGCTCGTCCACCCACAGGCGC | Deletion of *oprI* |
| H95 | TGAAAACCGGTCCCTCGGGGCCGGTTT | Deletion of *oprI* |
| I95 | AAACCGGCCCCGAGGGACCGGTTTTCAACCCATTACTTGTAGTAAGTAAACTG | Deletion of *oprI* |
| K99 | GTAG***GAATTC***AACTCTACCCAAGGTCCGGG | Cloning of *oprI* |
| M99 | ACGT***CTGCAG***CAGAGCCAGAGCAGAGAATTTC | Cloning of *oprI* |
| F112 | ACGT***GAATTC*** CAGGTTTC AAGGCATGAT TCG | Cloning of *oprD* |
| H112 | ACGT***CTGCAG*** GCTCCACTTCATCACTTTCATTG | Cloning of *oprD* |
| I26 | CCCCACACTACCATCGGCGATGCGTCG | Detection of 5S rRNA |
| J124 | AGTACAGCGTGCCCTACGAC | qRT-PCR: *mexD* (Forward) |
| K124 | AACCCCAGCAGGTACATCAC | qRT-PCR: *mexD* (Reverse) |
| X124 | CTCAAGCACCTGTTCGACCT | qRT-PCR: *msuE* (Forward) |
| Y124 | CGATCCGGTAGTTGTCGAAG | qRT-PCR: *msuE* (Reverse) |
| Q117 | AAGGCCCTGAAGAAGCACGG | qRT-PCR: *ropD* (Forward) |
| R117 | GATCGGCATGAACAGCTCGG | qRT-PCR: *rpoD* (Reverse) |
