# Supplementary material for: RNASeq Based Transcriptional Profiling of Pseudomonas aeruginosa PA14 after Short- and Long-Term Anoxic Cultivation in Synthetic Cystic Fibrosis Sputum Medium
Source: PLoS One. 2016 Jan 28;11(1):e0147811. doi: 10.1371/journal.pone.0147811 (PMC4731081; doi:10.1371/journal.pone.0147811)
Supplement: S2 Table — (DOCX) [file pone.0147811.s005.docx]

**Table S2. A)** **Functional classification of transcripts that are differentially abundant under the conditions A-30 and B-96 when compared with condition P.** The fold-change refers to the abundance of transcripts under the conditions A-30 and B-96 relative to condition P. Only changes ≥ ± 2.5-fold are shown. Genes are ordered according to the PA14-ID and their functional classification (http://www.pseudomonas.com/). Also identified by ^¶^Eichner *et al.* [27], *Wu *et al.* [30] and by^‡^Platt *et al.* [33]. **B) Up-regulated metabolic pathways under anoxic conditions.** The fold-change refers to the abundance of transcripts under the conditions A-30 and B-96 when compared with condition P. Only changes ≥ + 2.5-fold are shown. Genes are ordered according to pathway classification and PA14-ID adapted from the KEGG data base (http://www.kegg.jp/kegg-bin/show_organism?org=pau).

**Table S2A**

| **PA14-ID** | **Gene** | | **Fold change A-30 vs P** | **p-value** | **Fold change B-96 vs P** | **p-value** | **Function** |
| --- | --- | --- | --- | --- | --- | --- | --- |
| **Adaptation, Protection** | | |  |  |  |  |  |
| PA14_00710 | | *osmC* | -8.20 | 2.55E-38 | -35.98 | 5.64E-105 | osmotically inducible protein OsmC |
| PA14_01240 | |  | -2.50 | 7.76E-09 |  |  | carbonic anhydrase |
| PA14_01710^‡^* | | *ahpC* | -3.28 | 1.14E-06 |  |  | alkyl hydroperoxide reductase |
| PA14_01720 | | *ahpF* |  |  | -2.76 | 5.03E-12 | alkyl hydroperoxide reductase |
| PA14_06130 | |  | -3.82 | 5.58E-17 | -3.30 | 8.71E-17 | hypothetical protein |
| PA14_07770^‡^ | | *ostA* | 4.24 | 2.91E-21 | 2.66 | 1.62E-12 | organic solvent tolerance protein OstA |
| PA14_09150^‡^* | | *katA* |  |  | -2.71 | 1.88E-08 | catalase |
| PA14_14680 | | *suhB* | 9.92 | 1.99E-46 | 4.15 | 2.44E-22 | extragenic suppressor protein SuhB |
| PA14_17450 | | *surE* | 4.37 | 1.64E-20 | 2.85 | 9.47E-13 | stationary phase survival protein SurE |
| PA14_19490 | | *lsfA* |  |  | 82.87 | 5.70E-79 | antioxidant protein |
| PA14_20970* | | *cyp23* |  |  | -3.71 | 1.03E-19 | cytochrome P450 |
| PA14_21760 | | *capB* | 5.69 | 2.06E-29 | 24.88 | 1.86E-93 | cold acclimation protein B |
| PA14_25040* | |  | -7.34 | 2.09E-21 | -3.48 | 1.33E-13 | hypothetical protein |
| PA14_27480 | | *htpX* |  |  | 5.97 | 3.17E-35 | heat shock protein HtpX |
| PA14_27510* | | *msrB* | -6.13 | 7.98E-29 |  |  | methionine sulfoxide reductase B |
| PA14_27520 | | *gpo* |  |  | 2.62 | 3.67E-12 | glutathione peroxidase |
| PA14_32630 | |  | -5.86 | 1.72E-07 | -3.06 | 2.25E-05 | cytochrome P450 |
| PA14_33270 | | *pvdG* | -26.58 | 9.26E-37 |  |  | protein PvdG |
| PA14_33280 | | *pvdL* | -239.35 | 1.64E-179 | -7.61 | 9.27E-45 | peptide synthase |
| PA14_33630 | | *pvdJ* | -86.41 | 2.24E-133 | -8.37 | 2.53E-48 | protein PvdJ |
| PA14_33650 | | *pvdD* | -88.18 | 4.69E-65 | -7.22 | 3.05E-25 | pyoverdine synthetase D |
| PA14_33690 | | *pvdE* | -10.71 | 1.84E-40 |  |  | pyoverdine biosynthesis protein PvdE |
| PA14_33700 | | *pvdF* | -7.13 | 4.15E-33 |  |  | pyoverdine synthetase F |
| PA14_33740 | | *pvdP* | -9.13 | 3.22E-40 |  |  | protein PvdP |
| PA14_34460 | |  | -12.87 | 1.55E-50 |  |  | hypothetical protein |
| PA14_35080 | | *arsH* |  |  | 3.29 | 1.27E-09 | arsenical resistance protein |
| PA14_35100 | | *arsC* |  |  | 6.94 | 4.80E-14 | arsenate reductase |
| PA14_37790 | | *pcoA* | -4.86 | 3.53E-14 | 2.91 | 5.83E-11 | copper resistance protein A |
| PA14_41440* | | *uspA* | -5.37 | 2.40E-26 | -5.61 | 7.07E-34 | hypothetical protein |
| PA14_41880 | |  | -5.93 | 1.09E-08 |  |  | universal stress protein |
| PA14_42020 | | *arsC* |  |  | 2.92 | 1.01E-12 | hypothetical protein |
| PA14_45940 | | *lasI* |  |  | -6.61 | 7.79E-26 | autoinducer synthesis protein LasI |
| PA14_47550* | |  | -7.25 | 1.26E-12 | -3.55 | 7.79E-09 | glutathione peroxidase |
| PA14_49760 | | *rhlC* | -8.96 | 1.12E-36 |  |  | rhamnosyltransferase 2 |
| PA14_51830^‡^ | |  |  |  | -3.01 | 2.63E-15 | DNA-binding stress protein |
| PA14_56220 | |  | -3.26 | 9.87E-04 | -12.43 | 4.48E-09 | hypothetical protein |
| PA14_56590* | |  | -9.51 | 1.85E-44 | -68.01 | 4.27E-141 | hypothetical protein |
| PA14_56780 | | *sodB* | 4.43 | 5.25E-22 |  |  | superoxide dismutase |
| PA14_58000 | | *sodM* | -48.31 | 8.74E-108 | -14.42 | 3.68E-70 | superoxide dismutase |
| PA14_60950 | | *cstA* | -2.83 | 1.51E-11 | -4.33 | 1.10E-25 | hypothetical protein |
| PA14_63360 | |  | 2.57 | 6.61E-08 |  |  | hypothetical protein |
| PA14_64460 | | *psiF* | -14.81 | 9.48E-60 | -22.95 | 1.49E-88 | hypothetical protein |
| PA14_66090 | |  | 5.77 | 1.10E-11 | 4.47 | 3.22E-21 | hypothetical protein |
| PA14_66460 | |  | -9.60 | 2.33E-44 | -12.63 | 1.31E-64 | hypothetical protein |
| PA14_67560 | | *typA* | 5.22 | 7.05E-15 | 2.95 | 2.65E-14 | GTP-binding protein TypA |
| PA14_72760 | |  | -3.08 | 2.06E-04 |  |  | beta-lactamase |
| **Amino acid biosynthesis and metabolism** | | | | | | | |
| PA14_00290* | *aroE* | | 19.94 | 4.46E-68 |  |  | shikimate 5-dehydrogenase |
| PA14_00440 | *trpA* | | 3.37 | 5.97E-11 | 2.56 | 1.42E-10 | tryptophan synthase subunit alpha |
| PA14_00450 | *trpB* | | 2.83 | 1.14E-06 | 2.62 | 4.28E-11 | tryptophan synthase subunit beta |
| PA14_02730 |  | | -3.36 | 7.75E-08 | 2.88 | 3.37E-13 | dihydrodipicolinate synthetase |
| PA14_03020 | *aroE* | |  |  | 2.54 | 5.97E-03 | shikimate 5-dehydrogenase |
| PA14_03030 | *aroQ2* | |  |  | 5.73 | 1.17E-10 | 3-dehydroquinate dehydratase |
| PA14_03830 | *aguB* | |  |  | 3.28 | 6.78E-12 | N-carbamoylputrescine amidohydrolase |
| PA14_03860 |  | |  |  | 3.84 | 8.38E-22 | glutamine synthetase |
| PA14_03870 | *spuA* | | 3.25 | 6.45E-15 | 11.85 | 1.12E-61 | glutamine amidotransferase |
| PA14_03880 | *spuB* | |  |  | 4.35 | 2.35E-25 | glutamine synthetase |
| PA14_04110^‡^ | *serA* | | 5.75 | 1.40E-21 | 6.16 | 1.43E-34 | D-3-phosphoglycerate dehydrogenase |
| PA14_04320 | *ilvA1* | | 8.83 | 1.45E-40 |  |  | threonine dehydratase |
| PA14_05080 | *metX* | | 14.43 | 9.68E-48 | 4.30 | 1.57E-14 | homoserine O-acetyltransferase |
| PA14_05230 |  | | -3.06 | 1.34E-12 | -2.84 | 1.11E-13 | cystathionine gamma-lyase |
| PA14_06120 |  | | -2.83 | 4.65E-10 | -3.85 | 2.82E-17 | hypothetical protein |
| PA14_07940* | *trpE* | | 2.84 | 1.96E-11 |  |  | anthranilate synthase component I |
| PA14_08340 | *trpG* | | 3.27 | 1.76E-13 | 2.66 | 5.28E-11 | anthranilate synthase component II |
| PA14_08350 | *trpD* | |  |  | -2.63 | 3.32E-12 | anthranilate phosphoribosyltransferase |
| PA14_09730 | *dapA* | |  |  | -4.32 | 3.00E-10 | dihydrodipicolinate synthase |
| PA14_12010* | *proA* | | 3.20 | 6.28E-14 |  |  | gamma-glutamyl phosphate reductase |
| PA14_14020* |  | |  |  | 2.88 | 1.52E-12 | hypothetical protein |
| PA14_14700 | *cysE* | | 3.20 | 4.97E-14 | 3.28 | 7.25E-17 | serine O-acetyltransferase |
| PA14_14730^‡^ | *iscS* | | 2.85 | 3.49E-12 |  |  | cysteine desulfurase |
| PA14_14960 | *yafV* | | -3.18 | 2.13E-13 |  |  | hypothetical protein |
| PA14_15030* | *leuA* | |  |  | 2.94 | 1.52E-14 | 2-isopropylmalate synthase |
| PA14_15910 |  | | 3.49 | 1.06E-14 |  |  | hypothetical protein |
| PA14_16070* | *hom* | | 3.69 | 6.37E-17 |  |  | homoserine dehydrogenase |
| PA14_16090^‡^* | *thrC* | | 2.82 | 1.57E-11 |  |  | threonine synthase |
| PA14_16250 | *lasB* | | -81.98 | 3.02E-132 | -12.48 | 1.09E-64 | elastase LasB |
| PA14_16930 | *csdA* | |  |  | -3.21 | 2.74E-16 | pyridoxal-phosphate dependent protein |
| PA14_16950 | *dapD* | | 3.14 | 2.72E-13 |  |  | tetrahydrodipicolinate succinylase |
| PA14_16960 |  | | 3.71 | 3.84E-15 |  |  | amino acid transporter LysE |
| PA14_18120* | *mmsA* | | -16.82 | 5.13E-10 | -3.13 | 2.02E-04 | methylmalonate-semialdehyde dehydrogenase |
| PA14_18610* | *argF* | | 8.76 | 2.34E-40 | 7.21 | 4.27E-38 | ornithine carbamoyltransferase |
| PA14_18740 | *argG* | | 4.84 | 4.47E-23 | 3.19 | 3.46E-15 | argininosuccinate synthase |
| PA14_19140 | *pheC* | |  |  | 2.64 | 9.60E-12 | cyclohexadienyl dehydratase |
| PA14_19370 | *asnB* | |  |  | -5.45 | 7.16E-33 | asparagine synthetase |
| PA14_19770 |  | |  |  | 2.95 | 1.75E-11 | hypothetical protein |
| PA14_19870^‡^ | *ldh* | | -34.85 | 3.80E-62 | -10.38 | 1.05E-40 | leucine dehydrogenase |
| PA14_23270 | *serC* | | 2.75 | 1.01E-10 |  |  | phosphoserine aminotransferase |
| PA14_23290 | *hisC2* | | 3.60 | 1.60E-16 |  |  | histidinol-phosphate aminotransferase |
| PA14_23500 | *tyrB* | | 4.41 | 3.44E-21 | 4.09 | 3.51E-22 | aromatic amino acid aminotransferase |
| PA14_23750 | *leuC* | |  |  | 3.80 | 7.87E-21 | isopropylmalate isomerase large subunit |
| PA14_23800* | *asd* | | 2.85 | 2.72E-11 |  |  | aspartate-semialdehyde dehydrogenase |
| PA14_23810 |  | | 3.27 | 1.28E-13 |  |  | aspartate-semialdehyde dehydrogenase |
| PA14_23920* | *purF* | | 3.05 | 3.54E-13 |  |  | amidophosphoribosyltransferase |
| PA14_24290 | *gbt* | | 4.83 | 1.27E-22 |  |  | glycine betaine transmethylase |
| PA14_24445* | *gdhB* | | -2.56 | 1.67E-09 | -5.83 | 4.32E-35 | NAD-dependent glutamate dehydrogenase |
| PA14_25540 | *ptpA* | | 3.12 | 5.66E-13 |  |  | phosphotyrosine protein phosphatase |
| PA14_25790 |  | | -3.26 | 8.38E-14 |  |  | hypothetical protein |
| PA14_25980 | *aroF* | | 2.62 | 1.81E-08 | 6.75 | 1.28E-33 | phospho-2-dehydro-3-deoxyheptonate aldolase |
| PA14_31820 | *hisC* | |  |  | -2.70 | 9.86E-04 | aminotransferase |
| PA14_32985 | *gcvH2* | | -2.92 | 5.91E-04 | -3.56 | 4.02E-05 | glycine cleavage system protein H |
| PA14_33000^‡^* | *gcvP2* | | -3.02 | 1.12E-12 | -3.39 | 1.64E-18 | glycine dehydrogenase |
| PA14_33010^‡^* | *glyA2* | | -4.43 | 5.62E-20 | -7.54 | 2.14E-33 | serine hydroxymethyltransferase |
| PA14_33030 | *sdaA* | | -6.79 | 3.14E-33 | -9.75 | 1.07E-53 | L-serine dehydratase |
| PA14_33040^‡^ | *gcvT2* | | -10.82 | 2.96E-48 | -12.07 | 4.46E-62 | glycine cleavage system protein T2 |
| PA14_35500 | *bkdB* | | -13.18 | 1.60E-55 | -57.22 | 3.45E-131 | branched-chain alpha-keto acid dehydrogenase subunit E2 |
| PA14_35520^‡^* | *bkdA2* | | -14.75 | 1.75E-59 | -38.56 | 1.20E-112 | 2-oxoisovalerate dehydrogenase subunit beta |
| PA14_35530^‡^* | *bkdA1* | | -10.53 | 2.59E-17 | -9.00 | 2.18E-18 | 2-oxoisovalerate dehydrogenase subunit alpha |
| PA14_36370 |  | | -4.66 | 3.45E-13 |  |  | carboxylate-amine ligase |
| PA14_36660 |  | | -5.44 | 8.37E-22 | -6.65 | 3.14E-28 | alcohol dehydrogenase |
| PA14_37560 | *asnB* | |  |  | 13.15 | 2.11E-15 | asparagine synthetase, glutamine-hydrolysing |
| PA14_37610* |  | |  |  | -4.45 | 3.20E-18 | kynureninase |
| PA14_37830 | *iscS* | |  |  | 16.19 | 1.37E-68 | pyridoxal-phosphate dependent protein |
| PA14_38140 |  | | 4.60 | 1.18E-21 | 22.53 | 1.48E-85 | glutamine synthetase |
| PA14_38770 |  | | -5.54 | 3.43E-18 |  |  | peptidase |
| PA14_39100 |  | |  |  | 2.63 | 3.20E-08 | hypothetical protein |
| PA14_39590^‡^ | *metE* | |  |  | 126.97 | 1.17E-166 | 5- methyltetrahydropteroyltriglutamate/homocysteine S-methyltransferase |
| PA14_39720 |  | | 5.90 | 5.41E-16 |  |  | amino acid oxidase |
| PA14_40670* | *metH* | |  |  | 4.88 | 1.67E-28 | B12-dependent methionine synthase |
| PA14_41380* | *glnS* | | 2.50 | 4.10E-09 |  |  | glutaminyl-tRNA synthetase |
| PA14_41830 | *thrH* | | 3.97 | 3.07E-14 |  |  | phosphoserine phosphatase |
| PA14_41920* | *aroF1* | | 5.42 | 3.00E-26 | 2.53 | 3.71E-10 | phospho-2-dehydro-3-deoxyheptonate aldolase |
| PA14_42140 |  | | -3.98 | 9.65E-14 | -5.84 | 1.53E-21 | transglutaminase |
| PA14_42760* | *aroC* | | 2.96 | 6.85E-09 |  |  | chorismate synthase |
| PA14_43920 | *braB* | | 3.75 | 3.79E-17 | 2.82 | 6.71E-13 | branched chain amino acid transporter |
| PA14_44210 |  | | -3.52 | 3.55E-12 |  |  | glycine/D-amino acid oxidase |
| PA14_44240* |  | | 5.80 | 9.83E-11 |  |  | glutamine synthetase |
| PA14_45210 |  | | -3.40 | 4.28E-11 | -4.11 | 2.33E-14 | hypothetical protein |
| PA14_46860 |  | |  |  | -2.54 | 1.14E-04 | lysine decarboxylase |
| PA14_46970^‡^ | *ansB* | | -6.25 | 3.85E-30 | -52.76 | 9.17E-123 | glutaminase-asparaginase |
| PA14_47100 | *ilvA2* | | 5.94 | 5.93E-25 |  |  | threonine dehydratase |
| PA14_48570 |  | | -9.01 | 5.96E-39 | -20.48 | 2.89E-71 | 2-isopropylmalate synthase |
| PA14_48610 |  | | -6.47 | 2.81E-26 | -17.47 | 8.48E-53 | sparagine synthase |
| PA14_49380 | *dapE* | | 2.93 | 6.02E-06 |  |  | succinyl-diaminopimelate desuccinylase |
| PA14_51270* | *dapA* | | 3.24 | 5.42E-14 |  |  | dihydrodipicolinate synthase |
| PA14_52210 | *cysM* | | 4.62 | 1.46E-22 | 2.92 | 7.67E-14 | cysteine synthase B |
| PA14_52660* | *aruB* | | -2.57 | 1.32E-09 | -6.66 | 4.12E-39 | succinylarginine dihydrolase |
| PA14_52670* | *astD* | |  |  | -4.06 | 2.10E-22 | succinylglutamic semialdehyde dehydrogenase |
| PA14_52690* | *aruG* | |  |  | -2.87 | 6.99E-14 | arginine/ornithine succinyltransferase AII subunit |
| PA14_52990* | *phhA* | | -2.78 | 5.26E-06 |  |  | phenylalanine 4-monooxygenase |
| PA14_53000* | *phhB* | |  |  | -5.31 | 1.72E-31 | pterin-4-alpha-carbinolamine dehydratase |
| PA14_53010* | *phhC* | |  |  | -5.11 | 6.04E-31 | aromatic amino acid aminotransferase |
| PA14_57210 | *argJ* | | 3.17 | 8.21E-14 |  |  | bifunctional ornithine acetyltransferase/N-acetylglutamate synthase |
| PA14_57670 | *trpS* | | 4.51 | 1.77E-21 |  |  | tryptophanyl-tRNA synthetase |
| PA14_57710 | *cysN* | | 3.45 | 8.74E-16 | 13.50 | 6.37E-66 | bifunctional sulfate adenylyltransferase subunit 1/adenylylsulfate kinase |
| PA14_57720 | *cysD* | | 8.87 | 2.67E-39 | 53.98 | 3.23E-122 | sulfate adenylyltransferase subunit 2 |
| PA14_57770* | *hisC1* | | 3.26 | 5.21E-14 |  |  | histidinol-phosphate aminotransferase |
| PA14_57780* | *hisD* | | 7.60 | 6.66E-37 | 3.31 | 3.40E-16 | histidinol dehydrogenase |
| PA14_57800 | *hisG* | | 7.53 | 8.76E-37 | 3.17 | 2.80E-15 | ATP phosphoribosyltransferase |
| PA14_60890^‡^ | *glyA* | | 4.62 | 3.91E-22 | 6.45 | 5.15E-36 | serine hydroxymethyltransferase |
| PA14_62160 | *ilvI* | | 6.36 | 9.65E-33 | 6.30 | 4.44E-37 | acetolactate synthase 3 catalytic subunit |
| PA14_62940 | *dapB* | | 5.63 | 2.53E-28 |  |  | dihydrodipicolinate reductase |
| PA14_63110 |  | | 4.57 | 2.40E-14 |  |  | S-adenosylmethionine decarboxylase |
| PA14_63120 |  | | 2.54 | 1.57E-06 |  |  | hypothetical protein |
| PA14_64090* | *aroQ1* | | 15.15 | 1.25E-59 | 5.23 | 7.47E-27 | 3-dehydroquinate dehydratase |
| PA14_64850 |  | | -6.93 | 8.50E-28 | -17.24 | 4.54E-52 | ornithine cyclodeaminase |
| PA14_65050 |  | |  |  | -2.58 | 1.50E-05 | hypothetical protein |
| PA14_65560 | *serB* | | 3.35 | 1.13E-14 |  |  | phosphoserine phosphatase |
| PA14_66290^‡^* | *aceE* | | 3.83 | 7.61E-19 | -2.54 | 1.14E-11 | pyruvate dehydrogenase subunit E1 |
| PA14_66440* | *metY* | | 2.60 | 8.16E-08 |  |  | O-acetylhomoserine aminocarboxypropyltransferase |
| PA14_66570* | *gltB* | | 4.42 | 2.62E-22 | 2.63 | 2.97E-12 | glutamate synthase subunit alpha |
| PA14_66600 | *aroB* | | 2.73 | 1.18E-10 |  |  | 3-dehydroquinate synthase |
| PA14_67240* | *hutG* | | -4.23 | 5.05E-19 | -6.95 | 3.83E-37 | N-formylglutamate amidohydrolase |
| PA14_67250* | *hutI* | | -3.16 | 9.05E-13 | -7.79 | 1.92E-40 | imidazolonepropionase |
| PA14_67350* | *hutU* | | -2.55 | 1.23E-09 |  |  | urocanate hydratase |
| PA14_67600* | *glnA* | | 2.56 | 1.07E-09 | -3.23 | 3.35E-17 | glutamine synthetase |
| PA14_67880 | *hisF1* | | 2.50 | 2.99E-05 |  |  | imidazole glycerol phosphate synthase subunit HisF |
| PA14_67890 | *hisA* | | 3.65 | 2.37E-10 |  |  | 1-(5-phosphoribosyl)-5-[(5- phosphoribosylamino)methylideneamino] imidazole-4-carboxamide isomerase |
| PA14_68300 | *arcD* | |  |  | -145.67 | 2.54E-174 | arginine/ornithine antiporter |
| PA14_68330^‡^* | *arcA* | |  |  | -69.31 | 2.20E-139 | arginine deiminase |
| PA14_68340^‡^* | *arcB* | | 3.42 | 3.01E-16 | -50.93 | 5.33E-124 | ornithine carbamoyltransferase |
| PA14_68350^‡^* | *arcC* | | 6.40 | 1.22E-33 | -21.10 | 2.93E-83 | carbamate kinase |
| PA14_68850 | *gcvP1* | | -12.13 | 1.20E-51 | -14.97 | 1.59E-69 | glycine dehydrogenase |
| PA14_69500 | *argH* | | 3.11 | 9.44E-13 | 4.42 | 6.83E-24 | argininosuccinate lyase |
| PA14_69990 | *dadX* | | 5.05 | 1.01E-12 | -3.82 | 4.27E-17 | alanine racemase |
| PA14_70040* | *dadA* | |  |  | -2.70 | 2.04E-12 | D-amino acid dehydrogenase small subunit |
| PA14_70100 |  | | 4.07 | 1.27E-17 | 3.60 | 7.41E-17 | oxidoreductase |
| PA14_71560 | *fdhA* | | -6.08 | 1.63E-25 | -6.27 | 3.46E-29 | glutathione-independent formaldehyde dehydrogenase |
| PA14_71650* | *aspA* | | -4.66 | 7.43E-14 | 7.01 | 9.94E-42 | aspartate ammonia-lyase |
| **Antibiotic resistance and susceptibility** | | | |  |  |  |  |
| PA14_10670* | *aph* | | 3.91 | 6.32E-14 |  |  | aminoglycoside 3'-phosphotransferase type IIB |
| PA14_16820 |  | |  |  | -2.97 | 4.71E-14 | efflux transmembrane protein |
| PA14_20080 |  | | 2.76 | 6.20E-09 |  |  | hypothetical protein |
| PA14_20250 |  | | -11.83 | 1.01E-33 | 3.26 | 4.00E-15 | hypothetical protein |
| PA14_29830 |  | | 8.13 | 8.94E-20 |  |  | methyltransferase |
| PA14_32390 | *mexF* | | 2.76 | 9.69E-09 |  |  | RND multidrug efflux transporter MexF |
| PA14_32400 | *mexE* | | 10.50 | 1.18E-26 | 11.24 | 2.96E-29 | RND multidrug efflux membrane fusion protein MexE |
| PA14_33510 |  | | -26.35 | 1.94E-59 | -2.86 | 4.12E-14 | hypothetical protein |
| PA14_33820 | *pvdQ* | | -14.23 | 2.36E-31 |  |  | penicillin acylase-related protein |
| PA14_38020 |  | | -4.23 | 6.84E-13 |  |  | ntibiotic biosynthesis monooxygenase |
| PA14_38340 |  | | -18.66 | 3.34E-10 | -4.97 | 5.88E-06 | ring-cleaving dioxygenase |
| PA14_39190 | *bacA* | | 6.61 | 5.44E-25 | 3.49 | 1.31E-11 | UDP pyrophosphate phosphatase |
| PA14_46590 |  | |  |  | 3.72 | 6.21E-16 | AcrB/AcrD/AcrF family protein |
| PA14_46680 | *norM* | | 4.02 | 1.30E-17 |  |  | transporter |
| PA14_48240 |  | | -3.50 | 3.34E-06 |  |  | outer membrane component of multidrug efflux pump |
| PA14_55170 | *cat* | |  |  | 3.41 | 8.95E-15 | chloramphenicol acetyltransferase |
| PA14_56890 |  | | 2.99 | 7.74E-10 |  |  | multidrug efflux protein |
| PA14_60820 | *oprJ* | | 7.71 | 2.25E-01 | 3.96 | 1.27E-02 | outer membrane protein OprJ |
| PA14_60830 | *mexD* | | 13.68 | 1.80E-01 | 6.28 | 5.70E-02 | multidrug efflux RND transporter MexD |
| PA14_60850 | *mexC* | | 7.71 | 2.56E-01 | 7.81 | 7.22E-02 | multidrug efflux RND membrane fusion protein |
| PA14_68110 |  | | 3.98 | 7.80E-11 |  |  | transcriptional regulator |
| PA14_72410 |  | | 2.75 | 1.43E-09 |  |  | hypothetical protein |
| PA14_73090 |  | |  |  | -6.65 | 4.11E-34 | hypothetical protein |
| **Biosynthesis of cofactors, prosthetic groups and carriers** | | | | | | | |
| PA14_00280 | *hemF* | | 14.78 | 2.02E-59 |  |  | coproporphyrinogen III oxidase |
| PA14_01430 |  | | -2.56 | 2.29E-08 |  |  | short chain dehydrogenase |
| PA14_01440 |  | | -4.25 | 1.19E-17 | -5.86 | 3.64E-28 | hypothetical protein |
| PA14_04970 | *thiS* | | 18.01 | 7.57E-43 | 8.82 | 8.51E-23 | sulfur carrier protein ThiS |
| PA14_04980 | *thiG* | | 11.83 | 8.17E-47 | 3.80 | 1.52E-15 | thiazole synthase |
| PA14_06510 | *bioF* | |  |  | -4.98 | 2.94E-27 | 8-amino-7-oxononanoate synthase |
| PA14_06530 | *bioH* | |  |  | -10.10 | 1.63E-50 | biotin biosynthesis protein bioH |
| PA14_06540 | *bioC* | | 2.66 | 3.05E-10 | -8.46 | 5.67E-39 | biotin synthesis protein BioC |
| PA14_06570* | *bioD* | |  |  | -7.33 | 6.63E-34 | dithiobiotin synthetase |
| PA14_06890 |  | |  |  | 4.47 | 1.08E-24 | hypothetical protein |
| PA14_07050 |  | | -6.79 | 2.59E-31 | -6.22 | 2.80E-34 | hypothetical protein |
| PA14_07170* | *epd* | | 7.24 | 8.12E-34 |  |  | D-erythrose 4-phosphate dehydrogenase |
| PA14_07740* | *pdxA* | | 3.64 | 2.41E-16 |  |  | 4-hydroxythreonine-4-phosphate dehydrogenase |
| PA14_08620 | *birA* | | 2.60 | 3.33E-09 | 2.62 | 1.78E-10 | biotin--protein ligase |
| PA14_11140 |  | | -20.20 | 2.01E-11 |  |  | nonribosomal peptide synthetase |
| PA14_11410 | *ribC* | | 12.86 | 1.04E-52 | 4.80 | 1.17E-23 | riboflavin synthase subunit alpha |
| PA14_11420 | *ribB* | | 7.39 | 9.66E-36 | 3.44 | 6.54E-17 | bifunctional 3,4-dihydroxy-2-butanone 4-phosphate synthase/GTP cyclohydrolase II-like protein |
| PA14_11430* | *ribH* | | 6.63 | 2.14E-32 |  |  | 6,7-dimethyl-8-ribityllumazine synthase |
| PA14_11460 | *thiL* | | 11.72 | 3.56E-50 | 2.60 | 7.40E-10 | thiamine monophosphate kinase |
| PA14_11550 | *dxs* | | 6.65 | 2.70E-33 | 3.25 | 2.65E-16 | 1-deoxy-D-xylulose-5-phosphate synthase |
| PA14_11560 | *ispA* | | 4.16 | 3.58E-18 |  |  | geranyltranstransferase |
| PA14_12020 | *nadD* | | 3.09 | 3.95E-13 |  |  | nicotinic acid mononucleotide adenylyltransferase |
| PA14_12390* | *hemL* | | 4.36 | 9.94E-21 | 2.64 | 2.74E-11 | glutamate-1-semialdehyde aminotransferase |
| PA14_12400 | *thiE* | | 5.58 | 3.05E-25 | 2.70 | 3.60E-10 | thiamine-phosphate pyrophosphorylase |
| PA14_12410* | *thiD* | | 4.85 | 1.74E-22 |  |  | phosphomethylpyrimidine kinase |
| PA14_13230* | *moaC* | |  |  | 9.44 | 2.19E-51 | molybdenum cofactor biosynthesis protein MoaC |
| PA14_13240 | *moaD* | |  |  | 4.77 | 4.54E-26 | molybdopterin converting factor, small subunit |
| PA14_13250 | *moaE* | |  |  | 4.19 | 1.30E-22 | molybdopterin converting factor, large subunit |
| PA14_13260^‡^* | *moaB1* | | 12.88 | 7.38E-07 | 90.46 | 4.02E-135 | MoaB1 |
| PA14_13280^‡^* | *moeA1* | | 16.10 | 2.58E-08 | 55.55 | 1.80E-29 | molybdenum cofactor biosynthetic protein A1 |
| PA14_13680 |  | | 6.27 | 9.72E-27 |  |  | short chain dehydrogenase |
| PA14_13690* |  | | 3.73 | 8.31E-16 |  |  | methyltransferase |
| PA14_13850 | *moaA* | | 29.77 | 8.16E-09 | 5.04 | 5.84E-14 | molybdenum cofactor biosynthesis protein A |
| PA14_15670 | *cumA* | |  |  | -3.10 | 2.03E-03 | metallo-oxidoreductase |
| PA14_16780 |  | | 2.73 | 2.15E-09 |  |  | hypothetical protein |
| PA14_17130 | *dxr* | |  |  | 3.27 | 1.05E-12 | 1-deoxy-D-xylulose 5-phosphate reductoisomerase |
| PA14_17340 | *ispD* | | 4.30 | 6.16E-20 |  |  | 2-C-methyl-D-erythritol 4-phosphate cytidylyltransferase |
| PA14_19210 |  | | 5.72 | 3.03E-23 | 5.07 | 4.70E-22 | hypothetical protein |
| PA14_19630* | *folE1* | | 3.44 | 6.65E-15 |  |  | GTP cyclohydrolase I |
| PA14_20140 | *fpr* | |  |  | 4.56 | 9.52E-26 | ferredoxin--NADP+ reductase |
| PA14_21640 |  | | -2.73 | 1.51E-10 | -9.22 | 7.43E-32 | short chain dehydrogenase |
| PA14_24890 | *mobA* | | 2.61 | 5.75E-07 |  |  | molybdopterin-guanine dinucleotide biosynthesis protein MobA |
| PA14_24900* | *moaB2* | | 2.91 | 3.13E-09 |  |  | molybdopterin biosynthetic protein B2 |
| PA14_25710 | *pabC* | | 3.89 | 7.22E-19 |  |  | 4-amino-4-deoxychorismate lyase |
| PA14_25920 | *cobM* | | 2.66 | 1.43E-06 | 3.20 | 1.55E-09 | precorrin-3 methylase |
| PA14_25930 |  | | 5.39 | 1.07E-13 | 5.36 | 1.20E-13 | hypothetical protein |
| PA14_25990 |  | | 3.10 | 3.98E-12 |  |  | magnesium chelatase |
| PA14_26470 | *cbiD* | | 2.95 | 6.70E-11 |  |  | cobalt-precorrin-6A synthase |
| PA14_26480 | *cobL* | | 3.95 | 9.02E-17 | 3.29 | 7.41E-12 | precorrin-6y-dependent methyltransferase CobL |
| PA14_26500 | *cobH* | | 3.32 | 5.15E-11 | 2.53 | 2.29E-07 | precorrin-8X methylmutase |
| PA14_27850 | *queF* | | 6.26 | 2.21E-23 | 3.71 | 1.15E-12 | 7-cyano-7-deazaguanine reductase |
| PA14_29600* | *ptpS* | | 6.85 | 5.89E-20 | 6.59 | 7.59E-26 | 6-pyruvoyl-tetrahydropterin synthase |
| PA14_31510* |  | | -7.04 | 2.62E-34 | -34.19 | 1.26E-105 | short-chain dehydrogenase |
| PA14_33520 |  | | -19.44 | 2.21E-68 |  |  | thioesterase |
| PA14_33810 | *pvdA* | | -15.63 | 2.11E-11 |  |  | L-ornithine N5-oxygenase |
| PA14_38330 | *gor* | | -3.27 | 4.25E-10 | -2.90 | 1.86E-10 | glutathione reductase |
| PA14_38780 | *pqqE* | | -3.47 | 4.33E-14 |  |  | pyrroloquinoline quinone biosynthesis protein PqqE |
| PA14_38800 | *pqqC* | | -3.23 | 1.84E-12 |  |  | pyrroloquinoline quinone biosynthesis protein PqqC |
| PA14_38825 | *pqqA* | | -30.63 | 1.84E-31 |  |  | coenzyme PQQ synthesis protein PqqA |
| PA14_39010 | *pqqF* | |  |  | 4.47 | 1.57E-22 | pyrroloquinoline quinone biosynthesis protein F |
| PA14_39210 | *nadR* | | 2.53 | 6.09E-05 |  |  | hypothetical protein |
| PA14_39640 | *cobN* | | -13.70 | 3.57E-54 | -4.24 | 5.24E-24 | cobaltochelatase subunit CobN |
| PA14_41350* | *folD* | |  |  | 3.17 | 1.66E-14 | bifunctional 5,10-methylene-tetrahydrofolate dehydrogenase/ 5,10-methylene-tetrahydrofolate cyclohydrolase |
| PA14_44470* | *hemN* | | -3.46 | 5.92E-06 | -7.96 | 1.57E-12 | coproporphyrinogen III oxidase |
| PA14_46470 | *pdxB* | | 4.19 | 6.55E-18 | 3.13 | 4.10E-13 | erythronate-4-phosphate dehydrogenase |
| PA14_46880 |  | | -4.33 | 1.20E-17 | -4.32 | 5.08E-20 | glutathione synthase |
| PA14_47730 | *cobD* | | 4.39 | 1.20E-15 | 2.63 | 2.10E-07 | cobalamin biosynthesis protein |
| PA14_47760 | *cobB* | | 3.53 | 2.25E-14 |  |  | cobyrinic acid a,c-diamide synthase |
| PA14_50800* | *pdxH* | |  |  | -3.60 | 4.59E-04 | pyridoxamine 5'-phosphate oxidase |
| PA14_54290 | *pdxJ* | |  |  | -2.84 | 2.34E-09 | pyridoxine 5'-phosphate synthase |
| PA14_55580 | *nemO* | | -3.73 | 5.98E-17 |  |  | heme oxygenase |
| PA14_57160 | *apbA* | | -3.77 | 5.53E-03 | -3.56 | 3.24E-03 | 2-dehydropantoate 2-reductase |
| PA14_57180* |  | | -2.95 | 2.26E-05 | -3.50 | 1.40E-07 | hypothetical protein |
| PA14_60380 | *ribF* | | 5.11 | 7.79E-25 | 2.72 | 8.05E-12 | bifunctional riboflavin kinase/FMN adenylyltransferase |
| PA14_60470* | *ispB* | | 3.89 | 2.04E-17 |  |  | octaprenyl-diphosphate synthase |
| PA14_60920 |  | |  |  | -4.41 | 2.33E-25 | hypothetical protein |
| PA14_61680 |  | | 3.10 | 3.57E-09 |  |  | methyl transferase |
| PA14_61710* | *hemA* | | 5.83 | 4.66E-28 |  |  | glutamyl-tRNA reductase |
| PA14_61750 | *ipk* | | 7.97 | 3.26E-38 | 4.56 | 5.51E-26 | 4-diphosphocytidyl-2-C-methyl-D-erythritol kinase |
| PA14_62150 | *ilvH* | |  |  | 2.87 | 1.10E-13 | acetolactate synthase 3 regulatory subunit |
| PA14_62570 | *folK* | | 3.27 | 1.37E-13 | 2.55 | 3.29E-10 | 2-amino-4-hydroxy-6- hydroxymethyldihydropteridine pyrophosphokinase |
| PA14_62580* | *panB* | | 8.00 | 1.39E-35 | 3.49 | 4.28E-15 | 3-methyl-2-oxobutanoate hydroxymethyltransferase |
| PA14_62590* | *panC* | | 8.25 | 1.54E-34 | 2.72 | 5.77E-09 | pantoate--beta-alanine ligase |
| PA14_62600 | *panD* | | 8.61 | 1.21E-26 |  |  | aspartate alpha-decarboxylase |
| PA14_62850 | *folP* | | 3.41 | 8.51E-16 |  |  | dihydropteroate synthase |
| PA14_64650 | *ureE* | |  |  | -3.29 | 3.27E-06 | urease accessory protein UreE |
| PA14_64660 | *ureF* | |  |  | -11.43 | 8.26E-22 | urease accessory protein UreF |
| PA14_64670 | *ureG* | |  |  | -12.03 | 1.63E-33 | urease accessory protein UreG |
| PA14_64680 | *ureJ* | |  |  | -4.49 | 3.82E-12 | hypothetical protein |
| PA14_64940 |  | | -5.68 | 5.84E-14 | -21.59 | 1.12E-32 | hypothetical protein |
| PA14_64960 | *pncB1* | | 7.27 | 3.73E-35 |  |  | nicotinate phosphoribosyltransferase |
| PA14_64980 | *nadE* | | 3.48 | 3.71E-16 | -3.09 | 7.81E-14 | NAD synthetase |
| PA14_66550^‡^ | *hemE* | | 8.19 | 7.66E-32 | 3.88 | 2.32E-16 | uroporphyrinogen decarboxylase |
| PA14_66920 | *ubiB* | | 3.52 | 5.89E-16 |  |  | ubiquinone biosynthesis protein UbiB |
| PA14_67580* | *thiI* | | 16.34 | 1.44E-63 | 5.27 | 6.87E-28 | thiamine biosynthesis protein ThiI |
| PA14_69150 | *ubiD* | | 3.71 | 4.23E-17 |  |  | hypothetical protein |
| PA14_69260* | *elbB* | | -2.96 | 2.54E-12 | -9.83 | 5.17E-53 | isoprenoid biosynthesis protein with amidotransferase-like domain |
| PA14_69420 | *hemY* | |  |  | -3.97 | 1.10E-22 | enzyme of heme biosynthesis |
| PA14_69430 | *hemX* | |  |  | -2.56 | 5.21E-10 | hypothetical protein |
| PA14_70240* | *coaC* | | 6.19 | 5.36E-31 |  |  | bifunctional phosphopantothenoylcysteine decarboxylase/phosphopantothenate synthase |
| PA14_72870 |  | | 2.58 | 5.15E-09 | 5.69 | 6.70E-31 | aminotransferase |
| PA14_72880 | *fabG* | |  |  | 2.57 | 4.59E-09 | short-chain dehydrogenase |
| PA14_72980 |  | | -4.22 | 2.06E-19 |  |  | G3E family GTPase |
| PA14_73010 |  | | -15.77 | 1.06E-61 |  |  | hypothetical protein |
| **Carbon compound catabolism** | | | | | |  |  |
| PA14_01900 | *pcaH* | |  |  | 3.19 | 4.49E-04 | protocatechuate 3,4-dioxygenase subunit beta |
| PA14_02550 | *mdcA* | | 2.93 | 1.86E-11 | 5.34 | 3.00E-16 | malonate decarboxylase subunit alpha |
| PA14_02760 | *catI* | | 4.04 | 2.53E-11 | 2.70 | 5.33E-06 | CoA transferase, subunit A |
| PA14_02830 | *pcaB* | |  |  | -2.56 | 6.59E-10 | 3-carboxy-cis,cis-muconate cycloisomerase |
| PA14_02840 | *pcaD* | |  |  | -2.99 | 8.32E-12 | beta-ketoadipate enol-lactone hydrolase |
| PA14_02850 | *pcaC* | |  |  | -5.08 | 2.45E-24 | gamma-carboxymuconolactone decarboxylase |
| PA14_03490 |  | | -7.95 | 1.29E-35 | -8.37 | 3.66E-43 | hypothetical protein |
| PA14_07930* | *gph* | | 4.50 | 1.26E-20 | 3.75 | 1.74E-18 | phosphoglycolate phosphatase |
| PA14_08440* |  | | 2.64 | 2.00E-09 |  |  | short chain alcohol dehydrogenase |
| PA14_09710 | *aldH* | |  |  | -6.05 | 2.31E-19 | aldehyde dehydrogenase |
| PA14_10230 | *adh* | | -3.90 | 4.84E-13 | -3.13 | 2.37E-10 | 2,3-butanediol dehydrogenase |
| PA14_10240 | *acoC* | | -3.10 | 2.06E-09 | -2.69 | 6.29E-08 | branched-chain alpha-keto acid dehydrogenase subunit E2 |
| PA14_10250 | *acoB* | | -2.79 | 1.71E-06 | -2.90 | 1.40E-06 | acetoin catabolism protein AcoB |
| PA14_10260 | *acoA* | | -5.19 | 2.23E-07 | -3.16 | 1.62E-05 | dehydrogenase E1 component |
| PA14_10570 | *hpcH* | | -10.21 | 3.56E-06 | -5.48 | 1.18E-04 | 2,4-dihydroxyhept-2-ene-1,7-dioic acid aldolase |
| PA14_10590 | *hpcG* | | -5.26 | 1.83E-12 | -3.56 | 1.62E-05 | 2-oxo-hepta-3-ene-1,7-dioic acid hydratase |
| PA14_10610 | *hpcD* | |  |  | -2.68 | 2.64E-05 | 5-carboxymethyl-2-hydroxymuconate isomerase |
| PA14_10960 |  | |  |  | -2.68 | 5.09E-05 | ferredoxin oxidoreductase subunit |
| PA14_12970 | *tauD* | |  |  | 32.17 | 1.31E-49 | taurine dioxygenase |
| PA14_14300 |  | | -3.68 | 2.11E-11 | 5.59 | 4.26E-05 | zinc-binding oxidoreductase |
| PA14_18140* | *mmsB* | | -16.25 | 1.49E-48 | -6.61 | 3.76E-28 | 3-hydroxyisobutyrate dehydrogenase |
| PA14_18830 |  | | 32.32 | 3.73E-36 |  |  | adenylosuccinate lyase |
| PA14_18850 |  | | 13.32 | 1.85E-37 |  |  | adenylosuccinate lyase |
| PA14_19350 |  | |  |  | -25.66 | 3.87E-92 | hypothetical protein |
| PA14_22890* | *gapA* | |  |  | -5.51 | 1.04E-11 | glyceraldehyde-3-phosphate dehydrogenase |
| PA14_22930 | *glk* | | 5.40 | 7.15E-28 | -4.00 | 1.26E-20 | glucokinase |
| PA14_23070 | *zwf* | | 8.49 | 7.18E-42 | -3.78 | 3.02E-05 | glucose-6-phosphate 1-dehydrogenase |
| PA14_23090 | *edaA* | | 6.81 | 8.55E-35 | -5.92 | 4.39E-29 | keto-hydroxyglutarate-aldolase/keto-deoxy- phosphogluconate aldolase |
| PA14_23620 | *kdgA* | |  |  | 2.82 | 5.52E-11 | aldolase |
| PA14_24960 |  | |  |  | -2.89 | 4.75E-12 | carbohydrate kinase |
| PA14_25250* | *gapA* | | 5.26 | 4.89E-24 | 3.93 | 5.92E-22 | glyceraldehyde-3-phosphate dehydrogenase |
| PA14_26130 | *morB* | | 14.23 | 5.44E-05 | 49.61 | 6.20E-112 | morphinone reductase |
| PA14_30810 | *alkB1* | | -2.75 | 4.46E-09 |  |  | alkane-1 monooxygenase |
| PA14_32130 | *benD* | |  |  | -3.12 | 4.31E-03 | 1,6-dihydroxycyclohexa-2,4-diene-1-carboxylate dehydrogenase |
| PA14_32220 | *catB* | |  |  | 3.67 | 1.21E-04 | muconate cycloisomerase I |
| PA14_33450 | *treA* | | -7.74 | 6.07E-28 | -8.31 | 6.38E-33 | trehalase |
| PA14_33480 | *sndH* | | -12.34 | 2.00E-32 | -28.81 | 5.25E-52 | L-sorbosone dehydrogenase |
| PA14_34190 | *msuD* | |  |  | 21.49 | 1.63E-12 | methanesulfonate sulfonatase MsuD |
| PA14_34600* | *gapB* | | 2.74 | 9.75E-11 | -4.88 | 2.25E-22 | glyceraldehyde-3-phosphate dehydrogenase |
| PA14_34640 | *gntK* | | 19.15 | 4.90E-66 |  |  | gluconokinase |
| PA14_34870 | *chiC* | | -132.00 | 2.25E-152 | -8.75 | 3.27E-50 | chitinase |
| PA14_34970 | *gcd* | | -3.94 | 2.25E-18 | -21.31 | 1.21E-85 | glucose dehydrogenase |
| PA14_35290 | *gnd* | |  |  | -6.01 | 6.18E-33 | gluconate dehydrogenase |
| PA14_35340 | *kguK* | | -3.71 | 2.00E-09 | -9.68 | 5.84E-21 | 2-ketogluconate kinase |
| PA14_35360 | *kguE* | |  |  | -7.40 | 3.25E-25 | hypothetical protein |
| PA14_35600 | *pslL* | | -3.52 | 1.12E-10 |  |  | hypothetical protein |
| PA14_35670 | *pslG* | | -5.25 | 3.05E-21 | -3.17 | 5.08E-13 | glycosyl hydrolase |
| PA14_36500 |  | | -4.94 | 4.54E-20 | -5.25 | 6.79E-24 | hypothetical protein |
| PA14_36570 | *glgA* | | -7.06 | 1.36E-29 | -4.70 | 1.21E-22 | glycogen synthase |
| PA14_36590 | *malQ* | | -9.13 | 9.65E-37 | -14.41 | 7.28E-53 | 4-alpha-glucanotransferase |
| PA14_36630 | *glgX* | | -17.86 | 5.25E-62 | -16.27 | 1.53E-65 | glycosyl hydrolase |
| PA14_36730 |  | | -7.99 | 3.64E-36 | -16.54 | 6.82E-68 | trehalose synthase |
| PA14_37550 |  | |  |  | 5.87 | 2.35E-04 | ring-hydroxylating dioxygenase small subunit |
| PA14_37570 |  | | 2.51 | 2.40E-08 | 4.10 | 4.07E-20 | ring-hydroxylating dioxygenase, large terminal subunit |
| PA14_37770 |  | | -13.10 | 6.26E-50 |  |  | hydrolase |
| PA14_38460* | *gnyB* | |  |  | -6.96 | 7.25E-31 | acyl-CoA carboxyltransferase subunit beta |
| PA14_38470 | *gnyH* | |  |  | -7.13 | 6.15E-40 | gamma-carboxygeranoyl-CoA hydratase |
| PA14_38480* | *gnyA* | | -2.56 | 1.47E-09 | -9.19 | 4.55E-51 | alpha subunit of geranoyl-CoA carboxylase, GnyA |
| PA14_38490 | *gnyL* | | -3.68 | 1.64E-16 | -12.49 | 5.52E-62 | hydroxymethylglutaryl-CoA lyase |
| PA14_38530^‡^* | *fahA* | |  |  | -4.10 | 1.01E-23 | fumarylacetoacetase |
| PA14_38550^‡^* | *maiA* | |  |  | -4.16 | 9.44E-13 | maleylacetoacetate isomerase |
| PA14_38590 | *bdhA* | | -12.13 | 1.96E-48 |  |  | 3-hydroxybutyrate dehydrogenase |
| PA14_38860 | *exaA* | | -6.90 | 5.31E-19 |  |  | quinoprotein alcohol dehydrogenase |
| PA14_39280 | *rbsK* | |  |  | -3.68 | 5.92E-19 | ribokinase |
| PA14_39300 | *rbsR* | |  |  | -3.58 | 1.21E-18 | ribose operon repressor RbsR |
| PA14_42230 | *bglX* | | 3.31 | 1.29E-13 | 3.11 | 5.72E-14 | beta-glucosidase |
| PA14_42740 |  | |  |  | 2.51 | 2.20E-10 | methylthioribulose-1-phosphate dehydratase |
| PA14_44700 | *alkB2* | | -2.97 | 1.50E-02 |  |  | alkane-1 monooxygenase |
| PA14_44830 |  | | -8.17 | 1.19E-35 | 2.89 | 2.01E-14 | hypothetical protein |
| PA14_44850 | *alc* | | -10.45 | 7.81E-20 | -4.18 | 2.81E-12 | allantoicase |
| PA14_45030 | *ttuD* | | -2.87 | 4.92E-06 |  |  | hypothetical protein |
| PA14_51120 |  | |  |  | -5.41 | 2.87E-15 | acyl-CoA dehydrogenase |
| PA14_53220* | *fumC2* | | -2.51 | 2.34E-09 | -8.43 | 2.69E-47 | fumarate hydratase |
| PA14_53480 | *pta* | |  |  | -8.67 | 4.79E-11 | phosphate acetyltransferase |
| PA14_53940 | *prpB* | |  |  | 4.64 | 2.91E-27 | 2-methylisocitrate lyase |
| PA14_53950* | *prpC* | | -6.06 | 5.69E-06 |  |  | methylcitrate synthase |
| PA14_54000* | *prpD* | | -34.99 | 3.09E-93 | -7.32 | 5.50E-43 | 2-methylcitrate dehydratase |
| PA14_54670 |  | | -10.36 | 2.65E-46 | -16.65 | 2.47E-68 | 3-hydroxyisobutyrate dehydrogenase |
| PA14_57330 | *murC* | |  |  | -16.42 | 6.72E-75 | UDP-N-acetylmuramate--L-alanine ligase |
| PA14_57340* | *murG* | |  |  | -13.67 | 6.77E-66 | UDPdiphospho-muramoylpentapeptide beta-N- acetylglucosaminyltransferase |
| PA14_57890 | *yrbH* | | 3.56 | 4.76E-16 |  |  | hypothetical protein |
| PA14_60750 | *pra* | | -7.64 | 3.89E-17 | -24.70 | 1.86E-34 | protein activator |
| PA14_64800 | *vanA* | |  |  | 2.89 | 5.01E-06 | vanillate O-demethylase oxygenase |
| PA14_64810 | *vanB* | | -7.21 | 4.93E-23 | -5.06 | 4.52E-17 | vanillate O-demethylase |
| PA14_66830 | *phaD* | | -3.91 | 2.82E-08 | -3.78 | 2.31E-09 | poly(3-hydroxyalkanoic acid) depolymerase |
| PA14_70550 |  | | 2.56 | 8.94E-09 |  |  | hypothetical protein |
| PA14_70640 | *rubA1* | | 3.07 | 3.60E-10 | 2.79 | 9.45E-10 | rubredoxin 1 |
| PA14_70690 | *glcD* | |  |  | 2.82 | 2.68E-09 | glycolate oxidase subunit GlcD |
| PA14_71570 |  | |  |  | -3.25 | 1.59E-08 | hypothetical protein |
| PA14_71630^‡^ | *adhA* | |  |  | -22.85 | 4.12E-59 | alcohol dehydrogenase |
| **Cell division** |  | |  |  |  |  |  |
| PA14_04910 | *ftsE* | | 6.11 | 4.10E-30 | 2.60 | 6.43E-11 | cell division ATP-binding protein FtsE |
| PA14_17330 |  | |  |  | 2.85 | 1.66E-13 | hypothetical protein |
| PA14_22020* | *minD* | | 2.71 | 1.49E-10 | 2.62 | 1.33E-11 | cell division inhibitor MinD |
| PA14_22800 | *yciB* | | 2.54 | 3.08E-09 | 3.09 | 5.98E-15 | intracellular septation protein A |
| PA14_25610 | *maf1* | | 2.51 | 1.93E-05 | 2.54 | 1.24E-05 | Maf-like protein |
| PA14_30290 | *ftsK* | | 3.22 | 1.33E-14 |  |  | cell division protein FtsK |
| PA14_41250^‡^ | *tig* | | 2.58 | 2.23E-10 | 4.28 | 2.18E-25 | trigger factor |
| PA14_48870* | *ydaO* | | 3.93 | 4.13E-18 | 3.04 | 2.55E-14 | C32 tRNA thiolase |
| PA14_57275 | *ftsZ* | | -4.93 | 3.98E-24 | -47.71 | 8.81E-124 | cell division protein FtsZ |
| PA14_57290* | *ftsA* | |  |  | -18.41 | 1.15E-79 | cell division protein FtsA |
| PA14_57300 | *ftsQ* | |  |  | -15.84 | 7.41E-72 | cell division protein FtsQ |
| PA14_57360 | *ftsW* | |  |  | -9.49 | 7.24E-33 | cell division protein FtsW |
| PA14_57440 | *ftsL* | |  |  | -6.30 | 2.98E-34 | cell division protein FtsL |
| PA14_62870 | *rrmJ* | | 4.25 | 9.21E-21 | 2.98 | 1.76E-14 | cell division protein FtsJ |
| PA14_63200 |  | | -3.36 | 4.89E-14 | -3.81 | 6.32E-20 | hypothetical protein |
| PA14_66760 |  | | 3.19 | 1.91E-13 |  |  | hypothetical protein |
| PA14_67790 |  | | 2.83 | 4.08E-11 |  |  | membrane-bound metallopeptidase |
| PA14_73350 | *soj* | | 2.96 | 1.42E-12 | 2.64 | 5.05E-12 | chromosome partitioning protein Soj |
| PA14_73360* | *gidB* | | 2.81 | 2.37E-11 |  |  | 16S rRNA methyltransferase GidB |
| PA14_73370 | *gidA* | | 2.63 | 2.08E-10 |  |  | tRNA uridine 5-carboxymethylaminomethyl modification protein GidA |
| **Cell wall / LPS / capsule** | | |  |  |  |  |  |
| PA14_00570 |  | | 21.05 | 8.68E-29 | 3.36 | 1.33E-10 | lipoprotein |
| PA14_04950 | *mtgA* | | 4.36 | 1.76E-16 |  |  | monofunctional biosynthetic peptidoglycan transglycosylase |
| PA14_07790 |  | |  |  | -4.01 | 3.25E-21 | nucleotidyl transferase |
| PA14_12060 | *pbpA* | | 5.75 | 5.46E-19 | 3.40 | 1.68E-17 | penicillin-binding protein 2 |
| PA14_12070 | *rodA* | | 3.28 | 3.38E-14 | 2.79 | 1.09E-12 | rod shape-determining protein |
| PA14_12080 | *sltB1* | | 3.44 | 1.06E-15 |  |  | soluble lytic transglycosylase B |
| PA14_12280 | *lnt* | | 7.24 | 1.47E-33 |  |  | apolipoprotein N-acyltransferase |
| PA14_17110 | *uppS* | | 3.34 | 5.09E-08 | 2.91 | 7.84E-10 | UDP pyrophosphate synthetase |
| PA14_17150 |  | | 5.02 | 3.24E-26 | 4.04 | 1.51E-23 | outer membrane antigen |
| PA14_17180 | *lpxD* | | 2.71 | 1.32E-10 |  |  | UDP-3-O-[3-hydroxymyristoyl] glucosamine N-acyltransferase |
| PA14_17210 | *lpxA* | | 2.76 | 4.30E-11 |  |  | UDP-N-acetylglucosamine acyltransferase |
| PA14_17220 | *lpxB* | | 6.38 | 9.90E-32 | 3.81 | 3.48E-20 | lipid-A-disaccharide synthase |
| PA14_17310 | *kdsA* | | 6.97 | 2.64E-35 | 3.50 | 1.30E-18 | 2-dehydro-3-deoxyphosphooctonate aldolase |
| PA14_18380 | *algA* | | -6.30 | 3.00E-26 | -10.79 | 2.81E-42 | mannose-1-phosphate guanylyltransferase |
| PA14_18410 | *algF* | | -4.67 | 2.26E-11 | -7.96 | 1.62E-17 | alginate o-acetyltransferase AlgF |
| PA14_18430 | *algJ* | |  |  | -2.80 | 5.89E-04 | alginate o-acetyltransferase AlgJ |
| PA14_18470 | *algL* | | -2.86 | 4.34E-06 | -3.19 | 1.43E-06 | poly(beta-D-mannuronate) lyase |
| PA14_19170 | *yafL* | | 13.65 | 3.00E-19 | 26.99 | 1.63E-82 | hypothetical protein |
| PA14_20270 | *rhlG* | | 5.09 | 2.42E-18 |  |  | beta-ketoacyl reductase |
| PA14_20890* | *rfaD* | | -6.72 | 1.42E-32 | -23.05 | 9.20E-90 | ADP-L-glycero-D-manno-heptose-6-epimerase |
| PA14_22050 | *htrB* | | 5.30 | 1.56E-23 | 2.60 | 8.88E-08 | lipid A biosynthesis lauroyl acyltransferase |
| PA14_23440 | *orfL* | |  |  | -3.37 | 2.58E-05 | group 1 glycosyl transferase |
| PA14_23450 | *orfM* | |  |  | -4.24 | 1.04E-11 | NAD dependent epimerase/dehydratase |
| PA14_25510* | *lpxK* | | 4.56 | 1.90E-21 | 3.33 | 1.84E-10 | tetraacyldisaccharide 4'-kinase |
| PA14_25530 | *kdsB* | | 3.42 | 2.26E-15 | 2.52 | 6.55E-07 | 3-deoxy-manno-octulosonate cytidylyltransferase |
| PA14_25550 | *murB* | | 2.75 | 2.26E-10 |  |  | UDP-N-acetylenolpyruvoylglucosamine reductase |
| PA14_35640 | *pslI* | | -4.72 | 4.34E-15 | -4.14 | 2.11E-13 | transferase |
| PA14_35650 | *pslH* | | -5.65 | 6.61E-22 | -5.66 | 2.65E-23 | hypothetical protein |
| PA14_36840 | *glgP* | | -6.36 | 5.62E-28 | -7.38 | 1.14E-35 | glycogen phosphorylase |
| PA14_41090 | *mltD* | | 3.45 | 4.89E-13 | 6.98 | 9.56E-39 | membrane-bound lytic murein transglycosylase D |
| PA14_48520 | *mltA* | | 3.91 | 2.27E-16 | 2.84 | 3.05E-11 | membrane-bound lytic murein transglycosylase A |
| PA14_52150* | *lpxO2* | |  |  | 3.62 | 8.42E-19 | lipopolysaccharide biosynthetic protein LpxO2 |
| PA14_57260* | *lpxC* | |  |  | -2.69 | 6.66E-13 | UDP-3-O-[3-hydroxymyristoyl] N-acetylglucosamine deacetylase |
| PA14_57320* | *ddl* | |  |  | -14.40 | 3.73E-68 | D-alanine--D-alanine ligase |
| PA14_57370 | *murD* | |  |  | -8.93 | 8.21E-50 | UDP-N-acetylmuramoyl-L-alanyl-D-glutamate synthetase |
| PA14_57380 | *mraY* | |  |  | -6.88 | 9.57E-40 | phospho-N-acetylmuramoyl-pentapeptide- transferase |
| PA14_57390 | *murF* | |  |  | -9.18 | 4.72E-51 | UDP-N-acetylmuramoylalanyl-D-glutamyl-2,6- diaminopimelate--D-alanyl-D-alanine ligase |
| PA14_57410 | *murE* | |  |  | -8.66 | 3.00E-39 | UDP-N-acetylmuramoylalanyl-D-glutamate--2, 6-diaminopimelate ligase |
| PA14_57425 | *ftsI* | |  |  | -5.32 | 1.24E-31 | penicillin-binding protein 3 |
| PA14_57450 | *mraW* | |  |  | -4.95 | 3.53E-29 | S-adenosyl-methyltransferase MraW |
| PA14_57810* | *murA* | | 4.08 | 7.02E-20 |  |  | UDP-N-acetylglucosamine 1-carboxyvinyltransferase |
| PA14_58120 | *mreD* | | 35.83 | 1.43E-73 | 14.49 | 9.19E-10 | rod shape-determining protein MreD |
| PA14_58130 | *mreC* | | 12.29 | 4.19E-53 | 3.33 | 1.06E-15 | rod shape-determining protein MreC |
| PA14_58150^‡^* | *mreB* | | 15.23 | 5.49E-62 | 5.68 | 1.90E-31 | rod shape-determining protein MreB |
| PA14_58550 | *lpxO1* | | 2.69 | 1.31E-09 | 5.19 | 3.06E-14 | lipopolysaccharide biosynthetic protein LpxO1 |
| PA14_60230 | *comL* | | 5.96 | 2.55E-20 | 2.91 | 7.04E-13 | competence protein ComL |
| PA14_61740 | *lolB* | | 6.56 | 1.56E-30 | 4.13 | 3.97E-20 | outer membrane lipoprotein LolB |
| PA14_62200 | *mrcB* | | 7.18 | 6.96E-36 | 2.63 | 1.25E-11 | penicillin-binding protein 1B |
| PA14_63030 | *omlA* | | 6.84 | 4.85E-33 | 6.94 | 8.75E-38 | outer membrane lipoprotein OmlA precursor |
| PA14_65960 | *waaA* | | 3.49 | 9.26E-15 |  |  | 3-deoxy-D-manno-octulosonic-acid transferase |
| PA14_66100 |  | | 2.67 | 6.71E-08 |  |  | hypothetical protein |
| PA14_66220 | *waaP* | | 3.63 | 6.44E-17 |  |  | lipopolysaccharide kinase WaaP |
| PA14_66230 | *waaG* | | 4.16 | 1.75E-20 |  |  | UDP-glucose:(heptosyl) LPS alpha 1,3-glucosyltransferase WaaG |
| PA14_66240* | *waaC* | | 3.58 | 1.45E-16 |  |  | lipopolysaccharide heptosyltransferase I |
| PA14_66250* | *waaF* | | 2.67 | 1.35E-10 |  |  | heptosyltransferase II |
| PA14_66670 | *ponA* | | 3.20 | 3.92E-14 |  |  | penicillin-binding protein 1A |
| PA14_67065 | *mdoH* | | 2.92 | 1.06E-12 |  |  | glucosyltransferase MdoH |
| PA14_67450 | *blc* | |  |  | -3.49 | 2.65E-18 | outer membrane lipoprotein Blc |
| PA14_68170* | *rmlB* | |  |  | 2.67 | 3.42E-08 | dTDP-D-glucose 4,6-dehydratase |
| PA14_68400 | *ygaU* | | -8.90 | 8.81E-42 | -26.43 | 5.64E-97 | LysM domain/BON superfamily protein |
| PA14_71910 | *wbpZ* | |  |  | -3.02 | 3.42E-15 | glycosyltransferase WbpZ |
| PA14_71920 | *wbpY* | |  |  | -3.71 | 9.52E-20 | glycosyltransferase WbpY |
| PA14_71930 | *wbpX* | |  |  | -6.72 | 2.23E-36 | glycosyltransferase WbpX |
| PA14_71940 | *wzt* | |  |  | -3.90 | 2.38E-19 | ABC subunit of A-band LPS efflux transporter |
| PA14_71960 | *wzm* | | 3.50 | 6.40E-16 |  |  | membrane subunit of A-band LPS efflux transporter |
| PA14_71970 | *wbpW* | |  |  | -3.36 | 3.34E-17 | GDP-mannose pyrophosphorylase |
| PA14_72010 |  | | 4.14 | 3.81E-20 |  |  | glycosyltransferase |
| PA14_73040 | *amiA* | | -94.84 | 2.47E-135 | -6.33 | 6.58E-38 | N-acetylmuramoyl-L-alanine amidase |
| **Central intermediary metabolism** | | | |  |  |  |  |
| PA14_01310 | *coxG* | | -7.90 | 7.71E-07 | -5.33 | 2.58E-06 | cytochrome C oxidase assembly protein |
| PA14_03430^‡^* | *gabD* | | 2.58 | 4.78E-10 |  |  | succinate-semialdehyde dehydrogenase I |
| PA14_03980 |  | | 2.96 | 1.30E-11 |  |  | hypothetical protein |
| PA14_04150* | *ycgM* | |  |  | -3.63 | 1.20E-19 | hypothetical protein |
| PA14_04580 | *folA* | | 4.25 | 4.42E-13 |  |  | dihydrofolate reductase |
| PA14_04760 | *coaD* | | 7.16 | 3.70E-30 | 4.32 | 1.68E-18 | phosphopantetheine adenylyltransferase |
| PA14_07910 | *rpe* | | 8.38 | 6.52E-32 | 3.63 | 1.90E-12 | ribulose-phosphate 3-epimerase |
| PA14_09210 | *pchA* | | -15.60 | 6.26E-62 | -7.86 | 4.48E-46 | salicylate biosynthesis isochorismate synthase |
| PA14_09220 | *pchB* | | -12.86 | 3.52E-54 | -8.09 | 2.02E-46 | isochorismate-pyruvate lyase |
| PA14_09240 | *pchD* | | -11.55 | 1.40E-07 | -6.26 | 2.58E-06 | pyochelin biosynthesis protein PchD |
| PA14_09290 | *pchG* | | -28.01 | 2.40E-59 | -3.84 | 3.25E-20 | pyochelin biosynthetic protein PchG |
| PA14_10550 | *cysI* | | -2.50 | 2.17E-09 |  |  | sulfite or nitrite reductas |
| PA14_11760 | *eutC* | | -4.90 | 6.65E-22 | -12.18 | 2.12E-51 | ethanolamine ammonia-lyase small subunit |
| PA14_11770^‡^ | *eutB* | | -5.01 | 1.00E-09 | -11.02 | 1.62E-17 | ethanolamine ammonia-lyase large subunit |
| PA14_17040 | *glnD* | | 6.84 | 4.13E-34 | 3.49 | 6.17E-18 | PII uridylyl-transferase |
| PA14_17400 | *adhC* | |  |  | -4.38 | 2.68E-03 | alcohol dehydrogenase |
| PA14_18010 | *glpK* | | 11.31 | 1.07E-19 | 2.56 | 3.95E-09 | glycerol kinase |
| PA14_19190* | *sfcA* | | 2.59 | 1.66E-09 | 2.82 | 8.01E-13 | malate dehydrogenase |
| PA14_19530 | *ssuE* | | 3.57 | 8.62E-08 | 209.13 | 1.06E-161 | NAD(P)H-dependent FMN reductase |
| PA14_19560 | *ssuD* | |  |  | 27.46 | 1.67E-82 | alkanesulfonate monooxygenase |
| PA14_19590 | *ssuF* | | -23.03 | 2.12E-19 | 7.40 | 2.85E-43 | molybdopterin-binding protein |
| PA14_23080 | *pgl* | | 9.84 | 1.26E-46 | -6.82 | 1.25E-30 | 6-phosphogluconolactonase |
| PA14_28060 | *cpg2* | | -8.78 | 2.47E-04 | -5.72 | 5.96E-04 | glutamate carboxypeptidase |
| PA14_29020 | *cpo* | | -23.15 | 4.52E-74 | -16.59 | 2.00E-70 | chloroperoxidase |
| PA14_33730 |  | | -8.27 | 1.15E-35 |  |  | dipeptidase |
| PA14_34180 | *msuE* | |  |  | 38.62 | 2.12E-08 | NADH-dependent FMN reductase MsuE |
| PA14_34290 |  | |  |  | 19.69 | 6.85E-11 | DszA family monooxygenase |
| PA14_34750 | *tauD* | | -3.21 | 9.51E-06 | 11.46 | 1.03E-49 | taurine catabolism dioxygenase |
| PA14_36320* | *hcnB* | | 3.27 | 2.19E-14 |  |  | hydrogen cyanide synthase HcnB |
| PA14_36330 | *hcnA* | | 3.36 | 3.06E-08 |  |  | hydrogen cyanide synthase HcnA |
| PA14_36870 |  | | -10.88 | 3.35E-36 | -8.86 | 2.31E-32 | short-chain dehydrogenase |
| PA14_36890 |  | |  |  | -8.48 | 1.34E-09 | metallothionein |
| PA14_37070* |  | |  |  | -14.78 | 1.03E-18 | hypothetical protein |
| PA14_37340 |  | | -6.14 | 2.99E-24 | -4.09 | 1.13E-17 | thiamine pyrophosphate protein |
| PA14_37965 | *cynS* | | -6.65 | 4.78E-07 | -2.87 | 2.33E-04 | cyanate hydratase |
| PA14_38200 | *ilvG* | | -3.33 | 1.07E-12 |  |  | thiamine pyrophosphate protein |
| PA14_38210 |  | | -26.95 | 3.64E-56 | -15.59 | 1.49E-49 | hypothetical protein |
| PA14_40040 |  | | -3.27 | 2.67E-10 | -3.31 | 5.82E-11 | hypothetical protein |
| PA14_40770 | *cysI* | | 3.87 | 1.29E-16 | 31.97 | 4.83E-55 | sulfite reductase |
| PA14_41080 |  | | -2.51 | 6.26E-03 |  |  | hydroxyacylglutathione hydrolase |
| PA14_41530 | *nirB* | | -3.49 | 1.48E-08 |  |  | assimilatory nitrite reductase large subunit |
| PA14_41840* | *cysH* | |  |  | 12.44 | 3.04E-55 | phosphoadenosine phosphosulfate reductase |
| PA14_44860 | *allA* | | -3.74 | 2.21E-05 | -2.51 | 4.27E-04 | ureidoglycolate hydrolase |
| PA14_45000 | *gcl* | |  |  | 52.93 | 3.98E-118 | glyoxylate carboligase |
| PA14_45010 | *hyi* | | -5.69 | 2.62E-16 | 3.14 | 8.47E-13 | hydroxypyruvate isomerase |
| PA14_45470 |  | | -2.80 | 5.94E-06 |  |  | hypothetical protein |
| PA14_46630 |  | | 2.97 | 2.07E-07 | 3.95 | 5.06E-12 | hypothetical protein |
| PA14_46960 | *ggt* | | -2.59 | 1.09E-09 | -12.07 | 1.71E-58 | gamma-glutamyltranspeptidase |
| PA14_48020 |  | | -4.02 | 2.46E-16 |  |  | L-malate dehydrogenase |
| PA14_48620 |  | | -9.87 | 7.87E-32 | -17.10 | 7.61E-43 | clavaminic acid synthetase |
| PA14_52800 | *acsA* | | -4.04 | 9.95E-19 | 3.42 | 9.56E-19 | acetyl-CoA synthetase |
| PA14_55130 | *gloA2* | |  |  | -2.59 | 4.35E-04 | lactoylglutathione lyase |
| PA14_61210 | *hprA* | | 5.27 | 7.65E-22 | 3.85 | 6.75E-16 | glycerate dehydrogenase |
| PA14_61400 | *mqoB* | | 2.51 | 1.93E-09 | 8.28 | 8.44E-43 | malate:quinone oxidoreductase |
| PA14_62830 | *tpiA* | | 8.33 | 3.92E-42 | 4.41 | 7.09E-26 | triosephosphate isomerase |
| PA14_64335 | *ureD* | | 4.75 | 4.64E-18 |  |  | urease accessory protein |
| PA14_64350 | *ureA* | | 3.01 | 8.62E-09 |  |  | urease subunit gamma |
| PA14_64390* | *ureC* | |  |  | -2.69 | 1.85E-11 | urease subunit alpha |
| PA14_64520 |  | |  |  | -2.65 | 1.11E-05 | bacterioferritin |
| PA14_64950* | *pncA* | | 11.04 | 3.92E-39 |  |  | hypothetical protein |
| PA14_65480 | *rhdA* | | 5.24 | 1.37E-23 |  |  | thiosulfate sulfurtransferase |
| PA14_66170* |  | | 2.71 | 4.28E-11 |  |  | carbamoyl transferase |
| PA14_66820 | *phaC1* | | -2.95 | 5.76E-12 |  |  | poly(3-hydroxyalkanoic acid) synthase 1 |
| PA14_66840 | *phaC2* | | -8.93 | 5.05E-14 | -10.73 | 1.56E-17 | poly(3-hydroxyalkanoic acid) synthase 2 |
| PA14_66875 | *phaF* | | -4.34 | 1.12E-20 | -12.84 | 1.61E-65 | polyhydroxyalkanoate synthesis protein PhaF |
| PA14_67260* |  | |  |  | -3.43 | 1.00E-16 | histidine/phenylalanine ammonia-lyase |
| PA14_67320 | *hutH* | |  |  | 11.49 | 2.08E-51 | histidine ammonia-lyase |
| PA14_67500 | *gloA3* | |  |  | -3.29 | 1.75E-16 | lactoylglutathione lyase |
| PA14_69810 | *glnK* | | -5.79 | 4.45E-28 | -3.21 | 5.11E-17 | nitrogen regulatory protein P-II 2 |
| PA14_69925 | *poxB* | |  |  | -3.17 | 9.26E-14 | pyruvate dehydrogenase (cytochrome) |
| PA14_70270 | *algC* | | 2.58 | 5.84E-10 |  |  | phosphomannomutase |
| PA14_70720 | *ubiC* | | 4.73 | 3.28E-20 |  |  | hypothetical protein |
| PA14_71720^‡^* | *oadA* | |  |  | -6.06 | 3.89E-17 | pyruvate carboxylase subunit B |
| PA14_71740 | *accC* | | 2.73 | 6.36E-11 |  |  | pyruvate carboxylase subunit A |
| **Chaperones & heat shock proteins** | | | |  |  |  |  |
| PA14_03610 | *ycaL* | | 7.56 | 1.20E-05 | 2.58 | 1.62E-05 | Zn-dependent protease with chaperone function |
| PA14_14330* |  | | 3.57 | 2.76E-08 |  |  | chaperone |
| PA14_14770 | *hscB* | | 3.43 | 4.73E-10 |  |  | co-chaperone HscB |
| PA14_14780 | *hscA* | | 3.26 | 7.64E-09 |  |  | chaperone protein HscA |
| PA14_16050 | *dsbC* | | 4.36 | 1.17E-20 | 3.19 | 1.08E-12 | thiol:disulfide interchange protein DsbC |
| PA14_23680^‡^ | *ibpA* | | 9.74 | 7.11E-24 | 14.11 | 4.14E-55 | heat-shock protein IbpA |
| PA14_41190 | *ppiD* | | 4.38 | 8.99E-22 |  |  | peptidyl-prolyl cis-trans isomerase D |
| PA14_43850^‡^ | *htpG* | | 9.82 | 1.36E-46 | 3.40 | 1.80E-17 | heat shock protein 90 |
| PA14_50590* |  | | 4.64 | 3.04E-22 |  |  | HSP90 family protein |
| PA14_51520 | *spcU* | | -6.63 | 2.25E-12 | -4.57 | 3.54E-11 | SpcU |
| PA14_55280 |  | |  |  | -2.60 | 3.49E-06 | peptidyl-prolyl cis-trans isomerase, PpiC-type |
| PA14_57010^‡^* | *groEL* | | 4.29 | 1.02E-21 |  |  | chaperonin GroEL |
| PA14_57020 | *groES* | | 7.61 | 1.66E-38 |  |  | co-chaperonin GroES |
| PA14_60190* | *clpB* | |  |  | -3.35 | 2.73E-18 | clpB protein |
| PA14_62960* | *dnaJ* | | 5.40 | 3.23E-27 |  |  | chaperone protein DnaJ |
| PA14_62970^‡^ | *dnaK* | | 7.01 | 5.89E-36 |  |  | molecular chaperone DnaK |
| PA14_62990 | *grpE* | | 17.43 | 7.61E-66 | 8.58 | 2.72E-44 | heat shock protein GrpE |
| PA14_64080 | *dipZ* | | 7.97 | 6.62E-35 |  |  | thiol:disulfide interchange protein |
| PA14_66770 | *hslV* | | 28.26 | 6.72E-59 | 11.53 | 5.30E-49 | ATP-dependent protease peptidase subunit |
| PA14_66790 | *hslU* | | 15.17 | 1.20E-35 | 3.73 | 1.71E-18 | ATP-dependent protease ATP-binding subunit HslU |
| PA14_68610 | *hslO* | | 7.38 | 1.65E-32 | 3.40 | 4.15E-14 | Hsp33-like chaperonin |
| **Chemotaxis** |  | |  |  |  |  |  |
| PA14_02180 | *cheB* | | -4.07 | 9.08E-16 | -6.57 | 1.62E-27 | chemotaxis-specific methylesterase |
| PA14_02190 | *cheD* | | -2.62 | 3.84E-08 | -7.81 | 1.53E-28 | hypothetical protein |
| PA14_02200 | *cheR* | | -4.40 | 4.03E-20 | -18.33 | 1.47E-69 | chemotaxis protein methyltransferase |
| PA14_02220 |  | | -8.06 | 2.04E-38 | -31.23 | 6.10E-103 | chemotaxis transducer |
| PA14_02230 | *cheW* | | -9.96 | 7.72E-45 | -42.22 | 1.72E-110 | purine-binding chemotaxis protein |
| PA14_02270 |  | | -5.23 | 4.43E-09 | -3.89 | 1.89E-08 | chemotaxis transducer |
| PA14_05340* | *pilI* | | 4.32 | 2.03E-20 |  |  | twitching motility protein PilI |
| PA14_05360 | *pilJ* | | 5.39 | 7.14E-28 |  |  | twitching motility protein PilJ |
| PA14_05380 | *pilK* | | 4.70 | 1.06E-23 |  |  | methyltransferase PilK |
| PA14_05390* | *chpA* | |  |  | -2.95 | 7.15E-15 | ChpA |
| PA14_05410 | *chpC* | |  |  | -2.96 | 1.21E-12 | chemotaxis protein |
| PA14_05430 | *chpE* | | 2.79 | 3.93E-06 |  |  | chemotaxis protein |
| PA14_16440 | *wspB* | |  |  | -4.03 | 1.86E-21 | hypothetical protein |
| PA14_16450 | *wspC* | |  |  | -3.81 | 9.14E-20 | methyltransferase |
| PA14_16460 | *wspD* | |  |  | -3.05 | 3.64E-10 | CheW domain-containing protein |
| PA14_16470 | *wspE* | |  |  | -5.44 | 3.71E-32 | chemotaxis sensor/effector fusion protein |
| PA14_16480 | *wspF* | |  |  | -2.97 | 9.68E-15 | chemotaxis-specific methylesterase |
| PA14_16500 | *wspR* | | -4.02 | 2.10E-18 | -4.19 | 8.65E-24 | two-component response regulator |
| PA14_20750* |  | | -3.82 | 5.43E-18 | -4.57 | 1.30E-24 | chemotaxis protein |
| PA14_20760 | *cheR* | |  |  | -3.66 | 1.82E-20 | chemotaxis protein methyltransferase |
| PA14_26280 |  | | -11.60 | 8.62E-51 | -5.65 | 6.32E-34 | chemotaxis transducer |
| PA14_27000 |  | |  |  | -5.28 | 3.01E-09 | chemotaxis transducer |
| PA14_28050 |  | | -6.81 | 1.59E-24 | -7.39 | 6.24E-30 | chemotaxis transducer |
| PA14_30820 |  | | -47.69 | 1.22E-106 | -28.81 | 2.98E-99 | methyl-accepting chemotaxis transducer |
| PA14_31400 | *ctpH* | |  |  | 2.55 | 8.94E-09 | chemotaxis transducer |
| PA14_39560 |  | | -62.89 | 6.87E-49 | -9.29 | 1.17E-24 | chemotaxis transducer |
| PA14_44300 | *aer* | | -2.68 | 1.25E-10 | -7.96 | 1.21E-46 | aerotaxis receptor Aer |
| PA14_45500 | *cheW* | | -2.63 | 2.59E-09 | -3.74 | 7.56E-16 | purine-binding chemotaxis protein |
| PA14_45540 | *motD* | |  |  | -3.30 | 3.91E-17 | flagellar motor protein MotD |
| PA14_45560 | *motC* | |  |  | -3.35 | 3.38E-17 | flagellar motor protein |
| PA14_45580* | *cheB* | |  |  | -3.52 | 4.17E-19 | chemotaxis-specific methylesterase |
| PA14_45610 | *cheZ* | |  |  | -3.29 | 1.20E-17 | chemotaxis protein CheZ |
| PA14_46030 |  | | -5.08 | 1.20E-21 |  |  | chemotaxis transducer |
| PA14_48030 |  | | -8.78 | 1.70E-11 |  |  | methyl-accepting chemotaxis transducer |
| PA14_55750 |  | | -3.56 | 1.06E-15 | 4.18 | 7.40E-24 | chemotaxis transducer |
| PA14_55960* | *pctC* | |  |  | -3.21 | 4.47E-07 | chemotactic transducer PctC |
| PA14_56000 | *pctA* | | -3.46 | 3.43E-14 |  |  | chemotactic transducer PctA |
| PA14_64060 | *ctpL* | |  |  | 3.74 | 2.93E-18 | chemotaxis transducer |
| PA14_64920 |  | | -39.81 | 8.78E-99 | -24.11 | 3.15E-91 | methyl-accepting chemotaxis protein |
| PA14_67010 |  | | 4.62 | 2.17E-19 | 2.99 | 2.33E-11 | chemotaxis transducer |
| **DNA replication, recombination, modification and repair** | | | | | | | |
| PA14_00110 | *tag* | |  |  | 2.97 | 4.34E-10 | DNA-3-methyladenine glycosidase I |
| PA14_00230 | *smf* | | 3.63 | 4.89E-10 |  |  | Rossmann fold nucleotide-binding protein |
| PA14_00810 |  | | 3.32 | 1.44E-13 |  |  | DNA repair photolyase |
| PA14_04670 | *mutM* | | 3.63 | 1.14E-15 |  |  | formamidopyrimidine-DNA glycosylase |
| PA14_05000* | *trmB* | | 11.46 | 1.35E-40 | 4.49 | 1.43E-15 | tRNA (guanine-N(7)-)-methyltransferase |
| PA14_07530 | *dnaG* | | 4.17 | 1.78E-20 | 3.31 | 2.43E-17 | DNA primase |
| PA14_09180* | *uvrA* | | 4.12 | 1.75E-19 |  |  | excinuclease ABC subunit A |
| PA14_09200 | *ssb* | | 8.19 | 8.96E-40 | 4.71 | 4.44E-26 | single-stranded DNA-binding protein |
| PA14_11570 | *xseB* | | 4.61 | 1.84E-18 | 2.87 | 5.48E-10 | exodeoxyribonuclease VII small subunit |
| PA14_12200 | *holA* | | 4.09 | 2.84E-19 |  |  | DNA polymerase III subunit delta |
| PA14_12670* |  | | 2.84 | 2.31E-10 |  |  | hypothetical protein |
| PA14_15230 | *xseA* | | 3.71 | 1.13E-15 |  |  | exodeoxyribonuclease VII large subunit |
| PA14_16040 | *xerD* | | 4.00 | 4.83E-16 | 2.95 | 3.09E-11 | site-specific tyrosine recombinase XerD |
| PA14_16220 | *recJ* | | 6.60 | 7.41E-31 |  |  | single-stranded-DNA-specific exonuclease RecJ |
| PA14_17230 | *rnhB* | | 5.38 | 2.57E-25 | 3.48 | 2.06E-16 | ribonuclease HII |
| PA14_17260* | *dnaE* | | 3.40 | 1.02E-15 |  |  | DNA polymerase III subunit alpha |
| PA14_17500 | *mutS* | | 6.73 | 1.69E-32 | 3.89 | 8.69E-20 | DNA mismatch repair protein MutS |
| PA14_18700 | *rnt* | | 2.68 | 6.62E-10 |  |  | ribonuclease T |
| PA14_19290 | *srmB* | | 4.24 | 1.39E-19 |  |  | ATP-dependent RNA helicase |
| PA14_20810* | *recQ* | | 4.99 | 7.37E-25 |  |  | ATP-dependent DNA helicase RecQ |
| PA14_21790* | *rdgC* | | 6.07 | 1.48E-28 |  |  | recombination associated protein |
| PA14_22380 |  | | -3.78 | 3.86E-14 |  |  | DNA polymerase III subunit epsilon |
| PA14_23100 | *mutT* | | -2.78 | 1.64E-08 |  |  | hypothetical protein |
| PA14_23260* | *gyrA* | | 3.25 | 1.25E-14 |  |  | DNA gyrase subunit A |
| PA14_23480 |  | | -3.93 | 1.12E-15 | -5.20 | 4.14E-24 | hypothetical protein |
| PA14_25110* | *topA* | | 3.26 | 7.01E-15 |  |  | DNA topoisomerase I |
| PA14_25160 | *lexA* | |  |  | 3.71 | 5.90E-20 | LexA repressor |
| PA14_25230* | *mfd* | |  |  | 2.77 | 1.94E-10 | transcription-repair coupling factor |
| PA14_28720 | *ihfA* | | -3.14 | 3.34E-13 |  |  | integration host factor subunit alpha |
| PA14_28840 |  | | -2.84 | 3.28E-11 | -2.64 | 2.47E-12 | helicase |
| PA14_30160 | *mutT* | | 6.80 | 1.16E-26 | 7.96 | 1.49E-33 | hypothetical protein |
| PA14_31650* | *xthA* | | 2.67 | 2.00E-09 |  |  | exonuclease III |
| PA14_35570 | *pslN* | | -7.99 | 4.74E-33 | -10.89 | 2.00E-44 | hypothetical protein |
| PA14_36760 |  | | -10.46 | 4.97E-37 | -10.23 | 6.06E-38 | KU domain-containing protein |
| PA14_41210^‡^ | *hupB* | |  |  | -3.12 | 4.15E-12 | DNA-binding protein HU |
| PA14_44130 |  | | -2.53 | 1.81E-08 |  |  | hypothetical protein |
| PA14_44610 | *recR* | | 6.12 | 1.61E-27 | 5.80 | 7.27E-28 | recombination protein RecR |
| PA14_44630 | *dnaX* | | 3.84 | 3.06E-18 | 2.65 | 4.82E-12 | DNA polymerase III subunits gamma and tau |
| PA14_44660* | *ligA* | |  |  | -3.06 | 1.63E-13 | NAD-dependent DNA ligase LigA |
| PA14_51790 | *ruvA* | | 2.62 | 3.14E-10 | 2.77 | 3.79E-13 | Holliday junction DNA helicase RuvA |
| PA14_51800 | *ruvC* | | 3.71 | 6.52E-17 |  |  | Holliday junction resolvase |
| PA14_55660 | *recD* | | -3.82 | 3.34E-17 | -8.93 | 3.07E-48 | exodeoxyribonuclease V subunit alpha |
| PA14_55670 | *recB* | | -2.50 | 4.24E-09 | -5.03 | 1.65E-29 | exodeoxyribonuclease V subunit beta |
| PA14_56080* | *sbcB* | | 2.74 | 2.09E-10 | 2.69 | 1.07E-11 | exonuclease I |
| PA14_58070 |  | | -2.50 | 2.06E-09 | -9.29 | 5.58E-52 | hypothetical protein |
| PA14_59150 |  | | -5.99 | 6.34E-08 | -8.17 | 2.56E-11 | single-stranded DNA-binding protein |
| PA14_61640 | *phr* | | 2.69 | 6.60E-11 | -4.73 | 9.42E-27 | deoxyribodipyrimidine photolyase |
| PA14_65130 | *dnaB* | | 3.51 | 1.09E-15 | 3.65 | 4.44E-19 | replicative DNA helicase |
| PA14_65660* | *parE* | | 5.13 | 7.67E-26 |  |  | DNA topoisomerase IV subunit B |
| PA14_66700 |  | |  |  | -2.55 | 1.08E-04 | nuclease |
| PA14_66720 | *priA* | | 4.01 | 5.08E-18 |  |  | primosome assembly protein PriA |
| PA14_67990 | *mutY* | | 3.45 | 6.86E-16 |  |  | A/G-specific adenine glycosylase |
| PA14_69710 | *xerC* | | 2.57 | 1.94E-09 |  |  | site-specific tyrosine recombinase XerC |
| PA14_69910* | *rep* | | 19.57 | 6.50E-70 | 6.45 | 1.59E-33 | ATP-dependent DNA helicase Rep |
| PA14_70570* | *recG* | | 3.43 | 1.61E-15 |  |  | ATP-dependent DNA helicase RecG |
| PA14_72490 | *polA* | | 3.34 | 7.17E-15 |  |  | DNA polymerase I |
| **Energy metabolism** | | |  |  |  |  |  |
| PA14_01290^¶^ | *coxB* | | -8.22 | 2.90E-37 |  |  | cytochrome c oxidase subunit II |
| PA14_01300^¶^ | *coxA* | | -8.11 | 4.36E-29 | -3.58 | 7.57E-17 | cytochrome c oxidase subunit I |
| PA14_01320 | *coIII* | | -15.59 | 2.23E-11 | -8.82 | 8.24E-10 | cytochrome c oxidase subunit III |
| PA14_01360 | *ctaA* | | -5.20 | 1.82E-17 | -10.71 | 2.24E-29 | hypothetical protein |
| PA14_02450* |  | |  |  | -5.43 | 2.44E-29 | NAD(P) transhydrogenase subunit alpha part 1 |
| PA14_02460 |  | |  |  | -2.56 | 2.86E-07 | NAD(P) transhydrogenase subunit alpha part 2 |
| PA14_02470 | *pntB* | |  |  | -4.85 | 5.43E-25 | pyridine nucleotide transhydrogenase subunit beta |
| PA14_04140* |  | | 4.54 | 2.29E-22 |  |  | hypothetical protein |
| PA14_04310 | *rpiA* | | 4.29 | 3.97E-19 | 2.50 | 1.04E-06 | ribose-5-phosphate isomerase A |
| PA14_04810 |  | | -2.55 | 1.81E-09 |  |  | aldehyde dehydrogenase |
| PA14_05040 |  | | 6.82 | 5.30E-28 |  |  | coproporphyrinogen III oxidase |
| PA14_05500 |  | | 2.81 | 5.67E-11 | 2.98 | 5.73E-14 | hypothetical protein |
| PA14_05820 |  | | -9.73 | 4.62E-06 |  |  | hypothetical protein |
| PA14_06650 | *nirN* | | 101.66 | 8.75E-111 | 59.62 | 2.97E-74 | c-type cytochrome |
| PA14_06660* | *nirE* | | 112.02 | 2.23E-62 | 44.61 | 3.23E-36 | uroporphyrin-III c-methyltransferase |
| PA14_06670* | *nirJ* | | 76.42 | 2.56E-37 | 29.80 | 2.71E-28 | heme d1 biosynthesis protein NirJ |
| PA14_06680* | *nirH* | | 77.23 | 5.54E-35 | 31.46 | 4.49E-32 | hypothetical protein |
| PA14_06700^‡^* | *nirL* | | 151.28 | 5.55E-29 | 29.56 | 1.09E-31 | heme d1 biosynthesis protein NirL |
| PA14_06710* | *nirD* | | 98.84 | 6.34E-42 | 28.15 | 6.65E-33 | transcriptional regulator |
| PA14_06720^‡^ | *nirF* | | 125.42 | 2.71E-90 | 23.25 | 2.48E-37 | heme d1 biosynthesis protein NirF |
| PA14_06730 | *nirC* | | 254.33 | 5.96E-106 | 23.46 | 5.15E-57 | c-type cytochrome |
| PA14_06740^‡^ | *nirM* | | 235.59 | 1.63E-164 | 22.93 | 3.96E-68 | cytochrome c-551 |
| PA14_06750^‡^ | *nirS* | | 94.35 | 3.46E-113 | 16.71 | 6.65E-74 | nitrite reductase |
| PA14_06770^‡^* | *nirQ* | | 5.55 | 9.95E-29 |  |  | regulatory protein NirQ |
| PA14_06790 | *nirO* | | 55.93 | 1.61E-106 | 5.52 | 4.15E-11 | cytochrome c oxidase subunit |
| PA14_06810^‡^ | *norC* | | 79.36 | 1.90E-135 | 15.43 | 2.35E-72 | nitric-oxide reductase subunit C |
| PA14_06830^‡^ | *norB* | | 298.74 | 1.22E-192 | 26.98 | 1.14E-34 | nitric-oxide reductase subunit B |
| PA14_06840^‡^ | *norD* | | 404.28 | 9.69E-201 | 85.17 | 6.61E-29 | dinitrification protein NorD |
| PA14_06960* |  | | -14.62 | 7.15E-58 | 4.26 | 9.64E-25 | hypothetical protein |
| PA14_07030 |  | |  |  | 2.67 | 2.01E-10 | cytochrome c' |
| PA14_10500 | *ccoN* | | -2.53 | 1.67E-07 |  |  | cbb3-type cytochrome c oxidase subunit I |
| PA14_11340 | *trx1* | | 2.88 | 9.81E-12 |  |  | thioredoxin |
| PA14_11690* | *ppa* | | 10.27 | 5.85E-44 | 4.80 | 4.17E-23 | inorganic pyrophosphatase |
| PA14_13030 | *cioA* | | -3.80 | 2.79E-17 |  |  | CioA, cyanide insensitive terminal oxidase |
| PA14_13040 | *cioB* | | -7.14 | 6.24E-34 | -4.17 | 9.15E-24 | CioB, cyanide insensitive terminal oxidase |
| PA14_13500 |  | | 2.92 | 9.12E-10 |  |  | 2-hydroxyacid dehydrogenase |
| PA14_13780^‡^* | *narG* | | 80.67 | 3.88E-18 | 5.37 | 2.67E-29 | ^respiratory nitrate reductase alpha subun^ |
| PA14_13800^‡^* | *narH* | | 63.96 | 1.79E-27 | 7.53 | 5.45E-19 | respiratory nitrate reductase beta subuni |
| PA14_13810^‡^* | *narJ* | | 91.11 | 1.28E-17 | 10.59 | 4.74E-14 | respiratory nitrate reductase delta chain |
| PA14_13830^‡^ | *narI* | | 111.83 | 3.17E-14 | 10.86 | 2.00E-07 | respiratory nitrate reductase gamma chain |
| PA14_14990 | *mocA* | |  |  | -2.54 | 3.35E-05 | oxidoreductase |
| PA14_16260 |  | | -43.87 | 2.21E-104 | -29.49 | 1.41E-101 | FMN oxidoreductase |
| PA14_16690* | *ppc* | |  |  | -3.32 | 1.06E-12 | phosphoenolpyruvate carboxylase |
| PA14_17490* | *fdxA* | |  |  | 3.91 | 3.20E-21 | ferredoxin I |
| PA14_17930 | *glpD* | | 6.57 | 9.37E-09 | 3.87 | 1.64E-21 | glycerol-3-phosphate dehydrogenase |
| PA14_18060 |  | | 6.49 | 2.17E-15 | 21.82 | 9.49E-51 | hypothetical protein |
| PA14_18750 | *gloA1* | | 2.68 | 3.39E-10 |  |  | lactoylglutathione lyase |
| PA14_18910 | *rnfD* | | 3.06 | 4.09E-11 |  |  | hypothetical protein |
| PA14_18920 | *rnfC* | | 3.13 | 2.78E-13 |  |  | electron transport complex protein RnfC |
| PA14_18930 | *rnfB* | | 3.54 | 1.98E-13 |  |  | electron transport complex protein RnfB |
| PA14_18950 | *rnfA* | | 4.42 | 1.44E-18 | 2.75 | 5.19E-10 | Na(+)-translocating NADH-quinone reductase subunit E |
| PA14_19660 | *mioC* | | 2.59 | 7.48E-09 |  |  | flavodoxin |
| PA14_19900 |  | | -33.31 | 2.66E-90 | -20.97 | 1.70E-83 | pyruvate dehydrogenase E1 component subunit alpha |
| PA14_19910* | *pdhB* | | -26.99 | 8.84E-82 | -26.23 | 3.06E-92 | pyruvate dehydrogenase E1 component, beta chain |
| PA14_19920 |  | | -17.65 | 1.62E-64 | -22.21 | 1.09E-82 | branched-chain alpha-keto acid dehydrogenase subunit E2 |
| PA14_20150 | *nosL* | | 42.18 | 3.89E-22 | 32.77 | 4.34E-51 | NosL protein |
| PA14_20170 | *nosY* | | 175.95 | 2.94E-30 | 84.02 | 2.38E-24 | NosY protein |
| PA14_20180* | *nosF* | | 103.90 | 7.13E-57 | 45.09 | 2.07E-29 | NosF protein |
| PA14_20200^‡^* | *nosZ* | | 133.18 | 1.54E-149 | 47.24 | 5.88E-112 | nitrous-oxide reductase |
| PA14_21890 | *qor* | | -3.39 | 5.59E-15 | -2.63 | 3.20E-12 | oxidoreductase |
| PA14_22910* | *edd* | | 6.39 | 1.38E-32 | -3.20 | 1.35E-05 | phosphogluconate dehydratase |
| PA14_24860 | *snr1* | | -5.02 | 5.63E-23 | -3.63 | 1.93E-18 | cytochrome c Snr1 |
| PA14_25280* | *nqrA* | | 3.62 | 1.40E-16 |  |  | Na(+)-translocating NADH-quinone reductase subunit A |
| PA14_25305 | *nqrB* | | 3.99 | 2.46E-16 |  |  | Na(+)-translocating NADH-quinone reductase subunit B |
| PA14_25840* |  | | 5.38 | 1.59E-27 |  |  | electron transfer flavoprotein-ubiquinone oxidoreductase |
| PA14_26070 |  | | 7.07 | 1.12E-02 | 4.41 | 1.17E-10 | hypothetical protein |
| PA14_28180* | *ordL* | | 3.87 | 1.61E-17 | 6.10 | 7.71E-34 | hypothetical protein |
| PA14_29040 |  | |  |  | -3.09 | 3.06E-03 | ferredoxin |
| PA14_29050 |  | |  |  | 2.85 | 5.77E-03 | molybdopterin oxidoreductase |
| PA14_29640*^‡^ | *fhp* | | 8.74 | 4.73E-43 | 3.70 | 3.52E-20 | nitric oxide dioxygenase |
| PA14_30020^¶^ | *nuoA* | |  |  | 3.27 | 2.65E-17 | NADH dehydrogenase subunit A |
| PA14_30040 |  | |  |  | 2.61 | 3.32E-11 | hypothetical protein |
| PA14_30050 | *aceA* | |  |  | 6.95 | 2.23E-41 | isocitrate lyase |
| PA14_30180^¶^* | *idh* | |  |  | 6.42 | 3.32E-31 | monomeric isocitrate dehydrogenase |
| PA14_30190^¶‡^* | *icd* | | -4.60 | 1.33E-22 |  |  | isocitrate dehydrogenase |
| PA14_30390 |  | | -3.55 | 4.85E-15 | -2.79 | 1.37E-12 | sulfur relay protein TusC |
| PA14_30400 | *dsrE* | | -3.25 | 3.02E-13 |  |  | sulfur transfer complex subunit TusD |
| PA14_30460 |  | |  |  | 15.08 | 1.29E-59 | flavin-dependent oxidoreductase |
| PA14_30490 |  | |  |  | 7.45 | 4.63E-31 | lavin-dependent oxidoreductase |
| PA14_31350 |  | | -56.61 | 6.64E-112 | -2.71 | 6.96E-13 | hypothetical protein |
| PA14_31540* | *acdA* | | -8.57 | 1.72E-40 | -48.80 | 1.31E-122 | acyl-CoA dehydrogenase |
| PA14_32520 |  | |  |  | 2.55 | 2.76E-11 | hypothetical protein |
| PA14_32530 |  | | 3.01 | 1.33E-12 | -2.71 | 2.02E-11 | cytochrome c |
| PA14_34250 |  | | 3.87 | 1.13E-09 | 3.45 | 3.76E-08 | glycerophosphoryl diester phosphodiesterase |
| PA14_34920 |  | | 2.57 | 2.22E-06 |  |  | ferredoxin |
| PA14_34930 |  | | 4.88 | 2.62E-16 |  |  | phycobiliprotein |
| PA14_35270 |  | |  |  | -6.30 | 1.84E-31 | cytochrome c precursor |
| PA14_35320 | *kguD* | | -5.83 | 1.85E-26 | -24.73 | 7.05E-75 | 2-hydroxyacid dehydrogenase |
| PA14_35490* | *lpdV* | | -8.87 | 8.58E-42 | -75.42 | 1.32E-143 | dihydrolipoamide dehydrogenase |
| PA14_36710 | *glgB* | | -10.88 | 9.22E-45 | -19.17 | 2.35E-69 | glycogen branching protein |
| PA14_37470 |  | | -6.76 | 3.38E-22 | 8.68 | 1.09E-44 | flavin-dependent oxidoreductase |
| PA14_38750 |  | |  |  | 4.46 | 2.41E-24 | iron-containing alcohol dehydrogenase |
| PA14_38840* | *exaC* | | -8.60 | 3.01E-18 |  |  | NAD+ dependent acetaldehyde dehydrogenase |
| PA14_39520 |  | | -3.11 | 2.08E-11 | -2.63 | 5.34E-10 | hydroxylase large subunit |
| PA14_39530 |  | | -3.23 | 1.71E-10 | -2.80 | 4.49E-09 | hydroxylase molybdopterin-containing subunit |
| PA14_39540 |  | | -20.09 | 4.47E-63 | -8.83 | 1.39E-42 | ferredoxin |
| PA14_40510 | *ccoN2* | | 11.51 | 1.41E-06 | 9.01 | 6.64E-45 | cbb3-type cytochrome c oxidase subunit I |
| PA14_40830 | *yhfP* | | -2.64 | 4.80E-10 | -6.74 | 3.72E-39 | oxidoreductase |
| PA14_41470^‡^* | *acnB* | | 4.89 | 3.94E-25 | 4.72 | 5.84E-28 | bifunctional aconitate hydratase 2/2-methylisocitrate dehydratase |
| PA14_43780 |  | | 3.31 | 7.24E-09 | 7.00 | 1.35E-29 | oxidoreductase |
| PA14_43810 |  | | 11.37 | 1.67E-41 |  |  | cytochrome c |
| PA14_43940* | *sucD* | |  |  | -2.62 | 2.09E-12 | succinyl-CoA synthetase subunit alpha |
| PA14_43970^¶^* | *lpdG* | | -2.56 | 1.52E-09 | -3.94 | 9.85E-23 | dihydrolipoamide dehydrogenase |
| PA14_44020* | *sdhB* | | 3.66 | 9.21E-18 | 5.37 | 4.38E-32 | succinate dehydrogenase iron-sulfur subunit |
| PA14_44030^‡^* | *sdhA* | | 8.41 | 4.73E-42 | 6.29 | 5.95E-32 | succinate dehydrogenase flavoprotein subunit |
| PA14_44050 | *sdhD* | | 21.75 | 8.89E-43 | 13.54 | 2.96E-60 | succinate dehydrogenase (D subunit) |
| PA14_44060 | *sdhC* | | 21.24 | 8.27E-75 | 19.67 | 2.57E-79 | succinate dehydrogenase, cytochrome b556 subunit |
| PA14_44070* | *gltA* | |  |  | 3.66 | 1.14E-20 | type II citrate synthase |
| PA14_44290^‡^* | *acnA* | |  |  | -6.20 | 1.58E-37 | aconitate hydratase |
| PA14_44340 | *ccoN* | |  |  | -5.28 | 2.08E-15 | cbb3-type cytochrome c oxidase subunit I |
| PA14_44350 | *ccoO* | |  |  | -9.29 | 1.76E-48 | cbb3-type cytochrome c oxidase subunit II |
| PA14_44360 | *ccoP* | |  |  | -7.32 | 1.08E-40 | cytochrome c oxidase, cbb3-type subunit III |
| PA14_44370 | *ccoN* | | 4.66 | 1.22E-22 | 2.88 | 2.82E-13 | cbb3-type cytochrome c oxidase subunit I |
| PA14_44380 | *ccoO* | | 3.84 | 1.27E-17 | 3.14 | 2.40E-13 | cbb3-type cytochrome c oxidase subunit II |
| PA14_44390 |  | | 3.13 | 1.98E-10 | 2.73 | 4.34E-11 | cytochrome c oxidase subunit |
| PA14_44400 | *ccoP* | | 3.28 | 1.57E-14 |  |  | cytochrome c oxidase, cbb3-type subunit III |
| PA14_45050 | *pykF* | | -3.15 | 6.63E-09 |  |  | pyruvate kinase |
| PA14_45310 | *ccmF* | | 3.74 | 7.70E-18 | 2.58 | 4.47E-06 | cytochrome C-type biogenesis protein CcmF |
| PA14_47150 | *cyoE* | |  |  | -5.45 | 4.79E-10 | protoheme IX farnesyltransferase |
| PA14_47160^‡^ | *cyoD* | |  |  | -7.02 | 3.93E-09 | cytochrome o ubiquinol oxidase subunit IV |
| PA14_47180 | *cyoC* | |  |  | -6.24 | 3.13E-19 | cytochrome o ubiquinol oxidase subunit III |
| PA14_47190^‡^ | *cyoB* | |  |  | -6.72 | 2.72E-36 | cytochrome o ubiquinol oxidase subunit I |
| PA14_47210^‡^ | *cyoA* | |  |  | -5.43 | 1.43E-13 | cytochrome o ubiquinol oxidase subunit II |
| PA14_49210 | *napE* | | -16.76 | 5.56E-62 | -11.55 | 4.35E-57 | periplasmic nitrate reductase NapE |
| PA14_49220 | *napF* | | -10.58 | 1.32E-45 | -13.14 | 2.36E-60 | ferredoxin protein NapF |
| PA14_49230 | *napD* | | -8.13 | 1.18E-14 | -12.29 | 1.05E-20 | NapD protein of periplasmic nitrate reductase |
| PA14_49250 | *napA* | | -3.72 | 1.23E-16 | -8.14 | 4.22E-45 | nitrate reductase catalytic subunit |
| PA14_49260 | *napB* | |  |  | -6.29 | 5.50E-24 | cytochrome c-type protein NapB precursor |
| PA14_49270 | *napc* | |  |  | -4.11 | 3.15E-16 | cytochrome c-type protein NapC |
| PA14_53970* |  | | -4.50 | 2.36E-21 |  |  | aconitate hydratase |
| PA14_56300* | *fumA* | | 9.48 | 1.33E-28 | 6.04 | 9.97E-33 | fumarase |
| PA14_56540* |  | |  |  | -13.07 | 1.22E-09 | hypothetical protein |
| PA14_57540* |  | | 6.55 | 1.77E-33 | 4.26 | 8.12E-14 | cytochrome c1 |
| PA14_57560 |  | | 10.14 | 2.79E-47 | 5.37 | 2.51E-30 | cytochrome b |
| PA14_57650 |  | | 18.81 | 1.11E-63 | 8.28 | 1.84E-37 | hypothetical protein |
| PA14_58030 | *fumC* | | -35.58 | 1.17E-34 | -17.25 | 6.44E-29 | fumarate hydratase |
| PA14_60490 |  | | 7.94 | 7.95E-10 | -3.79 | 5.54E-09 | cytochrome c |
| PA14_60700^¶^ | *ccpR* | |  |  | -13.24 | 3.96E-17 | cytochrome c551 peroxidase |
| PA14_61060 | *fnr2* | |  |  | -2.71 | 1.75E-12 | oxidoreductase |
| PA14_61140 |  | | 7.65 | 1.26E-34 |  |  | hypothetical protein |
| PA14_63090 | *lldD* | | 4.17 | 5.81E-15 | 4.86 | 3.09E-27 | L-lactate dehydrogenase |
| PA14_63100 |  | | 2.98 | 6.15E-13 | 3.65 | 2.59E-20 | ferredoxin |
| PA14_63550 | *fdhE* | | 10.92 | 2.35E-40 |  |  | formate dehydrogenase accessory protein FdhE |
| PA14_63570 | *fdnI* | | 14.89 | 5.66E-48 | 3.29 | 2.06E-09 | nitrate-inducible formate dehydrogenase subunit gamma |
| PA14_63580* | *fdnH* | | 11.95 | 4.54E-17 | 3.40 | 6.97E-14 | nitrate-inducible formate dehydrogenase subunit beta |
| PA14_63605* | *fdnG* | | 2.60 | 2.20E-10 |  |  | formate dehydrogenase-O, major subunit |
| PA14_63850 | *lpd3* | | -3.26 | 1.82E-12 | 3.27 | 3.06E-16 | dihydrolipoamide dehydrogenase |
| PA14_64620 |  | | 22.39 | 3.01E-44 | 7.18 | 2.49E-16 | oxidoreductase |
| PA14_65000^¶^* | *azu* | |  |  | -3.70 | 6.84E-21 | azurin |
| PA14_65080 | *ygiR* | | 4.44 | 8.56E-21 | 6.67 | 1.22E-36 | hypothetical protein |
| PA14_65760 |  | |  |  | -3.79 | 2.08E-16 | NAD(P)H quinone oxidoreductase |
| PA14_66000* |  | | 6.92 | 1.10E-31 |  |  | hypothetical protein |
| PA14_66010 |  | | 4.21 | 4.96E-18 |  |  | hypothetical protein |
| PA14_66310 | *aceF* | | 3.03 | 1.01E-13 | -4.58 | 4.10E-11 | dihydrolipoamide acetyltransferase |
| PA14_68440 |  | | -3.28 | 7.07E-11 |  |  | oxidoreductase |
| PA14_68580* | *pckA* | | 8.55 | 2.06E-41 | 5.35 | 1.86E-30 | phosphoenolpyruvate carboxykinase |
| PA14_69110 |  | |  |  | -3.41 | 1.79E-03 | oxidoreductase |
| PA14_69140 | *ubiB* | | 3.37 | 3.19E-15 |  |  | CDP-6-deoxy-delta-3,4-glucoseen reductase |
| PA14_69950 |  | |  |  | -4.64 | 1.74E-09 | hypothetical protein |
| PA14_69970* | *cycB* | | 4.24 | 1.09E-20 |  |  | cytochrome c5 |
| PA14_70340 |  | |  |  | 4.07 | 1.37E-03 | cytochrome c(mono-heme type) |
| PA14_70670 | *glcF* | |  |  | -3.00 | 3.76E-05 | glycolate oxidase iron-sulfur subunit |
| PA14_71300 | *etfA1* | |  |  | -4.36 | 6.67E-05 | electron transfer flavoprotein alpha subunit |
| PA14_71310 | *etfB1* | |  |  | -3.06 | 2.43E-04 | hypothetical protein |
| PA14_71800 | *zwf* | | -5.39 | 1.31E-25 | -5.96 | 1.28E-34 | glucose-6-phosphate 1-dehydrogenase |
| PA14_72470 |  | | 7.17 | 2.22E-34 | 5.47 | 7.59E-30 | cytochrome |
| PA14_73250 | *atpG* | | 2.90 | 1.13E-12 |  |  | F0F1 ATP synthase subunit gamma |
| PA14_73260^‡^* | *atpA* | | 3.03 | 1.50E-13 |  |  | F0F1 ATP synthase subunit alpha |
| PA14_73280* | *atpH* | | 4.45 | 1.01E-16 | 2.87 | 1.38E-14 | F0F1 ATP synthase subunit delta |
| PA14_73290 | *atpF* | | 4.73 | 2.29E-16 | 3.19 | 3.80E-17 | F0F1 ATP synthase subunit B |
| PA14_73300 | *atpE* | | 6.30 | 8.06E-22 | 3.84 | 8.42E-14 | F0F1 ATP synthase subunit C |
| PA14_73310 | *atpB* | | 9.95 | 2.70E-43 | 4.51 | 5.46E-26 | F0F1 ATP synthase subunit A |
| PA14_73320 | *atpI* | | 9.51 | 2.56E-44 | 5.51 | 2.99E-30 | F0F1 ATP synthase subunit I |
| **Fatty acid and phospholipid metabolism** | | | |  |  |  |  |
| PA14_00580 |  | | 9.69 | 1.40E-11 |  |  | lipoprotein |
| PA14_00590 |  | | 8.41 | 7.33E-12 |  |  | lipoprotein |
| PA14_00960 |  | | 2.75 | 4.74E-10 |  |  | lipoprotein |
| PA14_03730* |  | |  |  | 8.32 | 2.33E-47 | fatty acid desaturase |
| PA14_04460 | *lgt* | | 6.37 | 1.79E-26 |  |  | prolipoprotein diacylglyceryl transferase |
| PA14_04680 |  | | 7.05 | 8.30E-29 |  |  | hypothetical protein |
| PA14_05840* | *gcdH* | | -30.20 | 3.54E-14 |  |  | glutaryl-CoA dehydrogenase |
| PA14_06600* |  | |  |  | -6.20 | 1.32E-37 | acyl-CoA dehydrogenase |
| PA14_06620 |  | |  |  | -3.55 | 1.52E-03 | acyl-CoA dehydrogenase |
| PA14_06640 |  | | 2.61 | 9.32E-10 |  |  | acyl-CoA dehydrogenase |
| PA14_09660 |  | |  |  | 5.88 | 9.94E-32 | acyl-CoA synthetase |
| PA14_10370 | *choS* | | -11.48 | 1.31E-43 | -2.88 | 1.86E-12 | hypothetical protein |
| PA14_11280 |  | | -3.25 | 3.51E-06 |  |  | hypothetical protein |
| PA14_11470 | *pgpA* | | 5.77 | 2.86E-24 |  |  | phosphatidylglycerophosphatase A |
| PA14_12450 |  | |  |  | -6.88 | 1.64E-40 | acyl-CoA dehydrogenase |
| PA14_12680 |  | | -20.32 | 6.98E-70 | -11.19 | 2.93E-57 | short chain dehydrogenase |
| PA14_12870 | *tesB* | | 2.59 | 1.17E-09 |  |  | acyl-CoA thioesterase |
| PA14_13110 |  | | -11.07 | 1.33E-06 | -3.79 | 3.61E-04 | long-chain-fatty-acid--CoA ligase |
| PA14_13320 |  | | 41.14 | 1.56E-90 | 113.20 | 2.64E-18 | hypothetical protein |
| PA14_14060 |  | |  |  | -3.29 | 6.96E-16 | AMP-binding protein |
| PA14_16010 |  | | 8.40 | 1.11E-28 | 3.28 | 5.51E-09 | hypothetical protein |
| PA14_16640 |  | | -4.94 | 7.39E-24 | -23.88 | 5.53E-92 | lipoprotein |
| PA14_16840 |  | |  |  | -2.86 | 1.55E-12 | lipoprotein |
| PA14_16860* | *plsB* | | 4.04 | 2.18E-18 | 2.56 | 2.42E-10 | glycerol-3-phosphate acyltransferase |
| PA14_17120 | *cdsA* | | 7.34 | 6.47E-19 | 5.36 | 3.33E-08 | phosphatidate cytidylyltransferase |
| PA14_17190* | *fabZ* | | 5.48 | 7.83E-28 | 3.41 | 1.71E-17 | (3R)-hydroxymyristoyl-ACP dehydratase |
| PA14_17675 | *dgkA* | | 5.74 | 1.58E-20 | 5.53 | 7.03E-21 | diacylglycerol kinase |
| PA14_18150* | *acsL* | | -3.86 | 1.79E-14 |  |  | acetyl-coa synthetase |
| PA14_19740 |  | | -3.18 | 3.38E-11 | -3.10 | 1.27E-12 | enoyl-CoA hydratase |
| PA14_20940 |  | | -3.66 | 8.56E-16 | -5.18 | 9.34E-28 | acyl carrier protein |
| PA14_20950 | *fabH2* | | -3.02 | 3.86E-12 | -5.36 | 9.19E-30 | 3-oxoacyl-ACP synthase |
| PA14_22490 | *acpD* | |  |  | 4.62 | 1.93E-20 | ACP phosphodieterase |
| PA14_23860* | *accD* | | 2.94 | 2.03E-12 |  |  | acetyl-CoA carboxylase subunit beta |
| PA14_24970 | *yegS* | | -6.58 | 1.49E-18 | -5.94 | 3.10E-20 | lipid kinase |
| PA14_25080* | *fadB* | | 3.66 | 2.21E-13 |  |  | multifunctional fatty acid oxidation complex subunit alpha |
| PA14_25090* | *fadA* | | 2.66 | 2.54E-07 |  |  | 3-ketoacyl-CoA thiolase |
| PA14_25640 | *plsX* | | 14.69 | 5.15E-60 | 7.51 | 2.23E-40 | glycerol-3-phosphate acyltransferase PlsX |
| PA14_25650* | *fabD* | | 4.77 | 1.83E-13 |  |  | malonyl-CoA-ACP transacylase |
| PA14_25670 | *acpP* | | 3.01 | 2.38E-13 | 4.30 | 2.01E-25 | acyl carrier protein |
| PA14_25690* | *fabF1* | | 4.08 | 5.64E-20 |  |  | 3-oxoacyl-ACP synthase |
| PA14_26670 |  | |  |  | -8.81 | 2.74E-35 | biotin carboxylase |
| PA14_26690 |  | |  |  | -8.52 | 2.04E-27 | enoyl-CoA hydratase/isomerase |
| PA14_26700 |  | |  |  | -5.69 | 5.85E-26 | acyl-CoA dehydrogenase |
| PA14_26720 | *mccB* | |  |  | -6.46 | 1.18E-27 | biotin-dependent carboxylase |
| PA14_27360 |  | | -4.44 | 1.56E-11 |  |  | enoyl-CoA hydratase |
| PA14_27730* | *fadE* | | -4.70 | 1.04E-22 | -12.60 | 3.32E-64 | acyl-CoA dehydrogenase |
| PA14_28310 |  | |  |  | 6.43 | 4.16E-27 | enoyl-CoA hydratase |
| PA14_29290 |  | | 4.30 | 1.74E-14 | 3.01 | 7.63E-09 | hypothetical protein |
| PA14_29720 |  | | -5.51 | 5.55E-26 | -7.02 | 3.24E-39 | hypothetical protein |
| PA14_30670 | *pgsA* | | 5.77 | 8.66E-26 | 5.43 | 3.14E-17 | CDP-diacylglycerol--glycerol-3-phosphate 3-phosphatidyltransferase |
| PA14_31470 |  | |  |  | -2.97 | 3.07E-06 | AMP-binding protein |
| PA14_31500* |  | | -4.70 | 2.89E-23 | -6.70 | 2.91E-36 | AMP-binding protein |
| PA14_31530* |  | | -9.78 | 5.81E-45 | -53.21 | 1.19E-127 | acyl-CoA thiolase |
| PA14_31580 |  | | -2.95 | 3.43E-05 | -9.31 | 5.31E-13 | acyl-CoA dehydrogenase |
| PA14_31720* |  | | 4.13 | 1.25E-18 |  |  | hypothetical protein |
| PA14_31750 |  | | 2.68 | 5.78E-08 |  |  | acyltransferase |
| PA14_34490 |  | | -8.45 | 2.59E-37 |  |  | hypothetical protein |
| PA14_35950 |  | |  |  | -2.76 | 2.27E-04 | dehydrogenase |
| PA14_36690 | *ybhO* | | -6.78 | 4.14E-23 | -5.88 | 9.64E-21 | cardiolipin synthase 2 |
| PA14_38440* | *gnyD* | | -3.28 | 7.49E-07 | -4.66 | 1.68E-10 | citronelloyl-CoA dehydrogenase, GnyD |
| PA14_38610 |  | |  |  | 4.70 | 2.63E-26 | hypothetical protein |
| PA14_38630^‡^* | *atoB* | | -2.64 | 5.37E-10 |  |  | acetyl-CoA acetyltransferase |
| PA14_38660 | *scoA* | | -4.09 | 2.78E-02 |  |  | CoA transferase, subunit A |
| PA14_38690 | *acsA* | |  |  | -2.71 | 1.20E-12 | acetoacetyl-CoA synthetase |
| PA14_39060 |  | |  |  | 5.33 | 6.49E-06 | lipoprotein |
| PA14_39150 | *acpD* | | 4.79 | 8.64E-12 |  |  | azoreductase |
| PA14_40310 |  | |  |  | 6.24 | 3.73E-15 | acyl carrier protein |
| PA14_40640 | *cti* | | 2.56 | 8.69E-10 |  |  | cis/trans isomerase |
| PA14_40800 |  | | -2.56 | 4.10E-06 | 4.54 | 1.48E-23 | hypothetical protein |
| PA14_41170 | *fabI* | |  |  | -3.99 | 2.16E-20 | NADH-dependent enoyl-ACP reductase |
| PA14_41650 | *estX* | | 12.89 | 1.60E-44 | 3.03 | 6.71E-10 | esterase |
| PA14_42080 | *fadB* | |  |  | -4.56 | 6.22E-24 | 3-hydroxyacyl-CoA dehydrogenase |
| PA14_43290 |  | | -3.66 | 3.36E-14 | -6.39 | 1.71E-27 | lipoprotein |
| PA14_43420 |  | | -2.57 | 5.37E-09 |  |  | acyl-CoA dehydrogenase |
| PA14_43440 |  | |  |  | 3.44 | 7.55E-09 | enoyl-CoA hydratase |
| PA14_43680 | *fabA* | | 3.13 | 1.67E-13 |  |  | 3-hydroxydecanoyl-ACP dehydratase |
| PA14_43880 |  | | 4.55 | 2.02E-13 | 2.52 | 2.70E-05 | hypothetical protein |
| PA14_46490 | *fabF2* | |  |  | 7.75 | 7.44E-24 | 3-oxoacyl-ACP synthase |
| PA14_53910 | *pmtA* | | -5.90 | 1.37E-20 | -5.04 | 8.76E-20 | phospholipid methyltransferase |
| PA14_56200 |  | | -3.09 | 3.04E-12 |  |  | lipoprotein |
| PA14_56570 |  | | -5.65 | 7.62E-23 | -20.85 | 6.35E-55 | acyltransferase |
| PA14_57480 |  | | 2.56 | 8.38E-08 |  |  | lipoprotein |
| PA14_58210 |  | | 4.99 | 1.35E-18 | 4.29 | 1.93E-16 | hypothetical protein |
| PA14_60690 |  | |  |  | 5.49 | 2.18E-06 | lipoprotein |
| PA14_61290 |  | | 5.40 | 1.78E-06 | 2.65 | 1.05E-09 | lipoprotein |
| PA14_61360* |  | | 5.00 | 1.34E-21 | 3.21 | 5.34E-13 | hypothetical protein |
| PA14_62120 | *pssA* | | 4.32 | 4.13E-20 | 2.96 | 2.14E-13 | phosphatidylserine synthase |
| PA14_63250 | *yfcY* | | -2.89 | 1.26E-04 | -5.83 | 5.25E-09 | acetyl-CoA acetyltransferase |
| PA14_63290 | *phaJ3* | |  |  | -5.41 | 1.54E-05 | hypothetical protein |
| PA14_63330 |  | | -3.44 | 4.96E-13 | -3.65 | 1.01E-15 | glycerolphosphodiesterase |
| PA14_63340 |  | | -3.42 | 4.96E-10 |  |  | lipoprotein |
| PA14_64010 |  | |  |  | -3.02 | 1.65E-14 | hypothetical protein |
| PA14_64100^¶^ | *accB* | | 3.66 | 1.00E-16 |  |  | acetyl-CoA carboxylase biotin carboxyl carrier protein subunit |
| PA14_64110* | *accC* | | 3.56 | 1.76E-16 |  |  | acetyl-CoA carboxylase biotin carboxylase subunit |
| PA14_64610 |  | | 21.26 | 9.87E-33 | 9.37 | 4.60E-11 | hypothetical protein |
| PA14_64990 |  | | 6.79 | 5.17E-30 |  |  | hypothetical protein |
| PA14_65500* | *psd* | | 4.27 | 1.17E-19 |  |  | phosphatidylserine decarboxylase |
| PA14_65720 |  | |  |  | -2.76 | 1.07E-02 | lipoprotein |
| PA14_65840 |  | |  |  | 5.48 | 4.76E-19 | enoyl-CoA hydratase/isomerase |
| PA14_66050 |  | | 4.07 | 3.69E-17 |  |  | acyl-CoA dehydrogenase |
| PA14_66350 |  | |  |  | -4.09 | 8.47E-09 | acyl-CoA dehydrogenase |
| PA14_67380 |  | | -2.79 | 7.43E-09 |  |  | fatty acid desaturase |
| PA14_67510 | *estA* | |  |  | -4.97 | 1.43E-29 | esterase EstA |
| PA14_69660 | *lppL* | |  |  | 3.01 | 2.32E-14 | lipopeptide LppL |
| PA14_70060 |  | |  |  | -2.97 | 3.49E-14 | lipoprotein |
| PA14_70350 |  | | -4.22 | 2.17E-07 | -2.51 | 3.43E-05 | hypothetical protein |
| PA14_71890 | *psecoA* | |  |  | 17.20 | 4.20E-73 | coenzyme A transferase |
| PA14_72040 |  | | 5.10 | 1.08E-22 |  |  | acyltransferase |
| PA14_72600 |  | |  |  | 3.64 | 3.58E-18 | lipoprotein |
| PA14_72900 |  | | -5.35 | 3.85E-20 |  |  | lipoprotein |
| PA14_73140 |  | | -4.43 | 4.81E-21 | -4.77 | 2.56E-28 | hypothetical protein |
| **Hypothetical, unclassified, unknown** | | | |  |  |  |  |
| PA14_00080 |  | | -10.01 | 4.41E-45 | -7.30 | 5.05E-42 | hypothetical protein |
| PA14_00130 |  | |  |  | 5.04 | 1.04E-22 | hypothetical protein |
| PA14_00300 | *plcB* | |  |  | -4.67 | 7.47E-08 | phospholipase C, PlcB |
| PA14_00320 |  | |  |  | -12.34 | 1.19E-18 | hypothetical protein |
| PA14_00470 |  | | -8.72 | 1.31E-40 | -7.57 | 5.59E-44 | hypothetical protein |
| PA14_00480* |  | | -7.81 | 3.09E-37 | -10.00 | 6.03E-55 | hypothetical protein |
| PA14_00520 |  | | 3.70 | 1.23E-13 | 7.59 | 3.46E-12 | hypothetical protein |
| PA14_00530 |  | | 3.00 | 4.69E-11 |  |  | hypothetical protein |
| PA14_00630 |  | | -3.81 | 4.19E-09 | -6.77 | 1.50E-15 | hypothetical protein |
| PA14_00650 |  | | -65.56 | 4.51E-114 | -44.32 | 4.24E-109 | hypothetical protein |
| PA14_00720 |  | | -4.73 | 7.74E-22 | -8.44 | 1.07E-44 | hypothetical protein |
| PA14_00730 |  | |  |  | -2.62 | 1.58E-10 | hypothetical protein |
| PA14_00820* |  | |  |  | -5.76 | 2.68E-33 | hypothetical protein |
| PA14_00925 |  | | 3.34 | 7.65E-14 |  |  | hypothetical protein |
| PA14_00940 |  | | 4.60 | 2.44E-21 |  |  | hypothetical protein |
| PA14_00970 |  | |  |  | -2.63 | 3.81E-08 | hypothetical protein |
| PA14_00990 |  | | 3.16 | 5.22E-14 | 4.23 | 5.07E-24 | hypothetical protein |
| PA14_01010 |  | | 5.50 | 2.67E-13 | 3.99 | 2.16E-20 | hypothetical protein |
| PA14_01020* |  | | 4.35 | 1.13E-11 | 3.31 | 1.62E-17 | hypothetical protein |
| PA14_01060 |  | | 12.21 | 5.20E-33 | 5.05 | 7.94E-13 | hypothetical protein |
| PA14_01070 |  | | 9.29 | 7.10E-28 | 5.40 | 3.39E-26 | hypothetical protein |
| PA14_01080 |  | | 5.01 | 8.04E-04 | 3.92 | 1.71E-17 | hypothetical protein |
| PA14_01140 |  | | 4.28 | 6.90E-06 |  |  | hypothetical protein |
| PA14_01150 |  | | 3.09 | 1.36E-11 | 2.72 | 1.91E-10 | hypothetical protein |
| PA14_01330 |  | | -31.55 | 3.74E-44 | -11.49 | 8.00E-32 | hypothetical protein |
| PA14_01340 |  | | -4.98 | 1.74E-15 | -7.30 | 1.18E-22 | hypothetical protein |
| PA14_01350 |  | | -10.17 | 2.07E-36 | -14.46 | 3.79E-45 | hypothetical protein |
| PA14_01540* |  | | 3.63 | 1.14E-13 |  |  | hypothetical protein |
| PA14_01730 |  | |  |  | -20.49 | 5.40E-38 | hypothetical protein |
| PA14_02520 |  | | -11.90 | 2.88E-51 | -11.74 | 4.28E-61 | hypothetical protein |
| PA14_02890 |  | | 3.74 | 3.00E-09 |  |  | hypothetical protein |
| PA14_03090 |  | | -10.31 | 1.17E-15 | -4.78 | 2.43E-11 | hypothetical protein |
| PA14_03100 |  | | 6.17 | 1.35E-12 | 5.04 | 2.66E-12 | hypothetical protein |
| PA14_03110 |  | | 10.21 | 2.69E-15 | 6.36 | 7.07E-10 | hypothetical protein |
| PA14_03160 |  | | -4.38 | 1.87E-20 | -3.01 | 5.17E-15 | hypothetical protein |
| PA14_03163 |  | | -3.13 | 3.82E-04 |  |  | hypothetical protein |
| PA14_03330 |  | | 4.49 | 1.28E-19 |  |  | hypothetical protein |
| PA14_03340 |  | | 2.86 | 4.12E-11 |  |  | hypothetical protein |
| PA14_03350 |  | |  |  | -3.09 | 4.43E-09 | hypothetical protein |
| PA14_03360 |  | |  |  | -3.15 | 9.44E-08 | hypothetical protein |
| PA14_03370 |  | |  |  | -2.87 | 7.79E-13 | hypothetical protein |
| PA14_03400 |  | | 3.50 | 6.58E-08 | 16.32 | 3.79E-33 | hypothetical protein |
| PA14_03420 |  | |  |  | 4.81 | 8.83E-02 | hypothetical protein |
| PA14_03510 |  | | -7.92 | 5.34E-34 | -10.83 | 4.91E-45 | hypothetical protein |
| PA14_03520 |  | | -10.59 | 5.66E-25 | -13.36 | 7.33E-32 | hypothetical protein |
| PA14_03710 |  | |  |  | 189.58 | 2.24E-68 | hypothetical protein |
| PA14_04010 |  | | -2.64 | 6.68E-10 | -7.35 | 2.00E-41 | hypothetical protein |
| PA14_04020 |  | | 3.11 | 6.30E-11 |  |  | hypothetical protein |
| PA14_04100 |  | | -9.32 | 6.36E-43 | -7.65 | 1.90E-44 | hypothetical protein |
| PA14_04180 |  | | 6.69 | 4.19E-23 |  |  | hypothetical protein |
| PA14_04300 |  | | -3.68 | 3.10E-07 | -6.97 | 2.29E-13 | hypothetical protein |
| PA14_04510 |  | |  |  | -2.82 | 2.64E-08 | hypothetical protein |
| PA14_04710 |  | | 4.93 | 1.20E-17 | 3.77 | 6.57E-13 | hypothetical protein |
| PA14_04850 |  | | 3.52 | 4.06E-13 |  |  | hypothetical protein |
| PA14_04940 |  | | 4.02 | 1.56E-18 |  |  | hypothetical protein |
| PA14_05020 |  | | -3.22 | 1.34E-11 | -3.29 | 4.81E-13 | hypothetical protein |
| PA14_05060 |  | | -3.99 | 1.62E-18 | -4.42 | 8.55E-26 | hypothetical protein |
| PA14_05120 |  | | 2.98 | 1.78E-10 |  |  | hypothetical protein |
| PA14_05450 |  | | 5.25 | 1.56E-20 |  |  | 16S ribosomal RNA methyltransferase RsmE |
| PA14_05580 |  | |  |  | 2.56 | 6.65E-12 | hypothetical protein |
| PA14_05775 |  | |  |  | 4.34 | 5.89E-03 | hypothetical protein |
| PA14_05970 |  | | 5.97 | 4.60E-23 | 4.07 | 9.97E-11 | hypothetical protein |
| PA14_06010 |  | |  |  | -9.05 | 2.88E-50 | hypothetical protein |
| PA14_06280 |  | |  |  | -2.58 | 2.22E-07 | hypothetical protein |
| PA14_06580 |  | |  |  | 6.16 | 3.07E-34 | hypothetical protein |
| PA14_06800 |  | | 198.97 | 3.39E-125 | 10.60 | 8.62E-08 | hypothetical protein |
| PA14_06860 |  | | 3.66 | 4.26E-17 | 7.20 | 6.92E-41 | hypothetical protein |
| PA14_07200 |  | | -3.68 | 2.46E-16 | -6.05 | 1.23E-34 | hypothetical protein |
| PA14_07210 |  | |  |  | -4.39 | 1.87E-24 | hypothetical protein |
| PA14_07240 |  | | 3.52 | 1.56E-14 |  |  | hypothetical protein |
| PA14_07330 |  | | 5.49 | 1.10E-20 |  |  | hypothetical protein |
| PA14_07355 |  | | -10.63 | 2.73E-42 | -5.67 | 6.31E-29 | hypothetical protein |
| PA14_07370 |  | |  |  | -5.22 | 5.59E-26 | hypothetical protein |
| PA14_07430 |  | | -9.96 | 2.88E-45 | -64.12 | 1.51E-134 | hypothetical protein |
| PA14_07440 |  | | 2.66 | 1.20E-05 |  |  | hypothetical protein |
| PA14_07450 |  | | 3.17 | 4.02E-12 | 2.51 | 4.71E-09 | hypothetical protein |
| PA14_07460 |  | | 7.31 | 1.07E-28 | 4.55 | 7.42E-18 | hypothetical protein |
| PA14_07500 |  | | -4.27 | 5.23E-20 | -9.27 | 1.48E-50 | hypothetical protein |
| PA14_07550 |  | | 54.12 | 3.49E-103 | 22.04 | 1.93E-70 | hypothetical protein |
| PA14_07630 |  | |  |  | -3.40 | 8.37E-08 | hypothetical protein |
| PA14_07650* | *ycgB* | | -12.22 | 5.67E-34 | -30.60 | 7.15E-58 | SpoVR family protein |
| PA14_07660 |  | | -11.48 | 1.84E-20 | -33.93 | 4.02E-37 | hypothetical protein |
| PA14_08100 |  | | -4.36 | 1.95E-18 | -9.33 | 3.79E-36 | hypothetical protein |
| PA14_08110 |  | | -3.96 | 6.71E-15 | -8.75 | 1.01E-32 | hypothetical protein |
| PA14_08180 |  | | -3.10 | 2.00E-09 | -9.77 | 6.21E-27 | hypothetical protein |
| PA14_08190 |  | |  |  | -4.44 | 2.36E-11 | hypothetical protein |
| PA14_08200 |  | |  |  | -3.57 | 9.29E-15 | hypothetical protein |
| PA14_08220 |  | | -3.14 | 8.96E-13 | -4.92 | 1.59E-26 | hypothetical protein |
| PA14_08230 |  | | -2.56 | 7.92E-09 | -5.45 | 8.13E-27 | hypothetical protein |
| PA14_08270 |  | | -2.51 | 3.37E-08 | -4.10 | 4.06E-19 | hypothetical protein |
| PA14_09350 | *fptB* | | -5.60 | 2.19E-16 | -3.56 | 4.03E-13 | hypothetical protein |
| PA14_09810 |  | |  |  | 7.80 | 1.09E-31 | hypothetical protein |
| PA14_10050 |  | |  |  | -3.64 | 8.48E-04 | hypothetical protein |
| PA14_10220 | *ygiM* | | 4.42 | 7.50E-15 |  |  | SH3 domain-containing protein |
| PA14_10360 |  | | -709.72 | 4.91E-231 | -129.84 | 2.51E-172 | hypothetical protein |
| PA14_10380 |  | | -44.17 | 5.36E-12 | -26.93 | 3.53E-11 | hypothetical protein |
| PA14_10410 |  | |  |  | 2.69 | 4.73E-01 | hypothetical protein |
| PA14_10490 |  | | -3.23 | 2.52E-13 |  |  | hypothetical protein |
| PA14_10560* |  | | -4.39 | 6.43E-21 | -5.03 | 4.95E-30 | hypothetical protein |
| PA14_10780 |  | | -2.66 | 1.49E-08 |  |  | hypothetical protein |
| PA14_10820 |  | | -8.44 | 4.04E-39 | -4.39 | 2.93E-25 | HDIG domain-containing protein |
| PA14_10950 |  | |  |  | -3.01 | 6.15E-07 | hypothetical protein |
| PA14_11050 | *moaF* | |  |  | 3.28 | 6.67E-05 | hypothetical protein |
| PA14_11160 |  | | 4.63 | 3.85E-11 |  |  | hypothetical protein |
| PA14_11170 |  | | 3.44 | 4.44E-13 |  |  | hypothetical protein |
| PA14_11320 |  | | -7.72 | 4.91E-09 |  |  | hypothetical protein |
| PA14_11330 |  | | -4.16 | 1.83E-17 |  |  | hypothetical protein |
| PA14_11380 | *nrdR* | | 4.18 | 1.34E-18 |  |  | transcriptional regulator NrdR |
| PA14_11520 |  | | 5.06 | 2.10E-22 | 3.04 | 2.60E-12 | hypothetical protein |
| PA14_11700 |  | | 7.06 | 3.66E-27 | 6.09 | 4.57E-25 | hypothetical protein |
| PA14_11740 |  | | -6.92 | 3.19E-07 | -7.09 | 1.85E-08 | hypothetical protein |
| PA14_11890* |  | | -11.49 | 3.15E-50 | -10.93 | 3.46E-58 | hypothetical protein |
| PA14_11920 |  | | 4.79 | 1.99E-17 | 4.09 | 3.93E-15 | hypothetical protein |
| PA14_11940 |  | | -3.49 | 1.84E-15 | -2.99 | 6.19E-15 | hypothetical protein |
| PA14_12170 |  | |  |  | 2.92 | 6.07E-02 | hypothetical protein |
| PA14_12180 |  | | 8.82 | 6.44E-26 | 9.73 | 1.30E-29 | hypothetical protein |
| PA14_12210 |  | | 5.01 | 2.64E-25 |  |  | hypothetical protein |
| PA14_12260 |  | | -40.65 | 1.33E-83 | -8.83 | 4.02E-47 | hypothetical protein |
| PA14_12350 | *orf2* | | 8.39 | 2.48E-38 | 6.95 | 3.87E-36 | (dimethylallyl)adenosine tRNA methylthiotransferase |
| PA14_12360 |  | | 6.52 | 2.03E-28 | 4.44 | 6.31E-17 | hypothetical protein |
| PA14_12470 |  | |  |  | -5.68 | 1.46E-15 | hypothetical protein |
| PA14_12530 |  | |  |  | 2.59 | 2.50E-11 | hypothetical protein |
| PA14_12550 |  | | 11.52 | 5.16E-24 | 3.59 | 2.18E-09 | hypothetical protein |
| PA14_12560 |  | | 4.32 | 1.37E-19 | 6.21 | 2.34E-33 | hypothetical protein |
| PA14_12650 |  | | 6.01 | 2.65E-18 | 4.13 | 1.86E-11 | hypothetical protein |
| PA14_12700 |  | | 5.30 | 2.08E-18 |  |  | hypothetical protein |
| PA14_12770 |  | | 3.16 | 1.17E-12 |  |  | hypothetical protein |
| PA14_12910 |  | | 2.56 | 5.16E-06 | 20.31 | 8.26E-65 | hypothetical protein |
| PA14_13010 |  | |  |  | 66.08 | 2.21E-133 | hypothetical protein |
| PA14_13050 |  | | -10.28 | 2.70E-29 | -4.35 | 2.35E-12 | hypothetical protein |
| PA14_13130 |  | | -14.56 | 2.66E-13 | -6.44 | 5.96E-10 | hypothetical protein |
| PA14_13140 |  | | -20.05 | 2.25E-69 | -11.92 | 2.28E-59 | hypothetical protein |
| PA14_13190* |  | | -3.25 | 3.62E-14 | -5.01 | 5.14E-30 | hypothetical protein |
| PA14_13200 |  | |  |  | -2.56 | 9.26E-10 | hypothetical protein |
| PA14_13210 |  | | -71.71 | 2.32E-41 | -7.17 | 4.79E-18 | hypothetical protein |
| PA14_13350 |  | | 3.37 | 3.88E-05 | 3.27 | 5.10E-13 | hypothetical protein |
| PA14_13360 |  | | 6.99 | 1.52E-08 | 4.03 | 1.38E-20 | hypothetical protein |
| PA14_13370 |  | | 6.27 | 1.83E-18 | 2.91 | 5.24E-08 | hypothetical protein |
| PA14_13380 |  | | 6.78 | 3.89E-22 |  |  | hypothetical protein |
| PA14_13390 |  | | 4.07 | 3.16E-18 |  |  | hypothetical protein |
| PA14_13670 |  | | 9.38 | 2.82E-22 |  |  | hypothetical protein |
| PA14_13710 |  | | 4.04 | 1.01E-17 | 2.60 | 4.70E-10 | hypothetical protein |
| PA14_13860 |  | |  |  | 3.26 | 1.75E-08 | hypothetical protein |
| PA14_13870 |  | |  |  | 3.07 | 5.47E-08 | hypothetical protein |
| PA14_13920 |  | | -3.42 | 1.23E-07 |  |  | hypothetical protein |
| PA14_13970 |  | |  |  | 3.55 | 1.07E-11 | hypothetical protein |
| PA14_14210 |  | | 4.68 | 9.58E-20 |  |  | hypothetical protein |
| PA14_14320 |  | |  |  | 3.44 | 4.49E-06 | hypothetical protein |
| PA14_14340 | *ybiN* | | 4.54 | 1.81E-07 | -2.76 | 5.29E-05 | SAM-dependent methyltransferase |
| PA14_14430 |  | | -4.74 | 8.10E-19 |  |  | hypothetical protein |
| PA14_14450 |  | | -2.86 | 2.36E-10 |  |  | hypothetical protein |
| PA14_14550 |  | | -3.00 | 5.46E-12 |  |  | hypothetical protein |
| PA14_14560 |  | | 26.49 | 8.25E-49 | 6.23 | 6.64E-12 | hypothetical protein |
| PA14_14975 |  | | -3.63 | 1.41E-15 | -5.58 | 1.94E-30 | hypothetical protein |
| PA14_15050 |  | | -3.95 | 3.85E-07 | -3.54 | 1.17E-07 | hypothetical protein |
| PA14_15090 |  | | -3.65 | 1.56E-14 | -4.29 | 1.71E-20 | hypothetical protein |
| PA14_15130 |  | | -7.97 | 2.55E-18 | -12.14 | 1.25E-26 | hypothetical protein |
| PA14_15140 |  | | -4.05 | 4.75E-06 | -4.85 | 7.59E-08 | hypothetical protein |
| PA14_15360 |  | | -4.03 | 1.17E-02 |  |  | hypothetical protein |
| PA14_15475 | *merT* | |  |  | 3.47 | 2.32E-09 | mercuric transport protein |
| PA14_15610 |  | | -2.84 | 2.53E-11 | -3.23 | 3.28E-17 | hypothetical protein |
| PA14_15710 |  | |  |  | 8.36 | 6.74E-44 | hypothetical protein |
| PA14_15840 |  | |  |  | 2.58 | 7.71E-12 | hypothetical protein |
| PA14_16020 |  | | -4.83 | 3.68E-23 |  |  | hypothetical protein |
| PA14_16100 |  | | -17.57 | 2.98E-60 |  |  | hypothetical protein |
| PA14_16110 |  | | -7.00 | 3.26E-32 | -3.26 | 1.86E-16 | hypothetical protein |
| PA14_16140 |  | | 5.71 | 3.46E-28 | 3.86 | 2.94E-20 | hypothetical protein |
| PA14_16160 |  | | 6.89 | 3.00E-25 |  |  | hypothetical protein |
| PA14_16180 |  | | 4.38 | 1.83E-17 |  |  | hypothetical protein |
| PA14_16190* |  | | 4.39 | 4.85E-18 |  |  | hypothetical protein |
| PA14_16210 |  | | 11.32 | 2.90E-42 | 3.28 | 6.19E-11 | hypothetical protein |
| PA14_16270 |  | | 3.01 | 3.81E-12 |  |  | hypothetical protein |
| PA14_16290 |  | | -3.42 | 5.53E-14 | 3.23 | 7.92E-17 | hypothetical protein |
| PA14_16300 |  | | -4.07 | 1.35E-15 |  |  | hypothetical protein |
| PA14_16340 |  | |  |  | -2.77 | 1.40E-07 | hypothetical protein |
| PA14_16580* |  | |  |  | -2.56 | 3.86E-07 | hypothetical protein |
| PA14_16590 |  | |  |  | -3.82 | 4.15E-19 | hypothetical protein |
| PA14_16680 |  | | -40.90 | 3.28E-80 | -16.63 | 3.75E-62 | hypothetical protein |
| PA14_16770 |  | | 3.01 | 3.36E-11 |  |  | hypothetical protein |
| PA14_16830 |  | | 3.46 | 2.16E-13 |  |  | hypothetical protein |
| PA14_16980 |  | |  |  | 3.43 | 2.55E-04 | hypothetical protein |
| PA14_16990 |  | |  |  | 5.07 | 6.13E-31 | hypothetical protein |
| PA14_17000 |  | |  |  | 3.10 | 5.58E-03 | hypothetical protein |
| PA14_17580 |  | |  |  | -7.69 | 2.36E-11 | hypothetical protein |
| PA14_17990 | *ybaK* | | 8.55 | 1.69E-34 | 3.74 | 1.78E-15 | hypothetical protein |
| PA14_18050 |  | | -6.82 | 1.15E-31 |  |  | hypothetical protein |
| PA14_18100 |  | | -5.86 | 3.08E-28 |  |  | hypothetical protein |
| PA14_18590 |  | | 4.08 | 3.83E-15 | 3.18 | 2.84E-11 | hypothetical protein |
| PA14_18810 |  | | 47.90 | 9.73E-89 | 4.43 | 6.47E-16 | hypothetical protein |
| PA14_18820 |  | | 114.86 | 9.58E-62 |  |  | hypothetical protein |
| PA14_18870 |  | | -2.82 | 5.55E-07 |  |  | hypothetical protein |
| PA14_18960 |  | |  |  | 3.99 | 7.73E-19 | hypothetical protein |
| PA14_18985 |  | |  |  | 3.40 | 6.33E-12 | hypothetical protein |
| PA14_19010 |  | | 3.48 | 5.51E-08 | 4.06 | 2.11E-18 | hypothetical protein |
| PA14_19020 |  | | 11.18 | 2.68E-45 | 5.64 | 2.50E-27 | hypothetical protein |
| PA14_19030 |  | | 4.76 | 5.07E-23 | 3.43 | 3.43E-17 | hypothetical protein |
| PA14_19205 |  | | 5.48 | 7.12E-20 | 3.94 | 1.12E-13 | hypothetical protein |
| PA14_19450 |  | | 3.11 | 1.14E-10 |  |  | hypothetical protein |
| PA14_19480 |  | | -16.47 | 3.68E-11 | -5.80 | 1.55E-07 | hypothetical protein |
| PA14_19600 |  | | 3.87 | 3.95E-17 |  |  | hypothetical protein |
| PA14_19610 |  | | 4.08 | 3.24E-18 | 2.92 | 1.35E-12 | hypothetical protein |
| PA14_19750 |  | |  |  | 3.14 | 3.02E-09 | hypothetical protein |
| PA14_19810 |  | | -2.67 | 2.12E-05 |  |  | hypothetical protein |
| PA14_19860 |  | | -3.08 | 3.29E-06 |  |  | hypothetical protein |
| PA14_19930 |  | |  |  | -2.54 | 2.32E-05 | hypothetical protein |
| PA14_19960 |  | | -3.87 | 9.55E-06 |  |  | hypothetical protein |
| PA14_20060 |  | | -2.58 | 1.33E-05 |  |  | hypothetical protein |
| PA14_20260 |  | | 8.29 | 2.94E-27 | 4.09 | 5.64E-12 | hypothetical protein |
| PA14_20460 |  | | -3.88 | 4.32E-15 | -6.27 | 6.48E-27 | hypothetical protein |
| PA14_20470 |  | | -4.35 | 4.03E-18 | -6.31 | 4.03E-29 | hypothetical protein |
| PA14_20480 |  | | -6.95 | 4.21E-30 | -3.01 | 1.47E-13 | hypothetical protein |
| PA14_20510 |  | |  |  | 2.55 | 2.46E-11 | hypothetical protein |
| PA14_20550 |  | |  |  | 2.95 | 2.14E-06 | hypothetical protein |
| PA14_20690 |  | | -3.71 | 4.01E-16 | -3.27 | 3.85E-16 | hypothetical protein |
| PA14_20880 |  | |  |  | 2.63 | 1.12E-07 | hypothetical protein |
| PA14_20920 |  | |  |  | -4.22 | 2.04E-21 | hypothetical protein |
| PA14_21190 |  | | -7.65 | 7.78E-36 | -10.40 | 1.70E-53 | hypothetical protein |
| PA14_21210 |  | | -2.56 | 2.23E-03 |  |  | hypothetical protein |
| PA14_21220 |  | | -3.80 | 1.99E-17 | -24.56 | 1.10E-93 | hypothetical protein |
| PA14_21240 |  | | -3.71 | 9.76E-16 |  |  | hypothetical protein |
| PA14_21260 |  | | -12.46 | 9.92E-16 | -11.70 | 2.05E-17 | hypothetical protein |
| PA14_21450 |  | | 4.77 | 1.18E-15 | 4.78 | 1.47E-16 | hypothetical protein |
| PA14_21460 |  | | 4.16 | 4.23E-03 | 6.14 | 7.21E-17 | hypothetical protein |
| PA14_21470 |  | |  |  | 5.17 | 1.01E-13 | hypothetical protein |
| PA14_21480 |  | | -3.19 | 1.31E-06 | 2.99 | 1.06E-10 | hypothetical protein |
| PA14_21490 |  | | -4.50 | 4.24E-21 |  |  | hypothetical protein |
| PA14_21510 |  | | -6.57 | 1.72E-18 | -4.60 | 1.32E-16 | hypothetical protein |
| PA14_21530 |  | | -5.91 | 3.25E-19 |  |  | ankyrin domain-containing protein |
| PA14_21570 |  | | -7.48 | 1.00E-09 | -17.08 | 1.09E-16 | hypothetical protein |
| PA14_21580 |  | | -3.96 | 8.33E-17 | -11.96 | 9.97E-50 | hypothetical protein |
| PA14_21590 |  | |  |  | -4.12 | 9.08E-11 | hypothetical protein |
| PA14_21630 |  | |  |  | -7.36 | 3.84E-31 | hypothetical protein |
| PA14_21650 |  | | 10.06 | 6.82E-34 | 4.07 | 1.75E-12 | hypothetical protein |
| PA14_21660 |  | | 4.14 | 4.55E-12 |  |  | hypothetical protein |
| PA14_21670 |  | | -14.85 | 1.04E-47 | -5.81 | 1.84E-26 | hypothetical protein |
| PA14_21680 |  | | -6.81 | 1.01E-29 | -6.71 | 2.73E-33 | hypothetical protein |
| PA14_21830 |  | | -3.59 | 8.33E-16 | -4.72 | 2.15E-26 | hypothetical protein |
| PA14_21860 |  | | -4.06 | 3.75E-18 | -5.10 | 5.32E-28 | hypothetical protein |
| PA14_21940 |  | | -3.16 | 4.46E-05 |  |  | hypothetical protein |
| PA14_22080 |  | |  |  | 5.22 | 2.98E-22 | resolvase |
| PA14_22090 |  | | -3.61 | 6.84E-03 | 3.21 | 9.57E-09 | hypothetical protein |
| PA14_22100 |  | |  |  | 3.07 | 1.24E-08 | hypothetical protein |
| PA14_22110 |  | | -2.61 | 2.98E-04 | 4.50 | 6.19E-20 | hypothetical protein |
| PA14_22120 |  | | -3.09 | 9.63E-03 |  |  | hypothetical protein |
| PA14_22130 |  | |  |  | 3.19 | 1.13E-04 | hypothetical protein |
| PA14_22140 |  | |  |  | 4.37 | 5.82E-12 | hypothetical protein |
| PA14_22160 |  | |  |  | 9.98 | 1.84E-04 | hypothetical protein |
| PA14_22180 |  | | 4.84 | 1.16E-11 | 18.77 | 3.15E-27 | hypothetical protein |
| PA14_22190 |  | |  |  | 9.97 | 1.14E-10 | hypothetical protein |
| PA14_22220 |  | |  |  | 2.91 | 1.31E-06 | hypothetical protein |
| PA14_22230 |  | |  |  | 4.15 | 4.12E-11 | hypothetical protein |
| PA14_22240 |  | |  |  | 5.84 | 2.27E-13 | hypothetical protein |
| PA14_22250 |  | |  |  | 5.54 | 8.50E-15 | hypothetical protein |
| PA14_22260 |  | |  |  | 9.87 | 8.58E-09 | hypothetical protein |
| PA14_22270 |  | |  |  | 2.58 | 4.33E-03 | recombinase |
| PA14_22400 |  | | -15.55 | 4.17E-20 | -5.42 | 6.85E-13 | hypothetical protein |
| PA14_22420 |  | |  |  | -2.74 | 4.80E-08 | hypothetical protein |
| PA14_22840 | *ypuG* | |  |  | -3.21 | 7.82E-16 | hypothetical protein |
| PA14_22880 |  | | -2.57 | 1.26E-06 |  |  | Fe-S protein |
| PA14_24150 |  | | 5.13 | 5.78E-23 | 4.50 | 4.76E-22 | hypothetical protein |
| PA14_24180 |  | | -2.66 | 3.86E-10 | -4.12 | 2.64E-22 | hypothetical protein |
| PA14_24210 |  | | -19.49 | 2.37E-67 | -13.70 | 3.49E-63 | hypothetical protein |
| PA14_24300 |  | | 4.53 | 2.54E-20 |  |  | hypothetical protein |
| PA14_24370 |  | | -3.14 | 6.72E-13 | -4.66 | 3.58E-25 | hypothetical protein |
| PA14_24570 |  | |  |  | 5.66 | 1.84E-22 | hypothetical protein |
| PA14_24620 |  | |  |  | 2.51 | 1.97E-10 | hypothetical protein |
| PA14_24630 |  | | -2.71 | 1.47E-04 |  |  | hypothetical protein |
| PA14_24740 |  | | -3.42 | 6.74E-15 | -18.68 | 1.62E-77 | hypothetical protein |
| PA14_24770 |  | | -5.84 | 1.94E-28 | -10.02 | 6.40E-55 | hypothetical protein |
| PA14_24980* |  | | -3.56 | 1.64E-06 |  |  | hypothetical protein |
| PA14_25030 |  | | 8.16 | 3.98E-34 |  |  | hypothetical protein |
| PA14_25050 |  | | 2.68 | 5.38E-07 |  |  | hypothetical protein |
| PA14_25100 |  | | 3.21 | 7.33E-14 |  |  | hypothetical protein |
| PA14_25140 |  | |  |  | 9.12 | 1.33E-49 | hypothetical protein |
| PA14_25470 |  | |  |  | 7.44 | 3.00E-38 | hypothetical protein |
| PA14_25520 |  | | 2.69 | 5.67E-10 |  |  | hypothetical protein |
| PA14_25620 |  | | 7.57 | 1.09E-34 | 7.72 | 1.23E-44 | hypothetical protein |
| PA14_26060 |  | | -13.58 | 1.01E-08 |  |  | hypothetical protein |
| PA14_26080 |  | |  |  | 2.78 | 1.48E-05 | hypothetical protein |
| PA14_26165 |  | |  |  | 4.15 | 3.25E-18 | hypothetical protein |
| PA14_26190 |  | | -8.98 | 4.84E-10 | -2.94 | 2.16E-05 | hypothetical protein |
| PA14_26200 |  | |  |  | 2.58 | 7.79E-05 | hypothetical protein |
| PA14_26300 |  | |  |  | -7.69 | 3.85E-13 | hypothetical protein |
| PA14_26540 |  | | 2.55 | 3.47E-09 |  |  | hypothetical protein |
| PA14_26580 |  | | 5.57 | 1.96E-14 | 3.01 | 8.39E-06 | hypothetical protein |
| PA14_26610 |  | |  |  | -2.66 | 1.69E-11 | hypothetical protein |
| PA14_26780 |  | | -14.50 | 5.61E-23 | -2.74 | 4.22E-08 | hypothetical protein |
| PA14_26980 |  | | -4.82 | 2.65E-07 |  |  | hypothetical protein |
| PA14_27170 |  | | 4.32 | 4.01E-15 | 2.59 | 4.43E-07 | hypothetical protein |
| PA14_27490 |  | | 8.08 | 1.00E-19 | 4.01 | 6.48E-08 | hypothetical protein |
| PA14_27630 | *rebB1* | | -6.09 | 4.47E-15 |  |  | protein associated with synthesis and assembly of refractile inclusion bodies |
| PA14_27650 |  | |  |  | -2.97 | 1.89E-04 | hypothetical protein |
| PA14_27660 |  | |  |  | -4.53 | 1.21E-06 | hypothetical protein |
| PA14_27720 |  | | -3.24 | 1.97E-12 | -2.80 | 1.56E-11 | hypothetical protein |
| PA14_27740 |  | | -3.30 | 9.19E-05 |  |  | hypothetical protein |
| PA14_27830 |  | |  |  | 3.32 | 6.17E-06 | hypothetical protein |
| PA14_27870 |  | | -2.77 | 7.36E-11 |  |  | hypothetical protein |
| PA14_27930 |  | | -18.44 | 1.30E-11 | -4.26 | 4.79E-06 | hypothetical protein |
| PA14_28000 |  | | 7.13 | 4.46E-31 | 3.91 | 5.37E-17 | hypothetical protein |
| PA14_28010 |  | | 7.52 | 3.95E-22 | 3.28 | 1.96E-07 | hypothetical protein |
| PA14_28020 |  | |  |  | 3.61 | 5.39E-04 | hypothetical protein |
| PA14_28030 |  | |  |  | 2.74 | 3.22E-12 | hypothetical protein |
| PA14_28040 |  | | 3.95 | 5.07E-10 | 3.11 | 5.98E-07 | hypothetical protein |
| PA14_28070 |  | |  |  | 21.38 | 3.01E-20 | hypothetical protein |
| PA14_28120 |  | | -5.51 | 7.57E-10 |  |  | hypothetical protein |
| PA14_28140 |  | | -57.13 | 5.17E-23 | -19.84 | 2.24E-17 | hypothetical protein |
| PA14_28150 |  | | -21.50 | 4.79E-70 | -29.99 | 2.18E-89 | hypothetical protein |
| PA14_28200 |  | | 5.67 | 2.66E-24 | 2.95 | 7.53E-11 | hypothetical protein |
| PA14_28210 |  | | 5.07 | 1.51E-11 | 2.65 | 1.18E-08 | hypothetical protein |
| PA14_28220 |  | | 3.74 | 5.80E-12 | 2.67 | 3.09E-07 | hypothetical protein |
| PA14_28230 |  | |  |  | 3.17 | 1.27E-08 | hypothetical protein |
| PA14_28240 |  | |  |  | 2.59 | 1.31E-08 | hypothetical protein |
| PA14_28260 |  | | -21.18 | 1.10E-70 | -7.59 | 1.78E-42 | hypothetical protein |
| PA14_28300 |  | |  |  | 3.76 | 4.76E-07 | hypothetical protein |
| PA14_28360 |  | |  |  | 8.06 | 4.31E-44 | hypothetical protein |
| PA14_28370 |  | | 3.83 | 1.20E-08 | 10.21 | 1.71E-29 | hypothetical protein |
| PA14_28380 |  | |  |  | 5.28 | 6.86E-13 | hypothetical protein |
| PA14_28410 |  | | -2.61 | 1.67E-03 |  |  | hypothetical protein |
| PA14_28460 |  | | -3.01 | 8.65E-12 | -3.68 | 5.71E-19 | hypothetical protein |
| PA14_28500 |  | | -5.97 | 1.25E-12 | -5.88 | 1.03E-14 | hypothetical protein |
| PA14_28520 |  | |  |  | -4.66 | 3.28E-05 | hypothetical protein |
| PA14_28580 |  | | 3.18 | 3.00E-09 | 8.01 | 4.44E-32 | hypothetical protein |
| PA14_28600 |  | | -7.99 | 9.18E-15 | -6.11 | 2.22E-14 | hypothetical protein |
| PA14_28610 |  | | -12.98 | 6.92E-06 |  |  | hypothetical protein |
| PA14_28620 |  | | -6.00 | 9.55E-25 | 4.51 | 8.82E-25 | hypothetical protein |
| PA14_28760 |  | | -3.19 | 5.41E-03 |  |  | hypothetical protein |
| PA14_28830 |  | |  |  | -2.55 | 2.24E-03 | hypothetical protein |
| PA14_28880 |  | | -3.00 | 1.06E-10 |  |  | hypothetical protein |
| PA14_28950 |  | | -6.65 | 7.92E-25 |  |  | hypothetical protein |
| PA14_28960 |  | | -10.32 | 1.50E-37 | -4.76 | 2.28E-21 | hypothetical protein |
| PA14_28990 |  | | 2.99 | 3.24E-09 | 2.91 | 1.91E-09 | hypothetical protein |
| PA14_29120 |  | | -4.55 | 1.05E-20 | -3.35 | 1.08E-16 | hypothetical protein |
| PA14_29190 |  | |  |  | 3.56 | 9.79E-11 | hypothetical protein |
| PA14_29200 |  | |  |  | 6.19 | 6.31E-27 | hypothetical protein |
| PA14_29250 |  | |  |  | -2.53 | 6.53E-04 | hypothetical protein |
| PA14_29320 |  | |  |  | 6.86 | 6.63E-39 | NADH dehydrogenase, FAD-containing subunit |
| PA14_29330 |  | | -49.32 | 3.62E-105 | -27.24 | 6.98E-93 | hypothetical protein |
| PA14_29390 |  | |  |  | 3.56 | 3.53E-19 | hypothetical protein |
| PA14_29470 |  | |  |  | 7.33 | 3.86E-42 | hypothetical protein |
| PA14_29575 |  | | -2.67 | 4.27E-07 |  |  | hypothetical protein |
| PA14_29650 |  | | 4.07 | 2.04E-18 |  |  | hypothetical protein |
| PA14_29690 |  | | 5.39 | 3.68E-19 | 4.99 | 1.44E-18 | hypothetical protein |
| PA14_29710 |  | | -7.46 | 3.28E-35 | -8.45 | 8.90E-47 | hypothetical protein |
| PA14_30030 |  | |  |  | 2.76 | 5.48E-02 | hypothetical protein |
| PA14_30100 |  | |  |  | -2.97 | 8.93E-09 | hypothetical protein |
| PA14_30140 |  | | 3.88 | 8.88E-17 | 3.83 | 1.29E-18 | hypothetical protein |
| PA14_30690 |  | | -4.15 | 7.48E-04 |  |  | hypothetical protein |
| PA14_30730 |  | |  |  | 2.62 | 1.46E-10 | hypothetical protein |
| PA14_31060 |  | |  |  | 5.01 | 1.06E-29 | hypothetical protein |
| PA14_31160 |  | |  |  | 2.66 | 2.25E-11 | hypothetical protein |
| PA14_31180 |  | | -3.35 | 3.46E-11 |  |  | hypothetical protein |
| PA14_31220 |  | |  |  | -3.67 | 1.64E-06 | hypothetical protein |
| PA14_31230 |  | |  |  | -2.55 | 1.77E-04 | hypothetical protein |
| PA14_31250 |  | |  |  | -3.21 | 1.52E-02 | hypothetical protein |
| PA14_31270 |  | | -6.32 | 3.98E-20 | -5.47 | 4.11E-21 | hypothetical protein |
| PA14_31300 |  | | -3.90 | 2.38E-16 |  |  | hypothetical protein |
| PA14_31340 |  | |  |  | 2.99 | 2.57E-05 | hypothetical protein |
| PA14_31360 |  | | -12.49 | 7.42E-49 |  |  | hypothetical protein |
| PA14_31390 |  | | -15.01 | 4.89E-56 | -7.64 | 3.26E-40 | hypothetical protein |
| PA14_31420 |  | |  |  | 7.14 | 4.55E-40 | hypothetical protein |
| PA14_31450 |  | | -3.09 | 6.88E-11 | -4.21 | 5.40E-18 | hypothetical protein |
| PA14_31740 |  | | 2.78 | 8.14E-07 |  |  | hypothetical protein |
| PA14_32250 |  | |  |  | 8.61 | 1.03E-27 | hypothetical protein |
| PA14_32280 |  | | -27.06 | 5.56E-82 | -27.59 | 6.87E-95 | hypothetical protein |
| PA14_32290 |  | | 3.96 | 2.80E-17 |  |  | hypothetical protein |
| PA14_32310 |  | | -9.64 | 3.78E-05 | -6.52 | 7.46E-05 | hypothetical protein |
| PA14_32480 |  | | -4.15 | 2.72E-17 | -2.62 | 1.58E-10 | hypothetical protein |
| PA14_32540 |  | | 2.52 | 2.25E-09 | -4.55 | 6.08E-23 | hypothetical protein |
| PA14_32640 |  | |  |  | -2.67 | 6.41E-02 | hypothetical protein |
| PA14_32750 |  | |  |  | 2.52 | 1.38E-04 | hypothetical protein |
| PA14_32770 |  | | 4.26 | 5.55E-18 | 2.59 | 1.68E-09 | hypothetical protein |
| PA14_32780 |  | | 11.42 | 7.36E-42 | 10.22 | 2.36E-41 | hypothetical protein |
| PA14_32790 |  | |  |  | 2.89 | 1.09E-14 | hypothetical protein |
| PA14_32905 |  | | -18.16 | 1.34E-60 | -3.03 | 3.18E-14 | hypothetical protein |
| PA14_32950 |  | | -39.73 | 2.31E-95 | -9.77 | 8.70E-52 | hypothetical protein |
| PA14_33050 |  | | -2.80 | 1.95E-05 |  |  | hypothetical protein |
| PA14_33060 |  | |  |  | 2.54 | 1.15E-07 | hypothetical protein |
| PA14_33080 |  | | -2.88 | 2.55E-06 |  |  | hypothetical protein |
| PA14_33110 |  | | -3.30 | 2.45E-05 |  |  | hypothetical protein |
| PA14_33120 |  | | -2.85 | 1.39E-10 |  |  | hypothetical protein |
| PA14_33150 |  | | -8.19 | 1.67E-27 | -6.22 | 7.29E-27 | hypothetical protein |
| PA14_33160 |  | | -15.21 | 2.70E-58 | -22.28 | 2.87E-82 | hypothetical protein |
| PA14_33190 |  | | -3.21 | 5.73E-11 | -4.10 | 1.39E-15 | hypothetical protein |
| PA14_33200 |  | |  |  | -7.03 | 2.57E-06 | hypothetical protein |
| PA14_33220 |  | | -6.86 | 1.00E-09 | -7.10 | 2.16E-11 | hypothetical protein |
| PA14_33240 |  | | -3.81 | 4.21E-09 |  |  | hypothetical protein |
| PA14_33250 |  | | -37.66 | 1.49E-28 |  |  | hypothetical protein |
| PA14_33290 |  | | -2.80 | 7.90E-05 |  |  | hypothetical protein |
| PA14_33300 |  | | -4.61 | 1.60E-14 | -2.53 | 1.82E-09 | hypothetical protein |
| PA14_33310 |  | | -4.53 | 1.50E-12 | -2.62 | 7.10E-09 | hypothetical protein |
| PA14_33320 |  | | -4.58 | 1.10E-13 |  |  | hypothetical protein |
| PA14_33330 |  | | -5.15 | 1.00E-06 |  |  | hypothetical protein |
| PA14_33360 |  | | -51.44 | 1.25E-07 |  |  | hypothetical protein |
| PA14_33370 |  | |  |  | -2.89 | 6.00E-04 | hypothetical protein |
| PA14_33570 |  | | -2.50 | 1.20E-02 | 5.03 | 8.85E-13 | hypothetical protein |
| PA14_33580 |  | | -4.72 | 8.28E-20 | 2.82 | 1.51E-07 | hypothetical protein |
| PA14_33590 |  | | -4.78 | 8.19E-06 | 2.90 | 1.61E-13 | hypothetical protein |
| PA14_33710 | *pvdO* | | -32.21 | 4.09E-80 |  |  | protein PvdO |
| PA14_33830 |  | | -86.04 | 1.06E-95 | -4.03 | 2.99E-23 | hypothetical protein |
| PA14_33870 |  | | -8.24 | 6.73E-14 |  |  | hypothetical protein |
| PA14_33930 |  | | -6.32 | 1.99E-08 | -2.67 | 1.18E-04 | hypothetical protein |
| PA14_33940 |  | | -4.83 | 8.84E-18 |  |  | hypothetical protein |
| PA14_33960 |  | | -10.24 | 1.68E-36 | -2.91 | 5.97E-12 | hypothetical protein |
| PA14_33970 |  | | -6.21 | 1.58E-22 |  |  | hypothetical protein |
| PA14_33980 |  | | -11.79 | 4.47E-15 |  |  | hypothetical protein |
| PA14_34000 |  | | -16.86 | 1.41E-34 | -6.62 | 1.33E-20 | hypothetical protein |
| PA14_34010 |  | | -11.35 | 6.88E-23 | -5.55 | 9.63E-17 | hypothetical protein |
| PA14_34020 |  | | -9.99 | 2.59E-08 | -5.71 | 7.16E-07 | hypothetical protein |
| PA14_34030 |  | | -13.11 | 1.90E-07 | -8.03 | 1.08E-06 | hypothetical protein |
| PA14_34050* |  | | -8.79 | 1.02E-35 | -4.20 | 2.30E-20 | hypothetical protein |
| PA14_34070 |  | | -5.39 | 7.11E-21 |  |  | hypothetical protein |
| PA14_34080 |  | | -14.14 | 1.70E-53 | -2.73 | 3.19E-12 | hypothetical protein |
| PA14_34100 |  | | -5.79 | 1.01E-26 | -3.54 | 7.98E-18 | hypothetical protein |
| PA14_34110 |  | | -3.83 | 1.55E-14 | -4.19 | 4.92E-18 | hypothetical protein |
| PA14_34130 |  | | -4.02 | 8.27E-18 | -2.89 | 6.89E-13 | hypothetical protein |
| PA14_34140 |  | | -9.45 | 2.69E-28 | -6.66 | 6.94E-26 | hypothetical protein |
| PA14_34170 |  | |  |  | 7.12 | 4.90E-25 | hypothetical protein |
| PA14_34610 |  | | 4.70 | 1.96E-17 | -9.04 | 4.48E-10 | hypothetical protein |
| PA14_34740 |  | |  |  | 185.13 | 1.86E-37 | hypothetical protein |
| PA14_34940 |  | | 3.44 | 2.08E-07 |  |  | hypothetical protein |
| PA14_35060 |  | | -3.00 | 3.01E-03 |  |  | hypothetical protein |
| PA14_35160 |  | | -29.81 | 6.82E-27 |  |  | hypothetical protein |
| PA14_35240 |  | |  |  | 3.14 | 1.14E-08 | hypothetical protein |
| PA14_35550 | *pslO* | | -5.58 | 5.45E-14 |  |  | hypothetical protein |
| PA14_35630 | *pslJ* | | -4.41 | 3.51E-15 | -2.97 | 6.34E-10 | hypothetical protein |
| PA14_35680 | *pslF* | | -4.24 | 1.04E-13 | -2.90 | 7.30E-09 | hypothetical protein |
| PA14_35700 |  | | -8.38 | 4.90E-05 |  |  | hypothetical protein |
| PA14_35760 |  | | -2.72 | 2.83E-05 |  |  | hypothetical protein |
| PA14_35770 |  | |  |  | 5.64 | 1.49E-33 | hypothetical protein |
| PA14_35780 |  | | 2.52 | 4.04E-04 | 4.42 | 1.40E-25 | hypothetical protein |
| PA14_35800 |  | | -2.57 | 1.49E-09 | -3.05 | 1.15E-15 | hypothetical protein |
| PA14_35810 |  | | -6.30 | 5.59E-31 | -6.56 | 2.75E-39 | hypothetical protein |
| PA14_35840 |  | | -3.16 | 5.68E-12 |  |  | hypothetical protein |
| PA14_35850 |  | | -3.25 | 4.95E-04 |  |  | hypothetical protein |
| PA14_36010 |  | | 2.68 | 3.05E-01 | 4.92 | 1.64E-02 | hypothetical protein |
| PA14_36020 |  | | 6.47 | 1.20E-01 | 11.75 | 3.33E-03 | paraquat-inducible protein B |
| PA14_36030 |  | | 5.92 | 1.34E-01 | 13.43 | 1.08E-03 | paraquat-inducible protein A |
| PA14_36190 |  | |  |  | 3.17 | 1.08E-06 | hypothetical protein |
| PA14_36250 |  | | 2.79 | 3.18E-08 | 2.51 | 2.83E-07 | hypothetical protein |
| PA14_36350 |  | | -5.89 | 2.65E-19 |  |  | hypothetical protein |
| PA14_36375 |  | | -5.01 | 1.04E-16 | -5.72 | 1.04E-18 | hypothetical protein |
| PA14_36400 |  | | -4.87 | 5.63E-14 | -5.26 | 2.84E-14 | hypothetical protein |
| PA14_36410 |  | | -8.65 | 1.15E-17 | -4.64 | 1.38E-13 | hypothetical protein |
| PA14_36450 |  | | -7.37 | 3.05E-24 | -4.68 | 4.55E-21 | hypothetical protein |
| PA14_36460 |  | | -10.22 | 5.36E-42 | -15.75 | 8.31E-61 | hypothetical protein |
| PA14_36470 |  | | -12.19 | 2.33E-07 | -5.63 | 1.14E-05 | hypothetical protein |
| PA14_36480 |  | | -7.66 | 1.98E-18 | -4.83 | 6.20E-14 | hypothetical protein |
| PA14_36490 |  | | -8.26 | 6.46E-29 | -15.16 | 4.49E-41 | hypothetical protein |
| PA14_36520 |  | | -6.68 | 3.69E-27 | -6.01 | 6.03E-27 | hypothetical protein |
| PA14_36530 |  | | -14.12 | 1.73E-48 | -12.90 | 7.55E-49 | hypothetical protein |
| PA14_36550 |  | | -3.35 | 7.46E-12 | -5.98 | 4.61E-23 | hypothetical protein |
| PA14_36560 |  | | -17.51 | 3.21E-57 | -8.20 | 6.97E-39 | hypothetical protein |
| PA14_36620 |  | | -32.12 | 2.64E-54 | -22.84 | 2.42E-43 | hypothetical protein |
| PA14_36650 |  | | -17.32 | 4.75E-59 | -18.51 | 1.46E-66 | hypothetical protein |
| PA14_36670 |  | | -5.60 | 5.95E-19 | -7.93 | 8.73E-25 | hypothetical protein |
| PA14_36770 |  | | -8.22 | 2.02E-21 | -12.52 | 1.58E-24 | hypothetical protein |
| PA14_36790 |  | | -9.58 | 7.39E-24 | -5.07 | 4.72E-14 | hypothetical protein |
| PA14_36820 |  | | -66.68 | 1.33E-102 | -5.73 | 2.02E-30 | hypothetical protein |
| PA14_36850 |  | | -15.04 | 6.81E-47 | -10.43 | 4.73E-39 | hypothetical protein |
| PA14_36860 |  | | -55.94 | 4.68E-89 | -20.36 | 2.19E-62 | hypothetical protein |
| PA14_36900 |  | |  |  | -3.98 | 1.10E-03 | hypothetical protein |
| PA14_37200 |  | |  |  | 2.84 | 9.64E-09 | hypothetical protein |
| PA14_37350 |  | |  |  | -2.69 | 5.86E-05 | hypothetical protein |
| PA14_37410 |  | |  |  | 5.36 | 3.08E-21 | hypothetical protein |
| PA14_37510 |  | | -5.45 | 1.99E-17 | 6.50 | 6.32E-06 | hypothetical protein |
| PA14_37520 |  | | -5.03 | 1.54E-13 | 5.77 | 3.86E-06 | hypothetical protein |
| PA14_37670 |  | | -10.97 | 7.27E-19 | -3.83 | 5.23E-11 | hypothetical protein |
| PA14_37780 |  | | -11.48 | 5.65E-44 | 4.40 | 6.49E-25 | hypothetical protein |
| PA14_37820 |  | | 7.45 | 1.60E-21 | 9.25 | 1.57E-34 | hypothetical protein |
| PA14_38000 |  | | 4.96 | 2.15E-14 | 10.61 | 2.19E-27 | hypothetical protein |
| PA14_38010 |  | |  |  | 4.54 | 1.72E-22 | hypothetical protein |
| PA14_38050 |  | | -3.29 | 3.08E-05 | -10.68 | 4.35E-12 | hypothetical protein |
| PA14_38060 |  | |  |  | 4.82 | 4.86E-20 | hypothetical protein |
| PA14_38180 |  | | -4.64 | 6.83E-20 |  |  | hypothetical protein |
| PA14_38190 |  | |  |  | 3.04 | 5.38E-05 | hypothetical protein |
| PA14_38260 |  | | -8.45 | 7.39E-34 | 3.04 | 8.84E-15 | hypothetical protein |
| PA14_38270 |  | | -12.48 | 2.38E-45 |  |  | hypothetical protein |
| PA14_38310 |  | |  |  | 6.67 | 3.07E-16 | hypothetical protein |
| PA14_38370 |  | | -25.52 | 6.09E-73 | -9.64 | 5.12E-47 | hypothetical protein |
| PA14_38420 |  | | -2.70 | 1.05E-04 |  |  | hypothetical protein |
| PA14_38880 |  | |  |  | -4.03 | 1.89E-03 | hypothetical protein |
| PA14_38920 |  | |  |  | 3.63 | 1.18E-06 | hypothetical protein |
| PA14_39090 |  | | -5.91 | 5.99E-27 | -36.48 | 8.64E-85 | hypothetical protein |
| PA14_39110 |  | | 5.28 | 3.05E-17 | 4.53 | 7.13E-15 | hypothetical protein |
| PA14_39140 |  | |  |  | 3.93 | 1.46E-13 | hypothetical protein |
| PA14_39220 |  | |  |  | 8.50 | 1.71E-05 | hypothetical protein |
| PA14_39270 |  | | -10.09 | 2.93E-12 | -5.15 | 2.21E-09 | hypothetical protein |
| PA14_39440 |  | |  |  | 2.89 | 3.24E-12 | hypothetical protein |
| PA14_39470 |  | |  |  | 2.58 | 1.59E-11 | hypothetical protein |
| PA14_39500 |  | | -5.58 | 2.95E-12 | -6.29 | 3.67E-15 | hypothetical protein |
| PA14_39620 |  | | -20.03 | 3.80E-62 | -5.99 | 2.26E-24 | hypothetical protein |
| PA14_39630 |  | | -10.01 | 6.64E-22 | -4.89 | 9.90E-22 | hypothetical protein |
| PA14_39660 |  | | -31.21 | 4.96E-82 | -3.23 | 2.66E-16 | hypothetical protein |
| PA14_39790* |  | | 2.54 | 1.53E-06 | 2.80 | 1.96E-10 | hypothetical protein |
| PA14_40020 |  | | -4.71 | 1.69E-14 |  |  | hypothetical protein |
| PA14_40050 |  | | -4.08 | 1.74E-10 | -3.00 | 4.53E-07 | hypothetical protein |
| PA14_40080 |  | |  |  | -2.51 | 4.89E-10 | hypothetical protein |
| PA14_40100 |  | | -59.38 | 2.11E-20 | -26.63 | 4.60E-17 | hypothetical protein |
| PA14_40110 |  | | -78.10 | 1.60E-65 | -40.40 | 3.37E-57 | hypothetical protein |
| PA14_40260 |  | | -86.82 | 3.51E-134 | -66.14 | 6.45E-139 | hypothetical protein |
| PA14_40300 |  | | -8.38 | 5.80E-36 |  |  | hypothetical protein |
| PA14_40340 |  | | 5.18 | 2.37E-08 | 5.25 | 2.29E-16 | hypothetical protein |
| PA14_40520 |  | | 11.02 | 3.06E-10 | 4.51 | 9.15E-10 | hypothetical protein |
| PA14_40650 |  | |  |  | 4.82 | 2.47E-06 | hypothetical protein |
| PA14_40660 |  | |  |  | 2.65 | 3.23E-03 | hypothetical protein |
| PA14_40690 |  | |  |  | 5.01 | 1.79E-25 | hypothetical protein |
| PA14_40700 |  | | 3.71 | 2.33E-15 | 3.37 | 7.79E-15 | hypothetical protein |
| PA14_40710 |  | | 6.07 | 4.23E-26 | 4.47 | 4.11E-20 | hypothetical protein |
| PA14_40740 |  | | -4.38 | 1.12E-12 |  |  | hypothetical protein |
| PA14_40750 |  | | -41.43 | 1.52E-71 | -13.84 | 3.69E-50 | hypothetical protein |
| PA14_40780 |  | | 2.80 | 1.05E-08 | 19.29 | 1.10E-55 | hypothetical protein |
| PA14_41030 |  | | -3.70 | 1.53E-15 |  |  | hypothetical protein |
| PA14_41420 |  | | 7.72 | 1.05E-13 | 2.62 | 1.38E-07 | hypothetical protein |
| PA14_41450 |  | |  |  | 2.73 | 8.31E-11 | hypothetical protein |
| PA14_41680 | *ydiA* | |  |  | 3.20 | 2.17E-16 | hypothetical protein |
| PA14_41690* |  | | 8.10 | 4.24E-10 | 10.69 | 6.32E-47 | hypothetical protein |
| PA14_41730* |  | | 3.07 | 4.88E-13 | 4.34 | 4.49E-24 | hypothetical protein |
| PA14_41740 |  | | -7.17 | 1.13E-29 | -3.59 | 1.29E-16 | hypothetical protein |
| PA14_41790 |  | | -3.92 | 5.81E-17 | -5.05 | 9.84E-27 | hypothetical protein |
| PA14_41860 |  | |  |  | 3.12 | 2.73E-12 | hypothetical protein |
| PA14_41970 |  | |  |  | -19.41 | 4.07E-82 | hypothetical protein |
| PA14_41980 |  | | -7.68 | 1.83E-31 | -3.43 | 1.46E-15 | hypothetical protein |
| PA14_42150 |  | |  |  | -3.40 | 1.79E-14 | hypothetical protein |
| PA14_42160 |  | |  |  | -2.83 | 6.20E-13 | hypothetical protein |
| PA14_42200 |  | | -13.07 | 1.58E-54 | -4.24 | 1.25E-24 | hypothetical protein |
| PA14_42220 |  | | 2.67 | 4.75E-10 | 3.34 | 7.92E-17 | membrane sensor domain-containing protein |
| PA14_42410 |  | | -3.88 | 1.21E-16 | -5.44 | 8.31E-28 | hypothetical protein |
| PA14_42520 | *pcr3* | |  |  | -5.40 | 9.06E-04 | hypothetical protein |
| PA14_42600 | *pscP* | |  |  | -3.15 | 1.26E-01 | translocation protein in type III secretion |
| PA14_42710 |  | | -7.59 | 6.32E-35 | -10.08 | 8.56E-51 | hypothetical protein |
| PA14_43000* |  | |  |  | 2.84 | 1.04E-13 | hypothetical protein |
| PA14_43020 |  | | 2.89 | 1.37E-07 |  |  | hypothetical protein |
| PA14_43150 |  | |  |  | 2.60 | 7.26E-08 | hypothetical protein |
| PA14_43240 |  | | 2.59 | 9.64E-08 |  |  | hypothetical protein |
| PA14_43250 |  | | -8.48 | 4.40E-26 | -5.82 | 1.60E-23 | hypothetical protein |
| PA14_43310 |  | | 10.72 | 1.08E-28 | 5.83 | 1.26E-22 | hypothetical protein |
| PA14_43760 |  | | -9.51 | 2.15E-09 | -4.51 | 5.83E-07 | hypothetical protein |
| PA14_43870 |  | | 2.64 | 2.73E-08 |  |  | hypothetical protein |
| PA14_43900 |  | |  |  | -2.95 | 3.41E-12 | hypothetical protein |
| PA14_43910 |  | | 4.34 | 1.89E-11 | 3.16 | 4.45E-07 | hypothetical protein |
| PA14_44230 |  | | 4.88 | 1.38E-03 | 3.29 | 2.87E-06 | hypothetical protein |
| PA14_44430 |  | |  |  | -3.34 | 1.29E-17 | hypothetical protein |
| PA14_44480 |  | | -9.60 | 5.78E-26 |  |  | hypothetical protein |
| PA14_44540 |  | |  |  | 3.68 | 1.95E-14 | hypothetical protein |
| PA14_44760 |  | | -3.19 | 7.57E-13 | -6.92 | 3.09E-36 | xanthine dehydrogenase accessory factor X |
| PA14_44840* |  | | -9.14 | 1.57E-11 |  |  | hypothetical protein |
| PA14_44990 |  | | -4.47 | 8.15E-11 | 14.06 | 5.10E-57 | hypothetical protein |
| PA14_45120 |  | | -3.79 | 1.68E-12 |  |  | hypothetical protein |
| PA14_45400 |  | |  |  | -3.11 | 4.69E-12 | hypothetical protein |
| PA14_45480 |  | |  |  | -4.04 | 1.14E-08 | hypothetical protein |
| PA14_45700 |  | | 4.36 | 7.96E-19 |  |  | hypothetical protein |
| PA14_45710 |  | | 4.21 | 8.76E-19 |  |  | hypothetical protein |
| PA14_45840 |  | |  |  | 3.82 | 1.11E-20 | hypothetical protein |
| PA14_45850 |  | | 4.55 | 4.40E-19 |  |  | hypothetical protein |
| PA14_46160 |  | | -3.34 | 6.88E-04 |  |  | hypothetical protein |
| PA14_46260 |  | | -2.55 | 1.56E-07 |  |  | hypothetical protein |
| PA14_46280 |  | | -6.00 | 4.90E-10 | -5.23 | 1.53E-10 | hypothetical protein |
| PA14_46340* |  | | 4.86 | 1.47E-18 | 3.83 | 1.45E-14 | hypothetical protein |
| PA14_46510 |  | | -13.93 | 1.65E-06 | -9.96 | 2.32E-06 | hypothetical protein |
| PA14_46520 |  | | -15.71 | 2.77E-06 | -11.78 | 2.71E-06 | hypothetical protein |
| PA14_46530 |  | | -17.10 | 7.02E-41 | -13.18 | 8.58E-41 | hypothetical protein |
| PA14_46540 |  | | -6.14 | 1.16E-15 | -4.41 | 5.10E-15 | hypothetical protein |
| PA14_46580 |  | | 6.02 | 4.33E-19 | 4.13 | 4.86E-12 | multidrug efflux gene product |
| PA14_46610 |  | |  |  | 3.85 | 6.96E-18 | hypothetical protein |
| PA14_46670 |  | | -4.28 | 1.40E-11 |  |  | hypothetical protein |
| PA14_46720* |  | | -3.33 | 5.09E-13 |  |  | hypothetical protein |
| PA14_46750 |  | | -16.71 | 7.55E-19 | -7.00 | 9.29E-14 | hypothetical protein |
| PA14_46760 |  | | -12.50 | 5.47E-10 | -5.19 | 4.77E-07 | hypothetical protein |
| PA14_46770 |  | | -4.28 | 6.29E-14 | -2.59 | 1.50E-07 | hypothetical protein |
| PA14_46780 |  | | -6.86 | 1.16E-22 | -6.42 | 1.96E-21 | hypothetical protein |
| PA14_46820 |  | | -7.46 | 7.79E-33 | -4.67 | 2.17E-24 | hypothetical protein |
| PA14_46830 |  | | -5.19 | 2.39E-20 | -4.31 | 3.42E-18 | hypothetical protein |
| PA14_46840 |  | | -9.57 | 2.68E-40 | -2.67 | 1.25E-11 | hypothetical protein |
| PA14_47010 |  | | -7.93 | 8.96E-26 |  |  | hypothetical protein |
| PA14_47030 |  | |  |  | 4.95 | 7.22E-24 | hypothetical protein |
| PA14_47110 | *yybH* | | 5.23 | 3.21E-23 | 4.06 | 8.28E-19 | hypothetical protein |
| PA14_47120 |  | | -7.27 | 1.84E-35 | -43.21 | 4.21E-118 | hypothetical protein |
| PA14_47130 |  | | -7.88 | 9.78E-38 | -23.54 | 8.94E-91 | hypothetical protein |
| PA14_47250 |  | | -2.96 | 2.19E-08 | -2.62 | 2.51E-07 | hypothetical protein |
| PA14_47350 |  | | 7.99 | 3.26E-36 | 7.81 | 1.46E-39 | hypothetical protein |
| PA14_47410 | *ycgN* | | 3.87 | 3.90E-12 |  |  | hypothetical protein |
| PA14_47530 |  | | -5.52 | 7.26E-04 | -8.94 | 1.13E-05 | hypothetical protein |
| PA14_47890 |  | |  |  | 2.96 | 2.14E-13 | hypothetical protein |
| PA14_47930 |  | |  |  | 3.72 | 8.48E-09 | hypothetical protein |
| PA14_48140 | *aprX* | | -30.86 | 2.54E-89 | -6.29 | 5.14E-38 | hypothetical protein |
| PA14_48170 |  | |  |  | -2.69 | 3.99E-11 | hypothetical protein |
| PA14_48380 |  | |  |  | 8.48 | 1.87E-02 | hypothetical protein |
| PA14_48510 |  | | 5.31 | 2.94E-13 | 3.24 | 2.08E-06 | hypothetical protein |
| PA14_48540 |  | |  |  | -4.33 | 4.19E-07 | hypothetical protein |
| PA14_48550 |  | |  |  | -3.98 | 2.07E-07 | hypothetical protein |
| PA14_48560 |  | | -10.23 | 4.11E-20 | -20.90 | 4.47E-31 | hypothetical protein |
| PA14_48590 |  | | -10.47 | 5.71E-43 | -39.69 | 3.04E-88 | hypothetical protein |
| PA14_48640 |  | | -3.94 | 7.38E-12 | -2.92 | 2.86E-08 | hypothetical protein |
| PA14_48650 | *yhhW* | | -2.59 | 5.24E-07 |  |  | hypothetical protein |
| PA14_48860 |  | | 4.72 | 2.52E-21 |  |  | hypothetical protein |
| PA14_49090 |  | | -4.00 | 3.75E-10 |  |  | hypothetical protein |
| PA14_49160 | *yegE* | | -3.32 | 2.81E-14 | -5.64 | 1.39E-32 | sensor protein |
| PA14_49290 |  | | 2.72 | 2.33E-08 |  |  | hypothetical protein |
| PA14_49310 |  | | -11.26 | 6.43E-44 | -14.68 | 9.52E-56 | hypothetical protein |
| PA14_49320 |  | | -13.20 | 2.08E-10 | -10.00 | 2.65E-10 | hypothetical protein |
| PA14_49330 |  | | -7.51 | 7.82E-05 | -4.42 | 4.47E-04 | hypothetical protein |
| PA14_49480 |  | | 3.79 | 6.45E-10 | 16.83 | 3.20E-73 | hypothetical protein |
| PA14_49500 |  | |  |  | 3.53 | 3.21E-09 | hypothetical protein |
| PA14_49710 | *yedU* | | -5.16 | 3.70E-19 |  |  | chaperone protein HchA |
| PA14_49720 |  | | -9.36 | 3.83E-10 |  |  | hypothetical protein |
| PA14_49740 |  | | 4.44 | 2.87E-21 |  |  | hypothetical protein |
| PA14_49810 |  | | 2.59 | 1.75E-08 | 4.64 | 1.04E-22 | hypothetical protein |
| PA14_49860 |  | | -2.69 | 2.45E-04 |  |  | hypothetical protein |
| PA14_49880 |  | |  |  | -2.91 | 2.53E-07 | hypothetical protein |
| PA14_49910 |  | | -5.97 | 1.73E-28 |  |  | hypothetical protein |
| PA14_49930 |  | |  |  | 4.16 | 8.04E-18 | hypothetical protein |
| PA14_49990 |  | |  |  | 7.06 | 4.31E-41 | hypothetical protein |
| PA14_50000 |  | | 8.64 | 5.90E-15 | 13.34 | 2.35E-57 | hypothetical protein |
| PA14_50020 |  | | -6.13 | 5.82E-27 |  |  | hypothetical protein |
| PA14_50030 |  | | 2.70 | 4.00E-09 | 2.90 | 1.43E-11 | hypothetical protein |
| PA14_50060 |  | | -8.06 | 1.77E-36 |  |  | hypothetical protein |
| PA14_50070 |  | |  |  | 3.98 | 3.94E-21 | hypothetical protein |
| PA14_50280 | *flaG* | |  |  | -4.53 | 2.41E-26 | hypothetical protein |
| PA14_50300* |  | |  |  | -6.31 | 2.48E-37 | hypothetical protein |
| PA14_50320 |  | |  |  | -3.34 | 2.45E-16 | hypothetical protein |
| PA14_50330 |  | |  |  | -2.68 | 4.47E-12 | hypothetical protein |
| PA14_50500 |  | |  |  | -7.23 | 1.04E-41 | hypothetical protein |
| PA14_50570* |  | | 3.84 | 3.99E-10 |  |  | hypothetical protein |
| PA14_50640 |  | |  |  | -3.41 | 6.08E-14 | hypothetical protein |
| PA14_50650 |  | |  |  | -3.60 | 3.51E-17 | hypothetical protein |
| PA14_50740 |  | | -4.41 | 4.25E-21 | -3.52 | 1.58E-19 | hypothetical protein |
| PA14_50790 | *yeaG* | |  |  | -3.19 | 4.09E-06 | hypothetical protein |
| PA14_50850 |  | | 3.03 | 1.31E-07 |  |  | hypothetical protein |
| PA14_50860 |  | | -2.53 | 1.13E-08 | -4.39 | 7.33E-22 | hypothetical protein |
| PA14_50870 |  | | -4.34 | 4.85E-18 | -6.93 | 1.57E-31 | hypothetical protein |
| PA14_50930 |  | | -3.49 | 2.93E-15 |  |  | hypothetical protein |
| PA14_50950 |  | |  |  | 5.84 | 4.82E-32 | hypothetical protein |
| PA14_51060 |  | | 3.29 | 4.46E-08 | 4.72 | 2.39E-14 | hypothetical protein |
| PA14_51190 |  | | 2.81 | 1.00E-09 |  |  | thiolase |
| PA14_51200 |  | | 7.26 | 1.05E-27 |  |  | hypothetical protein |
| PA14_51260 |  | | 3.94 | 7.97E-19 |  |  | hypothetical protein |
| PA14_51300 | *perM* | | 7.20 | 8.72E-32 | 2.90 | 6.98E-11 | hypothetical protein |
| PA14_51310 | *yrkI* | | 7.75 | 8.72E-28 | 5.15 | 2.22E-14 | hypothetical protein |
| PA14_51350* | *phnB* | | -2.52 | 3.47E-09 |  |  | anthranilate synthase component II |
| PA14_51380 | *pqsE* | | -2.54 | 2.69E-09 |  |  | quinolone signal response protein |
| PA14_51430 | *pqsA* | | 4.34 | 9.94E-22 |  |  | coenzyme A ligase |
| PA14_51490 |  | | -5.35 | 4.36E-22 |  |  | hypothetical protein |
| PA14_51500 |  | |  |  | 2.65 | 5.87E-05 | hypothetical protein |
| PA14_51580 |  | |  |  | 3.00 | 4.60E-04 | hypothetical protein |
| PA14_51590 |  | |  |  | 2.67 | 1.56E-11 | hypothetical protein |
| PA14_51600 |  | | -5.96 | 1.88E-11 |  |  | hypothetical protein |
| PA14_51810 | *yebC* | | 2.68 | 5.75E-11 |  |  | hypothetical protein |
| PA14_51850 |  | | -11.85 | 1.13E-48 | -11.14 | 9.36E-54 | hypothetical protein |
| PA14_51860 |  | | -9.44 | 3.81E-43 | -7.33 | 2.20E-42 | hypothetical protein |
| PA14_51910 |  | | 5.37 | 1.46E-25 | 4.13 | 4.95E-21 | hypothetical protein |
| PA14_51950 |  | | 3.31 | 6.64E-15 |  |  | hypothetical protein |
| PA14_52000 |  | | 2.92 | 3.00E-09 |  |  | hypothetical protein |
| PA14_52010* |  | | 8.90 | 2.51E-39 | 3.57 | 1.07E-15 | DNA replication initiation factor |
| PA14_52080 |  | | -4.12 | 5.55E-18 |  |  | hypothetical protein |
| PA14_52090 |  | | -4.20 | 1.91E-14 |  |  | hypothetical protein |
| PA14_52120 |  | |  |  | 2.53 | 1.64E-03 | hypothetical protein |
| PA14_52280 |  | | 3.25 | 1.02E-12 |  |  | hypothetical protein |
| PA14_52290 |  | | 4.71 | 9.79E-19 |  |  | hypothetical protein |
| PA14_52300 |  | | 2.81 | 3.56E-06 |  |  | hypothetical protein |
| PA14_52330 |  | | 3.54 | 7.77E-15 | 5.61 | 1.46E-29 | hypothetical protein |
| PA14_52440 |  | |  |  | 3.21 | 6.92E-05 | hypothetical protein |
| PA14_52520 |  | |  |  | 4.24 | 6.95E-22 | hypothetical protein |
| PA14_52730 |  | | -4.04 | 4.65E-14 |  |  | hypothetical protein |
| PA14_52760 | *aotO* | |  |  | -3.26 | 7.89E-17 | hypothetical protein |
| PA14_52940 |  | | -2.84 | 2.66E-08 |  |  | hypothetical protein |
| PA14_52960 |  | | 6.56 | 5.43E-21 | 7.41 | 3.87E-25 | hypothetical protein |
| PA14_53040* |  | | -2.53 | 4.15E-09 |  |  | lysozyme inhibitor |
| PA14_53140 |  | | -3.11 | 1.83E-13 | -3.02 | 9.69E-16 | hypothetical protein |
| PA14_53310 |  | |  |  | -2.63 | 4.27E-07 | hypothetical protein |
| PA14_53490 |  | | 4.53 | 8.42E-11 |  |  | acyltransferase |
| PA14_53500 |  | |  |  | -2.97 | 5.56E-13 | hypothetical protein |
| PA14_53670* |  | | -2.90 | 1.68E-11 |  |  | hypothetical protein |
| PA14_53680 |  | |  |  | 3.20 | 1.50E-03 | hypothetical protein |
| PA14_53740 |  | | -2.86 | 9.99E-07 |  |  | hypothetical protein |
| PA14_53750 |  | | -5.52 | 8.79E-21 | -3.11 | 1.06E-11 | hypothetical protein |
| PA14_53840 |  | |  |  | 4.41 | 3.68E-26 | hypothetical protein |
| PA14_53870 |  | |  |  | 2.63 | 1.73E-06 | hypothetical protein |
| PA14_53890 |  | |  |  | 3.35 | 4.29E-06 | hypothetical protein |
| PA14_53980* |  | | -8.42 | 6.17E-39 |  |  | hypothetical protein |
| PA14_54070 |  | | -2.56 | 9.54E-09 |  |  | hypothetical protein |
| PA14_54080 |  | | -45.02 | 8.23E-22 | -11.30 | 6.14E-14 | hypothetical protein |
| PA14_54180 |  | | -17.24 | 1.74E-64 | -6.40 | 3.66E-38 | hypothetical protein |
| PA14_54220 | *icp* | | 13.47 | 7.96E-47 | 2.85 | 4.78E-08 | inhibitor of cysteine peptidase |
| PA14_54230 |  | | 14.34 | 4.77E-43 | 13.78 | 3.19E-44 | hypothetical protein |
| PA14_54240 |  | | -14.51 | 5.34E-36 | -12.34 | 2.17E-37 | hypothetical protein |
| PA14_54270 | *yecP* | | 6.54 | 4.38E-21 | 4.79 | 8.66E-16 | hypothetical protein |
| PA14_54340 |  | |  |  | -2.70 | 2.03E-08 | hypothetical protein |
| PA14_54460 |  | | 3.91 | 3.90E-15 | 2.86 | 3.71E-10 | hypothetical protein |
| PA14_54470 |  | | 3.13 | 6.52E-12 | 2.95 | 4.54E-12 | hypothetical protein |
| PA14_54490 |  | | 3.09 | 1.16E-08 |  |  | hypothetical protein |
| PA14_54540* |  | | -6.07 | 1.73E-24 | -6.48 | 2.93E-28 | hypothetical protein |
| PA14_54550 |  | |  |  | -7.95 | 1.01E-09 | hypothetical protein |
| PA14_54570 |  | | -3.38 | 1.77E-11 | -5.04 | 3.81E-19 | hypothetical protein |
| PA14_54580 |  | | -3.32 | 4.80E-10 | -4.20 | 1.28E-13 | hypothetical protein |
| PA14_54680 |  | | -2.85 | 1.18E-07 |  |  | hypothetical protein |
| PA14_54690 | *ywnB* | | -5.81 | 2.31E-22 |  |  | hypothetical protein |
| PA14_54750 |  | | -2.71 | 2.22E-09 | -2.97 | 2.26E-12 | hypothetical protein |
| PA14_54790 |  | | 8.35 | 1.94E-20 | 9.16 | 9.36E-33 | hypothetical protein |
| PA14_54810 |  | | -5.83 | 2.85E-27 |  |  | hypothetical protein |
| PA14_54820 |  | |  |  | 7.20 | 8.33E-35 | hypothetical protein |
| PA14_54850 |  | | 13.19 | 7.80E-47 | 5.37 | 3.32E-21 | hypothetical protein |
| PA14_55080 |  | | -3.59 | 1.58E-10 |  |  | hypothetical protein |
| PA14_55090 |  | | -3.17 | 1.01E-06 |  |  | hypothetical protein |
| PA14_55100 |  | |  |  | -3.27 | 2.07E-12 | hypothetical protein |
| PA14_55110 |  | | -31.58 | 5.27E-05 | -9.60 | 1.16E-03 | hypothetical protein |
| PA14_55117 |  | | 3.24 | 1.70E-12 |  |  | hypothetical protein |
| PA14_55260 |  | | -2.68 | 9.85E-06 | -2.52 | 4.60E-05 | hypothetical protein |
| PA14_55290 |  | |  |  | -3.90 | 1.06E-06 | hypothetical protein |
| PA14_55300 |  | |  |  | -2.68 | 1.84E-02 | hypothetical protein |
| PA14_55400 |  | | -3.21 | 3.09E-13 |  |  | hypothetical protein |
| PA14_55410 |  | | -4.94 | 4.10E-14 |  |  | hypothetical protein |
| PA14_55510 | *hxcP* | | -3.31 | 1.56E-07 |  |  | hypothetical protein |
| PA14_55570 | *pigB* | | -4.04 | 2.36E-12 |  |  | hypothetical protein |
| PA14_55760 |  | | 5.91 | 1.78E-24 | 7.77 | 1.47E-25 | hypothetical protein |
| PA14_55840 |  | | -95.52 | 1.26E-121 | -149.19 | 3.45E-135 | hypothetical protein |
| PA14_55980 | *yjgR* | |  |  | -3.47 | 1.66E-18 | hypothetical protein |
| PA14_56090 |  | | 7.60 | 9.81E-35 | 4.30 | 5.65E-21 | hypothetical protein |
| PA14_56130 |  | | 10.30 | 2.17E-27 | 7.22 | 2.10E-31 | hypothetical protein |
| PA14_56140 |  | | 7.67 | 2.90E-33 | 7.87 | 5.43E-21 | hypothetical protein |
| PA14_56210 |  | | -2.58 | 9.00E-09 |  |  | hypothetical protein |
| PA14_56520 |  | |  |  | -2.84 | 7.48E-10 | hypothetical protein |
| PA14_56550* |  | | 3.18 | 1.69E-13 |  |  | hypothetical protein |
| PA14_56670 |  | | -6.85 | 6.00E-33 | -59.13 | 9.77E-123 | hypothetical protein |
| PA14_56730* |  | | -5.85 | 1.05E-04 | -8.42 | 2.80E-06 | hypothetical protein |
| PA14_56750 |  | |  |  | 8.71 | 9.56E-12 | hypothetical protein |
| PA14_56800 |  | | -5.59 | 2.58E-26 | -7.30 | 2.21E-40 | hypothetical protein |
| PA14_56910 |  | | -6.37 | 3.85E-30 | -5.86 | 1.46E-33 | hypothetical protein |
| PA14_56930 |  | |  |  | 3.26 | 6.10E-17 | hypothetical protein |
| PA14_56960 |  | | -4.62 | 5.29E-11 |  |  | hypothetical protein |
| PA14_56990 |  | | -25.22 | 4.08E-15 | -4.36 | 4.47E-07 | hypothetical protein |
| PA14_57070 |  | | 2.63 | 3.23E-08 |  |  | hypothetical protein |
| PA14_57250 |  | |  |  | 2.75 | 9.99E-12 | hypothetical protein |
| PA14_57690 |  | | 2.94 | 3.35E-12 | 2.94 | 3.28E-14 | hypothetical protein |
| PA14_57820 |  | | 7.05 | 1.03E-33 | 5.02 | 2.92E-10 | hypothetical protein |
| PA14_57910 |  | | 4.52 | 5.30E-22 | 2.53 | 1.16E-10 | hypothetical protein |
| PA14_57920 |  | | 3.98 | 3.02E-19 | 2.83 | 1.89E-13 | hypothetical protein |
| PA14_58010 |  | | -24.89 | 1.19E-79 | -14.95 | 5.55E-71 | hypothetical protein |
| PA14_58040 |  | | -15.09 | 8.33E-60 | -3.41 | 1.27E-18 | hypothetical protein |
| PA14_58260 |  | | 4.85 | 2.57E-15 | 3.21 | 1.25E-05 | hypothetical protein |
| PA14_58290 |  | | 4.18 | 8.02E-20 | 2.51 | 2.02E-10 | hypothetical protein |
| PA14_58330^‡^ |  | | -3.64 | 2.52E-15 |  |  | hypothetical protein |
| PA14_58620 |  | | -2.77 | 1.20E-07 |  |  | hypothetical protein |
| PA14_58690 |  | | -5.33 | 4.77E-26 | -5.97 | 4.41E-36 | hypothetical protein |
| PA14_58720 |  | | 2.75 | 5.41E-11 |  |  | hypothetical protein |
| PA14_58800 |  | | -3.44 | 1.62E-08 | -5.33 | 1.88E-14 | hypothetical protein |
| PA14_58810 |  | |  |  | -2.53 | 1.27E-09 | hypothetical protein |
| PA14_58820 |  | | -3.27 | 2.16E-13 | -4.48 | 5.63E-23 | hypothetical protein |
| PA14_58840 |  | | -5.24 | 8.97E-25 | -3.09 | 1.36E-15 | hypothetical protein |
| PA14_58850 |  | | -4.61 | 2.34E-21 | -4.64 | 5.40E-26 | hypothetical protein |
| PA14_58960 |  | | -2.63 | 9.68E-07 |  |  | hypothetical protein |
| PA14_59000 |  | |  |  | -2.99 | 2.76E-06 | hypothetical protein |
| PA14_59010 |  | |  |  | -3.32 | 7.16E-06 | hypothetical protein |
| PA14_59020 |  | | -3.68 | 3.89E-07 | -3.46 | 5.94E-07 | hypothetical protein |
| PA14_59030 |  | |  |  | -2.80 | 1.39E-03 | hypothetical protein |
| PA14_59050 |  | | -3.19 | 3.20E-06 | -4.70 | 6.17E-10 | nucleoid-associated protein NdpA |
| PA14_59060 |  | | -4.23 | 1.90E-05 | -3.16 | 8.20E-05 | hypothetical protein |
| PA14_59070 |  | | -2.64 | 1.47E-07 |  |  | hypothetical protein |
| PA14_59120 |  | | -2.90 | 6.64E-07 |  |  | hypothetical protein |
| PA14_59130 |  | |  |  | -3.45 | 3.96E-13 | hypothetical protein |
| PA14_59140 |  | |  |  | -3.54 | 6.76E-13 | hypothetical protein |
| PA14_59190 |  | | -7.01 | 5.62E-33 | -2.86 | 7.40E-14 | hypothetical protein |
| PA14_59370 |  | | -6.06 | 5.00E-04 |  |  | hypothetical protein |
| PA14_59380 |  | | -3.33 | 5.15E-03 |  |  | hypothetical protein |
| PA14_59400 |  | | -4.69 | 1.15E-04 | -4.30 | 3.87E-05 | hypothetical protein |
| PA14_59410 |  | | -4.38 | 2.80E-11 |  |  | hypothetical protein |
| PA14_59430 |  | | -8.73 | 1.09E-06 | -5.03 | 1.26E-05 | hypothetical protein |
| PA14_59440 |  | | -4.08 | 5.10E-06 |  |  | hypothetical protein |
| PA14_59470 |  | | -5.95 | 2.93E-05 | -6.14 | 3.44E-06 | hypothetical protein |
| PA14_59480 |  | | -4.36 | 3.01E-08 | -2.99 | 6.24E-07 | hypothetical protein |
| PA14_59490 |  | | -4.46 | 3.55E-07 | -3.12 | 7.12E-06 | hypothetical protein |
| PA14_59500 |  | | -3.59 | 1.13E-06 | -3.25 | 5.00E-07 | hypothetical protein |
| PA14_59510 |  | | -4.63 | 6.53E-07 |  |  | hypothetical protein |
| PA14_59520 |  | | -3.91 | 6.15E-11 | -2.78 | 8.82E-10 | hypothetical protein |
| PA14_59550 |  | | 8.73 | 8.52E-31 | 9.86 | 4.56E-52 | hypothetical protein |
| PA14_59610 |  | | -2.80 | 2.89E-03 |  |  | hypothetical protein |
| PA14_59620 |  | | -4.41 | 2.16E-03 |  |  | hypothetical protein |
| PA14_59650 |  | | -4.68 | 3.28E-13 | -2.72 | 6.72E-07 | hypothetical protein |
| PA14_59670 |  | |  |  | -5.09 | 5.98E-09 | hypothetical protein |
| PA14_59680 |  | |  |  | -2.61 | 4.93E-04 | hypothetical protein |
| PA14_59690 |  | | -2.95 | 1.00E-09 |  |  | hypothetical protein |
| PA14_59700 |  | |  |  | -2.58 | 5.31E-04 | hypothetical protein |
| PA14_59840 |  | | -2.64 | 7.92E-02 | -5.01 | 4.55E-03 | hypothetical protein |
| PA14_59845 |  | | -4.79 | 3.64E-05 |  |  | hypothetical protein |
| PA14_59870 |  | |  |  | -6.02 | 1.96E-07 | hypothetical protein |
| PA14_59880 |  | | -2.50 | 2.87E-04 | -3.58 | 5.90E-08 | hypothetical protein |
| PA14_59930 |  | |  |  | -2.91 | 3.47E-06 | hypothetical protein |
| PA14_59940 |  | |  |  | -2.55 | 8.75E-10 | hypothetical protein |
| PA14_59950 |  | | -2.89 | 1.49E-02 |  |  | hypothetical protein |
| PA14_59970 |  | | -3.77 | 8.74E-04 |  |  | hypothetical protein |
| PA14_59990 |  | | -3.09 | 4.92E-08 | -2.87 | 2.42E-07 | hypothetical protein |
| PA14_60010 |  | |  |  | -2.99 | 3.76E-05 | hypothetical protein |
| PA14_60020 |  | | -3.06 | 8.81E-07 |  |  | hypothetical protein |
| PA14_60080 |  | |  |  | -3.44 | 3.36E-06 | hypothetical protein |
| PA14_60100 | *dtd* | | 3.26 | 1.35E-14 |  |  | deoxycytidine triphosphate deaminase |
| PA14_60200 |  | | 4.94 | 1.94E-23 |  |  | hypothetical protein |
| PA14_60445 | *obgE* | | 2.65 | 5.11E-06 |  |  | GTPase ObgE |
| PA14_60480 |  | | -16.47 | 7.11E-63 | -2.99 | 2.51E-15 | hypothetical protein |
| PA14_60520 |  | | -31.54 | 2.19E-87 | -18.18 | 1.83E-76 | hypothetical protein |
| PA14_60540 |  | | -2.82 | 3.42E-10 |  |  | hypothetical protein |
| PA14_60560* |  | | -8.61 | 1.25E-40 | -17.31 | 7.77E-77 | hypothetical protein |
| PA14_60570 |  | |  |  | -2.56 | 1.13E-11 | hypothetical protein |
| PA14_60580 |  | | 3.44 | 2.11E-15 |  |  | hypothetical protein |
| PA14_60650* |  | |  |  | 3.58 | 8.60E-18 | hypothetical protein |
| PA14_60870 | *morA* | |  |  | 3.33 | 8.02E-18 | motility regulator |
| PA14_60900 |  | |  |  | -3.45 | 1.70E-17 | hypothetical protein |
| PA14_60930 |  | | -2.82 | 4.50E-11 | -6.57 | 2.28E-37 | hypothetical protein |
| PA14_60960 |  | | -69.69 | 2.22E-87 | -93.47 | 2.10E-103 | hypothetical protein |
| PA14_60970 |  | | -13.88 | 5.08E-57 | -3.53 | 1.34E-19 | hypothetical protein |
| PA14_61010 |  | | -7.31 | 8.97E-12 | -12.30 | 4.80E-18 | hypothetical protein |
| PA14_61200 |  | | -10.09 | 5.39E-21 | -2.62 | 1.31E-08 | hypothetical protein |
| PA14_61340 |  | | -6.16 | 6.33E-27 | -4.50 | 1.26E-22 | hypothetical protein |
| PA14_61350 |  | | -7.63 | 2.12E-33 | -3.74 | 7.41E-19 | hypothetical protein |
| PA14_61370 |  | |  |  | 5.12 | 1.01E-22 | hypothetical protein |
| PA14_61380 |  | | -21.03 | 1.09E-49 |  |  | hypothetical protein |
| PA14_61410 |  | | -24.52 | 1.88E-61 | -25.18 | 1.59E-68 | hypothetical protein |
| PA14_61430 |  | | 4.51 | 2.76E-17 | 2.80 | 5.49E-09 | hypothetical protein |
| PA14_61440 |  | | 4.19 | 2.54E-16 |  |  | hypothetical protein |
| PA14_61500 |  | | -12.64 | 3.05E-46 | -7.61 | 9.65E-36 | hypothetical protein |
| PA14_61510 |  | | -14.72 | 9.93E-43 | -24.41 | 8.83E-51 | hypothetical protein |
| PA14_61520 |  | |  |  | -8.36 | 9.97E-13 | hypothetical protein |
| PA14_61650 | *pagL* | | -3.44 | 7.79E-12 | -3.06 | 1.80E-12 | Lipid A 3-O-deacylase |
| PA14_61720 |  | | 3.49 | 1.15E-15 | 3.20 | 4.96E-16 | hypothetical protein |
| PA14_61870 |  | |  |  | -2.91 | 1.83E-10 | hypothetical protein |
| PA14_61890 |  | | 3.69 | 3.13E-15 |  |  | hypothetical protein |
| PA14_61910 |  | | -6.79 | 2.25E-07 | -20.20 | 1.20E-13 | hypothetical protein |
| PA14_61920 |  | | -3.42 | 3.15E-13 | -14.60 | 2.30E-49 | hypothetical protein |
| PA14_61940 |  | |  |  | -4.47 | 4.12E-20 | hypothetical protein |
| PA14_61950 |  | | 4.32 | 8.19E-20 | -5.40 | 2.15E-21 | hypothetical protein |
| PA14_61960 |  | | 6.28 | 1.13E-25 | 3.01 | 2.57E-10 | hypothetical protein |
| PA14_61980 |  | | 10.47 | 5.58E-30 | 5.59 | 2.35E-15 | hypothetical protein |
| PA14_61990 |  | | 3.27 | 2.51E-14 | 2.59 | 3.24E-11 | hypothetical protein |
| PA14_62240 |  | | -12.24 | 2.46E-51 | -9.40 | 1.99E-50 | hypothetical protein |
| PA14_62250 |  | | -7.45 | 2.47E-32 | -5.17 | 8.92E-27 | hypothetical protein |
| PA14_62380 |  | | -4.35 | 3.10E-19 | -2.58 | 6.09E-11 | hypothetical protein |
| PA14_62520 |  | | 4.54 | 1.98E-16 | 2.81 | 2.45E-08 | hypothetical protein |
| PA14_62650 |  | |  |  | -4.23 | 1.01E-24 | hypothetical protein |
| PA14_62660* |  | |  |  | -4.51 | 3.73E-04 | hypothetical protein |
| PA14_62670 |  | |  |  | -4.35 | 1.69E-12 | hypothetical protein |
| PA14_62680 |  | | -12.82 | 2.72E-16 | -69.22 | 6.19E-35 | hypothetical protein |
| PA14_62690 |  | | -8.68 | 2.04E-17 | -67.74 | 1.18E-46 | hypothetical protein |
| PA14_62780* | *yhbC* | | 36.16 | 3.12E-97 | 9.65 | 8.64E-50 | hypothetical protein |
| PA14_63220 |  | | 2.89 | 3.00E-09 |  |  | hypothetical protein |
| PA14_63300 |  | | 5.19 | 1.77E-15 | 3.43 | 9.95E-09 | hypothetical protein |
| PA14_63380 |  | | 3.40 | 3.24E-11 |  |  | hypothetical protein |
| PA14_63410 |  | | 7.55 | 2.11E-30 | 3.00 | 5.71E-10 | hypothetical protein |
| PA14_63420 |  | | 5.91 | 1.26E-14 |  |  | hypothetical protein |
| PA14_63430 |  | | 3.66 | 9.59E-14 |  |  | hypothetical protein |
| PA14_63650 |  | | 14.49 | 5.65E-23 | 4.83 | 4.86E-14 | hypothetical protein |
| PA14_63660 |  | | 8.74 | 6.34E-37 | 4.05 | 2.40E-17 | hypothetical protein |
| PA14_63680 |  | |  |  | 3.30 | 1.23E-03 | hypothetical protein |
| PA14_63740 |  | |  |  | -3.49 | 8.90E-05 | hypothetical protein |
| PA14_63820 |  | | 15.97 | 2.27E-26 | 23.71 | 2.40E-18 | hypothetical protein |
| PA14_63860 |  | | -3.68 | 1.21E-07 |  |  | hypothetical protein |
| PA14_63920 |  | | -11.19 | 1.54E-48 |  |  | hypothetical protein |
| PA14_63940 |  | | -14.27 | 6.27E-55 |  |  | hypothetical protein |
| PA14_64030 |  | |  |  | -3.19 | 6.33E-11 | hypothetical protein |
| PA14_64120 |  | | 14.36 | 2.61E-37 | 3.67 | 9.66E-07 | hypothetical protein |
| PA14_64170 |  | | 7.40 | 1.87E-34 | 3.72 | 1.05E-17 | hypothetical protein |
| PA14_64420 |  | | 3.97 | 6.69E-10 |  |  | hypothetical protein |
| PA14_64430 |  | | 4.91 | 1.21E-19 | 7.41 | 6.54E-34 | hypothetical protein |
| PA14_64470 |  | |  |  | -2.96 | 1.36E-04 | hypothetical protein |
| PA14_64490 |  | | -5.65 | 1.63E-25 | -4.23 | 4.95E-22 | hypothetical protein |
| PA14_64530 |  | |  |  | 14.86 | 1.64E-33 | hypothetical protein |
| PA14_65090 |  | | -17.17 | 2.76E-65 | -10.16 | 2.39E-55 | hypothetical protein |
| PA14_65520* |  | |  |  | -3.24 | 1.36E-07 | hypothetical protein |
| PA14_65640 |  | | 4.92 | 1.72E-19 |  |  | hypothetical protein |
| PA14_66020 |  | | 3.93 | 1.01E-08 | 2.89 | 5.61E-11 | hypothetical protein |
| PA14_66140 |  | | 4.12 | 7.19E-20 |  |  | hypothetical protein |
| PA14_66340 |  | | -7.92 | 3.14E-37 | -7.97 | 2.22E-44 | hypothetical protein |
| PA14_66800 |  | | 5.04 | 3.30E-11 | 2.66 | 5.81E-10 | hypothetical protein |
| PA14_66910* |  | | 2.63 | 1.75E-09 |  |  | hypothetical protein |
| PA14_66990 |  | | 3.27 | 1.34E-13 |  |  | 16S ribosomal RNA methyltransferase RsmE |
| PA14_67020 |  | |  |  | 2.60 | 4.67E-05 | hypothetical protein |
| PA14_67190 |  | | 2.54 | 8.94E-08 | 3.94 | 6.00E-17 | hypothetical protein |
| PA14_67220 |  | |  |  | 3.68 | 8.69E-19 | hypothetical protein |
| PA14_67230 |  | |  |  | 3.00 | 7.20E-13 | hypothetical protein |
| PA14_67540 |  | | -6.74 | 2.27E-31 | -8.69 | 4.72E-45 | hypothetical protein |
| PA14_67620 |  | | 15.16 | 1.87E-25 | 2.50 | 7.72E-05 | hypothetical protein |
| PA14_67830 |  | | 14.36 | 6.19E-40 | 3.09 | 3.84E-12 | hypothetical protein |
| PA14_67900 |  | | 3.97 | 2.15E-17 |  |  | hypothetical protein |
| PA14_67940 |  | | 3.30 | 1.78E-07 |  |  | hypothetical protein |
| PA14_67960 |  | | 2.91 | 5.62E-09 |  |  | hypothetical protein |
| PA14_67975* |  | | 3.99 | 6.45E-19 |  |  | hypothetical protein |
| PA14_68100 |  | | 9.07 | 2.41E-27 |  |  | hypothetical protein |
| PA14_68450 |  | |  |  | -3.50 | 3.14E-04 | hypothetical protein |
| PA14_68460 |  | |  |  | -2.65 | 2.40E-02 | hypothetical protein |
| PA14_68470 |  | |  |  | -2.93 | 2.19E-07 | hypothetical protein |
| PA14_68570 |  | | -3.47 | 3.90E-13 |  |  | hypothetical protein |
| PA14_68620 |  | | 2.92 | 6.00E-09 | 4.54 | 1.41E-18 | hypothetical protein |
| PA14_68720 |  | | 5.38 | 8.67E-19 | 2.56 | 2.28E-06 | hypothetical protein |
| PA14_68840 |  | | -3.74 | 8.20E-17 | -2.75 | 4.29E-13 | hypothetical protein |
| PA14_68940 |  | | -6.37 | 8.98E-30 |  |  | hypothetical protein |
| PA14_69050 |  | | -5.35 | 8.47E-09 |  |  | hypothetical protein |
| PA14_69330 |  | | 4.22 | 1.51E-19 |  |  | hypothetical protein |
| PA14_69350 |  | | -3.29 | 1.17E-08 |  |  | hypothetical protein |
| PA14_69600 |  | | -4.22 | 2.46E-19 | -5.70 | 2.13E-32 | hypothetical protein |
| PA14_69770 |  | | -2.69 | 3.20E-10 | -3.43 | 6.54E-18 | hypothetical protein |
| PA14_69820 |  | | 3.97 | 3.09E-12 | 5.79 | 1.07E-28 | hypothetical protein |
| PA14_70050 |  | | -2.58 | 1.71E-09 | -4.93 | 3.36E-28 | hypothetical protein |
| PA14_70170 |  | |  |  | 2.77 | 9.70E-11 | hypothetical protein |
| PA14_70430 | *yicC* | | 5.01 | 3.48E-24 |  |  | hypothetical protein |
| PA14_70590 |  | |  |  | 2.64 | 8.32E-11 | hypothetical protein |
| PA14_71080 |  | | -7.24 | 1.49E-30 | -6.98 | 2.82E-33 | hypothetical protein |
| PA14_71230 |  | | -7.44 | 9.84E-18 | -7.82 | 1.42E-19 | hypothetical protein |
| PA14_71320 |  | |  |  | 5.37 | 5.54E-23 | hypothetical protein |
| PA14_71340 |  | |  |  | 3.85 | 9.93E-04 | hypothetical protein |
| PA14_71360 |  | | 3.64 | 2.00E-08 | 3.19 | 8.32E-07 | hypothetical protein |
| PA14_71380 |  | | -3.24 | 1.64E-13 | -4.37 | 1.09E-23 | hypothetical protein |
| PA14_71400 |  | |  |  | -3.18 | 2.41E-06 | hypothetical protein |
| PA14_71580 |  | |  |  | -4.63 | 4.96E-25 | hypothetical protein |
| PA14_71760 |  | | 4.65 | 1.53E-20 |  |  | hypothetical protein |
| PA14_71830 |  | | 4.41 | 7.17E-10 |  |  | hypothetical protein |
| PA14_71840* |  | | 5.67 | 1.49E-28 |  |  | hypothetical protein |
| PA14_71850 |  | | 4.43 | 3.40E-19 | 2.58 | 2.44E-09 | hypothetical protein |
| PA14_71900 |  | | -3.63 | 5.71E-04 |  |  | hypothetical protein |
| PA14_72020 |  | | 3.81 | 7.96E-18 |  |  | hypothetical protein |
| PA14_72030 |  | | 3.52 | 1.69E-15 |  |  | hypothetical protein |
| PA14_72070 |  | |  |  | -2.71 | 8.53E-13 | hypothetical protein |
| PA14_72080 |  | | 9.62 | 6.13E-37 | 4.91 | 1.36E-19 | hypothetical protein |
| PA14_72090 |  | | 3.46 | 7.25E-14 |  |  | hypothetical protein |
| PA14_72110 |  | | 5.13 | 6.44E-25 |  |  | hypothetical protein |
| PA14_72150 |  | |  |  | 3.50 | 2.06E-13 | hypothetical protein |
| PA14_72210 |  | | 14.82 | 2.97E-57 |  |  | hypothetical protein |
| PA14_72350 |  | | -20.85 | 2.52E-10 | -4.32 | 1.18E-04 | hypothetical protein |
| PA14_72360 |  | | -5.51 | 6.46E-27 | -29.15 | 1.27E-95 | hypothetical protein |
| PA14_72370 |  | | -6.00 | 1.02E-28 | -20.20 | 8.74E-79 | hypothetical protein |
| PA14_72520^‡^* |  | |  |  | -8.04 | 4.18E-20 | hypothetical protein |
| PA14_72930 |  | | 3.20 | 1.19E-13 | 7.06 | 2.79E-40 | hypothetical protein |
| PA14_73000 |  | | -11.66 | 7.41E-50 | -2.75 | 2.09E-13 | hypothetical protein |
| PA14_73030 |  | | -3.75 | 1.32E-16 |  |  | hypothetical protein |
| PA14_73050 |  | | -95.50 | 1.93E-133 | -5.52 | 3.41E-33 | GTP cyclohydrolase |
| PA14_73100 |  | |  |  | -7.41 | 8.87E-06 | hypothetical protein |
| PA14_73390 |  | | 2.89 | 2.72E-06 |  |  | hypothetical protein |
| **Membrane proteins** | | |  |  |  |  |  |
| PA14_00140 |  | | 3.32 | 1.23E-09 | 2.87 | 8.07E-08 | hypothetical protein |
| PA14_00510 |  | |  |  | 3.23 | 4.45E-17 | hemagglutinin |
| PA14_02020 |  | | 6.61 | 7.17E-31 |  |  | outer membrane porin |
| PA14_02060 |  | | 4.56 | 3.00E-11 | 4.15 | 7.71E-10 | hypothetical protein |
| PA14_02370 |  | |  |  | 2.52 | 3.73E-04 | porin |
| PA14_02410 |  | |  |  | 3.37 | 2.46E-07 | TonB-dependent receptor |
| PA14_02490 | *tonB2* | |  |  | 45.29 | 1.09E-27 | hypothetical protein |
| PA14_02970 |  | | 4.18 | 2.16E-14 |  |  | hypothetical protein |
| PA14_03590 |  | |  |  | 3.55 | 7.38E-06 | hypothetical protein |
| PA14_03620 |  | | 3.41 | 4.71E-08 | 3.79 | 1.26E-09 | hypothetical protein |
| PA14_04290 |  | | 2.56 | 5.89E-09 |  |  | hypothetical protein |
| PA14_04330 |  | | -3.63 | 2.04E-16 | -8.52 | 5.13E-46 | hypothetical protein |
| PA14_04350 |  | |  |  | -2.69 | 2.24E-08 | integral membrane protein |
| PA14_04440 |  | | 6.53 | 9.78E-25 |  |  | permease |
| PA14_04960 |  | | 4.88 | 9.85E-19 | 2.89 | 4.15E-09 | hypothetical protein |
| PA14_05130 |  | | 2.94 | 2.95E-12 |  |  | hypothetical protein |
| PA14_05300 |  | | 5.51 | 9.04E-25 | 2.90 | 1.94E-11 | TonB domain-containing protein |
| PA14_05550 | *oprM* | |  |  | -4.75 | 3.85E-28 | major intrinsic multiple antibiotic resistance efflux outer membrane protein |
| PA14_05640 | *bfrG* | | -3.29 | 9.95E-12 | -2.95 | 2.92E-11 | hypothetical protein |
| PA14_05880 | *nfeD* | | -20.15 | 1.66E-70 | -9.68 | 9.30E-53 | membrane-bound protease |
| PA14_05890 |  | | -8.10 | 1.24E-34 | -6.83 | 4.58E-34 | stomatin-like protein |
| PA14_06320 | *rarD* | | 3.57 | 3.25E-14 |  |  | hypothetical protein |
| PA14_07010 |  | | 2.89 | 6.94E-08 |  |  | hypothetical protein |
| PA14_07020 |  | |  |  | -3.30 | 1.63E-05 | hypothetical protein |
| PA14_07580 |  | |  |  | 2.90 | 2.14E-12 | glycerol-3-phosphate acyltransferase PlsY |
| PA14_08450 |  | | 3.36 | 2.52E-14 |  |  | hypothetical protein |
| PA14_08470 |  | | 3.53 | 5.57E-13 | 2.90 | 2.22E-10 | hypothetical protein |
| PA14_09370 |  | | -3.27 | 6.82E-14 |  |  | hypothetical protein |
| PA14_09500 | *opmD* | | -57.47 | 8.55E-108 | -9.78 | 1.02E-53 | outer membrane protein |
| PA14_09540 | *mexG* | | -38.23 | 1.06E-74 | -4.24 | 8.18E-24 | hypothetical protein |
| PA14_10200 |  | |  |  | 2.66 | 3.13E-07 | TonB-dependent receptor protein |
| PA14_11270 | *oprG* | |  |  | -6.31 | 4.49E-38 | outer membrane protein OprG precursor |
| PA14_11290 |  | | -3.04 | 3.56E-09 |  |  | permease |
| PA14_11720 |  | | 8.29 | 2.10E-33 | 7.36 | 2.05E-32 | hypothetical protein |
| PA14_13450 | *fecR* | | -2.71 | 1.22E-09 | -2.87 | 3.51E-12 | transmembrane sensor |
| PA14_14500 | *yjgP* | | 4.17 | 1.79E-19 | 2.50 | 3.57E-08 | hypothetical protein |
| PA14_14510 | *yjgQ* | | 4.89 | 5.73E-24 | 2.53 | 1.35E-10 | hypothetical protein |
| PA14_14520 |  | | 2.62 | 1.38E-07 | 2.54 | 1.49E-07 | hypothetical protein |
| PA14_14530 |  | |  |  | -2.51 | 3.45E-10 | hypothetical protein |
| PA14_15080 |  | | -4.13 | 1.79E-18 |  |  | hypothetical protein |
| PA14_15100 |  | | -6.07 | 2.51E-28 | -5.16 | 9.12E-29 | hypothetical protein |
| PA14_15120 |  | | -10.35 | 6.34E-31 | -12.61 | 1.85E-39 | hypothetical protein |
| PA14_15940 |  | | 6.15 | 1.17E-10 | 2.89 | 5.74E-11 | hypothetical protein |
| PA14_16370 |  | | -7.03 | 1.55E-33 |  |  | hypothetical protein |
| PA14_16630 |  | | -4.15 | 1.20E-19 | -49.43 | 2.05E-125 | outer membrane protein, OmpA |
| PA14_17140 | *yaeL* | |  |  | 2.58 | 9.48E-12 | membrane-associated zinc metalloprotease |
| PA14_17660 |  | | 3.97 | 1.03E-12 | 3.34 | 2.73E-10 | hypothetical protein |
| PA14_17920 | *glpM* | | 5.07 | 2.70E-05 | 4.33 | 2.88E-10 | membrane protein GlpM |
| PA14_18320 |  | |  |  | -3.44 | 3.02E-01 | hypothetical protein |
| PA14_18660 |  | | 5.17 | 5.39E-23 |  |  | hypothetical protein |
| PA14_18790 | *opmE* | | 18.67 | 2.65E-23 |  |  | outer membrane efflux protein |
| PA14_19150 | *yigM* | | 3.27 | 1.06E-11 | 3.42 | 1.60E-13 | hypothetical protein |
| PA14_19230 | *ybdG* | | 9.13 | 2.38E-38 | 6.72 | 1.13E-31 | hypothetical protein |
| PA14_19390 |  | | 2.54 | 3.04E-08 |  |  | hypothetical protein |
| PA14_19650 |  | | 32.44 | 7.24E-23 |  |  | hypothetical protein |
| PA14_20000 |  | | -9.21 | 1.34E-40 | -5.24 | 4.75E-29 | transmembrane sensor |
| PA14_20070 |  | | -2.85 | 2.56E-08 | -2.72 | 4.85E-08 | hypothetical protein |
| PA14_20640 |  | | 2.72 | 2.05E-07 |  |  | hypothetical protein |
| PA14_20900 |  | |  |  | -6.06 | 2.45E-28 | MFS transporter |
| PA14_21280 |  | |  |  | -3.43 | 1.55E-17 | hypothetical protein |
| PA14_21290 |  | |  |  | -9.34 | 1.25E-25 | hypothetical protein |
| PA14_22320 |  | |  |  | 2.92 | 2.46E-07 | hypothetical protein |
| PA14_22340 | *yjcH* | | -3.55 | 2.55E-14 | 5.13 | 8.31E-30 | hypothetical protein |
| PA14_22350 | *actP* | | -2.71 | 1.33E-09 | 3.88 | 1.39E-21 | acetate permease |
| PA14_22630 |  | | -5.04 | 2.21E-05 | -4.01 | 3.27E-05 | hypothetical protein |
| PA14_22690 | *trkH* | | 4.04 | 2.28E-15 | 3.41 | 2.30E-14 | potassium uptake protein TrkH |
| PA14_23900 | *cvpA* | | 5.76 | 1.75E-27 | 3.89 | 2.15E-19 | hypothetical protein |
| PA14_24760 | *yqjE* | | -4.14 | 2.68E-19 | -8.58 | 1.63E-48 | hypothetical protein |
| PA14_25450 | *lolE* | | 4.05 | 3.73E-19 |  |  | hypothetical protein |
| PA14_26420 |  | | -2.79 | 1.85E-04 | 4.20 | 6.25E-06 | TonB-dependent receptor |
| PA14_26450 | *yebN* | |  |  | 3.59 | 1.23E-10 | hypothetical protein |
| PA14_27070 |  | | -4.92 | 3.48E-23 | -7.46 | 2.53E-40 | hypothetical protein |
| PA14_28400 | *oprQ* | | 4.01 | 9.75E-20 |  |  | outer membrane OprD family porin |
| PA14_28430 |  | |  |  | 3.14 | 5.05E-06 | hypothetical protein |
| PA14_28490 |  | | -3.86 | 1.68E-17 | -8.20 | 2.91E-46 | hypothetical protein |
| PA14_28530 |  | | -2.67 | 1.54E-09 | -3.91 | 1.05E-18 | hypothetical protein |
| PA14_29220 | *opdB* | | -4.73 | 1.08E-04 | -3.98 | 1.01E-04 | porin |
| PA14_29350 | *pfeA* | |  |  | 3.38 | 4.63E-13 | outer membrane receptor FepA |
| PA14_29660 |  | | 8.31 | 9.97E-41 |  |  | hypothetical protein |
| PA14_30410 | *yccA* | |  |  | 2.53 | 1.52E-11 | hypothetical protein |
| PA14_32720 |  | | -4.46 | 3.26E-20 |  |  | transmembrane sensor |
| PA14_33460 |  | | -8.29 | 3.08E-05 | -13.24 | 4.47E-07 | hypothetical protein |
| PA14_33600 |  | | -3.84 | 6.77E-08 | 3.07 | 2.36E-15 | hypothetical protein |
| PA14_34720 |  | | -6.29 | 6.34E-09 |  |  | hypothetical protein |
| PA14_35620 | *pslK* | | -3.78 | 1.76E-09 |  |  | hypothetical protein |
| PA14_36700 |  | | -5.69 | 1.78E-13 | -5.66 | 8.44E-14 | hypothetical protein |
| PA14_36980 |  | | -9.76 | 3.02E-34 | -5.73 | 6.97E-24 | hypothetical protein |
| PA14_38950 |  | |  |  | 2.88 | 2.06E-06 | hypothetical protein |
| PA14_39020 |  | |  |  | 3.20 | 2.42E-06 | hypothetical protein |
| PA14_39810 |  | | -3.58 | 2.43E-15 | -3.67 | 1.75E-18 | transmembrane sensor |
| PA14_40250 | *opmL* | | -44.08 | 5.61E-102 | -38.43 | 4.24E-109 | outer membrane protein |
| PA14_40540 |  | | 31.11 | 1.74E-15 | 12.23 | 1.16E-06 | hypothetical protein |
| PA14_41570^¶‡^ | *oprF* | | -2.51 | 3.36E-09 | -6.31 | 4.54E-38 | major porin and structural outer membrane porin OprF precursor |
| PA14_41630 | *cmaX* | |  |  | -3.03 | 2.78E-10 | cytoplasmic membrane-associated protein |
| PA14_41710 |  | | 3.75 | 1.60E-08 | 4.73 | 1.63E-26 | hypothetical protein |
| PA14_42910 |  | |  |  | -3.11 | 1.97E-13 | hypothetical protein |
| PA14_43650 |  | | 3.67 | 8.98E-16 | 2.54 | 8.75E-10 | hypothetical protein |
| PA14_44460 |  | | 5.12 | 8.63E-23 |  |  | hypothetical protein |
| PA14_44530 | *ygdE* | | 16.80 | 2.37E-45 | 69.34 | 3.08E-110 | multidrug efflux system protein MdtI |
| PA14_44880 |  | | -4.41 | 4.28E-20 | -4.94 | 1.15E-27 | hypothetical protein |
| PA14_46240 |  | | -2.91 | 1.38E-11 | -3.53 | 2.84E-18 | hypothetical protein |
| PA14_46650 |  | | 5.59 | 2.77E-17 | 6.29 | 9.32E-21 | transmembrane sensor |
| PA14_47040 | *yegH* | |  |  | 11.23 | 2.23E-42 | TerC family protein |
| PA14_47810 |  | | 4.00 | 3.04E-11 |  |  | hypothetical protein |
| PA14_48660 |  | | -3.31 | 1.16E-11 |  |  | hypothetical protein |
| PA14_49050 | *yohC* | | -3.25 | 2.52E-12 |  |  | hypothetical protein |
| PA14_49610 |  | |  |  | 4.21 | 1.30E-07 | MFS family transporter |
| PA14_49750 |  | |  |  | 5.99 | 9.29E-25 | MFS family transporter |
| PA14_49900 |  | | -2.65 | 1.22E-03 |  |  | hypothetical protein |
| PA14_50880 |  | | -89.77 | 1.68E-135 | -205.66 | 5.53E-190 | hypothetical protein |
| PA14_54040* |  | | 7.78 | 5.52E-35 | 15.85 | 1.55E-66 | amino acid permease |
| PA14_54520 | *opdH* | | -6.48 | 4.82E-22 | 3.04 | 3.54E-13 | porin |
| PA14_55720 |  | | 2.96 | 3.80E-06 | 2.94 | 4.88E-06 | hypothetical protein |
| PA14_55820 |  | | -36.49 | 2.30E-65 | -22.16 | 4.63E-59 | hypothetical protein |
| PA14_56050 |  | |  |  | -5.76 | 6.10E-18 | hypothetical protein |
| PA14_56100 |  | | 8.92 | 1.51E-37 | 3.54 | 2.68E-14 | hypothetical protein |
| PA14_56110 |  | | 8.83 | 6.81E-37 | 3.52 | 1.98E-08 | hypothetical protein |
| PA14_56810 |  | | -5.46 | 6.01E-04 | -4.96 | 2.79E-04 | lemA-like protein |
| PA14_56880 |  | | 3.38 | 5.61E-14 | 2.63 | 1.51E-10 | membrane fusion protein |
| PA14_57030 | *fxsA* | | 7.66 | 2.18E-36 | 3.32 | 1.63E-15 | FxsA protein |
| PA14_57880 |  | | 2.82 | 2.18E-11 |  |  | ABC transporter ATP-binding protein |
| PA14_58500 |  | |  |  | 7.26 | 8.49E-22 | hypothetical protein |
| PA14_60390 | *mviN* | | 2.85 | 1.38E-10 |  |  | virulence factor, membrane protein |
| PA14_60530 |  | | 5.11 | 5.31E-20 | 5.15 | 7.95E-22 | hypothetical protein |
| PA14_60630 |  | |  |  | 4.03 | 5.80E-21 | hypothetical protein |
| PA14_60730 |  | |  |  | -4.38 | 7.49E-17 | outer membrane protein |
| PA14_61050^‡^ | *mscL* | | -10.69 | 1.02E-28 | -17.63 | 2.38E-42 | large-conductance mechanosensitive channel |
| PA14_61170 |  | | 17.31 | 3.01E-24 | 2.85 | 2.10E-04 | transmembrane protein |
| PA14_61260 |  | | 16.50 | 2.14E-36 | 9.68 | 6.94E-25 | hypothetical protein |
| PA14_63720 |  | |  |  | 2.71 | 1.00E-01 | hypothetical protein |
| PA14_63910 |  | | -12.88 | 1.71E-51 |  |  | hypothetical protein |
| PA14_63960 |  | | -26.37 | 5.71E-82 |  |  | uter membrane protein |
| PA14_63970 |  | | -9.78 | 1.30E-15 |  |  | hypothetical protein |
| PA14_64480 | *osmE* | | -11.14 | 2.56E-36 | -27.96 | 1.02E-62 | DNA-binding transcriptional activator OsmE |
| PA14_64510 | *yhjG* | |  |  | -5.56 | 8.90E-33 | hypothetical protein |
| PA14_64690 |  | | -4.14 | 1.58E-18 | -2.83 | 4.79E-13 | transmembrane sensor |
| PA14_65030 |  | |  |  | -2.65 | 1.19E-11 | hypothetical protein |
| PA14_65040 |  | | -33.20 | 2.46E-59 | -23.88 | 2.62E-57 | hypothetical protein |
| PA14_65160 |  | | 11.56 | 7.93E-53 | 7.77 | 6.94E-44 | hypothetical protein |
| PA14_65580 | *ybcI* | | 5.22 | 4.30E-21 |  |  | membrane-bound metal-dependent hydrolase |
| PA14_66420 | *ytnM* | |  |  | 4.08 | 5.40E-20 | hypothetical protein |
| PA14_67780 |  | | 7.70 | 2.15E-14 |  |  | hypothetical protein |
| PA14_68050 |  | | 3.14 | 2.41E-09 | 3.04 | 2.49E-10 | hypothetical protein |
| PA14_68280 |  | | 4.38 | 3.67E-19 |  |  | dicarboxylate transporter |
| PA14_68290 |  | | 3.70 | 1.59E-16 |  |  | C4-dicarboxylate transporter |
| PA14_69090 | *yhiI* | |  |  | -14.48 | 3.43E-25 | hypothetical protein |
| PA14_69250 |  | | 8.29 | 8.29E-39 | 4.42 | 8.44E-23 | hypothetical protein |
| PA14_71100 | *yeiH* | | -3.64 | 2.05E-09 | 16.86 | 4.38E-19 | hypothetical protein |
| PA14_71390 |  | |  |  | -3.35 | 2.00E-17 | hypothetical protein |
| PA14_71590 |  | | -4.87 | 2.75E-23 | -14.41 | 1.82E-68 | hypothetical protein |
| PA14_72300 |  | |  |  | -2.59 | 5.04E-10 | hypothetical protein |
| PA14_72320 |  | | 2.76 | 6.10E-10 |  |  | hypothetical protein |
| PA14_72790 |  | | 3.31 | 4.61E-13 |  |  | hypothetical protein |
| PA14_73410* | *yidC* | | 13.19 | 2.38E-57 | 10.64 | 2.15E-55 | inner membrane protein translocase component YidC |
| **Motility & Attachment** | | |  |  |  |  |  |
| PA14_05190* | *pilU* | |  |  | -5.89 | 6.63E-35 | twitching motility protein PilU |
| PA14_11080 | *cupB3* | | -3.49 | 5.39E-12 |  |  | usher CupB3 |
| PA14_14850 | *pilF* | | 3.04 | 2.38E-13 |  |  | type 4 fimbrial biogenesis protein PilF |
| PA14_20610 | *lecB* | | -6.83 | 2.27E-22 | 4.67 | 2.61E-28 | fucose-binding lectin PA-IIL |
| PA14_23830 | *fimV* | |  |  | -6.52 | 2.90E-39 | pilus assembly protein |
| PA14_31290 | *pa1L* | | -344.39 | 5.76E-181 | -77.30 | 1.21E-136 | PA-I galactophilic lectin |
| PA14_33530 |  | | -4.93 | 5.60E-20 | 4.36 | 3.67E-25 | hypothetical protein |
| PA14_33560 |  | | -3.01 | 1.73E-11 | 3.53 | 4.75E-19 | adhesion protein |
| PA14_45410 |  | |  |  | -3.14 | 3.47E-12 | FlhB domain-containing protein |
| PA14_45680 | *flhA* | | 3.40 | 1.33E-15 |  |  | flagellar biosynthesis protein FlhA |
| PA14_45790 | *fliN* | |  |  | -3.12 | 4.32E-15 | flagellar motor switch protein |
| PA14_45830 |  | |  |  | -13.82 | 4.79E-24 | hypothetical protein |
| PA14_50140 | *fliF* | | 3.14 | 1.27E-13 |  |  | flagellar MS-ring protein |
| PA14_50160 | *fliE* | | 2.78 | 8.06E-11 |  |  | flagellar hook-basal body protein FliE |
| PA14_50270 | *fliD* | |  |  | -4.82 | 2.37E-25 | flagellar capping protein FliD |
| PA14_50360 | *flgK* | |  |  | -3.46 | 8.86E-19 | flagellar hook-associated protein FlgK |
| PA14_50420 | *flgH* | |  |  | -3.70 | 3.27E-06 | flagellar basal body L-ring protein |
| PA14_50430 | *flgG* | |  |  | -7.43 | 3.42E-19 | flagellar basal body rod protein FlgG |
| PA14_50440 | *flgF* | |  |  | -11.55 | 1.08E-56 | flagellar basal body rod protein FlgF |
| PA14_50450 | *flgE* | |  |  | -9.95 | 1.07E-53 | flagellar hook protein FlgE |
| PA14_50460 | *flgD* | |  |  | -13.04 | 1.45E-26 | flagellar basal body rod modification protein |
| PA14_50470 | *flgC* | |  |  | -10.00 | 6.68E-46 | flagellar basal body rod protein FlgC |
| PA14_50480 | *flgB* | |  |  | -4.04 | 6.57E-17 | flagellar basal body rod protein FlgB |
| PA14_51450 | *cupC3* | | -7.00 | 4.90E-33 |  |  | usher CupC3 |
| PA14_51470 | *cupC1* | | -2.60 | 9.57E-04 |  |  | fimbrial subunit CupC1 |
| PA14_58730 | *pilA* | |  |  | -4.47 | 3.51E-15 | type IV pilin structural subunit |
| PA14_58770 | *pilD* | |  |  | -2.54 | 6.51E-11 | type 4 prepilin peptidase PilD |
| PA14_59280 | *pilP2* | |  |  | -4.60 | 1.25E-08 | type IV B pilus protein |
| PA14_59290 | *pilQ2* | | -3.20 | 2.69E-09 | -2.65 | 2.14E-07 | type IV B pilus protein |
| PA14_59310 | *pilR2* | | -2.71 | 1.04E-04 |  |  | type IV B pilus protein |
| PA14_59320 | *pilS2* | | -4.09 | 6.90E-14 | -4.37 | 1.46E-15 | type IV B pilus protein |
| PA14_59340 | *pilT2* | | -5.92 | 4.63E-24 | -6.55 | 1.22E-28 | type IV B pilus protein |
| PA14_59710 | *cupD1* | |  |  | -2.51 | 3.19E-06 | fimbrial protein |
| PA14_60280 | *fimU* | | -2.63 | 7.14E-04 |  |  | type 4 fimbrial biogenesis protein FimU |
| PA14_61530 | *csuC* | | -13.53 | 2.05E-13 | -30.05 | 5.24E-20 | pili assembly chaperone |
| PA14_61540 | *csuD* | | -8.33 | 1.10E-30 | -12.56 | 7.98E-41 | hypothetical protein |
| PA14_66620 | *pilQ* | |  |  | -2.70 | 3.86E-06 | type 4 fimbrial biogenesis outer membrane protein PilQ precursor |
| PA14_66630 | *pilP* | |  |  | -3.24 | 1.93E-16 | type 4 fimbrial biogenesis protein PilP |
| PA14_66650 | *pilN* | |  |  | -2.73 | 1.51E-12 | type 4 fimbrial biogenesis protein PilN |
| PA14_69100 |  | |  |  | -2.54 | 5.21E-08 | flagellar basal body-associated protein FliL-like protein |
| PA14_69760 |  | | 6.06 | 6.71E-16 | 2.67 | 1.20E-04 | fimbrial protein |
| **Nucleotide biosynthesis and metabolism** | | | |  |  |  |  |
| PA14_00800 |  | |  |  | -2.79 | 1.72E-12 | hypothetical protein |
| PA14_01780 |  | | -3.34 | 6.18E-08 | -4.25 | 5.12E-11 | nucleoside 2-deoxyribosyltransferase |
| PA14_04480 | *thyA* | | 4.30 | 1.32E-19 | 2.57 | 3.58E-10 | thymidylate synthase |
| PA14_05050 |  | | -3.24 | 4.85E-14 | -4.23 | 1.53E-24 | deoxyribonucleotide triphosphate pyrophosphatase |
| PA14_05250 | *pyrC* | | 2.80 | 3.01E-11 |  |  | dihydroorotase |
| PA14_05700 | *codB* | | 6.38 | 2.04E-17 |  |  | cytosine permease |
| PA14_05770 | *dhT* | |  |  | 4.17 | 9.49E-20 | phenylhydantoinase |
| PA14_05790 |  | |  |  | 2.94 | 1.54E-06 | transporter |
| PA14_06360 | *comF* | | 3.28 | 2.90E-11 |  |  | phosphoribosyl transferase |
| PA14_08420 |  | | -17.94 | 2.20E-64 | -11.22 | 1.13E-55 | HIT family protein |
| PA14_15310* | *guaB* | | 4.55 | 3.47E-22 |  |  | inosine 5'-monophosphate dehydrogenase |
| PA14_15340* | *guaA* | | 5.78 | 1.24E-29 | 2.64 | 4.94E-12 | GMP synthase |
| PA14_15680 | *cumB* | |  |  | -3.10 | 5.98E-15 | hypothetical protein |
| PA14_15740* | *purL* | | 5.46 | 1.76E-28 | 2.98 | 3.25E-15 | phosphoribosylformylglycinamidine synthase |
| PA14_15850 |  | |  |  | 3.16 | 1.12E-15 | hypothetical protein |
| PA14_15890* | *purT* | | 3.22 | 1.09E-13 |  |  | phosphoribosylglycinamide formyltransferase 2 |
| PA14_16700* | *adk* | | 3.77 | 6.08E-18 |  |  | adenylate kinase |
| PA14_17080 | *pyrH* | | 4.38 | 3.65E-08 | 2.75 | 6.65E-13 | uridylate kinase |
| PA14_17290* | *pyrG* | | 5.40 | 2.96E-27 |  |  | CTP synthetase |
| PA14_18710 | *pyrC* | | 3.95 | 1.21E-14 |  |  | dihydroorotase |
| PA14_19090 | *dcd* | | 3.15 | 4.71E-13 |  |  | deoxycytidine triphosphate deaminase |
| PA14_21440 |  | |  |  | 2.57 | 3.45E-10 | HIT family protein |
| PA14_24640 | *pyrD* | | 7.42 | 1.23E-34 | 4.04 | 6.48E-20 | dihydroorotate dehydrogenase 2 |
| PA14_25390* | *sth* | |  |  | 2.86 | 3.73E-14 | soluble pyridine nucleotide transhydrogenase |
| PA14_25740 | *tmk* | | 3.34 | 2.99E-14 |  |  | thymidylate kinase |
| PA14_26890 | *pyrF* | | 5.55 | 1.30E-21 | 3.59 | 1.06E-12 | orotidine 5'-phosphate decarboxylase |
| PA14_32340 |  | |  |  | -4.07 | 1.30E-07 | deaminase |
| PA14_39690* | *nrdD* | |  |  | -2.60 | 2.85E-04 | anaerobic ribonucleoside triphosphate reductase |
| PA14_41430 | *miaE* | | 3.56 | 7.40E-13 |  |  | hypothetical protein |
| PA14_44500 | *apt* | | 4.02 | 1.99E-16 | 4.37 | 4.14E-21 | adenine phosphoribosyltransferase |
| PA14_44710 | *xdhA* | | -8.81 | 2.43E-18 |  |  | xanthine dehydrogenase |
| PA14_44740 | *xdhB* | | -6.01 | 6.85E-29 | -15.34 | 4.07E-69 | xanthine dehydrogenase |
| PA14_44770 | *gda1* | |  |  | -3.29 | 8.29E-15 | guanine deaminase |
| PA14_49460* | *nrdA* | | 3.46 | 2.17E-16 | 3.89 | 2.63E-22 | ribonucleotide-diphosphate reductase subunit alpha |
| PA14_49470* | *nrdB* | | 3.68 | 7.17E-18 | 5.33 | 1.51E-18 | ribonucleotide-diphosphate reductase subunit beta |
| PA14_52040* | *purM* | | 2.66 | 8.22E-10 |  |  | phosphoribosylaminoimidazole synthetase |
| PA14_53290 | *trxB2* | | -10.50 | 4.96E-43 | -5.72 | 1.48E-31 | thioredoxin reductase 2 |
| PA14_61470 | *upp* | |  |  | -3.05 | 2.94E-02 | uracil phosphoribosyltransferase |
| PA14_61770* | *prs* | | 35.92 | 1.03E-94 | 9.65 | 1.11E-49 | ribose-phosphate pyrophosphokinase |
| PA14_62910* | *carB* | | 3.14 | 2.88E-14 | 3.38 | 7.39E-10 | carbamoyl phosphate synthase large subunit |
| PA14_62930* | *carA* | | 3.39 | 1.20E-15 | 3.15 | 5.28E-09 | carbamoyl phosphate synthase small subunit |
| PA14_63390 |  | | 3.19 | 3.01E-10 |  |  | adenylate kinase |
| PA14_64200* | *purH* | | 7.34 | 5.01E-28 | 6.18 | 3.23E-33 | bifunctional phosphoribosylaminoimidazolecarboxamide formyltransferase/IMP cyclohydrolase |
| PA14_64220 | *purD* | | 7.58 | 9.06E-27 | 5.52 | 1.73E-26 | phosphoribosylamine--glycine ligase |
| PA14_64930* |  | |  |  | -20.14 | 3.11E-80 | hypothetical protein |
| PA14_67740* | *grx* | | 8.54 | 2.05E-36 | 3.42 | 9.59E-14 | glutaredoxin |
| PA14_69220 | *ppx* | | 2.74 | 9.27E-11 |  |  | exopolyphosphatase |
| PA14_69940 | *xpt* | | 5.21 | 3.86E-23 | 5.60 | 6.62E-28 | xanthine phosphoribosyltransferase |
| PA14_70260 | *dut* | | 6.38 | 2.78E-27 |  |  | deoxyuridine 5'-triphosphate nucleotidohydrolase |
| PA14_70440 | *gmk* | | 10.54 | 5.61E-46 | 2.89 | 1.01E-11 | guanylate kinase |
| PA14_71600 | *purK* | | 9.50 | 1.38E-41 | 2.75 | 2.34E-10 | phosphoribosylaminoimidazole carboxylase ATPase subunit |
| PA14_71620 | *purE* | | 15.26 | 1.38E-54 | 5.01 | 4.05E-20 | phosphoribosylaminoimidazole carboxylase catalytic subunit |
| PA14_72540^‡^* |  | |  |  | -4.86 | 3.85E-20 | ribonucleotide reductase |
| PA14_73070 | *pyrQ* | | -70.77 | 2.47E-122 | -9.54 | 4.56E-53 | dihydroorotase |
| **Protein secretion/export apparatus** | | | |  |  |  |  |
| PA14_00490 |  | | 16.19 | 6.79E-51 | 10.60 | 7.95E-39 | hemolysin activation/secretion protein |
| PA14_01040 |  | | 6.29 | 3.79E-15 | 3.05 | 1.71E-13 | secretion protein |
| PA14_01940 |  | |  |  | -3.95 | 4.56E-20 | RND efflux membrane fusion protein |
| PA14_04870 |  | | 3.91 | 1.69E-18 |  |  | hypothetical protein |
| PA14_04900 | *ftsY* | | 5.75 | 1.49E-28 | 2.51 | 4.23E-10 | signal recognition particle receptor FtsY |
| PA14_07040 | *yqjC* | | 4.00 | 6.65E-18 |  |  | hypothetical protein |
| PA14_08695 | *secE* | | 10.47 | 8.53E-50 | 3.63 | 2.00E-14 | preprotein translocase subunit SecE |
| PA14_09050 | *secY* | | 3.42 | 9.33E-17 | 4.49 | 2.19E-27 | preprotein translocase subunit SecY |
| PA14_10330 | *ompK* | | -18.70 | 1.52E-16 | -5.40 | 7.46E-10 | outer membrane protein |
| PA14_10340 | *cvaB* | | -12.16 | 1.35E-50 | -3.69 | 5.68E-20 | toxin transporter |
| PA14_10350 |  | | -36.32 | 1.75E-80 | -6.06 | 6.79E-36 | secretion protein |
| PA14_13520 |  | | 2.77 | 2.18E-08 |  |  | outer membrane protein |
| PA14_13530 |  | | 7.60 | 2.25E-26 |  |  | outer membrane protein |
| PA14_14230 |  | | -2.75 | 2.66E-07 | -5.22 | 9.41E-15 | hypothetical protein |
| PA14_14610 | *yajC* | | 7.38 | 2.16E-35 | 3.84 | 2.23E-19 | preprotein translocase subunit YajC |
| PA14_14630 | *secD* | | 9.78 | 3.20E-21 | 3.22 | 2.81E-16 | preprotein translocase subunit SecD |
| PA14_14650 | *secF* | | 2.95 | 4.66E-07 |  |  | preprotein translocase subunit SecF |
| PA14_14660* | *ycfJ* | | -5.71 | 2.17E-28 | -6.53 | 1.55E-39 | hypothetical protein |
| PA14_15960 | *ffh* | | 5.66 | 1.49E-21 | 2.51 | 1.89E-10 | signal recognition particle protein Ffh |
| PA14_17510 |  | |  |  | 3.31 | 3.52E-11 | hypothetical protein |
| PA14_19680 |  | | 5.15 | 4.45E-22 |  |  | hypothetical protein |
| PA14_20030 | *hasD* | | -57.49 | 3.19E-77 | -15.29 | 2.26E-51 | transport protein HasD |
| PA14_20040 | *hasE* | | -89.25 | 1.35E-118 | -16.62 | 1.71E-67 | metalloprotease secretion protein |
| PA14_20050 | *hasF* | | -77.28 | 1.06E-82 | -12.83 | 9.76E-46 | outer membrane protein |
| PA14_20860 |  | | -3.21 | 7.64E-06 | -5.71 | 2.76E-10 | Tfp pilus assembly protein FimV |
| PA14_23980 | *xcpP* | |  |  | 2.93 | 1.20E-12 | secretion protein XcpP |
| PA14_24020 | *xcpT* | | -3.13 | 3.71E-11 | -3.15 | 2.35E-13 | general secretion pathway protein G |
| PA14_24040 | *xcpU* | |  |  | -3.16 | 1.44E-15 | general secretion pathway outer membrane protein H precursor |
| PA14_24050 | *xcpV* | |  |  | -2.80 | 2.39E-12 | general secretion pathway protein I |
| PA14_24080 | *xcpY* | | -2.57 | 2.26E-09 | -3.08 | 3.11E-15 | general secretion pathway protein L |
| PA14_24100 | *xcpZ* | | -3.91 | 5.96E-17 | -4.35 | 6.50E-23 | general secretion pathway protein M |
| PA14_30070 |  | | -3.23 | 1.00E-13 | -2.62 | 5.52E-12 | hypothetical protein |
| PA14_31920 | *opmB* | |  |  | -4.65 | 9.59E-23 | outer membrane protein |
| PA14_40170 | *sugE* | | 4.89 | 4.72E-10 | 11.36 | 1.85E-27 | transporter |
| PA14_40230 |  | | -51.74 | 2.11E-106 | -32.95 | 3.52E-99 | secretion protein |
| PA14_40240 |  | | -35.43 | 5.72E-92 | -34.42 | 4.36E-102 | ABC transporter ATP-binding protein/permease |
| PA14_42290 | *pscH* | |  |  | -2.90 | 1.00E-05 | type III export protein PscH |
| PA14_42300 | *pscG* | |  |  | -2.73 | 1.71E-07 | type III export protein PscG |
| PA14_42310 | *pscF* | |  |  | -3.13 | 1.39E-09 | type III export protein PscF |
| PA14_42320 | *pscE* | |  |  | -3.28 | 3.22E-07 | type III export protein PscE |
| PA14_42400 | *exsB* | |  |  | -4.43 | 1.81E-19 | exoenzyme S synthesis protein B |
| PA14_42440 | *popD* | | -11.10 | 3.70E-47 | -24.93 | 2.69E-84 | translocator outer membrane protein PopD precursor |
| PA14_42450 | *popB* | | -10.97 | 1.46E-46 | -21.63 | 6.50E-79 | translocator protein PopB |
| PA14_42460* | *pcrH* | | -5.76 | 1.34E-23 | -18.29 | 9.85E-54 | regulatory protein PcrH |
| PA14_42470 | *pcrV* | | -4.61 | 6.21E-20 | -7.96 | 3.92E-36 | type III secretion protein PcrV |
| PA14_42480 | *pcrG* | | -2.73 | 9.24E-07 | -5.99 | 6.95E-15 | regulator in type III secretion |
| PA14_42530 | *pcr2* | |  |  | -2.60 | 6.83E-04 | type III secretion protein |
| PA14_42550 | *popN* | |  |  | -4.16 | 3.65E-08 | Type III secretion outer membrane protein PopN precursor |
| PA14_42610 | *pscQ* | |  |  | -4.29 | 8.36E-05 | type III secretion system protein |
| PA14_48090 | *aprF* | | -9.37 | 7.92E-43 | -3.91 | 3.43E-22 | alkaline protease secretion outer membrane protein AprF precursor |
| PA14_48115 | *aprD* | | -8.20 | 1.22E-20 | -3.98 | 3.64E-12 | alkaline protease secretion protein AprD |
| PA14_54370* | *lepA* | | 4.63 | 2.64E-23 |  |  | GTP-binding protein LepA |
| PA14_55530 | *hxcW* | | -2.57 | 1.02E-07 |  |  | type II secretion system protein |
| PA14_55790 |  | | -86.23 | 3.34E-16 | -24.91 | 6.47E-12 | hypothetical protein |
| PA14_55800 |  | | -6.36 | 2.79E-09 |  |  | hypothetical protein |
| PA14_55850 |  | | -69.89 | 2.28E-119 | -112.54 | 6.61E-147 | pilus assembly protein |
| PA14_55860 |  | | -37.14 | 3.28E-92 | -60.15 | 1.52E-119 | hypothetical protein |
| PA14_55880 |  | | -38.55 | 6.08E-24 | -78.82 | 1.89E-32 | hypothetical protein |
| PA14_55890 | *cpaF2* | | -27.65 | 1.56E-58 | -69.17 | 1.18E-85 | type II secretion system protein |
| PA14_55900 |  | | -27.56 | 2.32E-21 | -55.85 | 7.36E-30 | hypothetical protein |
| PA14_55920 |  | | -35.43 | 8.64E-93 | -46.01 | 4.86E-116 | type II secretion system protein |
| PA14_55930 |  | | -53.06 | 9.27E-110 | -43.23 | 4.23E-114 | pilus assembly protein |
| PA14_55940 |  | | -481.77 | 1.89E-69 | -99.87 | 7.56E-51 | hypothetical protein |
| PA14_57220^‡^ | *secA* | | 3.02 | 5.04E-13 |  |  | preprotein translocase subunit SecA |
| PA14_59860 |  | |  |  | -3.90 | 1.15E-02 | type III effector Hop protein |
| PA14_61190 |  | | -24.53 | 1.44E-78 | -13.77 | 6.57E-67 | hypothetical protein |
| PA14_65750 | *ompH* | | 3.32 | 3.99E-14 |  |  | outer membrane efflux protein |
| PA14_66980 | *tatC* | | 3.53 | 8.76E-16 | 2.98 | 6.01E-14 | sec-independent protein translocase TatC |
| PA14_67720* | *secB* | | 5.14 | 8.22E-26 | 4.06 | 2.66E-22 | preprotein translocase subunit SecB |
| PA14_68820 | *gspE* | | -3.28 | 1.81E-10 | -4.02 | 1.29E-14 | secretion pathway ATPase |
| PA14_70740 |  | | -22.25 | 2.50E-39 | -21.77 | 2.40E-43 | hypothetical protein |
| **Putative enzymes** | | |  |  |  |  |  |
| PA14_00640 | *phzH* | | -4.05 | 7.76E-16 |  |  | potential phenazine-modifying enzyme |
| PA14_01490 |  | | -31.07 | 1.65E-89 |  |  | hemolysin |
| PA14_01750 |  | | 7.90 | 7.85E-35 | 4.96 | 3.26E-23 | hydroxydechloroatrazine ethylaminohydrolase |
| PA14_02530 |  | | 10.47 | 9.61E-46 | 34.43 | 2.81E-102 | hypothetical protein |
| PA14_02930 |  | | 2.71 | 1.24E-05 |  |  | oxidoreductase |
| PA14_04890 |  | | 3.42 | 1.19E-14 |  |  | zinc protease |
| PA14_05400 |  | |  |  | -3.48 | 1.90E-17 | methylesterase |
| PA14_06270* | *catD* | | -5.66 | 1.86E-08 | -2.85 | 1.01E-03 | hydrolase |
| PA14_06300 |  | | -7.66 | 7.94E-32 | -2.87 | 2.92E-12 | GNAT family acetyltransferase |
| PA14_07250 |  | | -8.27 | 4.17E-28 |  |  | hypothetical protein |
| PA14_07290 | *ygjP* | | 3.08 | 4.00E-11 |  |  | hypothetical protein |
| PA14_07310 |  | | 6.75 | 5.57E-24 | 3.49 | 3.42E-06 | hydrolase |
| PA14_08160 | *lys* | | -3.88 | 4.67E-15 | -12.85 | 8.28E-43 | lytic enzyme |
| PA14_09400* | *phzS* | | -12.37 | 3.05E-53 | -14.96 | 5.67E-72 | hypothetical protein |
| PA14_09580* |  | |  |  | 3.37 | 7.03E-17 | hypothetical protein |
| PA14_09900 | *prpL* | | -59.72 | 2.51E-47 | -46.86 | 1.01E-47 | Pvds-regulated endoprotease, lysyl class |
| PA14_09940 | *pfpI* | | -4.63 | 3.22E-18 |  |  | protease |
| PA14_09950 |  | | -15.47 | 4.63E-59 |  |  | oxidoreductase |
| PA14_10730* |  | |  |  | -3.86 | 4.02E-22 | hypothetical protein |
| PA14_10740 |  | | 2.97 | 1.85E-11 |  |  | spermidine acetyltransferase |
| PA14_11130* |  | | -3.92 | 1.66E-17 |  |  | short chain dehydrogenase |
| PA14_11580 |  | | -8.48 | 5.81E-21 | -6.89 | 2.77E-21 | hypothetical protein |
| PA14_11810 |  | | -5.69 | 2.02E-27 | -61.61 | 1.40E-122 | aldehyde dehydrogenase |
| PA14_12160 |  | | 5.79 | 3.09E-28 |  |  | murein transglycosylase |
| PA14_13330 |  | | 6.36 | 5.78E-29 | 14.56 | 2.60E-24 | hypothetical protein |
| PA14_14270 |  | | -9.59 | 3.05E-44 | -6.65 | 1.77E-39 | isochorismatase family hydrolase |
| PA14_16390* |  | | -2.93 | 1.00E-09 |  |  | GMC-type oxidoreductase |
| PA14_17410 |  | |  |  | -3.90 | 1.16E-08 | esterase |
| PA14_17550 |  | | -3.86 | 6.98E-18 | -5.03 | 4.90E-30 | hypothetical protein |
| PA14_18300 |  | | -2.55 | 8.81E-06 |  |  | nucleotide sugar dehydrogenase |
| PA14_18630 | *eprS* | |  |  | -2.97 | 1.89E-10 | serine protease |
| PA14_19360 |  | |  |  | -24.19 | 7.47E-91 | GNAT family acetyltransferase |
| PA14_19640 | *ydgB* | | 3.87 | 2.26E-17 |  |  | short chain dehydrogenase |
| PA14_19730 |  | | -4.20 | 1.83E-03 | -5.72 | 1.34E-04 | oxidoreductase |
| PA14_20960 |  | |  |  | -4.47 | 8.43E-23 | isomerase |
| PA14_20980 |  | |  |  | -3.22 | 6.85E-16 | short chain dehydrogenas |
| PA14_21040 |  | |  |  | -2.88 | 2.27E-08 | hypothetical protein |
| PA14_21050 |  | | -2.72 | 4.00E-09 | -6.01 | 5.01E-26 | short chain dehydrogenase |
| PA14_22460* |  | |  |  | -4.29 | 6.32E-16 | alpha/beta hydrolase |
| PA14_22620 | *cyaB* | | 3.00 | 4.92E-12 | 3.61 | 3.45E-18 | hypothetical protein |
| PA14_23050* |  | |  |  | -3.49 | 3.72E-19 | hypothetical protein |
| PA14_23200 | *yciK* | | 2.80 | 9.51E-11 |  |  | short chain dehydrogenase |
| PA14_23650 | *ygfF* | | -3.11 | 2.22E-12 | -3.82 | 1.69E-17 | short chain dehydrogenase |
| PA14_24170 | *fadH1* | | 2.81 | 8.13E-10 |  |  | 2,4-dienoyl-CoA reductase |
| PA14_24810 |  | |  |  | 2.84 | 4.48E-07 | hypothetical protein |
| PA14_24820 |  | |  |  | 9.84 | 6.52E-33 | hypothetical protein |
| PA14_25195 | *nagZ* | | 3.81 | 4.91E-15 |  |  | beta-hexosaminidase |
| PA14_25730 |  | | 4.50 | 1.33E-21 |  |  | hypothetical protein |
| PA14_25900* |  | | 2.98 | 7.32E-07 |  |  | trans-2-enoyl-CoA reductase |
| PA14_26090 |  | | 13.85 | 4.28E-08 | 6.24 | 3.59E-27 | hydrolase |
| PA14_26310 |  | | -5.29 | 2.28E-15 | -3.18 | 8.22E-09 | short chain dehydrogenase |
| PA14_26350 |  | | -7.84 | 1.10E-36 |  |  | hypothetical protein |
| PA14_26485 |  | | 4.41 | 2.45E-19 | 3.72 | 2.70E-17 | oxidoreductase |
| PA14_26650 |  | | -2.61 | 6.70E-08 | -5.67 | 2.07E-21 | short chain dehydrogenase |
| PA14_26730 |  | |  |  | -3.15 | 1.06E-05 | short-chain dehydrogenase |
| PA14_26910 | *moxR* | | -3.02 | 2.23E-08 | -3.04 | 1.25E-10 | hypothetical protein |
| PA14_27470 |  | | 4.73 | 1.90E-18 | 5.24 | 5.71E-23 | zinc carboxypeptidase |
| PA14_27560 |  | | 2.57 | 6.51E-09 |  |  | hypothetical protein |
| PA14_28340 |  | | 2.60 | 4.47E-09 | 3.24 | 7.90E-15 | hypothetical protein |
| PA14_28630 |  | |  |  | 4.00 | 3.69E-18 | hydrolase |
| PA14_29230 |  | | -15.14 | 4.35E-57 | -19.57 | 3.59E-74 | hypothetical protein |
| PA14_29240 |  | | -4.02 | 2.45E-17 | -5.62 | 8.13E-29 | hydrolase |
| PA14_30440 |  | |  |  | 2.92 | 1.39E-11 | hypothetical protein |
| PA14_30500 |  | |  |  | 2.89 | 3.30E-10 | acyl-CoA dehydrogenase |
| PA14_31370 | *tam* | | -10.12 | 2.75E-43 | -3.07 | 6.27E-15 | hypothetical protein |
| PA14_31730 |  | | 4.03 | 4.10E-16 |  |  | hypothetical protein |
| PA14_33420 |  | | -4.63 | 4.46E-08 | -3.94 | 1.71E-08 | hydrolase |
| PA14_33610 |  | | -58.97 | 7.22E-117 | -4.74 | 2.09E-28 | peptide synthase |
| PA14_34200 | *msuC* | |  |  | 5.38 | 2.15E-07 | FMNH2-dependent monooxygenase |
| PA14_34300 |  | |  |  | 9.13 | 2.23E-27 | DszC family monooxygenase |
| PA14_34320 |  | |  |  | 10.91 | 9.64E-21 | DszC family monooxygenase |
| PA14_34540 |  | | -9.29 | 8.48E-37 |  |  | xenobiotic compound DszA family monooxygenase |
| PA14_34580 |  | | 25.83 | 1.13E-15 | 3.63 | 2.24E-03 | hypothetical protein |
| PA14_34670 |  | | -3.51 | 2.91E-11 | -3.50 | 8.89E-12 | cupin superfamily protein |
| PA14_34840* |  | | -9.86 | 8.32E-45 |  |  | non-ribosomal peptide synthetase |
| PA14_35050 |  | |  |  | 3.59 | 3.34E-09 | hypothetical protein |
| PA14_35880 |  | | -3.08 | 4.07E-12 | -5.14 | 3.74E-27 | gamma-aminobutyraldehyde dehydrogenase |
| PA14_35890 |  | | -2.78 | 1.92E-10 | -8.63 | 1.46E-41 | diaminobutyrate--2-oxoglutarate aminotransferase |
| PA14_35900 |  | |  |  | -3.87 | 2.77E-17 | dehydrogenase |
| PA14_36540 |  | | -4.65 | 1.47E-13 | -3.35 | 2.34E-09 | hypothetical protein |
| PA14_36580 | *glgB* | | -7.05 | 6.80E-29 | -11.60 | 8.35E-44 | glycosyl hydrolase |
| PA14_36605 |  | | -12.44 | 4.85E-48 | -13.92 | 3.38E-57 | maltooligosyl trehalose synthase |
| PA14_36680 |  | | -4.17 | 1.82E-09 | -6.41 | 2.27E-12 | hypothetical protein |
| PA14_36740 |  | | -4.27 | 1.33E-08 | -9.09 | 1.26E-15 | hypothetical protein |
| PA14_37530 |  | | -2.82 | 1.13E-06 | 10.40 | 5.24E-11 | hydrolase |
| PA14_37745* |  | | -22.54 | 9.40E-31 | 2.93 | 1.99E-14 | carbamoyl transferase |
| PA14_39860 |  | | -4.32 | 9.50E-06 |  |  | hypothetical protein |
| PA14_40030* |  | | -5.94 | 7.27E-19 | -4.47 | 2.21E-14 | hypothetical protein |
| PA14_40180 |  | | -4.13 | 2.96E-04 |  |  | oxidoreductase |
| PA14_40200 |  | | -6.63 | 1.45E-13 | -6.79 | 7.06E-16 | oxidoreductase |
| PA14_40840 | *sohB* | | -3.65 | 3.02E-16 |  |  | periplasmic protease |
| PA14_40890* |  | |  |  | -3.30 | 1.46E-16 | short chain dehydrogenase |
| PA14_41500 |  | | -54.63 | 8.08E-106 | -18.00 | 1.75E-73 | lyase |
| PA14_41780 |  | |  |  | -4.00 | 2.27E-12 | hypothetical protein |
| PA14_42050 |  | |  |  | 2.66 | 1.14E-06 | oxidoreductase |
| PA14_42870 |  | | -2.94 | 3.84E-11 |  |  | hypothetical protein |
| PA14_43180 |  | |  |  | -3.63 | 5.37E-08 | short chain dehydrogenase |
| PA14_43190 |  | | -3.30 | 1.47E-03 | -2.76 | 1.75E-03 | oxidoreductase |
| PA14_43630 |  | |  |  | -2.83 | 3.63E-08 | lipase |
| PA14_43790 |  | | 8.34 | 3.50E-22 | 5.07 | 3.62E-24 | aldehyde dehydrogenase |
| PA14_43840 |  | |  |  | -7.77 | 1.23E-38 | hypothetical protein |
| PA14_44090 |  | | 4.78 | 2.20E-10 | 3.87 | 1.61E-07 | Fe-S-cluster oxidoreductase |
| PA14_44260 |  | | 2.98 | 4.83E-08 |  |  | oxidoreductase |
| PA14_44560 |  | |  |  | 10.21 | 2.77E-46 | flavin-containing monooxygenase |
| PA14_44570 |  | |  |  | 2.94 | 1.15E-10 | short-chain dehydrogenase |
| PA14_44590 |  | | 3.21 | 1.64E-12 |  |  | acyl-CoA dehydrogenase |
| PA14_44960 |  | | 5.28 | 1.19E-19 |  |  | hypothetical protein |
| PA14_45020 | *glxR* | | -7.62 | 1.42E-30 |  |  | oxidoreductase |
| PA14_45430 |  | | -6.99 | 2.36E-18 | -6.32 | 8.90E-20 | short chain dehydrogenase |
| PA14_46150 |  | | -27.61 | 4.56E-69 | -10.74 | 2.70E-49 | hypothetical protein |
| PA14_46250 |  | |  |  | -6.11 | 2.10E-25 | hypothetical protein |
| PA14_46550 |  | | -4.54 | 1.10E-20 | -3.95 | 3.32E-21 | ribonuclease |
| PA14_46620 |  | |  |  | 16.79 | 8.91E-73 | pyridine nucleotide-disulfide oxidoreductase |
| PA14_46890 |  | | -12.57 | 5.06E-52 | -16.11 | 5.65E-70 | short-chain dehydrogenase |
| PA14_47060 |  | | -6.50 | 1.37E-23 |  |  | short chain dehydrogenase |
| PA14_47090 |  | | -12.13 | 6.86E-25 | -10.42 | 3.17E-26 | protease |
| PA14_47360 |  | | 3.49 | 1.20E-14 |  |  | oligopeptidase |
| PA14_47370 |  | | 3.87 | 1.59E-06 | 2.53 | 8.38E-06 | signal peptidase |
| PA14_47490 |  | | 4.10 | 2.35E-17 | 2.52 | 5.93E-09 | hypothetical protein |
| PA14_47750 |  | | 3.76 | 2.17E-13 |  |  | hypothetical protein |
| PA14_48010 |  | | -2.61 | 5.98E-07 |  |  | semialdehyde dehydrogenase |
| PA14_48440 |  | | 2.65 | 9.91E-08 |  |  | NAD(P)H dehydrogenase |
| PA14_48450 |  | | -3.41 | 2.66E-11 |  |  | peptidyl-arginine deiminase |
| PA14_48600 |  | | -9.13 | 1.09E-37 | -26.00 | 6.12E-72 | AMP-binding protein |
| PA14_48760 | *ycaC* | | -23.97 | 3.65E-75 | -21.85 | 1.68E-81 | hydrolase |
| PA14_49280 |  | | 2.88 | 1.10E-10 |  |  | transglycosylase |
| PA14_49300 |  | | -6.14 | 4.61E-28 | -5.78 | 4.00E-31 | lipoxygenase |
| PA14_49360 |  | | -10.33 | 1.68E-43 | -9.53 | 5.22E-47 | glucosyl transferase |
| PA14_49800 | *gsp69* | |  |  | 3.27 | 3.01E-11 | oxidoreductase |
| PA14_50010 | *yliI* | | -4.12 | 2.74E-08 | -2.51 | 1.15E-05 | dehydrogenase |
| PA14_50310 |  | |  |  | -3.33 | 1.89E-15 | sugar nucleotidyltransferase |
| PA14_50610 |  | | -5.52 | 9.47E-22 | -2.75 | 2.60E-10 | short chain dehydrogenase |
| PA14_50660 |  | |  |  | -2.94 | 8.21E-13 | hypothetical protein |
| PA14_50840 | *dinG* | | 7.41 | 1.74E-35 | 4.19 | 1.04E-21 | ATP-dependent DNA helicase DinG |
| PA14_51040 |  | | -2.73 | 9.29E-08 | -3.04 | 4.03E-10 | oxidoreductase |
| PA14_51050 | *pcd* | | -3.00 | 6.90E-11 | -2.96 | 5.31E-12 | aldehyde dehydrogenase |
| PA14_51080 |  | |  |  | -4.38 | 2.46E-16 | dioxygenase |
| PA14_51090 |  | | -2.94 | 1.37E-09 | -5.25 | 1.78E-20 | short-chain dehydrogenase |
| PA14_51100 |  | |  |  | -3.62 | 2.01E-12 | acyl-CoA dehydrogenase |
| PA14_51110 |  | |  |  | -6.35 | 2.27E-15 | enoyl-CoA hydratase/isomerase |
| PA14_51220 |  | | 2.99 | 1.46E-12 |  |  | glycosyl transferase family protein |
| PA14_51250 | *yycJ* | | 4.38 | 4.34E-21 | 2.92 | 1.11E-13 | hypothetical protein |
| PA14_51680 |  | | 4.15 | 5.14E-18 | 2.90 | 1.62E-06 | radical activating enzyme |
| PA14_52420* | *rimO* | | -2.69 | 4.73E-09 |  |  | ribosomal protein S12 methylthiotransferase |
| PA14_52610* |  | | 2.63 | 3.14E-07 |  |  | hypothetical protein |
| PA14_52850 |  | |  |  | -2.81 | 3.42E-04 | acyl-CoA lyase subunit beta |
| PA14_52870 |  | |  |  | -3.06 | 5.02E-04 | hypothetical protein |
| PA14_53110 |  | | -3.68 | 4.19E-13 | -3.33 | 3.20E-12 | oxidoreductase |
| PA14_53230* |  | | -2.54 | 4.70E-09 | -8.83 | 1.18E-45 | oxidoreductase |
| PA14_53400 |  | |  |  | -4.85 | 3.99E-06 | oxidoreductase |
| PA14_53420 | *btuE* | | -3.74 | 4.50E-17 | -5.19 | 6.24E-31 | glutathione peroxidase |
| PA14_53470 | *ackA* | |  |  | -5.46 | 1.60E-32 | acetate kinase |
| PA14_53850 |  | | -5.72 | 2.35E-11 | -3.35 | 8.30E-09 | oxidoreductase |
| PA14_54210 |  | | 6.54 | 5.06E-33 |  |  | ATP-dependent protease |
| PA14_54620 |  | | -9.04 | 1.32E-13 | -2.77 | 1.44E-06 | aldehyde dehydrogenase |
| PA14_54630* |  | | -6.16 | 7.87E-29 | -10.02 | 7.99E-51 | acyl-CoA dehydrogenase |
| PA14_54640 |  | | -9.43 | 4.92E-43 | -24.54 | 9.34E-90 | enoyl-CoA hydratase |
| PA14_54660* |  | | -8.06 | 3.44E-38 | -25.15 | 2.12E-90 | enoyl-CoA hydratase/isomerase |
| PA14_54700 | *yjcS* | | 9.91 | 1.87E-01 |  |  | beta-lactamase |
| PA14_54830 |  | | 14.92 | 9.02E-15 | 10.65 | 1.17E-41 | transferase |
| PA14_54910 |  | | -2.93 | 4.27E-10 | -5.42 | 2.53E-23 | thioesterase |
| PA14_54920 |  | | -2.92 | 2.46E-11 |  |  | non-ribosomal peptide synthetase |
| PA14_54930 |  | | -2.90 | 5.95E-11 | -2.79 | 2.46E-12 | non-ribosomal peptide synthetase |
| PA14_54940 |  | | -3.84 | 3.75E-16 |  |  | siderophore biosynthesis enzyme |
| PA14_55200 |  | | -2.61 | 9.12E-04 | -5.43 | 1.49E-07 | amidase |
| PA14_56040 |  | | -4.87 | 2.24E-23 | -10.70 | 1.60E-54 | hypothetical protein |
| PA14_56370* |  | | -3.08 | 4.15E-12 | -2.77 | 5.31E-12 | hypothetical protein |
| PA14_56450 |  | |  |  | -2.65 | 8.20E-10 | amidase |
| PA14_57050* | *fabG* | |  |  | -2.51 | 2.62E-05 | 3-ketoacyl-ACP reductase |
| PA14_57240 |  | | 6.35 | 5.08E-31 |  |  | hypothetical protein |
| PA14_57570* |  | | 5.74 | 4.93E-28 | 4.65 | 1.87E-25 | cytochrome c reductase, iron-sulfur subun |
| PA14_57900 |  | | 3.12 | 3.40E-13 |  |  | hypothetical protein |
| PA14_58080 |  | |  |  | -3.12 | 8.18E-16 | hypothetical protein |
| PA14_58610 |  | | 2.68 | 2.58E-08 | 7.89 | 1.16E-36 | hypothetical protein |
| PA14_61150 |  | | 6.36 | 3.29E-32 |  |  | oxidoreductase |
| PA14_61600 |  | | -7.48 | 4.24E-36 | -5.35 | 2.81E-32 | hypothetical protein |
| PA14_62420 |  | | -3.53 | 4.90E-16 | -4.13 | 8.13E-24 | hypothetical protein |
| PA14_63270 | *fabG* | |  |  | -7.06 | 1.05E-10 | 3-ketoacyl-ACP reductase |
| PA14_63470 |  | | -2.61 | 1.88E-05 |  |  | methyltransferase |
| PA14_63890 |  | |  |  | 4.22 | 2.36E-20 | short-chain dehydrogenase |
| PA14_64740 | *pchA* | | -2.59 | 4.00E-09 | -2.94 | 2.53E-13 | aldehyde dehydrogenase |
| PA14_65420* |  | | 3.14 | 2.81E-13 |  |  | ribosome-associated GTPase |
| PA14_65670 |  | | 8.59 | 7.31E-39 | 3.24 | 4.70E-14 | hypothetical protein |
| PA14_66690 |  | | -4.73 | 1.19E-22 | -5.30 | 2.64E-31 | protease |
| PA14_67090 | *mdoG* | | 2.97 | 1.17E-12 |  |  | glucan biosynthesis protein G |
| PA14_67440 |  | | -4.38 | 1.73E-20 |  |  | N-formimino-L-glutamate deiminase |
| PA14_67820 |  | | 4.24 | 3.82E-19 |  |  | hypothetical protein |
| PA14_67970 |  | | 3.35 | 7.81E-13 |  |  | hypothetical protein |
| PA14_68040 | *fixR* | | -3.58 | 1.41E-03 |  |  | short-chain dehydrogenase |
| PA14_68390 |  | |  |  | 2.51 | 6.88E-10 | hydrolase |
| PA14_69040 |  | | 3.04 | 4.12E-13 |  |  | 5-formyltetrahydrofolate cyclo-ligase |
| PA14_70140* | *aldH* | | 3.83 | 1.42E-18 | 3.42 | 1.48E-18 | aldehyde dehydrogenase |
| PA14_70650 | *glcG* | |  |  | -3.14 | 1.53E-03 | GlcG protein |
| PA14_71120 |  | |  |  | -3.50 | 3.43E-03 | hypothetical protein |
| PA14_71820* | *yegQ* | | 3.37 | 4.05E-14 | -2.99 | 2.62E-11 | peptidase |
| **Related to phage, transposon, or plasmid** | | | | | | | |
| PA14_03300 |  | | 3.84 | 3.11E-14 |  |  | hypothetical protein |
| PA14_03390 |  | |  |  | 8.62 | 4.41E-35 | hypothetical protein |
| PA14_08050 |  | | -3.36 | 5.46E-07 | -2.84 | 8.42E-07 | tail fiber protein |
| PA14_08060 |  | | -3.27 | 7.09E-07 | -3.07 | 2.09E-07 | tail fiber assembly protein |
| PA14_08070 | *gpFI* | | -4.49 | 6.39E-21 | -9.68 | 5.56E-51 | phage tail sheath protein |
| PA14_08090 | *gpFII* | | -4.60 | 5.19E-21 | -9.78 | 6.01E-49 | phage tail tube protein |
| PA14_08120 |  | | -4.45 | 9.14E-11 | -9.10 | 3.28E-20 | tail length determinator protein |
| PA14_08130 | *gpU* | |  |  | -4.96 | 9.16E-16 | hypothetical protein |
| PA14_08140 | *XR2* | |  |  | -4.44 | 3.09E-14 | hypothetical protein |
| PA14_08150 | *gpD* | | -2.68 | 3.30E-07 | -5.76 | 1.64E-16 | phage late control gene D protein |
| PA14_08210 |  | | -2.59 | 2.06E-09 | -4.06 | 9.42E-22 | hypothetical protein |
| PA14_08240 |  | |  |  | -2.98 | 1.85E-14 | hypothetical protein |
| PA14_08250 |  | |  |  | -3.28 | 1.68E-12 | hypothetical protein |
| PA14_08260 |  | |  |  | -3.87 | 7.65E-18 | minor tail protein L |
| PA14_08280 |  | | -2.96 | 8.80E-11 | -3.93 | 2.96E-18 | bacteriophage protein |
| PA14_08300 | *JF1* | | -3.14 | 5.46E-13 | -3.83 | 8.06E-21 | phage-related protein, tail component |
| PA14_13890 |  | |  |  | 2.61 | 1.13E-08 | integrase |
| PA14_15400 | *repC* | | -2.67 | 3.17E-09 |  |  | replication protein, RepC |
| PA14_15530 | *trbK* | | -4.89 | 6.56E-06 | -10.75 | 1.77E-10 | entry/exclusion protein TrbK |
| PA14_15540 | *trbL* | | -2.59 | 3.74E-09 | -4.86 | 2.44E-25 | mating pair formation protein TrbL |
| PA14_15570 |  | |  |  | 5.57 | 4.24E-32 | hypothetical protein |
| PA14_28850 |  | | -10.79 | 1.36E-12 | -5.74 | 4.39E-10 | hypothetical protein |
| PA14_28870 |  | | -2.63 | 2.64E-05 | -4.28 | 7.07E-10 | hypothetical protein |
| PA14_30850 | *trbI* | | -4.43 | 3.50E-12 |  |  | TrbI-like protein |
| PA14_30860 | *trbG* | | -5.57 | 2.12E-13 | -3.13 | 4.72E-07 | TrbG-like protein |
| PA14_30870 | *trbF* | | -12.52 | 5.88E-25 | -10.72 | 5.78E-20 | conjugal transfer protein TrbF |
| PA14_30900 | *trbJ* | | -13.00 | 9.96E-33 | -12.39 | 3.53E-29 | conjugal transfer protein TrbJ |
| PA14_30910 | *trbE* | | -6.57 | 2.79E-20 | -5.34 | 1.16E-16 | conjugal transfer ATPase TrbE |
| PA14_30930 | *trbC* | |  |  | -4.73 | 5.61E-05 | TrbC-like protein |
| PA14_30940 | *trbB* | |  |  | -2.78 | 2.49E-04 | conjugal transfer protein |
| PA14_31080 |  | |  |  | -5.80 | 4.13E-04 | conjugal transfer protein |
| PA14_31100 |  | | -6.12 | 1.47E-14 |  |  | plasmid partitioning protein |
| PA14_31280 |  | | -4.48 | 1.43E-18 | -2.88 | 5.60E-12 | integrase |
| PA14_35740 | *tpnA* | | -3.43 | 2.02E-08 |  |  | transposase |
| PA14_35750 | *tpnC* | | -10.99 | 1.72E-08 | -3.04 | 3.27E-03 | tpnA repressor protein |
| PA14_35820 | *tnpS* | | -5.54 | 3.04E-13 | -2.86 | 1.04E-08 | cointegrate resolution protein S |
| PA14_35830 | *tnpT* | |  |  | 4.79 | 3.87E-28 | cointegrate resolution protein T |
| PA14_48880 | *intT* | | -3.56 | 6.43E-07 |  |  | bacteriophage integrase |
| PA14_48890 |  | | -4.07 | 1.88E-14 | -2.52 | 4.61E-08 | hypothetical protein |
| PA14_48940^‡^ | *coaB* | | -6.07 | 2.92E-28 | -17.31 | 1.69E-69 | coat protein B of bacteriophage Pf1) |
| PA14_48950 |  | | -8.04 | 2.95E-35 | -15.67 | 1.35E-61 | hypothetical protein |
| PA14_48960 |  | |  |  | -10.19 | 6.53E-09 | hypothetical protein |
| PA14_48970 |  | |  |  | -2.71 | 5.60E-09 | helix destabilizing protein of bacteriophage Pf1 |
| PA14_49030 |  | | 2.64 | 1.83E-10 | 7.11 | 6.27E-41 | hypothetical protein |
| PA14_58910 |  | | 3.30 | 4.11E-11 | 3.16 | 3.71E-11 | chromosome partitioning-like protein |
| PA14_58970 |  | |  |  | -3.01 | 1.20E-04 | hypothetical protein |
| PA14_59530 |  | | -2.90 | 2.24E-08 |  |  | hypothetical protein |
| PA14_59540 |  | | -2.92 | 5.67E-06 |  |  | hypothetical protein |
| PA14_59560 |  | | 22.42 | 6.84E-43 | 11.94 | 5.95E-25 | transposase |
| PA14_59570 |  | | 4.99 | 9.39E-14 |  |  | transposase |
| PA14_61840 | *vapI* | | -4.39 | 1.05E-08 |  |  | virulence-associated protein |
| PA14_63440 |  | |  |  | 3.58 | 5.96E-04 | bacteriophage integrase |
| PA14_63450 |  | | 2.51 | 5.13E-08 | 2.79 | 7.45E-11 | hypothetical protein |
| **Secreted Factors (toxins, enzymes, alginate)** | | | | | |  |  |
| PA14_00560 | *exoT* | | -3.64 | 1.73E-15 | -6.11 | 5.69E-32 | exoenzyme T |
| PA14_09410 | *phzG1* | | -8.70 | 3.68E-39 | -4.22 | 1.44E-23 | pyrodoxamine 5'-phosphate oxidase |
| PA14_09440 | *phzE1* | | -6.55 | 3.43E-13 | -3.34 | 3.49E-09 | phenazine biosynthesis protein PhzE |
| PA14_09480 | *phzA1* | |  |  | 2.68 | 2.75E-12 | phenazine biosynthesis protein |
| PA14_19100 | *rhlA* | | -17.59 | 1.92E-17 |  |  | rhamnosyltransferase chain A |
| PA14_19110 | *rhlB* | | -12.21 | 5.92E-52 |  |  | rhamnosyltransferase chain B |
| PA14_24560 | *pelG* | | -3.13 | 1.89E-09 |  |  | hypothetical protein |
| PA14_26020* |  | | -49.99 | 6.73E-98 | -234.88 | 2.21E-161 | aminopeptidase |
| PA14_28250 |  | | -2.60 | 7.76E-09 |  |  | secreted acid phosphatase |
| PA14_35430 | *pvcA* | |  |  | 2.87 | 1.29E-04 | pyoverdine biosynthesis protein PvcA |
| PA14_35790 |  | |  |  | 3.41 | 9.07E-19 | homospermidine synthase |
| PA14_36345 | *exoY* | | -13.47 | 4.94E-51 | -7.50 | 1.99E-38 | adenylate cyclase |
| PA14_39880 | *phzG2* | | -146.60 | 8.93E-138 | -13.92 | 5.82E-63 | pyridoxamine 5'-phosphate oxidase |
| PA14_39910 | *phzE2* | |  |  | -8.07 | 1.81E-01 | phenazine biosynthesis protein PhzE |
| PA14_39925 | *phzD2* | | -26.67 | 6.75E-16 | -5.21 | 1.25E-07 | phenazine biosynthesis protein PhzD |
| PA14_39945 | *phzC2* | | -26.76 | 3.26E-13 | -4.41 | 4.72E-06 | phenazine biosynthesis protein PhzC |
| PA14_39960 | *phzB2* | | -19.04 | 4.38E-32 | -2.73 | 9.20E-10 | phenazine biosynthesis protein |
| PA14_39970 | *phzA2* | | -17.12 | 3.62E-52 |  |  | phenazine biosynthesis protein |
| PA14_42430 | *exsC* | | -3.20 | 1.49E-13 | -3.76 | 5.05E-20 | exoenzyme S synthesis protein C |
| PA14_43070 | *hcpD* | | -7.50 | 5.68E-30 |  |  | secreted protein Hcp |
| PA14_48040 | *aprI* | | -8.64 | 6.90E-22 | -3.14 | 3.11E-11 | alkaline proteinase inhibitor AprI |
| PA14_48060 | *aprA* | | -234.08 | 1.14E-179 | -46.63 | 1.02E-123 | alkaline metalloproteinase |
| PA14_48100 | *aprE* | | -8.04 | 9.49E-27 | -5.31 | 2.65E-22 | alkaline protease secretion protein AprE |
| PA14_49510 | *pyoS3I* | | -4.01 | 9.85E-15 |  |  | immunity protein S3I structureal gene |
| PA14_49520 | *pyoS3A* | | -3.61 | 2.67E-13 |  |  | pyocin killing protein |
| PA14_49560 | *toxA* | |  |  | -3.95 | 8.36E-14 | exotoxin A |
| PA14_51530 | *exoU* | | -6.58 | 7.49E-11 | -3.05 | 5.32E-07 | ExoU |
| PA14_53250* | *cpbD* | | -55.20 | 5.45E-114 | -9.91 | 5.62E-55 | chitin-binding protein CbpD |
| PA14_53360* | *plcH* | | -2.73 | 1.25E-03 |  |  | hemolytic phospholipase C |
| PA14_53370 | *plcR* | | -2.89 | 1.26E-07 | -3.58 | 5.21E-10 | phospholipase accessory protein PlcR |
| PA14_54390 | *mucD* | |  |  | -5.56 | 4.51E-14 | serine protease MucD |
| PA14_54400 | *mucC* | |  |  | -6.70 | 5.04E-39 | positive regulator for alginate biosynthesis MucC |
| PA14_54410 | *mucB* | |  |  | -7.01 | 5.66E-42 | negative regulator for alginate biosynthesis MucB |
| PA14_54420* | *mucA* | | -2.78 | 2.71E-11 | -5.70 | 8.60E-35 | anti-sigma factor MucA |
| PA14_56560 |  | | -2.94 | 1.44E-06 |  |  | hypothetical protein |
| PA14_59220 |  | | -3.99 | 3.86E-07 |  |  | pyocin S5 |
| PA14_69480 | *algZ* | | -3.71 | 9.09E-16 | -3.50 | 1.24E-17 | alginate biosynthesis protein AlgZ/FimS |
| **Transcription, RNA processing and degradation** | | | | |  |  |  |
| PA14_00180 | *sun* | | 4.24 | 2.46E-20 |  |  | tRNA and rRNA cytosine-C5-methylases |
| PA14_05950 | *dbpA* | | 4.69 | 5.49E-22 | 3.14 | 1.54E-14 | ATP-dependent RNA helicase DbpA |
| PA14_07520* | *rpoD* | | 2.51 | 2.12E-09 |  |  | RNA polymerase sigma factor RpoD |
| PA14_07730 | *ksgA* | | 6.18 | 8.76E-29 | 2.56 | 1.60E-09 | dimethyladenosine transferase |
| PA14_08710 | *nusG* | | 9.81 | 7.34E-48 | 3.70 | 8.81E-21 | transcription antitermination protein NusG |
| PA14_08820^‡^* | *fusA1* | | 3.67 | 3.85E-18 | 4.64 | 4.23E-28 | elongation factor G |
| PA14_09115^‡^ | *rpoA* | |  |  | 4.12 | 1.81E-24 | DNA-directed RNA polymerase subunit alpha |
| PA14_11450* | *nusB* | | 5.35 | 2.12E-26 |  |  | transcription antitermination protein NusB |
| PA14_12760 |  | | 3.94 | 8.66E-18 |  |  | ATP-dependent RNA helicase |
| PA14_13340 |  | | -5.15 | 3.70E-21 |  |  | extracellular nuclease |
| PA14_14040 | *rhl* | | 3.94 | 1.41E-18 | 2.80 | 6.12E-13 | ATP-dependent RNA helicase RhlB |
| PA14_15980 | *rimM* | | 10.83 | 1.27E-41 | 5.67 | 3.54E-33 | 16S rRNA-processing protein RimM |
| PA14_15990 | *trmD* | | 11.24 | 4.16E-50 | 5.21 | 2.76E-30 | tRNA (guanine-N(1)-)-methyltransferase |
| PA14_16000 | *rplS* | | 4.53 | 5.19E-19 |  |  | 50S ribosomal protein L19 |
| PA14_16150 |  | | 4.05 | 1.22E-19 |  |  | hypothetical protein |
| PA14_21230* | *hepA* | | 10.67 | 3.06E-48 | 5.20 | 2.52E-28 | ATP-dependent helicase HepA |
| PA14_22000 | *rluA* | | 8.56 | 1.91E-19 | 5.05 | 7.45E-18 | pseudouridine synthase |
| PA14_25560* | *rne* | | 3.24 | 8.13E-15 | 4.15 | 3.78E-24 | ribonuclease E |
| PA14_27370 | *deaD* | | 60.96 | 9.97E-112 | 43.03 | 9.28E-105 | ATP-dependent RNA helicase |
| PA14_30150 | *mnmA* | | 3.19 | 2.84E-13 | 2.87 | 4.97E-13 | tRNA-specific 2-thiouridylase MnmA |
| PA14_37430 |  | | -3.06 | 1.92E-06 | 9.18 | 1.64E-15 | RNA polymerase sigma factor |
| PA14_49060 |  | |  |  | 5.32 | 1.07E-13 | hypothetical protein |
| PA14_49390 | *rrmA* | | 3.33 | 5.83E-13 |  |  | rRNA methyltransferase |
| PA14_51820* | *aspS* | | 9.44 | 1.93E-45 | 3.29 | 6.54E-17 | aspartyl-tRNA synthetase |
| PA14_52600* | *alaS* | | 5.33 | 7.73E-27 | 2.67 | 4.94E-12 | alanyl-tRNA synthetase |
| PA14_54800 | *rsuA* | | 10.39 | 3.69E-24 | 8.07 | 4.21E-27 | pseudouridylate synthase |
| PA14_62560 | *pcnB* | | 4.34 | 2.06E-21 |  |  | poly(A) polymerase |
| PA14_62710* | *pnp* | | 3.00 | 4.68E-13 | 2.63 | 2.24E-12 | polynucleotide phosphorylase |
| PA14_62730* | *truB* | | 4.92 | 5.29E-25 | 4.94 | 6.03E-29 | tRNA pseudouridine synthase B |
| PA14_62770* | *nusA* | | 10.73 | 6.59E-51 | 4.72 | 7.13E-28 | transcription elongation factor NusA |
| PA14_64000 |  | | 2.85 | 2.39E-10 |  |  | translation initiation factor Sui1 |
| PA14_64190 | *fis* | | 4.41 | 2.45E-18 | 3.98 | 1.15E-17 | DNA-binding protein Fis |
| PA14_65190* | *yjfH* | |  |  | -2.85 | 1.47E-13 | TrmH family RNA methyltransferase , group 3 |
| PA14_65410 | *orn* | | -2.84 | 3.45E-11 |  |  | oligoribonuclease |
| PA14_68710* | *tex* | | 7.07 | 6.90E-34 | 3.28 | 2.51E-15 | hypothetical protein |
| PA14_69190* | *rho* | | 22.36 | 8.26E-69 | 11.29 | 3.58E-49 | transcription termination factor Rho |
| PA14_73420 | *rnpA* | | 10.77 | 1.80E-50 | 12.60 | 3.92E-63 | ribonuclease P |
| **Transcriptional regulators** | | | | |  |  |  |
| PA14_00460 | *trpI* | | 2.94 | 6.00E-09 | 5.23 | 3.97E-22 | transcriptional regulator TrpI |
| PA14_01410 |  | | -3.69 | 1.14E-15 | -4.32 | 2.01E-22 | hypothetical protein |
| PA14_01520 |  | |  |  | 3.11 | 1.05E-08 | hypothetical protein |
| PA14_01640 |  | |  |  | 6.56 | 3.18E-31 | LysR family transcriptional regulator |
| PA14_01930* | *pcaR* | |  |  | 4.98 | 1.03E-18 | transcriptional regulator PcaR |
| PA14_01980* |  | |  |  | -3.31 | 4.87E-16 | LysR family transcriptional regulator |
| PA14_02150 |  | | 2.98 | 4.20E-10 | 2.65 | 6.02E-09 | hypothetical protein |
| PA14_02290 |  | | 2.76 | 1.94E-09 |  |  | LysR family transcriptional regulator |
| PA14_02390 |  | |  |  | 10.29 | 3.40E-46 | transcriptional regulator |
| PA14_02660 |  | |  |  | 2.81 | 9.33E-12 | LysR family transcriptional regulator |
| PA14_03580 |  | | -2.56 | 7.12E-09 |  |  | transcriptional regulator |
| PA14_03630 |  | | -2.81 | 1.88E-09 |  |  | ArsR family transcriptional regulator |
| PA14_03840* | *aguR* | | 3.73 | 3.73E-13 |  |  | transcriptional regulator AguR |
| PA14_04270 |  | |  |  | -2.94 | 9.80E-13 | transcriptional regulator |
| PA14_05290 | *algH* | | 3.18 | 4.90E-13 | 2.78 | 5.68E-12 | hypothetical protein |
| PA14_05420 | *chpD* | |  |  | -3.12 | 1.42E-11 | transcriptional regulator |
| PA14_05660 |  | | -3.73 | 5.13E-17 |  |  | transcriptional regulator |
| PA14_05850 |  | | 3.64 | 1.81E-15 |  |  | LysR family transcriptional regulator |
| PA14_05960 | *cspB* | | 2.92 | 1.12E-12 | 13.61 | 7.32E-68 | cold-shock protein |
| PA14_06180 | *fiuI* | | -4.62 | 7.89E-21 |  |  | RNA polymerase sigma factor |
| PA14_06400 |  | | 2.68 | 6.73E-08 | 6.88 | 6.48E-31 | LysR family transcriptional regulator |
| PA14_06690 | *nirG* | | 164.64 | 2.97E-22 | 55.19 | 2.71E-43 | transcriptional regulator |
| PA14_06970 |  | | -5.16 | 4.44E-22 |  |  | Cro/CI family transcriptional regulator |
| PA14_07340 |  | | 5.52 | 1.36E-16 |  |  | transcriptional regulator |
| PA14_09260 | *pchR* | | -3.60 | 5.63E-16 |  |  | transcriptional regulator PchR |
| PA14_09570 |  | |  |  | 10.27 | 4.65E-48 | LysR family transcriptional regulator |
| PA14_09910 | *lysR* | | -2.66 | 6.11E-08 |  |  | transcriptional regulator |
| PA14_09960 |  | | -2.64 | 1.50E-03 |  |  | hypothetical protein |
| PA14_10090 |  | |  |  | -2.58 | 4.15E-10 | LysR family transcriptional regulator |
| PA14_10190 |  | | 4.06 | 5.99E-15 |  |  | transcriptional regulator |
| PA14_10320 |  | | -2.57 | 6.66E-09 |  |  | transcriptional regulator |
| PA14_10660 | *hpaA* | | -2.63 | 5.69E-07 |  |  | transcriptional regulator |
| PA14_10980 |  | |  |  | 2.60 | 1.25E-09 | hypothetical protein |
| PA14_11240 |  | | -4.42 | 7.31E-17 |  |  | DNA-binding transcriptional activator FeaR |
| PA14_12140 |  | | -5.06 | 1.28E-23 |  |  | transcriptional regulator |
| PA14_12440 |  | | -3.33 | 2.10E-06 | -2.84 | 6.69E-07 | AcrR family transcriptional regulator |
| PA14_12570 |  | |  |  | 2.54 | 5.14E-10 | transcription regulator AsnC |
| PA14_13000 |  | | -3.19 | 3.24E-10 | 10.07 | 2.60E-50 | transcriptional regulator |
| PA14_13150 |  | | -3.26 | 6.34E-14 | -2.69 | 1.61E-12 | transcriptional regulator |
| PA14_14310 |  | | -4.48 | 1.05E-13 | 3.12 | 1.14E-09 | transcriptional regulator |
| PA14_14710 |  | | 4.04 | 9.33E-21 | 5.64 | 9.75E-35 | Rrf2 family protein |
| PA14_15210 |  | | 3.58 | 1.57E-13 |  |  | LysR family transcriptional regulator |
| PA14_15240 |  | |  |  | 4.65 | 7.28E-08 | LysR family transcriptional regulator |
| PA14_15480 | *merR* | | -3.85 | 3.31E-17 |  |  | transcriptional regulator MerR |
| PA14_15650 |  | |  |  | 3.54 | 8.33E-17 | hypothetical protein |
| PA14_16280 |  | | -8.76 | 7.31E-05 |  |  | transcriptional regulator |
| PA14_17380 |  | | -3.38 | 1.27E-13 |  |  | LysR family transcriptional regulator |
| PA14_17480 | *rpoS* | | -2.81 | 2.87E-11 |  |  | RNA polymerase sigma factor RpoS |
| PA14_17720 |  | | -3.32 | 9.49E-06 |  |  | LuxR family transcriptional regulator |
| PA14_17790 |  | |  |  | 4.74 | 3.30E-21 | LysR family transcriptional regulator |
| PA14_17940 | *glpR* | | 4.36 | 7.88E-08 |  |  | glycerol-3-phosphate regulon repressor |
| PA14_18230 | *fruR* | | 3.11 | 4.08E-12 |  |  | DNA-binding transcriptional regulator FruR |
| PA14_19120 | *rhlR* | | -5.41 | 1.49E-26 |  |  | transcriptional regulator RhlR |
| PA14_19380 |  | |  |  | -6.05 | 5.88E-35 | transcriptional regulator |
| PA14_19850 |  | | -4.34 | 1.01E-17 | -6.87 | 1.37E-30 | transcriptional regulator |
| PA14_19990 |  | |  |  | 3.46 | 4.20E-14 | RNA polymerase ECF-subfamily sigma-70 factor |
| PA14_20230^‡^* | *nosR* | | 161.22 | 1.90E-160 | 105.07 | 1.92E-152 | regulatory protein NosR |
| PA14_20290 | *algZ* | |  |  | 2.57 | 1.25E-10 | DNA binding-protein |
| PA14_20770* |  | | -4.77 | 5.64E-23 | -3.10 | 3.63E-16 | hypothetical protein |
| PA14_21850 |  | | -3.67 | 3.00E-08 | -2.80 | 2.02E-07 | transcriptional regulator |
| PA14_21970 |  | | -5.53 | 9.68E-20 | -3.75 | 8.25E-15 | transcriptional regulator |
| PA14_22470 |  | |  |  | -4.22 | 6.76E-14 | LysR family transcriptional regulator |
| PA14_22860 |  | | 2.55 | 4.61E-09 |  |  | transcriptional regulator |
| PA14_23060 | *hexR* | | 2.74 | 1.11E-09 |  |  | DNA-binding transcriptional regulator HexR |
| PA14_23190 |  | | 5.22 | 3.00E-09 |  |  | transcriptional regulator |
| PA14_23590 |  | |  |  | 3.43 | 2.30E-14 | transcriptional regulator |
| PA14_23730 |  | | 3.65 | 3.12E-14 | 4.79 | 1.21E-22 | LysR family transcriptional regulator |
| PA14_24920 |  | | 2.60 | 2.98E-08 |  |  | transcriptional regulator |
| PA14_26140 |  | |  |  | 5.46 | 2.14E-32 | transcriptional regulator |
| PA14_26270 |  | | 3.30 | 4.00E-09 |  |  | transcriptional regulator |
| PA14_26760 |  | | -3.19 | 2.00E-13 | -3.91 | 6.99E-21 | transcriptional regulator |
| PA14_27230 |  | | -3.73 | 1.69E-12 |  |  | MarR family transcriptional regulator |
| PA14_27280^‡^ |  | |  |  | 2.83 | 4.88E-11 | LysR family transcriptional regulator |
| PA14_27440 | *trpR* | |  |  | 2.94 | 2.35E-12 | transcriptional regulator |
| PA14_28130 |  | | -3.51 | 1.26E-10 | 3.15 | 4.10E-13 | hypothetical protein |
| PA14_28320 |  | |  |  | 3.28 | 5.01E-13 | TetR family transcriptional regulator |
| PA14_29060 |  | | -4.03 | 7.60E-15 |  |  | transcriptional regulator |
| PA14_29180 |  | | 3.39 | 6.03E-14 |  |  | AraC family transcriptional regulator |
| PA14_29260 |  | | -2.80 | 5.61E-10 |  |  | transcriptional regulator |
| PA14_29590 |  | | 2.50 | 2.21E-09 |  |  | transcriptional regulator |
| PA14_29620 | *norR* | |  |  | 3.60 | 6.02E-19 | anaerobic nitric oxide reductase transcriptional regulator |
| PA14_30200^‡^ | *cspD* | | -5.66 | 1.87E-27 |  |  | cold-shock protein CspD |
| PA14_30580 |  | | -2.67 | 3.17E-10 |  |  | LuxR family transcriptional regulator |
| PA14_30620 |  | | -13.50 | 3.62E-55 | -3.87 | 1.01E-21 | AraC family transcriptional regulator |
| PA14_30770 |  | |  |  | 5.24 | 8.01E-28 | AsnC family transcriptional regulator |
| PA14_31560 |  | |  |  | 3.67 | 1.84E-19 | LysR family transcriptional regulator |
| PA14_31630 |  | | 3.26 | 8.82E-07 | 2.73 | 1.10E-03 | LysR family transcriptional regulator |
| PA14_31780 |  | | -2.55 | 7.28E-02 | -3.99 | 1.34E-02 | LysR family transcriptional regulator |
| PA14_32060 | *xylS* | |  |  | 6.69 | 1.68E-22 | transcriptional regulator XylS |
| PA14_32190 |  | |  |  | 4.61 | 6.80E-22 | transcriptional regulator |
| PA14_32200 | *catR* | | 3.99 | 2.12E-14 | 2.98 | 6.93E-10 | transcriptional regulator CatR |
| PA14_32450 |  | |  |  | 2.54 | 1.08E-08 | AraC family transcriptional regulator |
| PA14_32700 |  | |  |  | 3.79 | 2.81E-15 | transcriptional regulator |
| PA14_32710 |  | | -4.39 | 3.65E-13 |  |  | ECF subfamily RNA polymerase sigma-70 factor |
| PA14_32940 |  | | 2.55 | 6.71E-09 |  |  | transcriptional regulator |
| PA14_33260 | *pvdS* | | -2.99 | 5.81E-06 |  |  | extracytoplasmic-function sigma-70 factor |
| PA14_33800 |  | | -2.95 | 1.84E-11 |  |  | RNA polymerase sigma factor |
| PA14_33840 |  | | -3.38 | 6.10E-10 |  |  | transcriptional regulator |
| PA14_33920 |  | |  |  | 3.42 | 1.74E-12 | transcriptional regulator |
| PA14_34150 | *sfnR* | |  |  | 16.64 | 1.42E-71 | transcriptional regulator |
| PA14_34210 | *sfnR* | |  |  | 6.29 | 3.90E-06 | transcriptional regulator |
| PA14_34690 |  | |  |  | 2.65 | 2.89E-05 | LysR family transcriptional regulator |
| PA14_34730 |  | |  |  | 82.71 | 3.90E-79 | XRE family transcriptional regulator |
| PA14_34820* |  | | -6.46 | 9.07E-27 |  |  | regulatory protein |
| PA14_34830 |  | | -7.06 | 9.05E-33 |  |  | regulatory protein |
| PA14_34880 |  | |  |  | 5.07 | 2.44E-29 | GntR family transcriptional regulator |
| PA14_35070 |  | | 4.50 | 3.28E-19 |  |  | AraC family transcriptional regulator |
| PA14_35130 | *arsR* | |  |  | 18.35 | 4.27E-30 | arsenic resistance transcriptional regulator |
| PA14_35170 | *soxR* | | -5.87 | 6.65E-26 |  |  | redox-sensing activator of soxS |
| PA14_36880 |  | |  |  | -7.35 | 3.81E-12 | ompetence-damaged protein |
| PA14_37080 |  | | 3.20 | 1.55E-13 | -7.39 | 2.33E-31 | hypothetical protein |
| PA14_37140 |  | | -3.42 | 2.09E-12 |  |  | LysR family transcriptional regulator |
| PA14_37910 |  | | 3.57 | 1.42E-09 | 3.38 | 5.01E-09 | LysR family transcriptional regulator |
| PA14_37990 |  | |  |  | 98.55 | 1.29E-63 | RNA polymerase sigma factor |
| PA14_38040 |  | | -5.21 | 4.06E-03 |  |  | AraC family transcriptional regulator |
| PA14_38250* | *yjiR* | |  |  | 5.61 | 1.94E-28 | transcriptional regulator |
| PA14_38500 |  | | -3.00 | 8.39E-09 |  |  | IclR family transcriptional regulator |
| PA14_38680 |  | |  |  | 7.29 | 1.61E-37 | LysR family transcriptional regulator |
| PA14_38930 | *glpR* | |  |  | 9.30 | 4.24E-36 | transcriptional regulator |
| PA14_39360 |  | | -3.34 | 1.34E-05 | -3.00 | 9.75E-06 | sigma-54 dependent transcriptional regulator |
| PA14_39980 | *qscR* | |  |  | 2.74 | 1.46E-09 | transcriptional regulator |
| PA14_40150 |  | |  |  | 4.22 | 5.53E-11 | transcriptional regulator |
| PA14_40380 |  | | -28.08 | 2.37E-80 |  |  | TetR family transcriptional regulator |
| PA14_40550 |  | |  |  | 19.84 | 1.58E-76 | LysR family transcriptional regulator |
| PA14_40600 |  | |  |  | 6.03 | 7.40E-30 | transcriptional regulator |
| PA14_40790 |  | |  |  | 4.67 | 1.35E-23 | transcriptional regulator |
| PA14_40910 |  | | -4.37 | 1.83E-17 |  |  | LysR family transcriptional regulatory protein |
| PA14_41800 |  | |  |  | -3.94 | 3.25E-22 | transcriptional regulator |
| PA14_42060 |  | |  |  | 3.98 | 2.53E-16 | transcriptional regulator |
| PA14_42390 | *exsA* | | -2.50 | 9.84E-09 |  |  | transcriptional regulator ExsA |
| PA14_43140 |  | | 2.98 | 4.08E-05 | 4.46 | 6.19E-11 | MarR family transcriptional regulator |
| PA14_43430 |  | |  |  | 4.23 | 4.50E-14 | IclR family transcriptional regulator |
| PA14_43480 |  | |  |  | 5.61 | 1.07E-17 | GntR family transcriptional regulator |
| PA14_44490^¶^* | *anr* | |  |  | 5.92 | 5.14E-35 | transcriptional regulator Anr |
| PA14_44980 |  | | 4.68 | 1.89E-15 | 3.15 | 1.31E-09 | TetR family transcriptional regulator |
| PA14_45950 | *rsaL* | | -4.75 | 1.17E-13 |  |  | regulatory protein RsaL |
| PA14_45960 | *lasR* | | -5.59 | 2.45E-27 |  |  | transcriptional regulator LasR |
| PA14_46170 | *yneJ* | | 2.98 | 2.20E-11 |  |  | LysR family transcriptional regulator |
| PA14_46480 |  | | 2.81 | 1.42E-05 | 40.45 | 8.80E-85 | transcriptional regulator |
| PA14_46660 |  | | 2.92 | 3.40E-08 | 6.08 | 2.30E-24 | RNA polymerase ECF-subfamily sigma-70 factor |
| PA14_46710 |  | |  |  | 3.03 | 3.59E-12 | transcriptional regulator |
| PA14_46810 |  | | -5.32 | 5.99E-23 | -7.11 | 5.77E-34 | RNA polymerase ECF-subfamily sigma-70 factor |
| PA14_47270 |  | |  |  | 2.63 | 2.39E-09 | LysR family transcriptional regulator |
| PA14_47400 |  | | -12.84 | 9.79E-13 | -3.16 | 4.25E-06 | RNA polymerase ECF-subfamily sigma-70 factor |
| PA14_47580 |  | | -3.20 | 5.87E-05 | -2.61 | 1.40E-04 | MarR family transcriptional regulator |
| PA14_47610 |  | | -3.55 | 4.69E-14 |  |  | transcriptional regulator |
| PA14_47910 |  | |  |  | 3.18 | 9.19E-06 | transcriptional regulator |
| PA14_48190 |  | | 3.18 | 2.94E-11 |  |  | transcriptional regulator |
| PA14_48390 | *yeaM* | |  |  | 3.05 | 1.16E-10 | AraC family transcriptional regulator |
| PA14_48500 |  | |  |  | 5.53 | 2.81E-24 | transcriptional regulator |
| PA14_48810 |  | | -4.60 | 1.22E-21 | -7.10 | 3.90E-39 | NAD-dependent deacetylase |
| PA14_48830 |  | | -9.75 | 5.85E-44 | -15.94 | 2.41E-71 | transcriptional regulator |
| PA14_49590 |  | |  |  | 2.62 | 1.68E-08 | transcriptional regulator |
| PA14_49630 |  | | 2.80 | 2.49E-08 | 3.26 | 1.29E-11 | transcriptional regulator |
| PA14_49700 |  | | -2.93 | 1.00E-09 |  |  | transcriptional regulator |
| PA14_50220* | *fleQ* | |  |  | -2.55 | 6.48E-04 | transcriptional regulator FleQ |
| PA14_50600 |  | | 2.81 | 7.97E-08 |  |  | transcriptional regulator |
| PA14_51205 |  | | 2.90 | 6.68E-08 |  |  | transcriptional regulator |
| PA14_51280 |  | | 4.32 | 1.74E-15 | 3.44 | 8.10E-15 | hypothetical protein |
| PA14_51840 |  | | 12.66 | 5.71E-25 | 6.93 | 3.40E-30 | cold-shock protein |
| PA14_52070 |  | | -2.66 | 3.85E-07 |  |  | transcriptional regulator |
| PA14_52530 |  | |  |  | 3.49 | 1.37E-16 | transcriptional regulator |
| PA14_52570 | *rsmA* | | -3.71 | 5.81E-17 | -4.05 | 2.29E-23 | carbon storage regulator |
| PA14_53090 |  | |  |  | 2.95 | 3.98E-09 | transcriptional regulator |
| PA14_53520 | *oruR* | |  |  | 2.80 | 1.82E-11 | transcriptional regulator OruR |
| PA14_53720 |  | | -3.46 | 7.44E-14 |  |  | transcriptional regulator |
| PA14_53730 |  | | -4.88 | 6.95E-23 |  |  | transcriptional regulator |
| PA14_53920 |  | |  |  | 6.28 | 5.80E-34 | transcriptional regulator |
| PA14_54010 |  | | -5.70 | 3.01E-25 | -4.81 | 1.86E-24 | transcriptional regulator |
| PA14_54190 | *pruR* | |  |  | 2.80 | 1.02E-11 | proline utilization regulator |
| PA14_54430 | *algU* | |  |  | -2.61 | 3.07E-12 | RNA polymerase sigma factor AlgU |
| PA14_55160 | *toxR* | | -2.65 | 2.14E-03 |  |  | transcriptional regulator ToxR |
| PA14_55730 |  | |  |  | 8.39 | 7.19E-43 | AraC family transcriptional regulator |
| PA14_56070 | *mvaT* | |  |  | -2.75 | 2.03E-13 | transcriptional regulator MvaT, P16 subunit |
| PA14_56430 |  | | -2.64 | 1.72E-07 |  |  | transcriptional regulator |
| PA14_56620 |  | |  |  | 11.93 | 2.18E-35 | hypothetical protein |
| PA14_57630 |  | | 2.71 | 3.08E-09 | 4.00 | 1.12E-18 | AraC family transcriptional regulator |
| PA14_57940 | *rpoN* | |  |  | 2.87 | 2.94E-14 | RNA polymerase factor sigma-54 |
| PA14_58380 |  | |  |  | 7.56 | 5.61E-40 | transcriptional regulator |
| PA14_60810 | *nfxB* | | 2.59 | 2.92E-01 | 3.60 | 7.34E-03 | transcriptional regulator NfxB |
| PA14_60860 | *nfxB* | | 3.18 | 4.96E-02 | 7.08 | 1.55E-04 | transcriptional regulator NfxB |
| PA14_61620 |  | | 2.66 | 2.40E-10 | -4.95 | 2.58E-17 | MerR family transcriptional regulator |
| PA14_64500 |  | | 4.36 | 1.44E-20 |  |  | transcriptional regulator |
| PA14_64640 |  | | 14.06 | 4.63E-49 | 7.40 | 1.90E-30 | TetR family transcriptional regulator |
| PA14_64700 |  | | -3.29 | 5.19E-14 |  |  | RNA polymerase sigma factor |
| PA14_64780^‡^ |  | |  |  | 5.91 | 3.59E-28 | transcriptional regulator |
| PA14_65880 | *dmsR* | | 2.77 | 1.00E-09 | 2.61 | 9.65E-10 | two-component response regulator |
| PA14_66850 | *phaD* | | -9.29 | 8.46E-09 | -12.64 | 1.66E-11 | TetR family transcriptional regulator |
| PA14_66880 | *phaI* | | -6.66 | 3.97E-32 | -15.41 | 4.22E-72 | hypothetical protein |
| PA14_67170 |  | |  |  | 4.31 | 2.08E-20 | LysR family transcriptional regulator |
| PA14_67550 |  | | -4.52 | 3.30E-05 | -2.56 | 1.32E-03 | transcriptional regulator |
| PA14_69630 | *rnk* | | 4.78 | 2.43E-23 |  |  | nucleoside diphosphate kinase regulator |
| PA14_69980 |  | |  |  | 2.90 | 3.74E-14 | transcriptional regulator |
| PA14_70080* | *lrp* | |  |  | 2.53 | 5.66E-10 | leucine-responsive regulatory protein |
| PA14_70530 |  | | 3.06 | 2.36E-11 |  |  | AraC family transcriptional regulator |
| PA14_70560* |  | | 3.54 | 1.43E-15 |  |  | LysR family transcriptional regulator |
| PA14_70710 | *glcC* | | -3.38 | 5.95E-14 |  |  | DNA-binding transcriptional regulator GlcC |
| PA14_70970 | *betI* | | 2.99 | 3.17E-09 | 3.73 | 3.20E-11 | transcriptional regulator BetI |
| PA14_71330 |  | | 7.03 | 1.89E-20 | 6.01 | 1.98E-17 | transcriptional regulator |
| PA14_71750 |  | | 21.88 | 4.85E-68 | 8.90 | 2.76E-38 | LysR family transcriptional regulator |
| PA14_72560* | *np20* | | -3.90 | 1.09E-17 |  |  | transcriptional regulator np20 |
| PA14_72650 |  | |  |  | 6.99 | 1.33E-12 | hypothetical protein |
| PA14_73190 | *glmR* | | 4.15 | 5.47E-16 | 3.01 | 1.04E-10 | GlmR transcriptional regulator |
| **Translation, post-translational modification, degradation** | | | | | | |  |
| PA14_00090* | *glyS* | | 5.53 | 6.57E-28 |  |  | glycyl-tRNA synthetase subunit beta |
| PA14_00100 | *glyQ* | | 6.50 | 2.02E-30 | 2.83 | 1.39E-11 | glycyl-tRNA synthetase subunit alpha |
| PA14_00190* | *fmt* | | 4.48 | 3.49E-21 |  |  | methionyl-tRNA formyltransferase |
| PA14_00240 |  | | 4.10 | 8.62E-17 |  |  | hypothetical protein |
| PA14_00310 |  | |  |  | -14.97 | 6.65E-64 | peptidyl-prolyl isomerase |
| PA14_04650 | *pfpI* | | -5.85 | 2.36E-28 | -19.10 | 7.73E-79 | protease PfpI |
| PA14_06000 | *clpA* | | -2.80 | 1.78E-11 | -16.66 | 2.01E-76 | ClpA/B protease ATP binding subunit |
| PA14_07560 | *rpsU* | | 26.97 | 2.34E-83 | 29.77 | 1.08E-62 | 30S ribosomal protein S21 |
| PA14_07800 | *djlA* | |  |  | -3.66 | 3.97E-19 | hypothetical protein |
| PA14_08380* |  | | 2.83 | 3.15E-10 |  |  | hypothetical protein |
| PA14_08520 | *anmK* | | 2.86 | 7.01E-12 |  |  | anhydro-N-acetylmuramic acid kinase |
| PA14_08680 | *tufB* | | 20.49 | 3.30E-75 | 5.61 | 2.22E-32 | elongation factor Tu |
| PA14_08720 | *rplK* | | 7.70 | 1.95E-39 | 6.24 | 2.56E-37 | 50S ribosomal protein L11 |
| PA14_08730^‡^ | *rplA* | | 5.64 | 8.93E-30 | 5.23 | 8.87E-32 | 50S ribosomal protein L1 |
| PA14_08740 | *rplJ* | | 7.76 | 4.94E-40 | 6.27 | 8.34E-38 | 50S ribosomal protein L10 |
| PA14_08790* | *rpsL* | | 4.54 | 3.67E-23 | 4.63 | 1.96E-27 | 30S ribosomal protein S12 |
| PA14_08810 | *rpsG* | | 5.23 | 2.83E-27 | 5.11 | 1.21E-30 | 30S ribosomal protein S7 |
| PA14_08840 | *rpsJ* | | 4.00 | 5.94E-20 | 4.67 | 8.38E-28 | 30S ribosomal protein S10 |
| PA14_08850^‡^ | *rplC* | | 4.73 | 3.40E-24 | 4.12 | 9.21E-24 | 50S ribosomal protein L3 |
| PA14_08860 | *rplD* | | 5.18 | 8.16E-26 | 6.15 | 2.15E-36 | 50S ribosomal protein L4 |
| PA14_08870 | *rplW* | | 5.27 | 8.66E-27 | 7.28 | 8.16E-42 | 50S ribosomal protein L23 |
| PA14_08880* | *rplB* | | 4.81 | 8.91E-25 | 6.98 | 1.95E-41 | 50S ribosomal protein L2 |
| PA14_08890 | *rpsS* | | 3.55 | 3.77E-14 | 6.41 | 5.26E-38 | 30S ribosomal protein S19 |
| PA14_08900* | *rplV* | | 2.68 | 5.60E-08 | 5.73 | 1.12E-34 | 50S ribosomal protein L22 |
| PA14_08910 | *rpsC* | | 3.00 | 7.77E-08 | 6.36 | 1.32E-38 | 30S ribosomal protein S3 |
| PA14_08920 | *rplP* | | 2.82 | 6.82E-05 | 5.09 | 1.02E-30 | 50S ribosomal protein L16 |
| PA14_08930 | *rpmC* | |  |  | 5.04 | 1.08E-29 | 50S ribosomal protein L29 |
| PA14_08940* | *rpsQ* | | 3.16 | 3.57E-07 | 6.11 | 1.56E-36 | 30S ribosomal protein S17 |
| PA14_08950 | *rplN* | |  |  | 3.80 | 5.10E-22 | 50S ribosomal protein L14 |
| PA14_08960 | *rplX* | |  |  | 3.59 | 2.32E-20 | 50S ribosomal protein L24 |
| PA14_08970 | *rplE* | |  |  | 3.34 | 2.06E-18 | 50S ribosomal protein L5 |
| PA14_08990 | *rpsH* | | 7.08 | 8.90E-37 | 5.39 | 4.24E-25 | 30S ribosomal protein S8 |
| PA14_09000 | *rplF* | | 8.24 | 8.05E-21 | 5.68 | 1.78E-33 | 50S ribosomal protein L6 |
| PA14_09010 | *rplR* | | 6.22 | 6.68E-25 | 5.18 | 5.30E-27 | 50S ribosomal protein L18 |
| PA14_09020^‡^* | *rpsE* | | 6.76 | 3.43E-33 | 5.59 | 1.52E-33 | 30S ribosomal protein S5 |
| PA14_09030 | *rpmD* | | 6.13 | 3.48E-31 | 6.81 | 5.84E-40 | 50S ribosomal protein L30 |
| PA14_09040 | *rplO* | | 5.73 | 3.27E-30 | 6.42 | 1.53E-38 | 50S ribosomal protein L15 |
| PA14_09080* | *rpsM* | | 3.85 | 2.72E-19 | 5.80 | 3.04E-35 | 30S ribosomal protein S13 |
| PA14_09090* | *rpsK* | | 3.64 | 1.79E-05 | 5.06 | 2.96E-30 | 30S ribosomal protein S11 |
| PA14_09100* | *rpsD* | | 2.75 | 2.31E-04 | 4.59 | 1.70E-27 | 30S ribosomal protein S4 |
| PA14_09130 | *rplQ* | |  |  | 4.92 | 1.59E-29 | 50S ribosomal protein L17 |
| PA14_09920 |  | |  |  | -2.54 | 1.69E-03 | translation initiation inhibitor |
| PA14_10420 | *tyrS* | |  |  | 6.12 | 1.20E-25 | tyrosyl-tRNA synthetase |
| PA14_12230* | *leuS* | | 6.27 | 7.23E-32 |  |  | leucyl-tRNA synthetase |
| PA14_12840 |  | | -4.64 | 1.52E-21 | -4.97 | 5.77E-27 | acetyltransferase |
| PA14_12850^‡^ |  | |  |  | -3.11 | 1.43E-14 | acetyltransferase |
| PA14_13220 |  | | -2.65 | 2.23E-06 |  |  | protein-tyrosine-phosphatase |
| PA14_13290* |  | | 33.23 | 3.09E-77 | 36.12 | 4.64E-38 | protease |
| PA14_13300 |  | | 44.24 | 2.83E-99 | 77.58 | 1.03E-20 | hypothetical protein |
| PA14_13410 | *prfC* | | 3.90 | 1.45E-17 |  |  | peptide chain release factor 3 |
| PA14_13840* |  | | 36.70 | 3.13E-11 | 7.02 | 6.19E-10 | peptidyl-prolyl cis-trans isomerase, PpiC-type |
| PA14_14440* | *valS* | | 5.21 | 3.48E-26 | 3.07 | 3.85E-15 | valyl-tRNA synthetase |
| PA14_14590 | *queA* | | 10.26 | 1.67E-45 | 3.65 | 3.31E-17 | S-adenosylmethionine--tRNA ribosyltransferase-isomerase |
| PA14_14600 | *tgt* | | 8.40 | 1.48E-39 | 4.00 | 2.44E-20 | queuine tRNA-ribosyltransferase |
| PA14_14690 |  | | 6.80 | 3.93E-33 | 5.04 | 1.34E-27 | methyltransferase |
| PA14_14930 | *engA* | |  |  | -2.76 | 7.12E-12 | GTP-binding protein EngA |
| PA14_15970 | *rpsP* | | 9.09 | 6.38E-44 | 6.14 | 2.31E-35 | 30S ribosomal protein S16 |
| PA14_16710 | *yeaZ* | | 8.93 | 8.13E-40 | 3.39 | 7.45E-15 | hypothetical protein |
| PA14_17050* | *map* | | 2.62 | 7.44E-10 | 2.51 | 1.07E-10 | methionine aminopeptidase |
| PA14_17060* | *rpsB* | | 15.21 | 9.60E-50 | 10.50 | 1.07E-55 | 30S ribosomal protein S2 |
| PA14_17070^‡^ | *tsf* | | 7.60 | 7.50E-17 | 5.01 | 1.46E-29 | elongation factor Ts |
| PA14_17350* |  | |  |  | -3.14 | 1.40E-12 | hypothetical protein |
| PA14_17440 | *truD* | | 2.97 | 4.99E-12 |  |  | tRNA pseudouridine synthase D |
| PA14_17460 | *pcm* | | 2.68 | 1.70E-10 |  |  | protein-L-isoaspartate O-methyltransferase |
| PA14_17570 |  | | -4.69 | 8.18E-23 | -4.07 | 7.81E-24 | hypothetical protein |
| PA14_17700 | *rpmE2* | | -69.58 | 7.54E-46 | -2.77 | 7.24E-08 | 50S ribosomal protein L31 |
| PA14_17710 | *rpmJ* | | -51.81 | 1.05E-19 | -3.58 | 6.02E-06 | 50S ribosomal protein L36 |
| PA14_18650* | *ydhD* | |  |  | 3.81 | 6.89E-22 | hypothetical protein |
| PA14_19050^‡^* | *metG* | | 4.28 | 7.60E-21 | 3.03 | 5.38E-15 | methionyl-tRNA synthetase |
| PA14_21030 |  | | -3.13 | 2.08E-11 |  |  | ATP-dependent Clp protease proteolytic subunit |
| PA14_21820 |  | | 5.44 | 2.57E-27 |  |  | peptidyl-prolyl cis-trans isomerase, FkbP-type |
| PA14_22450 | *ppiA* | |  |  | -4.07 | 3.65E-22 | peptidyl-prolyl cis-trans isomerase A |
| PA14_22830 | *yciO* | |  |  | -2.78 | 2.92E-12 | SUA5/yciO/yrdC family:Sua5/YciO/YrdC/YwlC family protein |
| PA14_23250 | *mtnA* | | 3.63 | 2.29E-15 |  |  | methylthioribose-1-phosphate isomerase |
| PA14_23330 | *rpsA* | | 4.49 | 1.79E-15 | 5.66 | 5.85E-34 | 30S ribosomal protein S1 |
| PA14_23560* | *gltX* | | 2.85 | 2.65E-11 |  |  | glutamyl-tRNA synthetase |
| PA14_23720 |  | | -10.78 | 5.35E-47 | -6.36 | 6.32E-37 | translation initiation inhibitor |
| PA14_23840 | *truA* | |  |  | -3.46 | 4.16E-17 | tRNA pseudouridine synthase A |
| PA14_24650* | *rmf* | | -20.70 | 7.36E-15 |  |  | ribosome modulation factor |
| PA14_25630 | *rpmF* | | 7.77 | 8.67E-10 | 7.85 | 6.94E-45 | 50S ribosomal protein L32 |
| PA14_27210^‡^ | *efp* | | 6.29 | 1.56E-31 | 6.07 | 8.30E-35 | elongation factor P |
| PA14_27980 | *yjbN* | | 9.63 | 5.76E-40 | 4.49 | 7.97E-20 | tRNA-dihydrouridine synthase A |
| PA14_28590 | *mapB* | | 3.08 | 3.28E-11 | 5.37 | 1.35E-26 | methionine aminopeptidase |
| PA14_28650* | *thrS* | | 3.72 | 7.60E-18 |  |  | threonyl-tRNA synthetase |
| PA14_28660* | *infC* | | 2.64 | 8.64E-11 | 3.62 | 1.25E-20 | translation initiation factor IF-3 |
| PA14_28670 | *rpmI* | |  |  | 3.10 | 2.48E-11 | 50S ribosomal protein L35 |
| PA14_28680 | *rplT* | |  |  | 3.00 | 6.67E-16 | 50S ribosomal protein L20 |
| PA14_28690 | *pheS* | | 3.54 | 1.54E-16 |  |  | phenylalanyl-tRNA synthetase subunit alpha |
| PA14_30210 | *clpS* | | -8.17 | 1.34E-04 |  |  | ATP-dependent Clp protease adaptor protein ClpS |
| PA14_30230* | *clpA* | | -9.43 | 1.86E-43 | -7.09 | 3.43E-42 | ATP-dependent Clp protease, ATP-binding subunit ClpA |
| PA14_30240 | *infA* | | 8.21 | 1.67E-28 | 6.30 | 1.44E-34 | translation initiation factor IF-1 |
| PA14_30260 | *ate1* | | -12.06 | 6.08E-07 | -3.41 | 7.13E-04 | arginyl-tRNA-protein transferase |
| PA14_30270 | *aat* | | 4.70 | 1.17E-18 | 2.76 | 4.55E-09 | leucyl/phenylalanyl-tRNA--protein transferase |
| PA14_32610 | *dsbG* | | -2.57 | 1.06E-08 |  |  | disulfide isomerase/thiol-disulfide oxidase |
| PA14_33990 |  | | -15.09 | 3.55E-32 | -5.43 | 4.44E-19 | ClpA/B-type protease |
| PA14_34850 |  | | -4.62 | 1.53E-20 |  |  | tRNA synthase |
| PA14_36390 |  | | -3.93 | 1.83E-11 | -5.27 | 4.73E-15 | hypothetical protein |
| PA14_37710 | *fusA2* | | -12.05 | 3.40E-20 | -14.12 | 9.44E-25 | elongation factor G |
| PA14_38090 |  | | 3.67 | 1.24E-13 | 2.85 | 5.46E-10 | pseudouridylate synthase |
| PA14_39390 |  | | -2.80 | 5.62E-07 |  |  | 30S ribosomal protein S6 modification protein |
| PA14_39710* | *nrdG* | | 3.38 | 2.95E-13 |  |  | radical SAM protein |
| PA14_40290 | *lasA* | | -431.32 | 3.68E-205 | -25.84 | 7.38E-96 | LasA protease |
| PA14_42130 |  | | -4.73 | 1.10E-21 | -6.32 | 1.96E-33 | hypothetical protein |
| PA14_42890 | *stp1* | |  |  | -4.49 | 2.84E-19 | serine/threonine phosphoprotein phosphatase Stp1 |
| PA14_44270 |  | |  |  | 2.90 | 3.14E-12 | sulfur transfer protein SirA |
| PA14_49870 |  | | -4.92 | 6.78E-14 | -3.16 | 5.54E-11 | peptide deformylase |
| PA14_51900* | *proS* | | 4.71 | 3.74E-22 | 3.53 | 2.62E-18 | prolyl-tRNA synthetase |
| PA14_56480 |  | |  |  | -4.22 | 1.74E-20 | hydrolase |
| PA14_57580 | *rpsI* | | 10.96 | 3.52E-51 | 3.98 | 2.96E-22 | 30S ribosomal protein S9 |
| PA14_57590 | *rplM* | | 19.59 | 1.75E-72 | 8.37 | 1.45E-45 | 50S ribosomal protein L13 |
| PA14_58050* | *pmbA* | |  |  | -6.45 | 7.98E-38 | PmbA protein |
| PA14_58170 | *gatC* | | 9.96 | 5.66E-40 | 6.54 | 1.72E-29 | aspartyl/glutamyl-tRNA amidotransferase subunit C |
| PA14_58180* | *gatA* | | 3.90 | 2.14E-18 | 2.78 | 6.88E-13 | aspartyl/glutamyl-tRNA amidotransferase subunit A |
| PA14_58190 | *gatB* | | 4.20 | 1.23E-20 | 4.25 | 4.34E-24 | aspartyl/glutamyl-tRNA amidotransferase subunit B |
| PA14_59960 |  | | -3.79 | 2.06E-04 |  |  | protein-disulfide isomerase |
| PA14_60210 | *rluD* | | 6.02 | 1.07E-29 | 2.76 | 3.55E-12 | pseudouridine synthase |
| PA14_60370* | *ileS* | | 2.74 | 2.50E-11 |  |  | isoleucyl-tRNA synthetase |
| PA14_60400 | *rpsT* | | 34.43 | 9.37E-93 | 15.91 | 1.94E-67 | 30S ribosomal protein S20 |
| PA14_60450 | *rpmA* | | 9.35 | 3.75E-29 | 4.87 | 3.28E-28 | 50S ribosomal protein L27 |
| PA14_60460 | *rplU* | | 14.08 | 6.00E-58 | 8.01 | 4.28E-31 | 50S ribosomal protein L21 |
| PA14_60500 | *fklB* | | -3.02 | 2.06E-12 | -3.73 | 3.48E-20 | peptidyl-prolyl cis-trans isomerase FklB |
| PA14_61220 | *rsmC* | | 7.21 | 3.50E-29 | 3.30 | 1.09E-11 | hypothetical protein |
| PA14_61700* | *prfA* | | 8.17 | 2.20E-38 | 3.16 | 2.44E-14 | peptide chain release factor 1 |
| PA14_61780* | *rplY* | | 7.99 | 1.01E-40 | 4.66 | 1.45E-27 | 50S ribosomal protein L25 |
| PA14_61790 | *pth* | | 5.01 | 2.87E-21 |  |  | peptidyl-tRNA hydrolase |
| PA14_61820* | *ychF* | | 9.20 | 2.55E-24 | 7.75 | 9.39E-42 | GTP-dependent nucleic acid-binding protein EngD |
| PA14_62510 | *yadB* | | 9.53 | 8.00E-39 | 3.67 | 1.86E-14 | glutamyl-Q tRNA(Asp) synthetase |
| PA14_62740 | *rbfA* | | 3.81 | 6.64E-18 | 2.64 | 1.08E-11 | ribosome-binding factor A |
| PA14_62760* | *infB* | | 4.68 | 1.96E-24 | 3.05 | 3.22E-16 | translation initiation factor IF-2 |
| PA14_62880 | *yhbY* | | 8.28 | 3.90E-38 | 6.06 | 3.53E-19 | hypothetical protein |
| PA14_63060 | *smpB* | | 5.42 | 4.51E-22 | 4.22 | 3.93E-21 | SsrA-binding protein |
| PA14_64140 | *prmA* | | 6.07 | 2.21E-21 |  |  | 50S ribosomal protein L11 methyltransferase |
| PA14_64180 | *yhdG* | | 3.37 | 5.02E-15 | 4.42 | 7.27E-25 | hypothetical protein |
| PA14_65150 | *rplI* | | 5.47 | 1.11E-20 | 4.66 | 4.17E-27 | 50S ribosomal protein L9 |
| PA14_65170 | *rpsR* | | 13.21 | 1.47E-56 | 12.31 | 3.55E-60 | 30S ribosomal protein S18 |
| PA14_65180* | *rpsF* | | 12.22 | 1.32E-35 | 14.68 | 2.29E-57 | 30S ribosomal protein S6 |
| PA14_66710* | *rpmE* | | 13.88 | 3.05E-57 | 7.49 | 1.43E-42 | 50S ribosomal protein L31 |
| PA14_66750* | *argS* | |  |  | -2.89 | 1.98E-14 | arginyl-tRNA synthetase |
| PA14_69000* | *pepP* | |  |  | -2.84 | 3.04E-05 | aminopeptidase |
| PA14_69400 | *dsbH* | | 6.81 | 3.32E-30 |  |  | disulfide bond formation protein |
| PA14_69840 |  | | 3.36 | 1.04E-13 | 3.14 | 6.18E-14 | magnesium chelatase |
| PA14_70010 |  | |  |  | -2.77 | 1.84E-09 | hypothetical protein |
| PA14_70180 | *rpmG* | |  |  | 3.07 | 8.84E-16 | 50S ribosomal protein L33 |
| PA14_70190 | *rpmB* | | 6.39 | 8.71E-24 | 7.71 | 1.21E-43 | 50S ribosomal protein L28 |
| PA14_72200 | *prfH* | | 15.44 | 2.34E-31 |  |  | peptide chain release factor-like protein |
| PA14_73400 | *trmE* | | 2.54 | 2.71E-09 | 2.66 | 9.43E-12 | tRNA modification GTPase TrmE |
| **Transport of small molecules** | | | |  |  |  |  |
| PA14_00850 |  | |  |  | -4.55 | 1.23E-15 | hypothetical protein |
| PA14_00860 |  | |  |  | -3.35 | 6.91E-10 | ABC transporter ATP-binding protein |
| PA14_01250 | *ychM* | |  |  | -2.65 | 7.32E-09 | sulfate transporter |
| PA14_01460 | *dctA* | |  |  | 3.62 | 1.49E-17 | C4-dicarboxylate transporter DctA |
| PA14_01560* | *phnA* | | 6.94 | 1.18E-29 | 6.47 | 1.53E-30 | hypothetical protein |
| PA14_01960 |  | |  |  | -4.61 | 4.20E-23 | RND efflux membrane fusion protein |
| PA14_01970 |  | |  |  | -4.62 | 3.43E-23 | RND efflux transporter |
| PA14_02330 | *atsC* | |  |  | 3.20 | 1.82E-07 | ABC transporter ATP-binding protein |
| PA14_02340 | *atsB* | |  |  | 2.76 | 5.74E-08 | ABC transporter permease |
| PA14_02360 | *atsR* | | 2.70 | 4.24E-08 |  |  | ABC transporter substrate-binding protein |
| PA14_02500 | *exbB1* | |  |  | 5.12 | 4.15E-22 | transport protein ExbB |
| PA14_02510 | *exbD1* | | -7.89 | 8.77E-07 |  |  | transport protein ExbD |
| PA14_02700 |  | | -2.50 | 1.01E-05 | -5.40 | 3.34E-13 | aminotransferase |
| PA14_02720 |  | |  |  | -2.82 | 3.83E-04 | hypothetical protein |
| PA14_03040 |  | | 7.24 | 3.84E-28 | 4.22 | 3.29E-08 | MFS transporter |
| PA14_03080 |  | | -3.19 | 3.22E-12 | -3.90 | 8.03E-18 | acetyltransferase |
| PA14_03650 | *cysA* | |  |  | 6.02 | 5.82E-19 | sulfate transport protein CysA |
| PA14_03670 | *cysW* | | 2.99 | 1.59E-07 | 18.90 | 2.05E-60 | sulfate transport protein CysW |
| PA14_03680 | *cysT* | | 4.39 | 2.09E-13 | 40.15 | 7.30E-93 | sulfate transport protein CysT |
| PA14_03700 | *sbp* | | 3.13 | 1.77E-10 | 63.34 | 2.41E-126 | sulfate-binding protein |
| PA14_03760 |  | | 3.87 | 7.36E-11 | 2.61 | 1.03E-05 | sodium:solute symporter |
| PA14_03855 |  | | 2.65 | 2.18E-04 |  |  | periplasmic polyamine binding protein |
| PA14_04080 | *yecS* | |  |  | -3.49 | 2.73E-16 | ABC transporter permease |
| PA14_04090 | *fliY* | |  |  | -4.80 | 6.65E-09 | ABC transporter substrate-binding protein |
| PA14_04250 |  | | 3.10 | 1.78E-06 | 2.98 | 4.85E-06 | ABC transporter ATP-binding protein |
| PA14_04340 |  | |  |  | -5.08 | 6.78E-27 | hypothetical protein |
| PA14_04370 |  | | 3.65 | 5.43E-14 |  |  | MFS transporter |
| PA14_04610 | *yicE* | | 10.49 | 8.58E-38 | 3.28 | 4.76E-10 | transporter |
| PA14_04640 |  | |  |  | -2.92 | 1.27E-11 | hypothetical protein |
| PA14_05200 | *czcD* | | -3.11 | 1.35E-06 |  |  | cation efflux system protein |
| PA14_06160 | *fiuA* | |  |  | 2.81 | 3.49E-13 | hydroxamate-type ferrisiderophore receptor |
| PA14_06230 |  | | -2.60 | 1.75E-06 | 3.41 | 1.54E-14 | permease |
| PA14_07690 | *glpE* | | 2.67 | 5.10E-07 |  |  | thiosulfate sulfurtransferase |
| PA14_07860 |  | |  |  | 14.44 | 2.94E-66 | ABC transporter ATP-binding protein |
| PA14_07870 |  | | -5.90 | 1.13E-11 |  |  | ABC transporter substrate-binding protein |
| PA14_07890 |  | | -2.63 | 1.69E-05 |  |  | ABC transporter permease |
| PA14_09160* | *bfrA* | | 4.54 | 6.07E-22 |  |  | bacterioferritin |
| PA14_09195 | *yajR* | | 5.31 | 3.40E-26 | 4.34 | 9.41E-24 | major facilitator transporter |
| PA14_09230 | *pchC* | | -6.98 | 9.04E-34 | -5.14 | 1.52E-30 | pyochelin biosynthetic protein PchC |
| PA14_09270 | *pchE* | | -10.70 | 1.82E-48 | -6.02 | 1.27E-36 | dihydroaeruginoic acid synthetase |
| PA14_09280 | *pchF* | | -21.79 | 7.99E-65 | -4.31 | 2.43E-25 | pyochelin synthetase |
| PA14_09300 | *pchH* | | -21.94 | 1.14E-74 | -3.30 | 9.21E-18 | ABC transporter ATP-binding protein |
| PA14_09320 | *pchI* | | -25.41 | 3.42E-68 | -3.64 | 2.61E-20 | ABC transporter ATP-binding protein |
| PA14_09340 | *fptA* | | -10.53 | 4.24E-48 | -3.10 | 1.37E-16 | Fe(III)-pyochelin outer membrane receptor |
| PA14_09380 |  | | -8.05 | 2.41E-38 | -3.36 | 2.43E-18 | transporter |
| PA14_09520 | *mexI* | | -24.27 | 1.56E-41 | -6.03 | 4.33E-22 | RND efflux transporter |
| PA14_09530 | *mexH* | | -49.82 | 6.14E-107 | -6.60 | 9.19E-39 | RND efflux membrane fusion protein |
| PA14_10140 | *fepG* | |  |  | -4.19 | 1.86E-15 | ferric enterobactin transport protein FepG |
| PA14_10160 | *fepD* | |  |  | -3.47 | 3.22E-12 | ferric enterobactin transport protein FepD |
| PA14_10170 | *fepB* | | -3.16 | 6.46E-07 |  |  | iron-enterobactin transporter periplasmic binding protein |
| PA14_10180 | *fepC* | | -5.51 | 2.39E-24 |  |  | ferric enterobactin transport protein FepC |
| PA14_10440 | *opdL* | |  |  | 3.00 | 1.26E-08 | porin |
| PA14_10710 |  | |  |  | -2.53 | 8.40E-06 | hypothetical protein |
| PA14_10750 | *sotB* | | 9.92 | 1.76E-31 | 3.15 | 4.75E-10 | sugar efflux transporter |
| PA14_10910 | *phlE* | |  |  | 4.14 | 1.81E-04 | major facilitator transporter |
| PA14_11530 |  | | 4.43 | 3.05E-19 | 2.95 | 5.60E-12 | hypothetical protein |
| PA14_11620 |  | | -4.28 | 9.54E-03 | -2.77 | 2.51E-02 | ABC transporter |
| PA14_11790 |  | |  |  | -7.92 | 1.04E-34 | amino acid transporter |
| PA14_12920 |  | | 7.77 | 1.92E-24 | 122.51 | 4.42E-142 | taurine ABC transporter periplasmic protein |
| PA14_12940 |  | |  |  | 35.09 | 2.61E-85 | taurine ABC transporter ATP-binding protein |
| PA14_12960 |  | |  |  | 46.94 | 1.41E-84 | taurine ABC transporter permease |
| PA14_12990 |  | |  |  | 3.71 | 1.33E-17 | choline transporter |
| PA14_13170 |  | | 18.74 | 1.80E-72 |  |  | metal transporting P-type ATPase |
| PA14_13580 |  | |  |  | -12.58 | 7.84E-34 | ABC transporter ATP-binding protein |
| PA14_13590 |  | | 2.82 | 1.36E-06 | -6.93 | 1.32E-31 | ABC transporter permease |
| PA14_13600 |  | |  |  | -4.62 | 1.24E-09 | ABC transporter substrate-binding protein |
| PA14_13610 |  | | 3.21 | 2.09E-05 |  |  | ABC transporter permease |
| PA14_13620 | *nhaP* | | 5.47 | 2.07E-25 |  |  | Na+/H+ antiporter NhaP |
| PA14_13750 | *narK1* | | 6.83 | 1.37E-05 |  |  | nitrite extrusion protein 1 |
| PA14_13770^‡^ | *narK2* | | 16.58 | 6.49E-06 |  |  | nitrite extrusion protein 2 |
| PA14_13990 |  | |  |  | 5.22 | 1.35E-30 | amino acid ABC transporter |
| PA14_14100 | *yhdW* | | -12.06 | 2.86E-10 | -9.25 | 3.26E-10 | amino acid-binding protein |
| PA14_14360 |  | |  |  | -7.48 | 1.80E-41 | sodium:sulfate symporter |
| PA14_14370 |  | | 2.97 | 1.05E-11 |  |  | ABC-transporter ATP-binding component |
| PA14_14380 |  | | 7.22 | 7.12E-31 |  |  | ABC transporter permease |
| PA14_15070 | *oprC* | | -5.39 | 2.64E-26 |  |  | outer membrane copper receptor OprC |
| PA14_15180 |  | | 3.72 | 1.77E-10 | 3.08 | 6.26E-08 | transporter |
| PA14_15190 |  | |  |  | 2.82 | 4.38E-03 | hypothetical protein |
| PA14_15200 |  | | 10.67 | 5.71E-33 | 43.79 | 6.87E-81 | hypothetical protein |
| PA14_15270 |  | |  |  | -3.04 | 8.05E-03 | hypothetical protein |
| PA14_15700 |  | | 8.65 | 1.29E-35 | 24.45 | 3.11E-81 | amino acid permease |
| PA14_15720 | *yfhD* | | 2.58 | 2.33E-09 |  |  | transglycosylase |
| PA14_15780 | *nagE* | | -3.09 | 3.20E-11 | -6.77 | 6.60E-26 | PTS system N-acetylglucosamine-specific IIBC component |
| PA14_15790 | *nagF* | | -3.40 | 9.67E-13 |  |  | phosphoenolpyruvate-protein phosphotransferase |
| PA14_15920 | *yhjE* | | 4.80 | 7.51E-16 | 2.70 | 9.19E-07 | major facilitator transporter |
| PA14_15930 | *yfjD* | | 5.50 | 3.11E-16 | 3.35 | 3.55E-13 | hemolysin |
| PA14_16030 |  | |  |  | -5.81 | 3.09E-30 | sodium/hydrogen antiporter |
| PA14_16410 |  | | -2.53 | 4.57E-06 |  |  | MFS transporter |
| PA14_16920 | *sufE* | | -3.35 | 4.80E-14 | -4.57 | 2.11E-24 | hypothetical protein |
| PA14_17250 | *yaaJ* | | 5.23 | 8.92E-25 |  |  | amino acid permease |
| PA14_17640 | *potA* | |  |  | 2.62 | 9.26E-03 | polyamine transport protein PotA |
| PA14_17980^‡^ | *glpF* | |  |  | -3.73 | 1.12E-16 | glycerol uptake facilitator protein |
| PA14_18070 |  | | 19.65 | 5.51E-71 |  |  | periplasmic metal-binding protein |
| PA14_18090 |  | | 13.73 | 2.10E-52 | 3.03 | 5.73E-11 | major facilitator subfamily transporter protein |
| PA14_18250* | *fruB* | |  |  | -2.56 | 7.94E-10 | phosphotransferase system enzyme I |
| PA14_18260 | *fruK* | |  |  | -4.66 | 3.91E-13 | 1-phosphofructokinase |
| PA14_18275 | *fruA* | |  |  | -4.75 | 1.32E-18 | phosphotransferase system, fructose-specific IIBC component |
| PA14_18600 |  | | 4.88 | 1.22E-17 | 4.55 | 8.93E-25 | ABC transporter ATP-binding protein |
| PA14_18670* | *bfrB* | | 5.79 | 2.85E-30 | 3.86 | 5.23E-22 | bacterioferritin |
| PA14_18680 | *bfd* | | -4.09 | 9.09E-19 |  |  | hypothetical protein |
| PA14_18760 | *mexE* | | 68.81 | 5.07E-26 |  |  | RND efflux membrane fusion protein |
| PA14_18780 | *mexF* | | 34.33 | 1.69E-23 |  |  | RND efflux transporter |
| PA14_18800 |  | | -4.46 | 3.65E-17 |  |  | hypothetical protein |
| PA14_19310 |  | | -9.12 | 1.48E-42 | -19.87 | 7.46E-83 | hypothetical protein |
| PA14_19500 | *ssuA* | |  |  | 82.95 | 3.02E-89 | hypothetical protein |
| PA14_19510 | *ssuC* | |  |  | 11.41 | 6.47E-16 | ABC transporter permease |
| PA14_19520 | *ssuB* | |  |  | 15.24 | 6.75E-08 | ABC transporter ATP-binding protein |
| PA14_19540 | *ssuA* | |  |  | 37.48 | 1.89E-97 | hypothetical protein |
| PA14_19570 | *ssuC* | |  |  | 27.09 | 2.85E-62 | ABC transporter permease |
| PA14_19580 | *ssuB* | | -9.72 | 1.24E-11 | 9.48 | 9.51E-50 | aliphatic sulfonates transport ATP-binding subunit |
| PA14_20010 | *hasR* | | -34.16 | 4.05E-30 | -3.20 | 7.34E-09 | heme uptake outer membrane receptor HasR |
| PA14_20020 | *hasAp* | | -4072.85 | 4.22E-302 | -372.17 | 8.38E-221 | heme acquisition protein HasAp |
| PA14_20190^‡^ | *nosD* | | 59.69 | 4.69E-112 | 21.38 | 2.04E-37 | copper ABC transporter periplasmic substrate-binding protein |
| PA14_20360 | *phnG* | |  |  | -4.60 | 3.72E-06 | phosphonate metabolism protein PhnG |
| PA14_20370 | *phnH* | | -2.59 | 3.43E-05 | -3.70 | 1.63E-07 | carbon-phosphorus lyase complex subunit |
| PA14_20380 | *phnI* | | -2.67 | 1.23E-06 | -3.12 | 3.18E-08 | hypothetical protein |
| PA14_20390 | *phnJ* | | -2.55 | 3.37E-06 | -3.58 | 1.40E-09 | hypothetical protein |
| PA14_20400 | *phnK* | | -2.99 | 2.74E-07 | -5.27 | 1.46E-12 | phosphonate C-P lyase system protein PhnK |
| PA14_20420 | *phnL* | |  |  | -4.63 | 3.80E-09 | phosphonate ABC transporter ATPase |
| PA14_20430 | *phnM* | | -2.63 | 2.04E-08 | -2.88 | 1.01E-10 | phosphonate metabolism protein |
| PA14_21140 |  | | 6.40 | 1.37E-14 | 2.72 | 5.38E-04 | ABC transporter permease |
| PA14_21150 |  | | 7.35 | 3.41E-22 | 2.60 | 6.20E-05 | ABC transporter permease |
| PA14_21300 |  | | 4.13 | 3.78E-17 | 4.30 | 3.73E-18 | MFS transporte |
| PA14_21620 | *oprP* | | -5.74 | 4.68E-14 | -3.17 | 2.46E-07 | phosphate-specific outer membrane porin OprP precursor |
| PA14_21730 | *fecA* | | 4.15 | 5.38E-08 | 12.58 | 1.31E-54 | TonB-dependent receptor |
| PA14_21770 | *sugE* | | 6.34 | 1.09E-07 | 3.26 | 3.40E-06 | DMT family permease |
| PA14_21780 |  | | 12.64 | 1.89E-29 | 4.69 | 9.99E-17 | transporter |
| PA14_21960 |  | | -11.63 | 3.91E-08 | -10.55 | 1.02E-08 | hypothetical protein |
| PA14_22440 | *bapA* | |  |  | -3.68 | 6.96E-20 | ABC transporter ATP-binding protein/permease |
| PA14_22740 |  | |  |  | 5.17 | 1.76E-09 | hypothetical protein |
| PA14_22980 | *gltB* | |  |  | -146.17 | 1.22E-168 | sugar ABC transporter substrate-binding protein |
| PA14_22990 | *gltF* | | 4.26 | 2.91E-21 | -31.27 | 6.12E-72 | ABC sugar transporter permease |
| PA14_23000 | *gltG* | | 3.80 | 2.29E-18 | -22.28 | 5.82E-36 | ABC sugar transporter permease |
| PA14_23010 | *gltK* | |  |  | -66.12 | 7.95E-129 | ABC transporter ATP-binding protein |
| PA14_23030 | *oprB* | |  |  | -59.75 | 2.59E-126 | glucose/carbohydrate outer membrane porin OprB precursor |
| PA14_23160 | *gltS* | | 3.37 | 3.44E-08 |  |  | sodium/glutamate symporter |
| PA14_23530 | *mdr* | | 5.36 | 4.85E-12 | 3.35 | 5.79E-06 | secretion protein |
| PA14_24790 | *opdQ* | |  |  | 5.41 | 1.12E-29 | outer membrane porin |
| PA14_24830 |  | |  |  | 8.40 | 5.14E-39 | glutathione S-transferase |
| PA14_25020 | *uup* | | 3.27 | 3.06E-14 |  |  | ABC transporter ATP-binding protein |
| PA14_25270 | *aroP1* | | 7.63 | 6.77E-20 | 3.96 | 1.29E-20 | aromatic amino acid transport protein AroP1 |
| PA14_25430 | *lolC* | | 5.12 | 3.22E-25 |  |  | hypothetical protein |
| PA14_25440 | *lolD* | | 2.99 | 6.05E-12 |  |  | lipoprotein releasing system, ATP-binding protein |
| PA14_25490 |  | | 4.24 | 2.47E-19 | 2.61 | 4.88E-09 | tolQ-type transport protein |
| PA14_25500 | *exbD* | | 3.81 | 2.46E-10 | 2.66 | 9.29E-11 | hypothetical protein |
| PA14_26050 |  | | -4.03 | 1.70E-08 | 2.75 | 3.23E-11 | transporter |
| PA14_26110 |  | | 73.57 | 1.50E-06 | 26.01 | 7.95E-49 | MFS transporter |
| PA14_26160 |  | | 4.82 | 9.77E-07 | 8.22 | 1.21E-25 | hypothetical protein |
| PA14_26400 |  | |  |  | 4.38 | 3.87E-05 | ABC transporter ATP-binding protein |
| PA14_27140 |  | |  |  | 3.99 | 2.92E-15 | hypothetical protein |
| PA14_27150 |  | |  |  | 4.64 | 1.30E-23 | ABC transporter ATP-binding protein |
| PA14_27430 |  | |  |  | 2.56 | 3.39E-05 | multidrug efflux MFS transporter |
| PA14_27500 | *yfbQ* | | 5.80 | 2.13E-25 | 4.86 | 5.72E-23 | aminotransferase |
| PA14_27770 | *yadG* | | 2.71 | 1.07E-09 |  |  | ABC transporter ATP-binding protein |
| PA14_27780 | *yadH* | | 3.26 | 4.53E-13 |  |  | ABC transporter permease |
| PA14_28170 | *yfdC* | | -7.07 | 1.28E-32 | -8.66 | 4.39E-45 | formate/nitrate transporter |
| PA14_29090 | *potF4* | |  |  | 3.69 | 1.65E-14 | periplasmic spermidine/putrescine-binding protein |
| PA14_29210 | *tetV* | | -7.50 | 1.51E-25 | -7.50 | 1.64E-25 | MFS transporter |
| PA14_29480 | *wzm* | |  |  | 3.89 | 4.43E-10 | ABC-2 transporter permease |
| PA14_29770 |  | | 3.07 | 3.38E-10 |  |  | transporter |
| PA14_30470 |  | |  |  | 3.48 | 5.81E-16 | periplasmic aliphatic sulfonate-binding protein |
| PA14_30520 |  | |  |  | 2.85 | 1.88E-03 | periplasmic aliphatic sulfonate-binding protein |
| PA14_30540 |  | |  |  | 3.33 | 5.42E-08 | periplasmic aliphatic sulfonate-binding protein |
| PA14_30550 | *ssuA* | |  |  | 18.89 | 6.30E-71 | periplasmic aliphatic sulfonate-binding protein |
| PA14_30570 | *potF* | |  |  | 2.61 | 6.43E-12 | periplasmic spermidine/putrescine-binding protein |
| PA14_31610 |  | |  |  | 3.01 | 1.15E-14 | TerC family protein |
| PA14_31870 |  | | 3.13 | 2.82E-12 |  |  | RND efflux membrane fusion protein |
| PA14_31890 |  | | 3.31 | 2.25E-14 |  |  | RND efflux transporter |
| PA14_33130 |  | | -4.09 | 9.29E-19 | -3.24 | 8.24E-17 | cation-transporting P-type ATPase |
| PA14_33500 | *pvdH* | | -20.06 | 9.71E-64 | -3.85 | 1.53E-21 | diaminobutyrate--2-oxoglutarate aminotransferase |
| PA14_33540 |  | |  |  | 5.91 | 1.00E-18 | ABC transporter permease |
| PA14_33550 |  | | -4.19 | 3.80E-18 | 3.10 | 9.21E-11 | ABC transporter ATP-binding protein |
| PA14_33680^‡^ | *fpvA* | | -5.54 | 1.86E-26 |  |  | ferripyoverdine receptor |
| PA14_33720 | *pvdN* | | -10.96 | 7.04E-14 |  |  | protein PvdN |
| PA14_33750 | *opmQ* | | -3.94 | 2.38E-13 |  |  | outer membrane protein |
| PA14_33780 |  | | -3.32 | 2.60E-03 |  |  | transmembrane sensor |
| PA14_33910 |  | | -31.92 | 8.73E-78 | -8.84 | 1.88E-42 | hypothetical protein |
| PA14_34270 | *metN1* | |  |  | 8.91 | 4.72E-07 | ABC transporter ATP-binding protein |
| PA14_34280 | *metQi* | |  |  | 11.65 | 1.38E-12 | hypothetical protein |
| PA14_34330* |  | | -3.76 | 8.82E-14 | -4.50 | 1.18E-17 | hypothetical protein |
| PA14_34500 |  | | -4.34 | 1.65E-12 |  |  | ABC transporter ATP-binding protein |
| PA14_34510 | *srpL* | | -5.87 | 2.97E-23 |  |  | hypothetical protein |
| PA14_34520 | *srpM* | | -5.52 | 6.18E-21 |  |  | ABC transporter permease |
| PA14_34630 | *gnuT* | | 13.14 | 6.75E-55 | -2.67 | 3.04E-08 | gluconate permease |
| PA14_34770 |  | |  |  | 20.80 | 5.97E-61 | ABC transporter substrate-binding protein |
| PA14_34780 |  | |  |  | 24.88 | 1.10E-60 | ABC transporter ATP-binding protein |
| PA14_34790 |  | |  |  | 5.71 | 9.47E-12 | ABC transporter permease |
| PA14_34800 |  | |  |  | 2.69 | 5.15E-09 | amino acid transporter LysE |
| PA14_34960 | *opbA* | | -5.05 | 2.41E-24 | -18.08 | 1.67E-64 | glucose-sensitive porin |
| PA14_34990 |  | |  |  | -10.83 | 2.33E-54 | TonB-dependent receptor |
| PA14_35110 | *arsB* | |  |  | 11.05 | 1.12E-22 | arsenite-antimonite efflux pump ArsB |
| PA14_35330 | *kguT* | |  |  | -9.14 | 8.22E-40 | 2-ketogluconate transporter |
| PA14_36120 |  | |  |  | 3.38 | 7.41E-11 | MFS transporter |
| PA14_36200 |  | |  |  | 44.00 | 5.46E-90 | ABC transporter substrate-binding protein |
| PA14_36220 | *gltJ* | |  |  | 54.76 | 8.08E-26 | amino acid permease |
| PA14_36230 | *glnP* | |  |  | 30.34 | 1.68E-24 | amino acid ABC transporter permease |
| PA14_36780 |  | | -7.81 | 9.15E-26 | -6.71 | 4.85E-23 | hypothetical protein |
| PA14_37290 |  | |  |  | -7.39 | 1.99E-07 | hypothetical protein |
| PA14_37310 |  | |  |  | -7.33 | 3.99E-09 | hypothetical protein |
| PA14_37420 |  | | -3.88 | 2.11E-11 | 8.26 | 6.54E-41 | transmembrane sensor protein |
| PA14_37440 |  | | -5.56 | 1.26E-16 | 10.22 | 4.19E-49 | MFS transporter |
| PA14_37460 |  | | -6.62 | 1.28E-20 | 9.83 | 1.21E-48 | permease |
| PA14_37490 |  | | -3.71 | 4.52E-14 | 9.31 | 1.50E-08 | TonB-dependent receptor |
| PA14_37630 |  | | 2.55 | 1.61E-07 |  |  | amino acid permease |
| PA14_37730 |  | | -3.83 | 1.56E-11 |  |  | TonB dependent receptor |
| PA14_37760 |  | | -10.25 | 1.08E-20 | 3.68 | 2.72E-20 | MFS transporter |
| PA14_37840 |  | | -2.64 | 1.35E-07 |  |  | ABC transporter ATP-binding protein |
| PA14_37880 |  | |  |  | 3.10 | 1.10E-06 | ABC transporter substrate-binding protein |
| PA14_37980 |  | |  |  | 14.63 | 6.99E-27 | Fe2+-dicitrate sensor, membrane protein |
| PA14_38110* | *ygjU* | | 4.70 | 7.08E-21 | 3.95 | 8.45E-19 | serine/threonine transporter SstT |
| PA14_38130 | *ycjJ* | | 4.78 | 2.02E-19 | 19.91 | 9.53E-73 | amino acid permease |
| PA14_38160 | *azlC* | | 8.58 | 1.19E-14 | 6.71 | 2.74E-19 | hypothetical protein |
| PA14_38220 |  | | -37.37 | 5.09E-97 | -18.22 | 4.84E-80 | hypothetical protein |
| PA14_38320 | *yfeH* | | -3.58 | 1.68E-14 |  |  | bile acid/Na+ symporter family transporter |
| PA14_38560 | *pcaK* | |  |  | -3.22 | 1.49E-09 | MFS transporter |
| PA14_38580 |  | | -5.95 | 2.47E-06 | 3.05 | 6.48E-09 | hypothetical protein |
| PA14_38730 | *yhhS* | | 11.23 | 1.33E-38 | 4.02 | 6.92E-13 | major facilitator superfamily transporter |
| PA14_39050 | *braZ* | | 9.66 | 4.93E-18 | 12.23 | 5.26E-38 | branched-chain amino acid transport carrier |
| PA14_39130* | *ybiT* | | 5.62 | 7.15E-28 | 3.81 | 3.28E-20 | ABC transporter ATP-binding protein |
| PA14_39330 | *rbsA* | |  |  | -3.31 | 3.63E-14 | ribose transporter |
| PA14_39410 |  | | -4.34 | 2.78E-07 | -2.69 | 1.30E-05 | hypothetical protein |
| PA14_39650 | *cirA* | | -35.32 | 5.67E-91 | -3.73 | 6.03E-21 | TonB-dependent receptor |
| PA14_39750 |  | |  |  | 2.77 | 2.62E-03 | amino acid permease |
| PA14_39780 | *hvn* | | -186.45 | 1.52E-158 | -99.65 | 9.89E-146 | hypothetical protein |
| PA14_40430 |  | | -9.85 | 2.72E-08 | -6.74 | 9.87E-08 | hypothetical protein |
| PA14_40860 |  | |  |  | -5.62 | 2.12E-25 | hypothetical protein |
| PA14_42010 |  | |  |  | 5.27 | 9.66E-32 | amidotransferase |
| PA14_42750 |  | | 7.12 | 8.08E-30 |  |  | MFS metabolite transporter |
| PA14_42860 |  | | -8.49 | 5.48E-40 | -17.44 | 3.30E-78 | hypothetical protein |
| PA14_43160 | *benE* | | 3.72 | 2.17E-13 |  |  | transporter |
| PA14_43370 | *kdpC* | |  |  | -3.00 | 2.25E-03 | potassium-transporting ATPase subunit C |
| PA14_43570 |  | | 10.35 | 4.47E-22 |  |  | hypothetical protein |
| PA14_43660 |  | | 3.32 | 8.21E-11 | 2.69 | 4.45E-08 | hypothetical protein |
| PA14_44440 | *fixI* | | 4.00 | 2.68E-19 |  |  | cation-transporting P-type ATPase |
| PA14_44520 | *ydgF* | | 32.90 | 7.02E-53 | 213.87 | 9.40E-167 | drug efflux transporter |
| PA14_44820* |  | | -5.26 | 2.70E-23 | 4.98 | 6.51E-29 | transthyretin family protein |
| PA14_44950 | *ygfU* | |  |  | 26.63 | 8.56E-85 | transporter |
| PA14_45100 | *muiA* | |  |  | 9.94 | 1.61E-45 | hypothetical protein |
| PA14_45110 | *cysP* | | 2.57 | 2.16E-09 | 9.62 | 1.08E-51 | sulfate-binding protein of ABC transporter |
| PA14_45340 |  | | 4.32 | 2.83E-16 |  |  | heme exporter protein |
| PA14_45350 | *ccmC* | | 3.52 | 2.59E-15 |  |  | heme exporter protein CcmC |
| PA14_45370 | *ccmB* | | 2.61 | 1.23E-08 |  |  | heme exporter protein CcmB |
| PA14_45380* | *ccmA* | | 8.99 | 5.18E-36 | 7.20 | 4.36E-19 | cytochrome c biogenesis protein CcmA |
| PA14_45970 |  | | -2.60 | 3.98E-10 | -18.05 | 3.73E-70 | cation-transporting P-type ATPase |
| PA14_45980 | *yjaB* | | 10.56 | 2.24E-32 | 2.96 | 3.22E-05 | acetyltransferase |
| PA14_46010 |  | | 4.38 | 7.36E-15 |  |  | ABC transporter ATP-binding protein |
| PA14_46640 |  | |  |  | 3.85 | 3.86E-16 | siderophore receptor |
| PA14_46700 | *ybiF* | | 11.63 | 2.38E-10 | 4.76 | 2.29E-15 | hypothetical protein |
| PA14_46910 | *ybeJ* | | -7.24 | 5.81E-35 | -7.92 | 7.05E-46 | ABC transporter substrate-binding protein |
| PA14_46920 | *gltJ* | |  |  | -6.83 | 3.97E-34 | ABC transporter permease |
| PA14_46930 | *gltK* | | -3.00 | 1.19E-11 | -6.37 | 1.10E-33 | ABC transporter permease |
| PA14_46950^‡^* | *gltL* | | -2.67 | 6.93E-10 | -8.02 | 7.37E-43 | ABC transporter ATP-binding protein |
| PA14_47380 | *hxuC* | | -3.66 | 1.62E-08 |  |  | heme utilization protein |
| PA14_47800 | *btuB* | | 5.55 | 1.30E-24 | 3.26 | 1.58E-13 | tonB-dependent receptor |
| PA14_47900 |  | |  |  | 3.64 | 5.17E-05 | MFS transporter |
| PA14_47920 |  | |  |  | 29.45 | 4.53E-59 | ABC transporter substrate-binding protein |
| PA14_47940 |  | |  |  | 2.66 | 7.95E-03 | ABC transporter permease |
| PA14_48630 |  | | -6.51 | 3.28E-23 | -10.93 | 2.86E-33 | MFS transporter |
| PA14_48680 |  | | -5.44 | 2.93E-25 | -5.57 | 3.76E-30 | hypothetical protein |
| PA14_48780 |  | |  |  | -7.60 | 1.30E-35 | hypothetical protein |
| PA14_50520^‡^ | *braC* | | -2.62 | 3.33E-10 | -3.30 | 4.78E-18 | branched-chain amino acid transport protein BraC |
| PA14_50540 | *livM* | |  |  | -3.24 | 3.24E-15 | leucine/isoleucine/valine transporter permease subunit |
| PA14_50550 | *livG* | |  |  | -3.83 | 9.50E-19 | leucine/isoleucine/valine transporter ATP-binding subunit |
| PA14_50560^‡^ | *braG* | |  |  | -6.57 | 9.55E-22 | branched-chain amino acid transport protein BraG |
| PA14_50770 |  | | 7.25 | 1.38E-08 |  |  | transporter |
| PA14_51880 | *oprD* | |  |  | -5.07 | 2.25E-30 | basic amino acid, basic peptide and imipenem outer membrane porin OprD precursor |
| PA14_52400 | *kup* | | -4.64 | 5.22E-22 | -4.58 | 3.34E-26 | potassium uptake protein Kup |
| PA14_52750* | *aotP* | |  |  | -2.67 | 5.72E-11 | arginine/ornithine transport protein AotP |
| PA14_52790 | *aotJ* | |  |  | -2.82 | 6.44E-06 | arginine/ornithine binding protein AotJ |
| PA14_52820 | *dctQ* | | -3.88 | 3.60E-13 |  |  | C4-dicarboxylate transporter |
| PA14_53050^‡^ | *aroP2* | | 6.63 | 4.15E-16 | 4.05 | 1.72E-20 | aromatic amino acid transport protein AroP2 |
| PA14_53330 |  | | 2.93 | 5.07E-11 |  |  | sulfate transport protein CysZ |
| PA14_54150^‡^ | *putP* | | 7.24 | 5.28E-36 |  |  | sodium/proline symporter PutP |
| PA14_54980 |  | | -2.71 | 6.61E-09 |  |  | poossible ABC-type transporter protein |
| PA14_55000 |  | | -5.50 | 5.64E-20 |  |  | ABC transporter periplasmic protein |
| PA14_55020 |  | | -2.80 | 1.82E-07 |  |  | ABC transporter permease |
| PA14_55770 | *pitA* | | 10.59 | 9.15E-37 | 7.15 | 7.94E-36 | phosphate transporter |
| PA14_56030 |  | | -9.66 | 4.72E-19 | -8.84 | 7.00E-21 | hypothetical protein |
| PA14_56640 |  | | 3.40 | 6.13E-09 | 3.35 | 6.98E-09 | MFS transporter |
| PA14_56680 | *feoB* | | -6.19 | 2.72E-13 | -39.04 | 2.12E-35 | ferrous iron transport protein B |
| PA14_56690 | *feoA* | | -5.95 | 5.23E-08 | -4.84 | 3.44E-08 | ferrous iron transport protein A |
| PA14_56770 |  | |  |  | 7.98 | 1.71E-13 | transporter |
| PA14_57100 | *ampG* | | 3.06 | 1.22E-12 |  |  | permease |
| PA14_57110 |  | |  |  | -4.33 | 3.72E-08 | hypothetical protein |
| PA14_57870 |  | | 4.94 | 2.88E-23 |  |  | ABC transporter permease |
| PA14_57980 |  | |  |  | 2.80 | 1.55E-13 | phosphoryl carrier protein |
| PA14_57990 |  | | -34.96 | 1.02E-93 | -22.68 | 4.48E-89 | hypothetical protein |
| PA14_58350 |  | | -2.53 | 2.07E-06 | -4.28 | 2.06E-13 | ABC transporter substrate-binding protein |
| PA14_58360 |  | |  |  | -4.34 | 8.07E-17 | ABC transporter substrate-binding protein |
| PA14_58390 |  | | 2.53 | 1.40E-08 | 4.39 | 1.11E-25 | ABC transporter substrate-binding protein |
| PA14_58410 |  | | 4.24 | 9.34E-21 |  |  | porin |
| PA14_58440 |  | |  |  | -2.68 | 1.10E-10 | ABC transporter permease |
| PA14_58450 | *dppC* | |  |  | -3.89 | 2.42E-17 | ABC transporter permease |
| PA14_58470* | *dppD* | |  |  | -7.34 | 6.08E-36 | ABC transporter ATP-binding protein |
| PA14_58490* | *dppF* | |  |  | -6.47 | 7.61E-34 | dipeptide transporter ATP-binding subunit |
| PA14_58570 | *piuA* | |  |  | 3.93 | 2.76E-19 | outer membrane ferric siderophore receptor |
| PA14_61080 | *dctP* | | 4.49 | 2.24E-18 |  |  | c4-dicarboxylate-binding protein |
| PA14_61250 | *lysP* | | 9.42 | 4.86E-37 | 6.22 | 9.90E-27 | APC family lysine-specific permease |
| PA14_61280 |  | | 4.00 | 1.83E-17 |  |  | epimerase |
| PA14_61480 | *uraA* | | -2.77 | 4.84E-07 |  |  | uracil permease |
| PA14_61560 |  | | 7.90 | 3.72E-35 |  |  | MFS transporter |
| PA14_62000 | *hitA* | | -3.43 | 3.30E-15 | -4.87 | 7.42E-29 | ferric iron-binding periplasmic protein HitA |
| PA14_62010 | *hitB* | |  |  | -3.01 | 8.95E-14 | iron ABC transporter, permease |
| PA14_62350 | *phuR* | |  |  | 3.83 | 1.15E-21 | heme/hemoglobin uptake outer membrane receptor PhuR |
| PA14_62440 | *yieG* | | 3.47 | 5.38E-15 |  |  | transporter |
| PA14_62920 | *yeaS* | | 6.40 | 2.05E-31 | 4.06 | 5.21E-06 | leucine export protein LeuE |
| PA14_63050 | *yfjG* | | -3.13 | 4.15E-13 | -2.69 | 1.94E-12 | hypothetical protein |
| PA14_63080 | *lldP* | | 6.32 | 3.98E-31 | 8.70 | 7.09E-47 | L-lactate permease |
| PA14_63230 | *yedA* | | 4.08 | 2.04E-17 |  |  | hypothetical protein |
| PA14_63310 | *smtA* | | 4.03 | 2.07E-13 |  |  | hypothetical protein |
| PA14_63730 |  | | 3.75 | 1.31E-10 |  |  | transporter |
| PA14_64270 |  | |  |  | 2.58 | 7.34E-02 | hypothetical protein |
| PA14_64310 |  | |  |  | -2.53 | 1.74E-04 | ABC transporter ATP-binding protein |
| PA14_64320 |  | | 2.54 | 6.92E-08 |  |  | hypothetical protein |
| PA14_64360 | *yncA* | | 2.77 | 4.19E-09 |  |  | hypothetical protein |
| PA14_64590 |  | | 6.99 | 5.86E-30 | 3.69 | 1.54E-09 | MFS transporter |
| PA14_64860 | *livF* | | -8.49 | 8.45E-35 | -14.00 | 1.84E-52 | ABC transporter ATP-binding protein |
| PA14_64870^‡^ | *braF* | | -4.04 | 1.30E-15 | -8.31 | 1.70E-32 | ABC transporter ATP-binding protein |
| PA14_64880^‡^ | *braE* | |  |  | -4.72 | 1.17E-13 | branched chain amino acid ABC transporter permease |
| PA14_64890^‡^ | *braD* | | -3.49 | 1.32E-10 | -2.61 | 2.29E-07 | branched chain amino acid ABC transporter permease |
| PA14_64900 |  | | -10.24 | 9.11E-45 | -2.76 | 4.63E-13 | ABC transporter substrate-binding protein |
| PA14_65250* | *hisZ* | | 3.75 | 6.61E-18 |  |  | ATP phosphoribosyltransferase |
| PA14_65920 |  | | 5.01 | 3.20E-17 | 6.37 | 2.66E-24 | hypothetical protein |
| PA14_66080 | *msbA* | | 14.59 | 5.17E-61 | 5.27 | 8.06E-30 | transport protein MsbA |
| PA14_66510 | *ynfM* | |  |  | 5.93 | 3.15E-04 | MFS transporter |
| PA14_67030 |  | | 3.86 | 4.13E-12 |  |  | ABC transporter ATP-binding protein |
| PA14_67040 |  | | 8.65 | 6.56E-36 | 2.68 | 9.81E-09 | ABC transporter permease |
| PA14_67050 |  | | 5.78 | 2.10E-26 |  |  | ABC transporter substrate-binding protein |
| PA14_67270 | *proV* | | -2.80 | 1.89E-10 |  |  | ABC transporter ATP-binding protein |
| PA14_67280 | *proW* | | -3.68 | 3.60E-15 | -2.50 | 8.09E-10 | ABC transporter permease |
| PA14_67300 | *proX* | | -2.86 | 5.14E-10 |  |  | ABC transporter substrate-binding protein |
| PA14_67310 | *hutT* | |  |  | 4.24 | 4.81E-20 | amino acid permease |
| PA14_67340 | *codB* | |  |  | 4.96 | 1.34E-26 | cytosine/purines uracil thiamine allantoin permease |
| PA14_67370 |  | | -15.16 | 6.14E-32 | -5.64 | 4.51E-21 | hypothetical protein |
| PA14_67400 |  | | -3.58 | 1.66E-14 |  |  | ABC transporter substrate-binding protein |
| PA14_67750 | *yigN* | | 9.96 | 7.96E-43 | 3.88 | 1.73E-17 | rhodanese-like domain-containing protein |
| PA14_67850 |  | | 6.43 | 2.34E-13 |  |  | ABC-type amino acid transport protein, periplasmic component |
| PA14_67860 |  | | 11.08 | 1.68E-22 | 2.58 | 2.89E-06 | ABC-type amino acid transporter |
| PA14_68060* |  | | 3.08 | 2.60E-12 |  |  | ABC transporter ATP-binding protein |
| PA14_68080 |  | | 7.99 | 1.21E-33 |  |  | ABC transporter permease |
| PA14_68090 |  | | 3.37 | 2.48E-13 |  |  | ABC transporter permease |
| PA14_68780 |  | |  |  | -8.50 | 6.78E-05 | phosphate transporter |
| PA14_68800 |  | | -4.88 | 1.82E-23 | -10.09 | 1.18E-53 | hypothetical protein |
| PA14_68890 |  | | 4.12 | 8.39E-18 | 2.56 | 1.80E-09 | iron ABC transporter, permease |
| PA14_68930 |  | | -3.42 | 2.89E-12 |  |  | permease |
| PA14_69060 | *yhhJ* | | 4.38 | 3.16E-22 | -3.53 | 1.99E-15 | ABC transporter permease |
| PA14_69070 | *yhiH* | |  |  | -10.13 | 2.57E-55 | ABC transporter ATP-binding protein/permease |
| PA14_69130 | *glpT* | | 3.74 | 3.61E-15 |  |  | sn-glycerol-3-phosphate transporter |
| PA14_69340 |  | | 6.25 | 3.58E-31 |  |  | ABC transporter ATP-binding protein |
| PA14_69570 | *corA* | | 3.42 | 1.48E-13 | 3.38 | 6.57E-15 | magnesium/cobalt transport protein |
| PA14_69590 |  | | 2.62 | 6.80E-10 |  |  | ABC-type amino acid transporter |
| PA14_69850* | *betT* | | 5.19 | 1.42E-26 |  |  | choline transporter |
| PA14_70120 |  | | 14.15 | 7.83E-55 | 4.23 | 5.13E-19 | MFS transporter |
| PA14_70160 | *bioA* | |  |  | 5.91 | 1.05E-33 | omega amino acid--pyruvate transaminase |
| PA14_70510 |  | | 3.14 | 1.60E-06 |  |  | hypothetical protein |
| PA14_70770 |  | | 6.52 | 2.22E-32 | 2.87 | 3.40E-13 | hypothetical protein |
| PA14_70810 | *pstB* | |  |  | 5.01 | 1.46E-26 | phosphate transporter ATP-binding protein |
| PA14_70830 | *pstA* | |  |  | 11.62 | 3.87E-49 | phosphate ABC transporter permease |
| PA14_70850 | *pstC* | |  |  | 15.43 | 1.23E-58 | membrane protein component of ABC phosphate transporter |
| PA14_70860 |  | |  |  | 8.67 | 1.93E-46 | hypothetical protein |
| PA14_70920 |  | | 4.18 | 6.93E-18 |  |  | major facilitator transporter |
| PA14_71000 |  | |  |  | -4.09 | 8.62E-21 | lycine betaine/L-proline ABC transporter, ATP-binding subunit |
| PA14_71030 |  | |  |  | -2.77 | 4.14E-06 | hypothetical protein |
| PA14_71410* |  | |  |  | -2.52 | 4.17E-08 | ring hydroxylating dioxygenase, alpha-subunit |
| PA14_71700 |  | |  |  | -2.70 | 1.14E-05 | GNAT family acetyltransferase |
| PA14_71710 | *mtr* | | 14.55 | 3.50E-56 |  |  | tryptophan permease |
| PA14_72230 |  | |  |  | -6.20 | 9.45E-37 | hypothetical protein |
| PA14_72260 |  | | -11.98 | 2.94E-52 | -10.89 | 5.15E-59 | hypothetical protein |
| PA14_72340 | *gltP* | | 7.98 | 5.84E-22 |  |  | glutamate/aspartate:proton symporter |
| PA14_72550 | *znuA* | | -3.75 | 6.12E-17 |  |  | adhesin |
| PA14_72620 | *metN* | | 15.20 | 3.98E-55 | 10.13 | 1.62E-44 | DL-methionine transporter ATP-binding subunit |
| PA14_72630 | *yaeE* | | 10.10 | 3.28E-43 | 5.65 | 8.64E-28 | ABC transporter permease |
| PA14_72640 |  | | 3.67 | 2.33E-17 |  |  | TonB-dependent receptor |
| PA14_72800 | *rosB* | | 3.95 | 2.52E-18 |  |  | potassium efflux transporter |
| PA14_72970* | *tonB* | | -7.59 | 2.50E-36 |  |  | TonB protein |
| PA14_73060 | *cynT* | | -89.46 | 3.84E-131 | -10.21 | 2.09E-55 | hypothetical protein |
| PA14_73110 |  | |  |  | -3.08 | 1.25E-11 | hypothetical protein |
| PA14_73160 |  | | 3.08 | 2.26E-08 |  |  | permease |
| **Two-component regulatory systems** | | | |  |  |  |  |
| PA14_00430 | *bvga* | | 5.67 | 4.46E-17 | 4.27 | 5.05E-12 | two-component response regulator |
| PA14_02250 | *cheA* | | -7.55 | 1.09E-22 | -29.29 | 3.99E-49 | two-component sensor |
| PA14_02260 | *cheY* | | -12.79 | 1.24E-09 | -5.84 | 2.46E-07 | two-component response regulator |
| PA14_03470 |  | | 2.67 | 3.89E-10 |  |  | hypothetical protein |
| PA14_03720 |  | | 4.47 | 4.09E-20 |  |  | sensory box GGDEF domain-containing protein |
| PA14_03790 |  | | -4.21 | 2.00E-18 |  |  | sensory box GGDEF domain-containing protein |
| PA14_04660 |  | | 5.78 | 6.79E-26 | 2.57 | 3.53E-08 | hypothetical protein |
| PA14_05330 | *pilH* | | 3.40 | 2.01E-15 |  |  | twitching motility protein PilH |
| PA14_06060 | *creB* | | 3.70 | 2.28E-15 |  |  | DNA-binding response regulator CreB |
| PA14_06170 | *fiuR* | | -4.55 | 1.54E-11 |  |  | transmembrane sensor |
| PA14_06310 |  | | -16.72 | 7.03E-13 | -6.13 | 7.19E-09 | ACT domain-containing protein |
| PA14_07680^‡^* | *prkA* | | -27.04 | 4.11E-84 | -42.80 | 1.30E-119 | hypothetical protein |
| PA14_09680 |  | | 2.81 | 2.07E-11 |  |  | two-component sensor |
| PA14_10700 |  | |  |  | -2.61 | 3.97E-12 | bacteriophytochrome |
| PA14_10770 |  | | -7.57 | 5.08E-36 | -12.31 | 3.75E-62 | sensor/response regulator hybrid |
| PA14_11120 | *rcsB* | | -4.16 | 1.20E-17 | -7.16 | 1.49E-34 | response regulator |
| PA14_12780 |  | | 5.11 | 5.55E-24 | 5.19 | 1.53E-27 | two-component response regulator |
| PA14_13660 |  | | 4.81 | 3.45E-11 | 4.02 | 1.46E-08 | hypothetical protein |
| PA14_13730 | *narL* | |  |  | -6.36 | 5.83E-36 | transcriptional regulator NarL |
| PA14_15290 |  | | -5.18 | 5.21E-10 |  |  | transcriptional regulator |
| PA14_16350 |  | | -4.05 | 9.63E-13 |  |  | two-component response regulator |
| PA14_19340 |  | |  |  | -2.74 | 4.81E-11 | sensor/response regulator hybrid |
| PA14_20780* |  | | -5.30 | 5.17E-26 | -6.04 | 3.59E-36 | two-component response regulator |
| PA14_21700 |  | |  |  | 5.99 | 7.64E-20 | two-component sensor |
| PA14_22370 |  | | -2.75 | 5.52E-10 |  |  | hypothetical protein |
| PA14_22760 | *cpxR* | | 3.36 | 6.59E-14 |  |  | two-component response regulator |
| PA14_22940 | *gltR* | | 4.56 | 7.23E-23 | -4.67 | 3.66E-23 | two-component response regulator GltR |
| PA14_22960 |  | | 4.58 | 1.73E-22 | -4.00 | 7.25E-18 | two-component sensor |
| PA14_24340 |  | | 2.56 | 5.25E-08 | 2.65 | 2.64E-09 | two-component sensor |
| PA14_24350 |  | | 7.27 | 2.54E-21 | 4.74 | 8.07E-13 | two-component response regulator |
| PA14_24710 |  | |  |  | 2.80 | 5.99E-10 | two-component response regulator |
| PA14_26970 |  | | -6.56 | 2.46E-30 | -3.59 | 7.98E-19 | hypothetical protein |
| PA14_27940 |  | | -2.81 | 4.93E-11 |  |  | two-component response regulator |
| PA14_27950 |  | | -3.40 | 2.65E-03 |  |  | hypothetical protein |
| PA14_29730 |  | |  |  | -4.51 | 1.83E-24 | two-component response regulator |
| PA14_30830 |  | | -30.18 | 5.13E-64 | -9.41 | 5.82E-42 | two-component response regulator |
| PA14_30840 |  | | -17.62 | 2.76E-18 | -9.30 | 5.69E-15 | signal transduction histidine kinase |
| PA14_31950 | *cztS* | | 4.69 | 2.41E-11 |  |  | two-component sensor |
| PA14_31960 | *czrR* | |  |  | 4.00 | 1.18E-13 | two-component response regulator |
| PA14_36260 |  | | 2.72 | 7.28E-10 |  |  | signal transduction protein |
| PA14_36420 |  | | -6.37 | 4.44E-19 | -5.82 | 1.07E-20 | sensor/response regulator hybrid |
| PA14_37690 |  | | -18.48 | 1.19E-66 | -10.00 | 2.51E-53 | sensory box protein |
| PA14_38970 |  | | -4.06 | 5.70E-14 |  |  | two-component sensor |
| PA14_39770 |  | | -2.68 | 8.83E-10 |  |  | regulatory protein |
| PA14_40570 |  | | -4.38 | 7.82E-19 |  |  | two-component response regulator |
| PA14_41260 |  | |  |  | 4.49 | 1.78E-20 | two-component response regulator |
| PA14_41270 |  | | 2.67 | 4.43E-08 | 4.53 | 1.80E-16 | two-component sensor |
| PA14_43340 | *kdpE* | | -2.65 | 9.36E-09 | -2.70 | 3.99E-10 | two-component response regulator KdpE |
| PA14_43350 | *kdpD* | | -3.47 | 1.52E-14 | -3.79 | 3.87E-19 | two-component sensor KdpD |
| PA14_45590 | *cheA* | |  |  | -2.93 | 4.49E-15 | two-component sensor |
| PA14_45620 | *cheY* | |  |  | -5.42 | 1.53E-31 | two-component response regulator CheY |
| PA14_46360 |  | | 2.59 | 3.51E-07 | 3.76 | 5.71E-09 | two-component response regulator |
| PA14_46370 |  | | 7.85 | 8.98E-33 | 3.57 | 5.24E-14 | two-component sensor |
| PA14_46990 |  | |  |  | -3.02 | 1.84E-12 | two-component response regulator |
| PA14_47390 |  | | -11.05 | 9.79E-21 | -3.85 | 9.05E-12 | transmembrane sensor |
| PA14_48160 |  | |  |  | -8.42 | 1.23E-44 | sensor/response regulator hybrid |
| PA14_50180 | *fleR* | | 2.79 | 3.83E-11 |  |  | two-component response regulator |
| PA14_50200 | *fleS* | | 3.91 | 1.21E-18 |  |  | two-component sensor |
| PA14_54510 |  | | 2.79 | 1.62E-06 | 2.52 | 3.25E-07 | two-component response regulator |
| PA14_55780 |  | | -55.90 | 3.43E-113 | -51.39 | 7.00E-124 | two-component sensor |
| PA14_55810 |  | | -31.81 | 1.69E-89 | -8.87 | 4.27E-50 | two-component response regulator |
| PA14_59770 | *rcsB* | | 2.58 | 3.12E-10 |  |  | two component response regulator |
| PA14_59780 | *rcsC* | |  |  | -4.14 | 8.97E-24 | kinase sensor protein |
| PA14_59790 | *pvrR* | | -4.46 | 8.02E-04 | -8.01 | 4.00E-06 | two component response regulator |
| PA14_61020 | *ankB* | | -2.89 | 3.81E-09 | -2.62 | 1.70E-08 | hypothetical protein |
| PA14_62530 | *cbrA* | |  |  | 2.88 | 1.93E-14 | two-component sensor CbrA |
| PA14_63150 | *pmrA* | |  |  | -3.44 | 3.24E-06 | two-component response regulator |
| PA14_63160 | *pmrB* | |  |  | -3.67 | 1.03E-14 | PmrB: two-component regulator system signal sensor kinase PmrB |
| PA14_63210 |  | | -19.41 | 1.71E-69 | -28.24 | 2.44E-96 | two-component response regulator |
| PA14_64050 |  | |  |  | 2.98 | 9.50E-16 | two-component response regulator |
| PA14_64410 | *ybiI* | |  |  | 6.36 | 3.20E-18 | hypothetical protein |
| PA14_67670 | *ntrB* | | 5.78 | 7.69E-05 |  |  | two-component sensor NtrB |
| PA14_67680 | *ntrC* | |  |  | -7.30 | 1.96E-40 | two-component response regulator NtrC |
| PA14_68230 |  | | 2.70 | 7.58E-11 |  |  | two-component sensor |
| PA14_68680 | *envZ* | | 2.79 | 7.96E-11 |  |  | two-component sensor EnvZ |
| PA14_69470 | *algR* | | -4.44 | 1.83E-21 | -7.86 | 4.96E-46 | alginate biosynthesis regulatory protein AlgR |
| PA14_70580 |  | | -3.02 | 9.33E-12 |  |  | hypothetical protein |
| PA14_72740 | *dctB* | | 6.99 | 2.88E-32 |  |  | two-component sensor |
| PA14_73020 |  | | -17.88 | 3.55E-23 |  |  | DksA/TraR family C4-type zinc finger protein |

**Table S2B**

| **PA14-ID** | **Gene** | **FoldChange A-30 vs P** | **p-value** | **FoldChange A-96 vs P** | **p-value** | **Function** |
| --- | --- | --- | --- | --- | --- | --- |
| **Amino acid biosynthesis and metabolism** | | | | | | |
| **Monobactam biosynthesis** | | |  |  |  |  |
| PA14_23800 | *asd* | 2.85 | 2.72E-11 |  |  | aspartate-semialdehyde dehydrogenase |
| PA14_51270 | *dapA* | 3.24 | 5.42E-14 |  |  | dihydrodipicolinate synthase |
| PA14_57710 | *cysN* | 3.45 | 8.74E-16 | 13.5 | 6.37E-66 | bifunctional sulfate adenylyltransferase subunit 1/adenylylsulfate kinase |
| PA14_57720 | *cysD* | 8.87 | 2.67E-39 | 53.98 | 3.23E-122 | sulfate adenylyltransferase subunit 2 |
| PA14_62940 | *dapB* | 5.63 | 2.53E-28 |  |  | dihydrodipicolinate reductase |
| **Biosynthesis of cofactors, prosthetic groups and carriers** | | | | | | |
| **Thiamine metabolism** | |  |  |  |  |  |
| PA14_04980 | *thiG* | 11.83 | 8.17E-47 | 3.8 | 1.52E-15 | thiazole synthase |
| PA14_11460 | *thiL* | 11.72 | 3.56E-50 | 2.6 | 7.40E-10 | thiamine monophosphate kinase |
| PA14_11550 | *dxs* | 6.65 | 2.70E-33 | 3.25 | 2.65E-16 | 1-deoxy-D-xylulose-5-phosphate synthase |
| PA14_12400 | *thiE* | 5.58 | 3.05E-25 | 2.7 | 3.60E-10 | thiamine-phosphate pyrophosphorylase |
| PA14_12410 | *thiD* | 4.85 | 1.74E-22 |  |  | phosphomethylpyrimidine kinase |
| PA14_14730 | *iscS* | 2.85 | 3.49E-12 |  |  | cysteine desulfurase |
| PA14_37830 | *iscS* |  |  | 16.19 | 1.37E-68 | pyridoxal-phosphate dependent protein |
| **Sulfur relay system** | |  |  |  |  |  |
| PA14_04970 | *thiS* | 18.01 | 7.57E-43 | 8.82 | 8.51E-23 | sulfur carrier protein ThiS |
| PA14_13230 | *moaC* |  |  | 9.44 | 2.19E-51 | molybdenum cofactor biosynthesis protein MoaC |
| PA14_13240 | *moaD* |  |  | 4.77 | 4.54E-26 | molybdopterin converting factor, small subunit |
| PA14_13250 | *moaE* |  |  | 4.19 | 1.30E-22 | molybdopterin converting factor, large subunit |
| PA14_13850 | *moaA* | 29.77 | 8.16E-09 | 5.04 | 5.84E-14 | molybdenum cofactor biosynthesis protein A |
| PA14_14730^‡^ | *iscS* | 2.85 | 3.49E-12 |  |  | cysteine desulfurase |
| PA14_30150 | *mnmA* | 3.19 | 2.84E-13 | 2.87 | 4.97E-13 | tRNA-specific 2-thiouridylase MnmA |
| PA14_37830 | *iscS* |  |  | 16.19 | 1.37E-68 | pyridoxal-phosphate dependent protein |
| PA14_44270 |  |  |  | 2.9 | 3.14E-12 | sulfur transfer protein SirA |
| PA14_65480 | *rhdA* | 5.24 | 1.37E-23 |  |  | thiosulfate sulfurtransferase |
| PA14_67580 | *thiI* | 16.34 | 1.44E-63 | 5.27 | 6.87E-28 | thiamine biosynthesis protein ThiI |
| **Riboflavin metabolism** | |  |  |  |  |  |
| PA14_11410 | *ribC* | 12.86 | 1.04E-52 | 4.8 | 1.17E-23 | riboflavin synthase subunit alpha |
| PA14_11420 | *ribB* | 7.39 | 9.66E-36 | 3.44 | 6.54E-17 | bifunctional 3,4-dihydroxy-2-butanone 4-phosphate synthase/GTP cyclohydrolase II-like protein |
| PA14_11430 | *ribH* | 6.63 | 2.14E-32 |  |  | 6,7-dimethyl-8-ribityllumazine synthase |
| PA14_19530 | *ssuE* | 3.57 | 8.62E-08 | 209.13 | 1.06E-161 | NAD(P)H-dependent FMN reductase |
| PA14_34180 | *msuE* |  |  | 38.62 | 2.12E-08 | NADH-dependent FMN reductase MsuE |
| PA14_47750 |  | 3.76 | 2.17E-13 |  |  | hypothetical protein |
| PA14_60380 | *ribF* | 5.11 | 7.79E-25 | 2.72 | 8.05E-12 | bifunctional riboflavin kinase/FMN adenylyltransferase |
| **Pantothenate and CoA biosynthesis** | | |  |  |  |  |
| PA14_04760 | *coaD* | 7.16 | 3.70E-30 | 4.32 | 1.68E-18 | phosphopantetheine adenylyltransferase |
| PA14_05770 | *dhT* |  |  | 4.17 | 9.49E-20 | phenylhydantoinase |
| PA14_62150 | *ilvH* |  |  | 2.87 | 1.10E-13 | acetolactate synthase 3 regulatory subunit |
| PA14_62160 | *ilvI* | 6.36 | 9.65E-33 | 6.3 | 4.44E-37 | acetolactate synthase 3 catalytic subunit |
| PA14_62580 | *panB* | 8 | 1.39E-35 | 3.49 | 4.28E-15 | 3-methyl-2-oxobutanoate hydroxymethyltransferase |
| PA14_62590 | *panC* | 8.25 | 1.54E-34 | 2.72 | 5.77E-09 | pantoate--beta-alanine ligase |
| PA14_62600 | *panD* | 8.61 | 1.21E-26 |  |  | aspartate alpha-decarboxylase |
| PA14_70240 | *coaC* | 6.19 | 5.36E-31 |  |  | bifunctional phosphopantothenoylcysteine decarboxylase/phosphopantothenate synthase |
| **Folate biosynthesis** | |  |  |  |  |  |
| PA14_04580 | *folA* | 4.25 | 4.42E-13 |  |  | dihydrofolate reductase |
| PA14_13230 | *moaC* |  |  | 9.44 | 2.19E-51 | molybdenum cofactor biosynthesis protein MoaC |
| PA14_13240 | *moaD* |  |  | 4.77 | 4.54E-26 | molybdopterin converting factor, small subunit |
| PA14_13250 | *moaE* |  |  | 4.19 | 1.30E-22 | molybdopterin converting factor, large subunit |
| PA14_13330 |  | 6.36 | 5.78E-29 | 14.56 | 2.60E-24 | hypothetical protein |
| PA14_13850 | *moaA* | 29.77 | 8.16E-09 | 5.04 | 5.84E-14 | molybdenum cofactor biosynthesis protein A |
| PA14_19630 | *folE1* | 3.44 | 6.65E-15 |  |  | GTP cyclohydrolase I |
| PA14_19640 | *ydgB* | 3.87 | 2.26E-17 |  |  | short chain dehydrogenase |
| PA14_25710 | *pabC* | 3.89 | 7.22E-19 |  |  | 4-amino-4-deoxychorismate lyase |
| PA14_27850 | *queF* | 6.26 | 2.21E-23 | 3.71 | 1.15E-12 | 7-cyano-7-deazaguanine reductase |
| PA14_29600 | *ptpS* | 6.85 | 5.89E-20 | 6.59 | 7.59E-26 | 6-pyruvoyl-tetrahydropterin synthase |
| PA14_51680 |  | 4.15 | 5.14E-18 | 2.9 | 1.62E-06 | radical activating enzyme |
| PA14_62570 | *folK* | 3.27 | 1.37E-13 | 2.55 | 3.29E-10 | 2-amino-4-hydroxy-6- hydroxymethyldihydropteridine pyrophosphokinase |
| PA14_62850 | *folP* | 3.41 | 8.51E-16 |  |  | dihydropteroate synthase |
| **Energy metabolism** | | | | | | |
| **Nitrogen metabolism (Denitrification)** | | | |  |  |  |
| PA14_03860 |  |  |  | 3.84 | 8.38E-22 | glutamine synthetase |
| PA14_03880 | *spuB* |  |  | 4.35 | 2.35E-25 | glutamine synthetase |
| PA14_06750 | *nirS* | 94.35 | 3.46E-113 | 16.71 | 6.65E-74 | nitrite reductase |
| PA14_06810 | *norC* | 79.36 | 1.90E-135 | 15.43 | 2.35E-72 | nitric-oxide reductase subunit C |
| PA14_06830 | *norB* | 298.74 | 1.22E-192 | 26.98 | 1.14E-34 | nitric-oxide reductase subunit B |
| PA14_09580 |  |  |  | 3.37 | 7.03E-17 | hypothetical protein |
| PA14_13750 | *narK1* | 6.83 | 1.37E-05 |  |  | nitrite extrusion protein 1 |
| PA14_13770 | *narK2* | 16.58 | 6.49E-06 |  |  | nitrite extrusion protein 2 |
| PA14_13780 | *narG* | 80.67 | 3.88E-18 | 5.37 | 2.67E-29 | respiratory nitrate reductase alpha subun |
| PA14_13800 | *narH* | 63.96 | 1.79E-27 | 7.53 | 5.45E-19 | respiratory nitrate reductase beta subuni |
| PA14_13810 | *narJ* | 91.11 | 1.28E-17 | 10.59 | 4.74E-14 | respiratory nitrate reductase delta chain |
| PA14_13830 | *narI* | 111.83 | 3.17E-14 | 10.86 | 2.00E-07 | respiratory nitrate reductase gamma chain |
| PA14_20200 | *nosZ* | 133.18 | 1.54E-149 | 47.24 | 5.88E-112 | nitrous-oxide reductase |
| PA14_38140 |  | 4.6 | 1.18E-21 | 22.53 | 1.48E-85 | glutamine synthetase |
| PA14_44240 |  | 5.8 | 9.83E-11 |  |  | glutamine synthetase |
| PA14_66570 | *gltB* | 4.42 | 2.62E-22 | 2.63 | 2.97E-12 | glutamate synthase subunit alpha |
| **Protein secretion/export apparatus** | | | | | | |
| **Protein export** | |  |  |  |  |  |
| PA14_04900 | *ftsY* | 5.75 | 1.49E-28 | 2.51 | 4.23E-10 | signal recognition particle receptor FtsY |
| PA14_08695 | *secE* | 10.47 | 8.53E-50 | 3.63 | 2.00E-14 | preprotein translocase subunit SecE |
| PA14_09050 | *secY* | 3.42 | 9.33E-17 | 4.49 | 2.19E-27 | preprotein translocase subunit SecY |
| PA14_14610 | *yajC* | 7.38 | 2.16E-35 | 3.84 | 2.23E-19 | preprotein translocase subunit YajC |
| PA14_14630 | *secD* | 9.78 | 3.20E-21 | 3.22 | 2.81E-16 | preprotein translocase subunit SecD |
| PA14_14650 | *secF* | 2.95 | 4.66E-07 |  |  | preprotein translocase subunit SecF |
| PA14_15960 | *ffh* | 5.66 | 1.49E-21 | 2.51 | 1.89E-10 | signal recognition particle protein Ffh |
| PA14_47370 |  | 3.87 | 1.59E-06 | 2.53 | 8.38E-06 | signal peptidase |
| PA14_57220 | *secA* | 3.02 | 5.04E-13 |  |  | preprotein translocase subunit SecA |
| PA14_66980 | *tatC* | 3.53 | 8.76E-16 | 2.98 | 6.01E-14 | sec-independent protein translocase TatC |
| PA14_67720 | *secB* | 5.14 | 8.22E-26 | 4.06 | 2.66E-22 | preprotein translocase subunit SecB |
| PA14_73410 | *yidC* | 13.19 | 2.38E-57 | 10.64 | 2.15E-55 | inner membrane protein translocase component YidC |
| **Sulphur metabolism** | | | | | | |
| **Sulphur metabolism** | |  |  |  |  |  |
| PA14_02340 | *atsB* |  |  | 2.76 | 5.74E-08 | ABC transporter permease |
| PA14_02360 | *atsR* | 2.7 | 4.24E-08 |  |  | ABC transporter substrate-binding protein |
| PA14_03650 | *cysA* |  |  | 6.02 | 5.82E-19 | sulfate transport protein CysA |
| PA14_03670 | *cysW* | 2.99 | 1.59E-07 | 18.9 | 2.05E-60 | sulfate transport protein CysW |
| PA14_03680 | *cysT* | 4.39 | 2.09E-13 | 40.15 | 7.30E-93 | sulfate transport protein CysT |
| PA14_03700 | *sbp* | 3.13 | 1.77E-10 | 63.34 | 2.41E-126 | sulfate-binding protein |
| PA14_06740 | *nirM* | 235.59 | 1.63E-164 | 22.93 | 3.96E-68 | cytochrome c-551 |
| PA14_07690 | *glpE* | 2.67 | 5.10E-07 |  |  | thiosulfate sulfurtransferase |
| PA14_12920 |  | 7.77 | 1.92E-24 | 122.51 | 4.42E-142 | taurine ABC transporter periplasmic protein |
| PA14_12940 |  |  |  | 35.09 | 2.61E-85 | taurine ABC transporter ATP-binding protein |
| PA14_12960 |  |  |  | 46.94 | 1.41E-84 | taurine ABC transporter permease |
| PA14_12970 | *tauD* |  |  | 32.17 | 1.31E-49 | taurine dioxygenase |
| PA14_14700 | *cysE* | 3.2 | 4.97E-14 | 3.28 | 7.25E-17 | serine O-acetyltransferase |
| PA14_19500 | *ssuA* |  |  | 82.95 | 3.02E-89 | hypothetical protein |
| PA14_19510 | *ssuC* |  |  | 11.41 | 6.47E-16 | ABC transporter permease |
| PA14_19520 | *ssuB* |  |  | 15.24 | 6.75E-08 | ABC transporter ATP-binding protein |
| PA14_19530 | *ssuE* | 3.57 | 8.62E-08 | 209.13 | 1.06E-161 | NAD(P)H-dependent FMN reductase |
| PA14_19540 | *ssuA* |  |  | 37.48 | 1.89E-97 | hypothetical protein |
| PA14_19560 | *ssuD* |  |  | 27.46 | 1.67E-82 | alkanesulfonate monooxygenase |
| PA14_19570 | *ssuC* |  |  | 27.09 | 2.85E-62 | ABC transporter permease |
| PA14_30460 |  |  |  | 15.08 | 1.29E-59 | flavin-dependent oxidoreductase |
| PA14_30490 |  |  |  | 7.45 | 4.63E-31 | lavin-dependent oxidoreductase |
| PA14_30520 |  |  |  | 2.85 | 1.88E-03 | periplasmic aliphatic sulfonate-binding protein |
| PA14_30540 |  |  |  | 3.33 | 5.42E-08 | periplasmic aliphatic sulfonate-binding protein |
| PA14_30550 | *ssuA* |  |  | 18.89 | 6.30E-71 | periplasmic aliphatic sulfonate-binding protein |
| PA14_34180 | *msuE* |  |  | 38.62 | 2.12E-08 | NADH-dependent FMN reductase MsuE |
| PA14_34190 | *msuD* |  |  | 21.49 | 1.63E-12 | methanesulfonate sulfonatase MsuD |
| PA14_34750 | *tauD* | 3.21 | 9.51E-06 | 11.46 | 1.03E-49 | taurine catabolism dioxygenase |
| PA14_40770 | *cysI* | 3.87 | 1.29E-16 | 31.97 | 4.83E-55 | sulfite reductase |
| PA14_41840 | *cysH* |  |  | 12.44 | 3.04E-55 | phosphoadenosine phosphosulfate reductase |
| PA14_45110 | *cysP* | 2.57 | 2.16E-09 | 9.62 | 1.08E-51 | sulfate-binding protein of ABC transporter |
| PA14_52210 | *cysM* | 4.62 | 1.46E-22 | 2.92 | 7.67E-14 | cysteine synthase B |
| PA14_57710 | *cysN* | 3.45 | 8.74E-16 | 13.5 | 6.37E-66 | bifunctional sulfate adenylyltransferase subunit 1/adenylylsulfate kinase |
| PA14_57720 | *cysD* | 8.87 | 2.67E-39 | 53.98 | 3.23E-122 | sulfate adenylyltransferase subunit 2 |
| PA14_65480 | *rhdA* | 5.24 | 1.37E-23 |  |  | thiosulfate sulfurtransferase |
| **RNA degradation** | | | | | | |
| **Transcription, RNA processing and degradation** | | | |  |  |  |
| PA14_14040 | *rhl* | 3.94 | 1.41E-18 | 2.8 | 6.12E-13 | ATP-dependent RNA helicase RhlB |
| PA14_20810 | *recQ* | 4.99 | 7.37E-25 |  |  | ATP-dependent DNA helicase RecQ |
| PA14_25560 | *rne* | 3.24 | 8.13E-15 | 4.15 | 3.78E-24 | ribonuclease E |
| PA14_27370 | *deaD* | 60.96 | 9.97E-112 | 43.03 | 9.28E-105 | ATP-dependent RNA helicase |
| PA14_62560 | *pcnB* | 4.34 | 2.06E-21 |  |  | poly(A) polymerase |
| PA14_62710 | *pnp* | 3 | 4.68E-13 | 2.63 | 2.24E-12 | polynucleotide phosphorylase |
| PA14_62970 | *dnaK* | 7.01 | 5.89E-36 |  |  | molecular chaperone DnaK |
| PA14_69190 | *rho* | 22.36 | 8.26E-69 | 11.29 | 3.58E-49 | transcription termination factor Rho |
| **Translation, post-translational modification, degradation** | | | | | | |
| **Aminoacyl-tRNA biosynthesis** | | |  |  |  |  |
| PA14_00090 | *glyS* | 5.53 | 6.57E-28 |  |  | glycyl-tRNA synthetase subunit beta |
| PA14_00100 | *glyQ* | 6.5 | 2.02E-30 | 2.83 | 1.39E-11 | glycyl-tRNA synthetase subunit alpha |
| PA14_00190 | *fmt* | 4.48 | 3.49E-21 |  |  | methionyl-tRNA formyltransferase |
| PA14_10420 | *tyrS* |  |  | 6.12 | 1.20E-25 | tyrosyl-tRNA synthetase |
| PA14_12230 | *leuS* | 6.27 | 7.23E-32 |  |  | leucyl-tRNA synthetase |
| PA14_14440 | *valS* | 5.21 | 3.48E-26 | 3.07 | 3.85E-15 | valyl-tRNA synthetase |
| PA14_19050 | *metG* | 4.28 | 7.60E-21 | 3.03 | 5.38E-15 | methionyl-tRNA synthetase |
| PA14_23560 | *gltX* | 2.85 | 2.65E-11 |  |  | glutamyl-tRNA synthetase |
| PA14_28650 | *thrS* | 3.72 | 7.60E-18 |  |  | threonyl-tRNA synthetase |
| PA14_28690 | *pheS* | 3.54 | 1.54E-16 |  |  | phenylalanyl-tRNA synthetase subunit alpha |
| PA14_41380 | *glnS* | 2.5 | 4.10E-09 |  |  | glutaminyl-tRNA synthetase |
| PA14_51820 | *aspS* | 9.44 | 1.93E-45 | 3.29 | 6.54E-17 | aspartyl-tRNA synthetase |
| PA14_51900 | *proS* | 4.71 | 3.74E-22 | 3.53 | 2.62E-18 | prolyl-tRNA synthetase |
| PA14_52600 | *alaS* | 5.33 | 7.73E-27 | 2.67 | 4.94E-12 | alanyl-tRNA synthetase |
| PA14_57670 | *trpS* | 4.51 | 1.77E-21 |  |  | tryptophanyl-tRNA synthetase |
| PA14_58170 | *gatC* | 9.96 | 5.66E-40 | 6.54 | 1.72E-29 | aspartyl/glutamyl-tRNA amidotransferase subunit C |
| PA14_58180 | *gatA* | 3.9 | 2.14E-18 | 2.78 | 6.88E-13 | aspartyl/glutamyl-tRNA amidotransferase subunit A |
| PA14_58190 | *gatB* | 4.2 | 1.23E-20 | 4.25 | 4.34E-24 | aspartyl/glutamyl-tRNA amidotransferase subunit B |
| PA14_60370 | *ileS* | 2.74 | 2.50E-11 |  |  | isoleucyl-tRNA synthetase |
