# Supplementary material for: RNASeq Based Transcriptional Profiling of Pseudomonas aeruginosa PA14 after Short- and Long-Term Anoxic Cultivation in Synthetic Cystic Fibrosis Sputum Medium
Source: PLoS One. 2016 Jan 28;11(1):e0147811. doi: 10.1371/journal.pone.0147811 (PMC4731081; doi:10.1371/journal.pone.0147811)
Supplement: S3 Table — (DOCX) [file pone.0147811.s006.docx]

**Table S3: Selected up-regulated functions in B-96 cells scrutinized for anoxic biofilm formation**. Single genes or genes that represent the first gene of an operon are listed that were (i) at least 10-fold up-regulated under condition B-96 *versus* P, that were (ii) not differentially abundant under condition A-30 *versus* P (Table S1), and (iii) for which insertion mutants were available from the PA14 transposon library [57].

| **PA14-ID** | **Gene** | **Fold change B-96 vs P** | **p-value** | **Function** |
| --- | --- | --- | --- | --- |
| PA14_39590 | *metE* | 126.97 | 1.17E-166 | 5- methyltetrahydropteroyltriglutamate/homocysteine S-methyltransferase |
| PA14_37990 |  | 98.55 | 1.29E-63 | RNA polymerase sigma factor |
| PA14_19490 | *lsfA* | 82.87 | 5.70E-79 | antioxidant protein |
| PA14_34730 |  | 82.71 | 3.90E-79 | XRE family transcriptional regulator |
| PA14_13010 |  | 66.08 | 2.21E-133 | hypothetical protein |
| PA14_36220 | *gltJ* | 54.76 | 8.08E-26 | amino acid permease |
| PA14_45000 | *gcl* | 52.93 | 3.98E-118 | glyoxylate carboligase |
| PA14_27370 | *deaD* | 43.03 | 9.28E-105 | ATP-dependent RNA helicase |
| **PA14_34180** | ***msuE*** | **38.62** | **2.12E-08** | **NADH-dependent FMN reductase MsuE** |
| PA14_47920 |  | 29.45 | 4.53E-59 | ABC transporter substrate-binding protein |
| **PA14_44950** | ***ygfU*** | **26.63** | **8.56E-85** | **Probable purine permease** |
| PA14_28070 |  | 21.38 | 3.01E-20 | hypothetical protein |
| PA14_34770 |  | 20.80 | 5.97E-61 | ABC transporter substrate-binding protein |
| PA14_40550 |  | 19.84 | 1.58E-76 | LysR family transcriptional regulator |
| PA14_35130 | *arsR* | 18.35 | 4.27E-30 | arsenic resistance transcriptional regulator |
| PA14_71890 | *psecoA* | 17.20 | 4.20E-73 | coenzyme A transferase |
| **PA14_46620** |  | **16.79** | **8.91E-73** | **pyridine nucleotide-disulfide oxidoreductase** |
| **PA14_30460** |  | **15.08** | **1.29E-59** | **flavin-dependent oxidoreductase** |
| PA14_07860 |  | 14.44 | 2.94E-66 | ABC transporter ATP-binding protein |
| **PA14_56620** |  | **11.93** | **2.18E-35** | **hypothetical protein** |
| PA14_34280 | *metQ-l* | 11.65 | 1.38E-12 | hypothetical protein |
| **PA14_70830** | ***pstA*** | **11.62** | **3.87E-49** | **phosphate ABC transporter permease** |
| PA14_67320 | *hutH* | 11.49 | 2.08E-51 | histidine ammonia-lyase |
| PA14_47040 | *yegH* | 11.23 | 2.23E-42 | TerC family protein |
| PA14_09570 |  | 10.27 | 4.65E-48 | LysR family transcriptional regulator |
| PA14_44560 |  | 10.21 | 2.77E-46 | flavin-containing monooxygenase |
